# Towards the Systematic Mapping and Engineering of the Protein Prenylation Machinery in *Saccharomyces cerevisiae*

**DOI:** 10.1371/journal.pone.0120716

**Published:** 2015-03-13

**Authors:** Viktor Stein, Marta H. Kubala, Jason Steen, Sean M. Grimmond, Kirill Alexandrov

**Affiliations:** Institute for Molecular Bioscience, The University of Queensland, St Lucia, Queensland, Australia; University of South Florida College of Medicine, UNITED STATES

## Abstract

Protein prenylation is a widespread and highly conserved eukaryotic post-translational modification that endows proteins with the ability to reversibly attach to intracellular membranes. The dynamic interaction of prenylated proteins with intracellular membranes is essential for their signalling functions and is frequently deregulated in disease processes such as cancer. As a result, protein prenylation has been pharmacologically targeted by numerous drug discovery programs, albeit with limited success. To a large extent, this can be attributed to an insufficient understanding of the interplay of different protein prenyltransferases and the combinatorial diversity of the prenylatable sequence space. Here, we report a high-throughput, growth-based genetic selection assay in *Saccharomyces cerevisiae* based on the Ras Recruitment System which, for the first time, has allowed us to create a comprehensive map of prenylatable protein sequences in *S*. *cerevisiae*. We demonstrate that potential prenylatable space is sparsely (6.2%) occupied leaving room for creation of synthetic orthogonal prenylatable sequences. To experimentally demonstrate that, we used the developed platform to engineer mutant farnesyltransferases that efficiently prenylate substrate motives that are not recognised by endogenous protein prenyltransferases. These uncoupled mutants can now be used as starting points for the systematic engineering of the eukaryotic protein prenylation machinery.

## Introduction

Protein prenylation is a widespread post-translational modification (PTM) in eukaryotic cells which is highly conserved from yeast to mammals [1–5]. It is mediated by three different protein prenyl-transferases (PPTase) that catalyse the transfer of an isoprenoid moiety to the C-terminus of their protein substrate increasing their affinity for intracellular membranes. Two of these enzymes, farnesyl-transferase (FTase) and geranylgeranyl-transferase I (GGTase I) recognise a peptide motif at the C-terminus of their protein substrate referred to as the CaaX-box motif [1,5]. Here, ‘C’ represents Cys which accepts the isoprenoid-moiety while ‘a’ represents aliphatic residues and ‘X’ refers to residues in the anchoring C-terminal position. The anchoring residue X also determines whether a protein is prenylated by either FTase or GGTase I resulting in the transfer of a farnesyl- or geranylgeranyl moiety, respectively. In mammalian cells, more than 200 proteins have experimentally been shown to be prenylated either by FTase or GGTase I. However, bioinformatics analysis of human genome identified more than 600 potentially prenylatable ORFs that contain a Cys residue four amino acids away from the C-terminus [6,7].

Following prenylation, effector proteins then undergo further post-translational processing at the endoplasmic reticulum (ER) where the three terminal amino acids-aaX are removed by a carboxypeptidase and the C-terminal carboxylate is methyl-esterfied by a carboxymethyltransferase [8]. This enhances the C-terminal hydrophobicity and increases the residence time of prenylated proteins in intracellular membranes. Here, they often exert key regulatory roles in signal transduction pathways that originate at the cell surface: e.g. FTase prenylates members of the Ras family of small GTPases as well as the γ-subunit of heterotrimeric G-proteins [2,9]. Conversely, GGTase I prenylates members of the Rho family of small GTPases regulating cytoskeletal remodelling and cell polarity [10]. Additional targets include nuclear lamins which confer structural integrity to the nuclear envelope and peroxisomal proteins. Prenylated proteins involved in cellular signalling frequently undergo further PTMs: e.g. members of the Ras family of small GTPases including yeast Ras, H-Ras, N-Ras and K-Ras-4A are C-palmitoylated at defined Cys residues in the hypervariable region (HVR) just N-terminal to the CaaX-box motif [9,11,12]. As C-palmitoylation is reversible, it can dynamically control the subcellular trafficking, localisation and thus protein function [11]. Similarly, the HVR of K-Ras4B has been shown to be phosphorylated and thus modulate membrane affinity, trafficking, localisation and function of K-Ras4B [13,14].

Due to the critical role of prenylated proteins, such as K-Ras, in cancer, both FTase and GGTase I have been targeted by numerous drug development efforts [15,16]. While FTase inhibitors have shown beneficial effects in pre-clinical models [15], their success did not readily translate into the clinic [16]. Notably, beneficial effects are frequently independent of K-Ras mutational status and thus likely acting through alternative pathways [17]. A detailed mechanistic understanding how FTase inhibitors exert their effect has therefore remained elusive to date. At least in parts, this is due to the experimental difficulties associated with studying the potentially very large combinatorial diversity of prenylatable sequence space. This analysis is further complicated by the insufficiently developed methodologies to analyse membrane-associated biological processes. Substrate profiling studies employing synthetic peptide libraries have been employed to dissect the substrate specificity of FTase [18–22]. However, these only cover a fraction of possible substrate space and generally have to be validated *in vivo* for their physiological significance. Conversely, various tagging strategies using isoprenoid analogues have been developed to investigate the prenylation status of effector proteins *in vivo* [23–26]. These are technically challenging and do not necessarily recapitulate the chemical and functional identity of different isoprenoid PTMs. In addition, *in vivo* tagging strategies depend on mass spectrometric analysis which is semi-quantitative and inevitably has limited sensitivity.

To address these challenges, we devised an experimental platform to study protein prenylation using a growth-based *Saccharomyces cerevisiae* genetic selection system. Specifically, we adopted the Ras Recruitment system (RRS) as a screening platform to study protein prenylation in high-throughput. The selection system relies on the genetic complementation of a temperature sensitive mutant of *cdc25–2* that cannot grow at 36°C [27]. Growth rescue is achieved by recruiting a constitutively active derivative of H-Ras, to the plasma membrane. Originally, this system was designed to study protein-protein interactions [28,29], but was also modified to monitor intracellular protease activity [30]. Here, we expand the utility of the RRS to study protein prenylation by creating a global map of CaaX-box dependent membrane recruitment space in *S*. *cerevisiae*. In addition, we validate the RRS as a screening tool to engineer components of the protein prenylation machinery. Specifically, we create FTase mutants that recognise CaaX-box motives with altered substrate specificities in X that are not recognised by the endogenous protein prenylation machinery. These mutant FTases can now serve as starting points to conduct systematic sequence-structure-function relationship studies, engineer FTases with new CaaX-box substrate specificities or serve as model enzyme systems for *in vitro* evolutionary studies using the RRS.

## Materials and Methods

### Materials

The RRS including the temperature sensitive RRS screening strain (*MATα ura3 lys2 leu2 trp1 hisΔ200 ade2–101 cdc25–2*) and plasmid 05484 were a kind gift by Ami Aronheim (Technion, Israel Institute of Technology).

### Cloning Procedures

Plasmids used in this study are summarised in Table 1. Synthetic oligonucleotides (Integrated DNA Technologies) used to clone and assemble different DNA constructs are summarised in S1 File. All plasmids were subcloned in *Escherichia coli* and verified by sequencing (AGRF Brisbane) prior to transformation into *Saccharomyces cerevisiae*. Pfu C_x_ DNA polymerase (Agilent) was used to amplify DNA fragments by PCR according to manufacturer’s instructions. Re-annealing temperatures were chosen as T_M_—2°C where the melting temperature T_M_ was calculated with the Sigma OligoEvaluator. A combination of USER Enzyme (New England Biolabs) mediated cloning in combination with single strand extension was employed to generate different CaaX-box motives including a fully randomised CaaX-box with the three most C-terminal amino acids of the Ras61p reporter protein fully randomised [31,32] (S1 File). The assembled Ras61 reporter constructs were then inserted *via* HindIII and BamHI restriction sites into plasmid 05484. In case of the CaaX-box library, the transformation efficiency was quantified as >10^5^ to ensure the theoretical library diversity was saturated approximately 10-fold. A single-chain αβ-FTase fusion protein was assembled by overlap extension PCR using primers VS184 and VS185 to amplify α-FTase and primers VS187 and VS192 to amplify β-FTase. The 5’-α-FTase primer VS192 additionally included an optimal translation initiation site 5’-AACACAATGTCT-3’. The assembled DNA product was inserted *via* KpnI and EcoRI restriction sites into pYES2 to yield plasmid 05685. The coding nucleotide sequence of the single-chain αβ-FTase fusion construct is given in S1 File. Point mutants of the single-chain αβ-FTase fusion protein with negatively charged amino acids at the bottom of the active site at β-G142D and β-G142E were created by means of USER Enzyme DNA assembly and inserted into 05685 via BamHI and PmlI as summarised in S1 File.

**Table 1:** Summary of constructs employed in this study.

Plasmid Name	Characteristics	Backbone	Source
00253	*P*_*GAL1*_, *URA3*, 2μ Origin, High-Copy	pYES2	Commercial
05484	*P*_*MET25*_, *MYC-RAS61-ACAAX*, *LEU2*, 2μ Origin, High-Copy	pRS425	Ami Aronheim
05547	*P*_*MET25*_, *MYC-RAS61-KCVLS*, *LEU2*, 2μ Origin, High-Copy	pRS425	This Study
05548	*P*_*MET25*_, *MYC-RAS61- KCSIM*, *LEU2*, 2μ Origin, High-Copy	pRS425	This Study
05549	*P*_*MET25*_, *MYC-RAS61-KCVLL*, *LEU2*, 2μ Origin, High-Copy	pRS425	This Study
05667	*P*_*MET25*_, *MYC-RAS61-KCAIL*, *LEU2*, 2μ Origin, High-Copy	pRS425	This Study
05668	*P*_*MET25*_, *MYC-RAS61-CCIIS*, *LEU2*, 2μ Origin, High-Copy	pRS425	This Study
05669	*P*_*MET25*_, *MYC-RAS61-ACVIA*, *LEU2*, 2μ Origin, High-Copy	pRS425	This Study
05670	*P*_*MET25*_, *MYC-RAS61-ASRSAS*, *LEU2*, 2μ Origin, High-Copy	pRS425	This Study
05671	*P*_*MET25*_, *MYC-RAS61-TCTIL*, *LEU2*, 2μ Origin, High-Copy	pRS425	This Study
05727	*P*_*MET25*_, *MYC-RAS61-KCXXX*, *LEU2*, 2μ Origin, High-Copy	pRS425	This Study
05685	*P*_*GAL1*_, *αβ-FTASE (β*-W102T), *URA3*, 2μ Origin, High-Copy	pYES2	This Study
05691	*P*_*MET25*_, *MYC-RAS61-ACIIR*, *LEU2*, 2μ Origin, High-Copy	pRS425	This Study
05692	*P*_*MET25*_, *MYC-RAS61-ACIIK*, *LEU2*, 2μ Origin, High-Copy	pRS425	This Study
05693	*P*_*MET25*_, *MYC-RAS61-ACIIE*, *LEU2*, 2μ Origin, High-Copy	pRS425	This Study
05694	*P*_*MET25*_, *MYC-RAS61-ACIID*, *LEU2*, 2μ Origin, High-Copy	pRS425	This Study
05707	*P*_*GAL1*_, *αβ-FTASE (x*-W102T, *β*-G142D), *URA3*, 2μ Origin, High-Copy	pYES2	This Study
05708	*P*_*GAL1*_, *αβ-FTASE (β*-W102T, *β-*G142E), *URA3*, 2μ Origin, High-Copy	pYES2	This Study

### Screening with the Ras Recruitment System

The RRS was applied as previously described with minor modifications [28,29]. *Saccharomyces cerevisiae* (MAT*α ura3 lys2 leu2 trp1 hisΔ200 ade2–101 cdc25–2*) served as the screening strain for the RRS. Yeast was generally transformed using a standard lithium acetate procedure and grown on Hartwell’s Complete (HC) media for 3–4 days under permissive conditions at 25°C. Yeast transformed with Ras61p coding constructs were grown in HC-Leu or in HC-Leu-Ura if single-chain αβ-FTase coding constructs were co-transformed. Furthermore, the expression of Ras61p was under the control of the methionine-repressible MET25 promoter while the expression of single-chain αβ-FTases was under the control of the galactose-inducible GAL1 promoter. To suppress gene expression from pMET25 promoter, methionine was included at 50 μg/mL. To induce expression of the GAL1 promoter, glucose was replaced with galactose medium consisting of 3% galactose, 2% raffinose, 2% glycerol. For dilution spot assays, individual colonies were picked and grown to saturation in liquid HC-Leu or HC-Leu-Ura if single-chain αβ-FTase coding constructs were co-transformed. Serial 5-fold dilutions of the liquid cultures were then spotted on HC-Leu or HC-Leu-Ura agar and grown for 3–6 days under restrictive and permissive conditions at 37°C and 25°C respectively. In library selections, plasmid DNA was isolated using the Zymoprep Yeast Plasmid Miniprep II according to manufacturer’s instructions (Zymoresearch).

### Ion Torrent Sequencing

A schematic summary on preparing libraries for next-generation sequencing with the Ion-Torrent system is given in S1 File. Briefly, the CaaX-box coding region from the plasmid DNA was PCR amplified with primers VS340 and VS296 and the PCR product was treated with USER Enzyme (1 U per 1 μg DNA) to create single stranded 3’ extensions. The resulting fragment was then ligated to DNA cassettes that provide the sites for immobilizing and amplifying DNA for sequencing with the Ion Torrent System (200 U T4 DNA Ligase per 1 μg DNA). Libraries were prepared for sequencing on the Ion Torrent platform as per manufacturer’s instructions (Life Technologies) and a single Ion Torrent 314 Chip was used for each sample. The number of sequence reads for each different library set are summarised in Table 2. Enrichment factors were determined for each of the 8000 different CaaX-box motives that occurred at least twice by calculating the frequency of each peptide motif under restrictive conditions at 37°C and normalising it over its frequency under permissive conditions at 25°C. If a sequence did not occur in the 25°C set, it was assigned a value of 1 to enable normalisation. Enrichment factors of sequence motives that occurred less than 5 times under both restrictive and permissive conditions should be treated with caution as small stochastic variations yield comparatively large changes in enrichment factors and should thus be treated with low confidence. A complete list of CaaX-box motives, their enrichment factors and counts in the naïve, 25°C and 37°C library sets is given in S2 File. A complete list of potentially untransformed CaaX-box motives that have neither been observed in the 25°C library set nor in the 37°C library set is given in S3 File.

**Table 2:** Summary of NGS counts and library coverage.

Library Name	NGS Counts	Primary Coverage	Secondary Coverage
Naïve: Following Transformation in *E*.*coli*	168,627	99.45% ^1^	99.69% ^2^
25°C: After Permissive Growth in Yeast	63,393	86.35% ^1^	89.13% ^3^
37°C: After Restrictive Growth in Yeast	158,782	48.31% ^1^	N/A

^1^Based on the observation that a sequence motif is detected at least twice in a particular NGS run.

^2^Based on the observation that a sequence motif is detected at least twice in the Naïve, 25°C or 37°C data set.

^3^Based on the observation that a sequence motif is detected at least twice in either the 25°C or 37°C data set.

### Construction and characterisation of GFP-αβ-FTase Fusion Proteins

Single-chain αβ-FTase fusion proteins were constructed from *Rattus Norvegicus* FTase subunits by fusing the C-terminus of α-subunit at position 377 to the N-terminus of the β-subunit at position 1 via a Tobacco Etch Virus (TEV) protease cleavable linker with the sequence ENLYFQG. The resulting open reading frame was cloned into pLTE vector, expressed in *Leishmania tarentolae* cell-free system (200 μL) as a fusion protein with GFP and purified on green fluorescent protein (GFP) capturing beads (30 μL, 50% GFP-Cap bead slurry) as previously described [33,34]. Following expression, the activity of the purified GFP-αβ-FTase fusion protein was assayed *in vitro* on microbeads (15 min) utilizing the fluorescent farnesylpyrophosphate (FPP) analogue NBD-GPP (5 μM, Jena Bioscience) and m-Cherry-K-Ras (5 μM) as substrates as described before [35]. Following elution in the presence of SDS loading buffer, the reactions were resolved on SDS-PAGE and the fluorescent bands corresponding to the prenylated protein substrate mCherry-K-Ras were visualised by fluorescent image scanning (Typhoon Trio, Amersham Biosciences). As negative controls, the reactions were performed in the presence of a 5-fold molar excess of FPP (25 μM) over the fluorescent isoprenoid NBP-GPP [35]. To examine the structural integrity of the fusion protein, the expressed fusion protein GFP-αβ-FTase was treated with TEV protease and the cleavage products were resolved by SDS-PAGE and detected by Western Blotting using monoclonal anti-GFP antibody (Sigma). The signal was visualised using Odyssey Infrared Imaging System protocol with primary antibodies at a 1:2000 dilution and secondary antibody: IRDye 680 goat anti-mouse (Li-Cor Biosciences) at 1:10000 dilution.

## Results

### Validating the Ras Recruitment System to Assay Protein Prenylation

**Fig 1:**
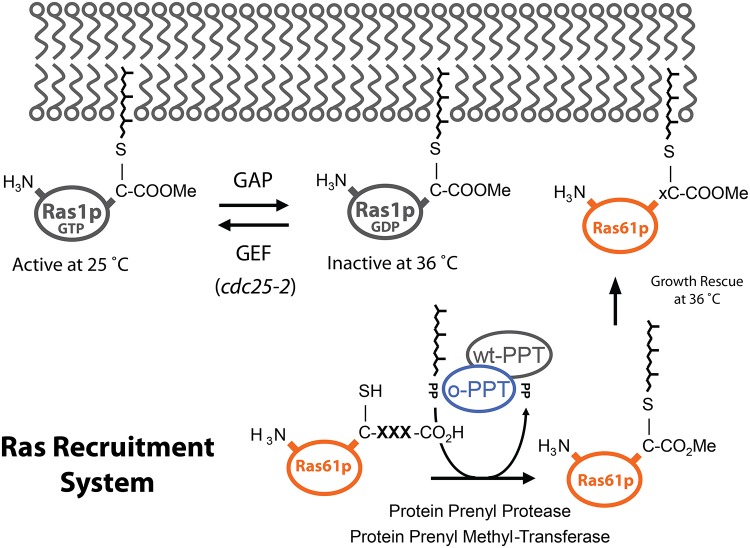
The principle of the Ras Recruitment System (RRS). The system is based on a temperature sensitive GDP exchange factor (encoded by the *cdc25–2* allele) that is rendered inactive at 36°C trapping endogenous Ras1p in its inactive GDP bound form. Growth is rescued by genetic complementation with a constitutively active mutant of mammalian H-Ras (*RAS61*). To exert its function and rescue growth, Ras61p needs to be directed to the plasma membrane. This can either occur through protein-protein interactions or lipid modifications such as myristoylation or prenylation. Specifically, prenylation can either be mediated by endogenous protein prenyltransferases (wt-PPTases) that recognise naturally occurring, prenylatable CaaX-box motives or engineered protein prenyltransferases (o-PPTases) that recognise orthogonal CaaX-box motives that are not recognised by the endogenous machinery. For optimal membrane recruitment and genetic complementation in the RRS, the three most C-terminal amino acids of prenylated CaaX-box motives are removed by highly specific protein prenyl proteases located in the endoplasmic reticulum followed by carboxymethylesterification of the C-terminus.

Over the past 20 years, numerous genetic studies in *Saccharomyces cerevisiae* have generated a wealth of insight into the function of the eukaryotic protein prenylation machinery [3,4,9,36,37]. The most commonly used assay is the **a**-factor screen that has been used to probe the substrate specificity underlying CaaX-box farnesylation and proteolysis [38,39]. The assay relies on a visual analysis of a halo around the yeast colony which reflects the efficiency of post-translational maturation of **a**-factor. While very powerful, this screen has limited throughput and cannot be used for the selection of large libraries. Instead, we chose to adopt the yeast based Ras Recruitment System (RRS) to assay protein prenylation functions in high-throughput (Fig. 1) [28,29]. The RRS was originally developed as an alternative to the yeast-2-hybrid system with the aim of identifying protein-protein interactions that could include membrane proteins. It is based on a temperature sensitive *cdc25–2* mutant which encodes for a GDP exchange factor (GEFs) that is rendered inactive at 37°C and thus traps endogenous Ras1p in its inactive GDP bound form [27]. Growth is rescued by genetic complementation with a constitutively active mutant of the mammalian H-Ras isoform, termed Ras61p, that needs to be directed to the plasma membrane. This can either occur *via* protein-protein interactions [28,29] or lipid modifications such as prenylation or myristoylation.

**Fig 2:**
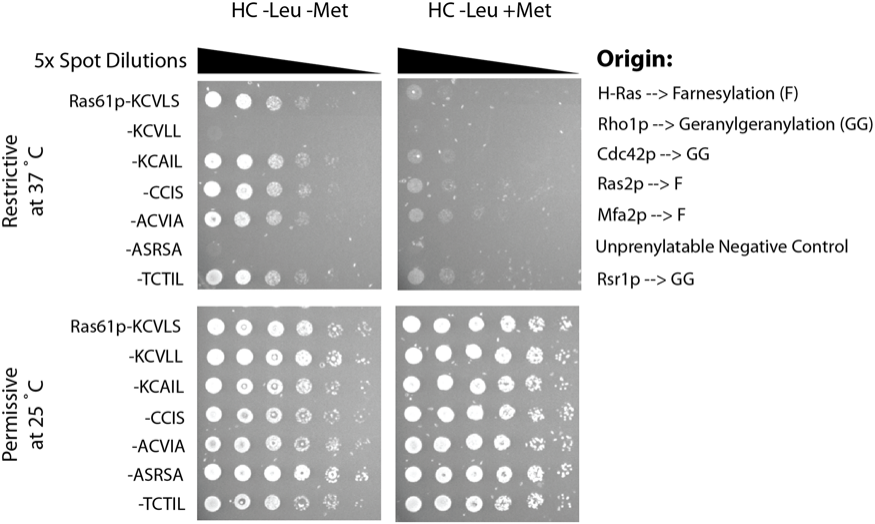
Validation of the RRS as a screening assay for protein prenylation. Ras61p with several CaaX-box motives known to be farnesylated, geranylgeranylated or both were analysed for their ability to complement growth in the RRS. Proteins known to be farnesylated generally rescued growth while the unprenylatable motif-SRSA did not. This includes the mono-geranylgeranylated motives-KCAIL of CDC42p and -TCTIL of Rsr1p which are known to be cross-farnesylated, but not-KCVLL of Rho1p which is exclusively geranylgeranylated. This suggests that only farnesylation is detected in the RRS (+Met denotes 5 μg/mL methionine in the medium to suppress gene expression while in its absence gene expression is induced).

To validate the RRS as a genetic screening tool for assaying protein prenylation function in yeast, we introduced different peptide motives that are known to be either farnesylated or geranylgeranylated at the C-terminus of the Ras61p reporter protein, and examined whether they could successfully direct Ras61p to the plasma membrane and rescue growth (Fig. 2A). Peptide motives known to be farnesylated could effectively rescue growth while peptide motives known to be mono-geranylgeranylated yielded mixed results (Fig. 2B). For instance, -CTIL and -CAIL (which are derived from Rsr1p and Cdc42p) were functional while-CVLL (which is derived from Rho1p) was not. It is conceivable that only farnesylation efficiently directs the reporter protein Ras61p to the plasma membrane while sole mono-geranylgeranylation does not. This phenomenon has previously been described for both mammalian and yeast FTase [19,40]. Notably, biochemical studies have previously confirmed that yeast FTase can farnesylate peptide motives with Leu in the X position including—CAIL and -CTIL of Cdc42p and Rsr1p, albeit with lower efficiency [40]. Based on these observations, we conclude that a positive read-out in the RRS depends on farnesylation. Furthermore, the CaaX-box dependent growth complementation pattern in the RRS suggests that mono-geranylgeranylation by GGTase I is not detected.

### Mapping the CaaX-Box Dependent Membrane Recruitment Space in *S*. *cerevisiae*

To investigate the substrate specificity of the endogenous protein prenylation machinery in *S*. *cerevisiae*, we chose to comprehensively map the CaaX-box dependent membrane recruitment space as defined by the ability of the sequences to rescue cell growth in the RRS. To this end, a synthetic CaaX-box library was created with the three C-terminal amino acids fully randomised. Quality control by Ion Torrent sequencing of the naive library showed that 7956 out of 8000 theoretically possible CaaX-box motives could be detected at least twice within 168,627 sequence reads accounting to a primary library coverage of 99.45% (Table 2). Furthermore, a relatively large spread was observed in the occurrence of individual sequence motives as the most frequent peptide occurred 153 times. This bias could be largely attributed to stochastic effects associated with degenerate NNS codons that encode amino acids with varying frequencies (S4 File).

**Fig 3:**
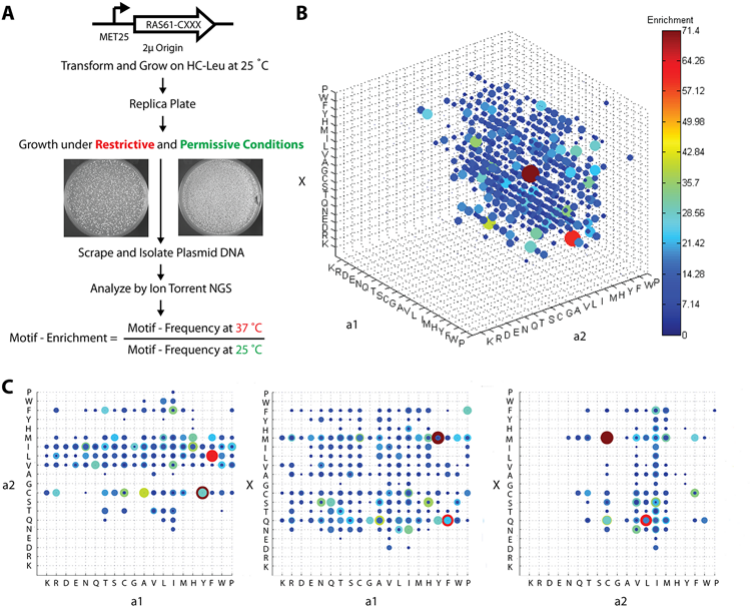
Mapping CaaX-box dependent membrane recruitment space in yeast. (A) Flow chart of the mapping experiment. The CaaX-box library was transformed into the RRS screening strain, grown for 4 days under permissive conditions at 25°C, replica plated and then grown for another 4–6 days under restrictive and permissive conditions at 37°C and 25°C. CaaX-box coding plasmid DNA was then isolated and analysed for the two different library sets by NGS with the Ion Torrent system. The enrichment was determined for each of the 8000 different CaaX-box motives by measuring the frequency of each peptide under restrictive conditions and normalising it over its frequency under permissive conditions. (B) Graphic representation of the enrichment factors of 8000 different CaaX-box motives is summarised in a 4D plot: Each axis represents the 20 different amino acids while the size of each dot is proportional to the enrichment of a specific CaaX-box motif. Only CaaX-box motives that have been enriched >3 are shown. (C) Cross-sectional views along the a_2_-a_1_, X-a_1_ and X-a_2_ axis illustrate that a_2_ exerts the greatest specificity on substrate specificity with small hydrophobic residues highly preferred followed by the anchoring position X and a_1_.

To map CaaX-box dependent membrane recruitment space in *S*. *cerevisiae*, the library was transformed into the RRS screening strain and grown for 4 days under permissive conditions at 25°C. The library was replica plated and then grown for another 4–6 days under restrictive and permissive conditions at 37°C and 25°C (Fig. 3A). Yeast colonies were then scraped off, the plasmid DNA isolated and the libraries was analysed as described above. The enrichment was determined for each of the 8000 different CaaX-box motives by measuring the frequency of each peptide under restrictive conditions and normalising it over its frequency under permissive conditions. Global analysis showed that more than 6.2% of prenylatable sequence space led to membrane recruitment in the RRS after applying an enrichment cut-off > 3 with the majority of hits considered canonical. Furthermore, the a_2_ position displayed the most stringent substrate specificity with small hydrophobic residues highly preferred while X and a_1_ appear to be comparatively promiscuous (Fig. 3B). This is in accord with results from recent *in vitro* substrate profiling studies with yeast and mammalian FTases that, deviating from the previously held consensus, attribute a relatively loose contribution towards the specificity of the anchoring residue X [18,19,21,22].

Furthermore, comparing the data set against all 73 endogenous yeast ORFs that contain a hypothetical CaaX-box motif based on the S288C reference genome shows a clear segregation into membrane associated protein functions for enrichment factors >3 (S5 File). The only notable exception was-CVLL of Rho1p with an enrichment factor < 0.12 which confirmed our original substrate mapping experiment (Fig. 2B). Furthermore, Leu in X, which constitutes a key recognition feature for GGTase I, was generally underrepresented in the screen as all sequence motives ending with-LL were enriched less than 1 (S6 File). A similar pattern was observed for CaaX-box motives ending with—VL of which only 3 out of 20 were modestly enriched between 3- and 5-fold; only sequence motives ending with—IL were significantly selected in the RRS with 13 out of 20 motives enriched between 4- and 17-fold (S6 File). This includes—CTIL of Rsr1p,—CAIL of Cdc42p and—CIIL of Rho2p which have previously been shown to be substrates for the FTase of *S*. *cerevisiae in vitro* [40,41]. Overall, this provides further evidence that mono-geranylgeranylation does not lead to a positive read-out in our RRS screen.

In addition, we observed a comparatively large number of -CXCC and -CCXC motives that match the consensus for GGTase II (S7 File). While these overlap with the substrate specificity of FTase, we cannot determine whether di-geranylgeranylation mediated by GGTase II can rescue growth in the RRS. This is however highly unlikely as GGTase II prenylation requires interaction of Rab Escort Protein (Mrs6p) with both GGTase II and the Rab GTPase domain of the substrate. Lack of such sequences in *S*. *cerevisiae* (S5 File), may suggest an evolutionary selection against ambiguous CaaX-box motives that could potentially cause mislocalisation of prenylated effector proteins.

To further validate the results from our high-throughput screen and examine to what extent proteolytic processing is necessary for a positive read-out in the RRS, we compared our membrane recruitment data with that obtained in previous **a**-factor screens using 60 different CaaX-box motives [39]. Generally, a strong correlation was observed with the only notable deviation occurring for large hydrophobic residues in the a_1_ position which rescued growth in the RRS, but lead to negative read-outs in the **a**-factor screen (S8 File). Overall, this suggests that all three PTM steps including proteolytic processing are required for optimal complementation and growth rescue in the RRS. An exception may apply to large hydrophobic residues in the a_1_ position that may adversely impact the efficiency of proteolytic processing which is essential for a positive read-out in the **a**-factor assay, but not necessarily in the RRS. In this respect, it has previously been shown that constitutively active yeast Ras2p function can be attenuated, but not necessarily abolished, when-aaX cannot be proteolytically removed in yeast [8]. A similar scenario may apply to the RRS where Tyr, Trp and Phe in a_1_ may assist with membrane association of the Ras61p reporter protein. Functionally, this has no implications in yeast as no such sequences occur in the set of 73 potential CaaX-box motives (S5 File). Yet, it is intriguing to speculate to what extent the same holds true for mammalian protein prenylation processes that contain a greater diversity of potential CaaX-box motives.

Other notable, non-canonical CaaX-box motives feature a number of sequences with negatively charged amino acids in the anchoring position X (S9 File). These strongly converge on an optimal consensus motif that strictly requires Ile in a_2_ and highly prefers β-branched amino acids in a_1_ (S9 File). Given no such sequences naturally occur in yeast, this has no direct functional implications *in vivo*. Yet, similar substrate motives have been described for mammalian proteins and have also been shown to be substrates for mammalian FTases *in vitro* [18,21]. This implies the scope of non-canonical CaaX-box motives that can be farnesylated and confer functionality is potentially greater than previously thought while it remains to be determined to what extent these non-canonical substrate motives confer functionality *in vivo* and can efficiently compete for prenylation with endogenous CaaX-box motives.

Beyond CaaX-box dependent protein prenylation determinants, the identity of amino acids N-terminal to the prenylated Cys are also likely to affect intracellular protein trafficking, membrane localisation and thus growth rescue in the RRS. Specifically, the reporter protein Ras61p features two Cys residues that can be potentially palmitoylated. In this regard, studies with Ras2p in *S*. *cerevisiae* and Rho2p in *S*. *pombe* show that palmitoylation is essential for correct subcellular localisation to the plasma membrane, signalling and function [42,43]. In the future, it is conceivable to analyse in high-throughput how growth rescue in the RRS depends on these regulatory PTMs as well as other plasma membrane localizing features such as polybasic motives, and examine any context dependencies with CaaX-box-dependent protein prenylation specificity features.

### Uncoupling FTases from the Endogenous PPTase Machinery

The developed selection platform provides not only the opportunity to map FTase substrate space, but also enables the systematic engineering of protein prenyltransferases. We first sought to create mutant FTases that can be operated independently of the endogenous protein prenylation machinery in yeast. In this way, they could be mutated without any detrimental effects on the house-keeping function of endogenous FTases. Specifically, this requires (i) an orthogonal CaaX-box substrate that does not cross-react with endogenous PPTases and (ii) a mutant FTase which can prenylate the orthogonal substrate, and thus rescue growth under restrictive conditions in the RRS. Previous studies have shown that CaaX-box motives with charged residues in a_2_ can be prenylated *in vitro* by mammalian FTase and GGTase I mutants that feature complementary charges in their a_2_ binding pocket [44,45]. In addition, these mutant FTases were shown to prenylate charged CaaX-box motives on fluorescent reporter proteins within mammalian cells [44].

Here, we focus on residue X to engineer the substrate specificity of FTases, which, similar to a_2_, provides key specificity features towards substrate recognition (Fig. 3C). Notably, charged residues in X are not efficiently recognised by the endogenous protein prenylation machinery, but can be prenylated by mutant yeast FTases^β-G159D, E, K and R^ with complementary charged amino acids in the anchoring position as previously shown using a combination of **a**-factor screen and growth based selection assays based on constitutively active Ras2p mutants in a Δ*ram1* genetic background that is deficient in FTase function [38].

**Fig 4:**
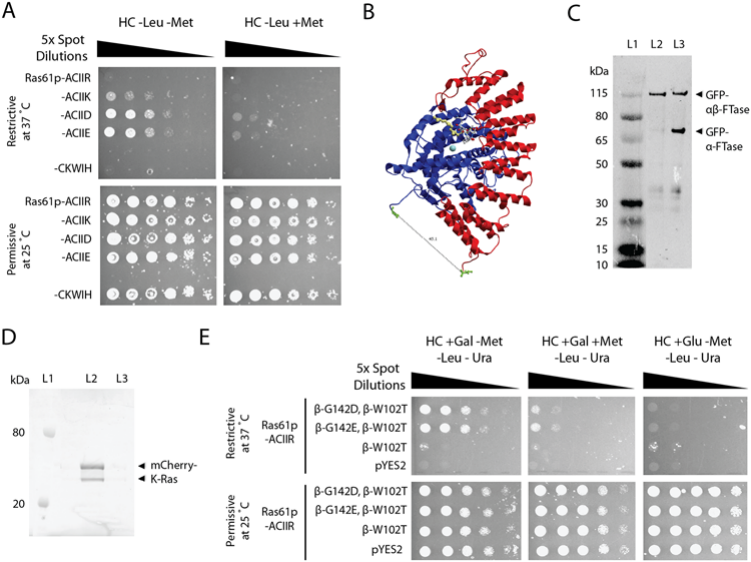
Engineering FTases with altered substrate specificities. (A) CaaX-box motives with positively charged residues in the anchoring position X cannot rescue growth in the RRS and thus provide poor substrates for endogenous FTases in *Saccharomyces cerevisiae*. (B) Structural model of the αβ-FTase heterodimer derived from *Rattus norvegicus* (PDB: 1KZO). The C-terminus of the α-subunit (highlighted in blue) is separated by 40 Å from the N-terminus of the β-subunit (highlighted in red). (C) Western blot analysis of GFP-αβ-FTase fusion proteins derived from *R*. *norvegicus* expressed in *Leishmania tarantolae* cell-free expression system. The linker connecting α- and β-subunits contained a TEV protease cleavage site that is cleaved with exogenously added TEV protease. L1: Protein Ladder; L2: Uncleaved GFP-αβ-FTase; L3: GFP-αβ-FTase cleaved with TEV Protease. (D) Fluorescent scan of SDS–PAGE loaded with mCherry-K-Ras *in vitro* prenylation reaction containing single-chain GFP-αβ-FTase fusion proteins and fluorescent phosphoisoprenoid NBD-GPP [35]. Addition of FPP to the reaction prevents formation of the fluorescent reaction product due to competition with the fluorescent lipid donor. L1: Protein Ladder; L2: GFP-αβ-FTase bound to GFP-Cap beads, 5 μM mCherry-K-Ras, 5 μM NBP-GPP; L3: GFP-αβ-FTase bound to GFP-Cap beads, 5 μM mCherry-K-Ras, 5 μM NBD-GPP, 25 μM FPP. (E) To facilitate expression and prevent cross-heterodimerisation between yeast and exogenous FTase subunits, a single-chain αβ-FTase was created based on mutant β-W102T while introducing negative charges at the bottom of the active site at β-G142D and β-G142E enabling FTase to farnesylate a CaaX-box motif with a positive charge in X and thus rescue growth in the RRS. Controls: pYES2 denotes vector control and β-W102T the unmodified, single-chain αβ-FTase^β-W102T^ mutant neither of which can prenylate the orthogonal CaaX-box motif.

We thus chose to probe how these CaaX-box and FTase mutants would affect growth-based selection in the RRS. In the first instance, we focused on developing an orthogonal protein prenylation substrate with charged amino acids in the anchoring position X in the context of a-CIIX motif (Fig. 4A). Dilution spot assays showed that a positively charged Lys in the anchoring position X provides a very poor prenylation substrate while Arg is not recognised at all. Conversely, negatively charged amino acids in the anchoring position X still led to a positive read-out in the RRS. This also validated the finding in our CaaX-box mapping experiments where a limited set of motives with negatively charged residues in the anchoring position X including -CIID and -CIIE could be enriched in the RRS screen (S8 File).

Next, we engineered FTases that can recognise CaaX-box motives with positively charged residues in the anchoring position X. To facilitate stoichiometric expression of heterodimeric FTase in yeast without the danger of cross-heterodimerisation with the endogenous α-subunit, we sought to create a single-chain αβ-FTase fusion protein. Here, FTase crystal structures guided the design of the linkers connecting the C-terminus of *Rattus norvegicus* FTase α-subunit and the N-terminus of β-subunit resulting in a continuous polypeptide that was additionally fused to GFP to assist purification (Fig. 4B). To ascertain that the resulting fusion protein GFP-αβ-FTase was folded and functional, we expressed the fusion protein using our recently developed *Leishmania*-based cell-free expression system [33] which yielded a homogenous polypeptide of the expected size that was able to prenylate protein substrates *in vitro* (Fig. 4C and D). We then introduced an additional mutation β-W102T to expand the bottom of the enzyme’s active site [23,46]. Note, for the purpose of creating FTases with altered substrate specificities, it is not necessary to adhere to the original wild-type context, but it is sufficient to probe its reactivity for the orthogonal CaaX-box substrate.

To enable prenylation of positively charged CaaX-box motives in yeast, we introduced complementary negative charges at the bottom of the active site at β-G142D and β-G142E in our single-chain αβ-FTase^β-W102T^ mutant [38]. Co-transforming the-CIIR construct subsequently rescued growth in the RRS and was strictly dependent on the expression of both the reporter protein Ras61p and the single-chain αβ-FTase^β-W102T, β-G142D^ and αβ-FTase^β-W102T, β-G142E^ mutants (Fig. 4E). In summary, we have created a mutant FTase that can selectively prenylate Ras61p with an orthogonal CaaX-box motif effectively uncoupling it from the endogenous protein prenylation machinery. This substrate-enzyme pair can now serve as a starting point for further, systematic protein engineering using the RRS.

## Discussion

In this study, we successfully adopted the RRS for the high-throughput analysis of protein prenylation in *S*.*cerevisiae*. This enabled us to create the first comprehensive map of CaaX-box dependent membrane recruitment space and to engineer components of the eukaryotic protein prenylation machinery. Overall, the developed experimental framework should open up new avenues of studying protein prenylation in high-throughput with significant advantages over current experimental approaches: Notably, a growth-based selection assay in combination with next-generation sequencing enables an unprecedented holistic view on the combinatorial diversity associated with protein prenylation. Furthermore, growth rescue in the RRS depends on localisation of the reporter protein to the plasma membrane which is physiologically more relevant compared to profiling enzyme activities *in vitro* [7,18–21]. In addition, yeast-based genetic selection experiments are much cheaper and more versatile compared to screening chemically synthesised peptide libraries [7,18–21] and technically less challenging compared to proteomic tagging strategies [23–25]. For instance, it is possible to modulate expression levels with high- and low-copy plasmids as well as a range of well characterised promoter systems to fine-tune the expression levels of either the reporter gene or single-chain αβ-FTase mutants [47,48].

Beyond studying farnesylation, a wealth of knowledge has accumulated over the past 30 years that can principally be exploited to establish growth-based selection assays in order to assay alternative protein prenylation functions in yeast. For instance, genetic studies have shown that strains deficient in GGTase I function are not viable, which, at the molecular level, strictly depends on mono-geranylgeranylation of Cdc42p and Rho1p [36,37]. Thus, both Cdc42p and Rho1p could be employed as reporter proteins to assay for mono-geranylgeranylation in high-throughput. In the context of drug discovery, our experimental framework could also be applied to investigate the effect of PPTase inhibitors on the prenylation efficiency of different CaaX-box libraries. This would provide a cheaper and technically less challenging alternative to proteomic tagging strategies with isoprenoid analogues [23–26]. Here, it is conceivable to “humanise” defined parts of the protein prenylation machinery in yeast as many components of the eukaryotic protein prenylation are functionally interchangeable [49,50]. Expression as a single-chain PPTase overcomes any potential problems that could arise from cross-heterodimerisation with endogenous PPTase subunits. In this way, one could account for subtle structural differences that influence the prenylation efficiency of individual CaaX-box motives at different concentrations of PPTase inhibitors. Similar strategies have been undertaken to identify and correlate how different FTase inhibitors affect the prenylation efficiency of native targets in yeast and mammalian cell lines and examine its effect on gene expression [51].

Single-chain αβ-FTases would also enable large scale genetic and evolutionary studies with the aim of probing structural requirements of the αβ-subunit interface or determine how residues lining the active site impact the substrate specificity of FTase in the a_1_ and a_2_ position. Similar studies have been conducted *in vitro* for FTase and GGTase I, but ultimately depend on the availability of synthetic substrate libraries and cannot readily screen large libraries of PPTase mutants [44,45]. In addition, *in vitro* evolutionary studies could be conducted measuring the impact of defined mutational loads over successive generations on stability, subunit organisation and substrate specificity. Substrate specificities can subsequently diverge further until a fully orthogonal substrate-FTase pair has been created. Such systems could ultimately be employed as research tools to control the prenylation status of a defined set of prenylated proteins *in vivo* through fully orthogonal protein prenylation pathway(s).

## Supporting Information

S1 File Oligonucleotides and summary of cloning schemes. (PDF)

S2 File Summary of global CaaX-box mapping experiments (CXXX). (PDF)

S3 File Untransformed CaaX-box motives. (PDF)

S4 File Comparison with a simulated CaaX-box library distribution. (PDF)

S5 File Comparison with naturally occurring CaaX-box motives in *S*.*cerevsiae*. (PDF)

S6 File Summary of GGTase I specific motives (CXXL). (PDF)

S7 File Summary of GGTase II specific motives (CCXC and CXCC). (PDF)

S8 File Summary of CaaX-box motives with charged residues in X. (PDF)

S9 File Comparison with a-factor screens. (PDF)
