# Supplementary material for: Towards the Systematic Mapping and Engineering of the Protein Prenylation Machinery in Saccharomyces cerevisiae
Source: PLoS One. 2015 Mar 13;10(3):e0120716. doi: 10.1371/journal.pone.0120716 (PMC4358939; doi:10.1371/journal.pone.0120716)
Supplement: S1 File — (PDF) [file pone.0120716.s001.pdf]

Table S1: Summary of oligonucleotides used to create different constructs in this study.

Name	Primer Sequence	Notes
VS112	5’-GGAAGGAAAAGCTTCCCGGGAC-3’	Forward Ras61, HindIII
VS113	5’-CAGCTCAUGCAGCCGGG-3’	Reverse Ras61, For Uracil-Excision-Extension
VS114	5’-GGAAGAAGGATCCTTACTAAGACAGAACACATTTACAGCTCAT-3’	-KCVLS Extension, BamHI
VS115	5’-GGAAGAAGGATCCTTACTACATAATAGAACATTTACAGCTCAT-3’	-KCSIM Extension, BamHI
VS116	5’-GGAAGAAGGATCCTTACTACAACAAAACACATTTACAGCTCAT-3’	-KCVLL Extension, BamHI
VS117	5’-GGAAGAAGGATCCTTACTASNNSNNSNNACATTTACAGCTCAT-3’	-KCXXX Extension, 8000-mer Library, BamHI
VS177	5’-GGAAGAAGGATCCTTACTAAAGAATAGCACATTTACAGCTCAT-3’	-KCAIL Extension, BamHI
VS178	5’-GGAAGAAGGATCCTTACTAAGAAATAATACAACAACAGCTCAT-3’	-CCIIS Extension, BamHI
VS179	5’-GGAAGAAGGATCCTTACTAAGCAATAACACAAGCACAGCTCAT-3’	-ACVIA Extension, BamHI
VS180	5’-GGAAGAAGGATCCTTACTAAGCAGAAGCTCTAGAAGCACAGCTCAT-3’	-ASRASA Extension, BamHI
VS181	5’-GGAAGAAGGATCCTTACTATAGAATAGTACAAGTACAGCTCAT-3’	-TCTIL Extension, BamHI
VS184	5’-GAGAATCTGTACTTCCAGGGCGCTTCTTCGAGTTCCTTCACCTATTATTG-3’	5’-β-FTase
VS185	5’-AACAGTGAATTCTTACTAAAGCTTGTCAGTGGCAGGATC-3’	3’-β-FTase
VS187	5’-GCCCTGGAAGTACAGATTCTCTACACTCGCCGGTATGTCACTTTCTC-3’	3’-α-FTase
VS192	5’-AACAGTGGTACCAACACAATGTCTCCAGCACAGCAGCCGCAGGAA-3’	5’-α-FTase + Optimal Translation Initiation
VS212	5’-GGAAGAAGGATCCTTACTATCTAATAATACAAGCACAGCTCAT-3’	-ACIIR Extension, BamHI
VS213	5’-GGAAGAAGGATCCTTACTATTTAATAATACAAGCACAGCTCAT-3’	-ACIIK Extension, BamHI
VS214	5’-GGAAGAAGGATCCTTACTAATCAATAATACAAGCACAGCTCAT-3’	-ACIID Extension, BamHI
VS215	5’-GGAAGAAGGATCCTTACTATTCAATAATACAAGCACAGCTCAT-3’	-ACIIE Extension, BamHI
VS296	5’-TTACUAGTGGATCAACCCAAAATCACTA-3’	Reverse Primer, Ion Torrent, Amplicon
VS340	5’-GAGUGGCCCCGGCTGCATG-3’	Forward Primer, Ion Torrent, Amplicon
VS341	5’-CCATCTCATCCCTGCGTGTCTCCGACTCAGGAGT-3’	5’-Flanking Region, Ion Torrent DNA Cassette
VS342	5’-PO_3-CTGAGTCGGAGACACGCAGGGATGAGATGG-3’	5’-Flanking Region, Ion Torrent DNA Cassette
VS343	5’-PO_3-ATCACCGACTGCCCATAGAGAGG-3’	3’-Flanking Region, Ion Torrent DNA Cassette
VS344	5’-CCTCTCTATGGGCAGTCGGTGATTTACT-3’	3’-Flanking Region, Ion Torrent DNA Cassette
VS200	5’-ACGCTCTGCTACTGGATCCTGC-3’	5’-Fragment #1 $\alpha\beta$-FTase, BamHI
VS201	5’-TCCAAAUCCACCGTCTGGACTCTGACAC-3’	3’-Fragment #1 $\alpha\beta$-FTase, USER
VS204	5’-P-GATGGCCCTGGTCAGTACCCACACCTCGCTCCCACGTATGCAGCT-3’	Synthetic DNA, Fragment #2, Forward β-G142D
VS205	5’-P-GAAGGCCCTGGTCAGTACCCACACCTCGCTCCCACGTATGCAGCT-3’	Synthetic DNA, Fragment #2, Forward β-G142E
VS208	5’-P-ATACGTGGGAGCGAGGTGTGGGTACTGACCAGGGCCATCTCCAAAT-3’	Synthetic DNA, Fragment #2, Reverse β-G142D
VS209	5’-P-ATACGTGGGAGCGAGGTGTGGGTACTGACCAGGGCCTTCTCCAAAT-3’	Synthetic DNA, Fragment #2, Reverse β-G142E
VS210	5’-GCAGCUGTCAACGCGCTATG-3’	5’-Fragment #3 $\alpha\beta$-FTase, USER
VS211	5’-GGTAGAAGTCACGTGACTTGCCAG-3’	3’-Fragment #4 $\alpha\beta$-FTase, PmlI

1. Assembly of CAAX-Box Reporter Proteins Exemplified for the 8000-mer CAAX-Box Library

(1) PCR Amplify Ras61

```
5′-GGAAGGAAAAGCTTCCCGGGAC-3′ (VS112)

5′-GGAAGGAAAAGCTTCCCGGGAC......ATGAGAGTGGCCCCGGCTGCATGAGCTG-3′
3′-CCTTCCTTTTCGAAGGGCCCTG......TACTCTCACCGGGGCCGACGUACTCGAC-5′

                                          3′-GGGCCGACGUACTCGAC-5′  (VS113)
```

(2) Create 3′ Single Stranded Extensions by Treating PCR Product with USER Enzyme

```
5′-GGAAGGAAAAGCTTCCCGGGAC......ATGAGAGTGGCCCCGGCTGCATGAGCTG-3′
3′-CCTTCCTTTTCGAAGGGCCCTG......TACTCTCACCGGGGCCGACG-P-5′
```

(3) Attach CAAX-Box Library Coding Oligonucleotide to the Recessed 5′ End with T4 DNA Ligase

```
5′-GGAAGGAAAAGCTTCCCGGGAC......ATGAGAGTGGCCCCGGCTGCATGAGCTG-3′
3′-CCTTCCTTTTCGAAGGGCCCTG......TACTCTCACCGGGGCCGACGTACTCGACATTTACANNSNNSNNSATCATTCCTAGGAAGAAGG-5′

                     3′-TACTCGACATTTACANNSNNSNNSATCATTCCTAGGAAGAAGG-5′   (VS117)
```

(3) Fill in 3′ End with Pfu C_x Polymerase

```
          HindIII                                               BamHI

                              E  S  G  P  G  C  M  S  C  K  C  X  X  X  *  *
5′-GGAAGGAAAAGCTTCCCGGGAC......ATGAGAGTGGCCCCGGCTGCATGAGCTGTAAATGTNNSNNSNNSTAGTAAGGATCCTTCTTCC-3′
3′-CCTTCCTTTTCGAAGGGCCCTG......TACTCTCACCGGGGCCGACGTACTCGACATTTACANNSNNSNNSATCATTCCTAGGAAGAAGG-5′
```

2. Creation of Single-Chain αβ-FTase Constructs

Nucleotide Sequence of the Single-Chain αβ-FTase (*β*-W102T) => Construct 05685:

ATGTCTCCAGCACAGCAGCCGCAGGAAGAAGAGATGGCGGCCGAGGCCGGGGAAGCAGCGGCGTCCCCTATGGACGACGGGTTTCTGAGCCTGGACTCGCCCACCTATGTCTTGTACAGGGACAGGGCAGAGTGGGCTGACATAGACCCAGTGCCCCAGAATGATGGCCC
CAGTCCAGTGGTCCAGATCATCTACAGTGAAAAGTTTAGAGACGTCTATGATTACTTCCGAGCTGTTCTGCAGCGCGATGAAAGAAGCGAACGAGCCTTTAAGCTCACTCGAGATGCTATTGAGTTAAACGCAGCCAACTATACGGTGTGGCATTTTCGGAGAGTTCTCT
TGAGGTCGCTTCAGAAGGATCTGCAAGAAGAAATGAACTACATCACTGCAATAATTGAGGAACAGCCCAAAAACTATCAAGTTTGGCACCATAGGAGAGTATTAGTGGAGTGGCTGAAAGATCCTTCTCAAGAGCTCGAGTTCATCGCCGATATCCTTAATCAGGATGCA
AAGAATTACCATGCCTGGCAGCATCGACAGTGGGTCATTCAGGAGTTTCGACTTTGGGATAATGAGCTGCAGTATGTGGACCAGCTTCTCAAAGAGGATGTGAGAAATAACTCTGTGTGGAACCAAAGACACTTCGTCATTTCTAATACCACTGGCTACAGTGATCGCGC
TGTGTTGGAGAGAGAAGTCCAATATACTCTGGAAATGATCAAATTAGTGCCACACAATGAGAGTGCGTGGAACTACTTGAAAGGGATTTTGCAGGACCGTGGTCTTTCCAGATACCCTAATCTATTAAACCAGTTGCTTGATTTACAACCAAGTCACAGCTCCCCCTACC
TAATTGCCTTTCTTGTGGATATCTATGAAGACATGCTGGAAAACCAGTGTGACAACAAGGAGGACATTCTTAATAAAGCACTAGAGTTATGTGAGATTCTAGCTAAAGAAAAGGACACTATAAGAAAGGAATATTGGAGATATATTGGACGGTCCCTCCAGAGTAAACAC
AGCAGAGAAAGTGACATACCGGCGAGTGTAGAGAATCTGTACTTCCAGGGCGCTTCTTCGAGTTCCTTCACCTATTATTGTCCTCCATCTTCTTCCCCTGTTTGGTCAGAACCGCTGTATAGTCTGAGACCTGAGCACGCGCGGGAGCGGTTGCAAGACGACTCAGTGGA
AACAGTCACGTCCATAGAACAGGCCAAAGTAGAAGAAAAGATCCAGGAGGTCTTCAGTTCTTACAAGTTTAACCACCTCGTACCAAGGCTCGTTCTGCAGAGGGAGAAGCACTTCCATTATCTGAAAAGAGGCCTTCGACAACTGACAGATGCCTATGAGTGTCTGGATG
CCAGCCGCCCCACGCTCTGCTACTGGATCCTGCACAGCTTGGAGCTCCTCGACGAACCCATCCCCCAAATAGTGGCTACAGATGTGTGTCAGTTCTTGGAGCTGTGTCAGAGTCCAGACGGTGGCTTTGGAGGGGGCCCTGGTCAGTACCCACACCTCGCTCCCACGTAT
GCAGCTGTCAACGCGCTATGCATCATTGGCACGGAGGAAGCCTACAACGTCATTAACAGAGAGAAGCTCCTTCAGTACTTGTACTCCCTAAAGCAACCGGATGGCTCTTTTCTCATGCACGTCGGAGGAGAGGTGGATGTAAGAAGTGCGTACTGTGCTGCCTCAGTAGC
CTCTCTCACCAACATCATCACTCCTGACCTCTTCGAAGGCACTGCTGAATGGATAGCAAGGTGCCAGAACTGGGAAGGCGGCATTGGCGGGGTGCCAGGGATGGAAGCCCACGGTGGCTACACCTTCTGTGGCTTGGCTGCGCTGGTGATCCTCAAGAAGGAACGTTCTT
TGAACCTGAAGAGCTTGCTACAATGGGTGACAAGCCGGCAGATGCGGTTCGAAGGAGGATTTCAGGGCCGCTGCAACAAGCTGGTGGACGGCTGCTACTCCTTCTGGCAGGCAGGACTTCTGCCCCTGTTGCACCGGGCACTCCACGCTCAAGGTGACCCTGCCCTCAGC
ATGAGCCACTGGATGTTCCATCAGCAGGCGCTGCAGGAGTACATCCTCATGTGCTGCCAGTGTCCGGCTGGGGGTCTCCTGGACAAACCTGGCAAGTCACGTGACTTCTACCATACTTGCTACTGCCTGAGCGGCCTGTCCATTGCCCAGCATTTTGGAAGTGGAGCCAT
GCTGCACGATGTGGTCATGGGTGTGCCTGAAAATGTTCTGCAGCCCACTCACCCTGTGTACAACATCGGACCTGATAAGGTGATCCAGGCCACCACACACTTTCTGCAGAAGCCGGTCCCAGGCTTTGAGGAATGCGAAGATGCGGTGACCTCAGATCCTGCCACTGACA
AGCTT

Cloning Strategy for Single Chain αβ-FTase => Constructs 05707 and 05708:

- Strategy: DNA assembly of fragments #1, #2 and #3 by means of USER mediated ligation followed by insertion into 05685 cut with BamHI and PmlI. Restriction sites are highlighted in red and adenine/uracil residues defining the splice sites for USER mediated assembly are highlighted in grey.

Fragment #1:

Template: 05685
Forward Primer: 5′-ACGCTCTGCTACTGGATCCTGC-3′ VS200
Reverse Primer: 5′-TCCAAAUCCACCGTCTGGACTCTGACAC-3′ VS201

PCR Product including BamHI:
ACGCTCTGCTACTGGATCCTGCACAGCTTGGAGCTCCTCGACGAACCCATCCCCCAAATAGTGGCTACAGATGTGTGTCAGTTCTTGGAGCTGTGTCAGAGTCCAGACGGTGGATTTGGA

Fragment #2:

Synthetic DNA Cassette Coding β-G142D Mutant

```
   5′-P-GATGGCCCTGGTCAGTACCCACACCTCGCTCCCACGTATGCAGCT-3′   VS204
3′-TAAACCTCTACCGGGACCAGTCATGGGTGTGGAGCGAGGGTGCATA-P-5′     VS208
```

Synthetic DNA Cassette Coding β-G142E Mutant

```
   5′-P-GAAGGCCCTGGTCAGTACCCACACCTCGCTCCCACGTATGCAGCT-3′   VS205
3′-TAAACCTCTTCCGGGACCAGTCATGGGTGTGGAGCGAGGGTGCATA-P-5′     VS209
```

Fragment #3:

Template: 05685
Forward Primer: 5′-GCAGCUGTCAACGCGCTATG-3′ VS210
Reverse Primer: 5′-GGTAGAAGTCACGTGACTTGCCAG-3′ VS211

PCR Product including PmlI:
GCAGCUGTCAACGCGCTATGCATCATTGGCACGGAGGAAGCCTACAACGTCATTAACAGAGAGAAGCTCCTTCAGTACTTGTACTCCCTAAAGCAACCGGATGGCTCTTTTCTCATGCACGTCGGAGGAGAGGTGGATGTAAGAAGTGCGTACTGTGCTGCCTCAGTAGC
CTCTCTCACCAACATCATCACTCCTGACCTCTTCGAAGGCACTGCTGAATGGATAGCAAGGTGCCAGAACTGGGAAGGCGGCATTGGCGGGGTGCCAGGGATGGAAGCCCACGGTGGCTACACCTTCTGTGGCTTGGCTGCGCTGGTGATCCTCAAGAAGGAACGTTCTT
TGAACCTGAAGAGCTTGCTACAATGGGTGACAAGCCGGCAGATGCGGTTCGAAGGAGGATTTCAGGGCCGCTGCAACAAGCTGGTGGACGGCTGCTACTCCTTCTGGCAGGCAGGACTTCTGCCCCTGTTGCACCGGGCACTCCACGCTCAAGGTGACCCTGCCCTCAGC
ATGAGCCACTGGATGTTCCATCAGCAGGCGCTGCAGGAGTACATCCTCATGTGCTGCCAGTGTCCGGCTGGGGGTCTCCTGGACAAACCTGGCAAGTCACGTGACTTCTACC

3. Assembly of Ion Torrent Sequencing Libraries

(1) PCR Amplify CAAX-Box Library from DNA Plasmid Preparations Following Selections

```
5′-GAGUGGCCCCGGCTGCATG-3′ (VS340)

5′-GAGUGGCCCCGGCTGCATGAGCTGTAAATGTNNNNNNNNNTGATAGTGATTTTGGGTTGATCCACTAGTAA-3′
3′-CTCACCGGGGCCGACGTACTCGACATTTACANNNNNNNNNACTATCACTAAAACCCAACTAGGTGAUCATT-5′

                                            3′-ATCACTAAAACCCAACTAGGTGAUCATT-5′      (VS296)
```

(2) Create 3′ Single Stranded Extensions by Treating PCR Product with USER Enzyme

```
  5′-P-GGCCCCGGCTGCATGAGCTGTAAATGTNNNNNNNNNTGATAGTGATTTTGGGTTGATCCACTAGTAA-3′
3′-CTCACCGGGGCCGACGTACTCGACATTTACANNNNNNNNNACTATCACTAAAACCCAACTAGGTGA-P-5′
```

(3) Introduce Flanking Regions for Ion Torrent Sequencing *via* Synthetic DNA Cassettes

```
5′-CCATCTCATCCCTGCGTGTCTCCGACTCAGGAGT-3′     (VS341)
3′-GGTAGAGTAGGGACGCACAGAGGCTGAGTC-P-5′       (VS342)

    5′-GGCCCCGGCTGCATGAGCTGTAAATGTNNNNNNNNNTGATAGTGATTTTGGGTTGATCCACTAGTAA-3′
3′-CTCACCGGGGCCGACGTACTCGACATTTACANNNNNNNNNACTATCACTAAAACCCAACTAGGTGA-5′

                                     5′-P-ATCACCGACTGCCCATAGAGAGG-3′     (VS343)
                                   3′-TCATTTAGTGGCTGACGGGTATCTCTCC-5′    (VS344)
```

(4) Fully Assembled DNA Product (124 bp)

```
          Ion Torrent Sequencing Primer

5′-CCATCTCATCCCTGCGTGTCTCCGACTCAG-3′

5′-CCATCTCATCCCTGCGTGTCTCCGACTCAGGAGTGGCCCCGGCTGCATGAGCTGTAAATGTNNNNNNNNNTGATAGTGATTTTGGGTTGATCCACTAGTAAATCACCGACTGCCCATAGAGAGG-3′
3′-GGTAGAGTAGGGACGCACAGAGGCTGAGTCCTCACCGGGGCCGACGTACTCGACATTTACANNNNNNNNNACTATCACTAAAACCCAACTAGGTGATCATTTAGTGGCTGACGGGTATCTCTCC-5′

                                                                                                 3′-TAGTGGCTGACGGGTATCTCTCC-5′

                                                                                             Ion Torrent Bead Immobilization Primer
```
