# Supplementary material for: Towards the Systematic Mapping and Engineering of the Protein Prenylation Machinery in Saccharomyces cerevisiae
Source: PLoS One. 2015 Mar 13;10(3):e0120716. doi: 10.1371/journal.pone.0120716 (PMC4358939; doi:10.1371/journal.pone.0120716)
Supplement: S2 File — (PDF) [file pone.0120716.s002.pdf]

			Counts	
**Motif**	**Enrichment**	**37 °C**	**25 °C**	**Naive**
-CLRS	0.0075329	1	53	134
-CVGS	0.0084946	1	47	45
-CTSG	0.010237	1	39	54
-CLPS	0.010506	1	38	79
-CAKS	0.01079	1	37	33
-CIPL	0.011743	1	34	36
-CTNV	0.012098	1	33	41
-CNTS	0.012476	1	32	56
-CPTL	0.012476	1	32	55
-CPTS	0.012476	1	32	66
-CDTS	0.012879	1	31	39
-CLTE	0.012879	1	31	46
-CPRL	0.012879	1	31	59
-CRES	0.012879	1	31	26
-CSKL	0.012879	1	31	54
-CRTG	0.013308	1	30	62
-CSLG	0.013308	1	30	66
-CEVR	0.014259	1	28	41
-CKSS	0.014518	2	55	56
-CFSV	0.014787	1	27	35
-CLDI	0.014787	1	27	19
-CLKT	0.014787	1	27	35
-CLNR	0.014787	1	27	76
-CLST	0.014787	2	54	65
-CLTT	0.014787	1	27	86
-CSDP	0.014787	1	27	39
-CERR	0.015356	1	26	52
-CSGI	0.015356	1	26	29
-CSTI	0.015356	1	26	46
-CVRE	0.015356	1	26	26
-CYAL	0.015356	1	26	56
-CDSK	0.01597	1	25	29
-CRSK	0.01597	1	25	32
-CVPR	0.01597	1	25	37
-CFKS	0.016635	1	24	24
-CGSF	0.016635	1	24	19
-CKLP	0.016635	1	24	43
-CKSV	0.016635	1	24	39
-CLGT	0.016635	1	24	38
-CLSL	0.01687	3	71	118
-CPEL	0.016989	2	47	39
-CERK	0.017359	1	23	19
-CLGK	0.017359	1	23	33
-CLTN	0.017359	1	23	50
-CNTR	0.017359	1	23	39
-CQSP	0.017359	1	23	34
-CTGS	0.017359	1	23	69
-CVDR	0.017359	1	23	31
-CYED	0.017359	1	23	16
-CGLV	0.017744	2	45	56
-CARL	0.018148	1	22	62
-CASR	0.018148	1	22	64
-CGSK	0.018148	1	22	40

-CIGI	0.018148	1	22	22
-CNGV	0.018148	1	22	38
-CPGL	0.018148	1	22	57
-CSPV	0.018148	1	22	47
-CSQV	0.018148	1	22	48
-CSTN	0.018148	1	22	48
-CTLP	0.018148	1	22	48
-CTPV	0.018148	1	22	48
-CVNR	0.018148	1	22	49
-CYHS	0.018148	1	22	37
-CLFT	0.019012	1	21	38
-CSPN	0.019012	1	21	47
-CSRD	0.019012	1	21	34
-CTKS	0.019012	1	21	21
-CTSC	0.019012	1	21	27
-CVAI	0.019012	1	21	34
-CLSE	0.019475	2	41	50
-CTAR	0.019475	2	41	74
-CAKT	0.019962	1	20	34
-CDDV	0.019962	1	20	29
-CLNT	0.019962	2	40	55
-CMGL	0.019962	1	20	37
-CPER	0.019962	1	20	33
-CSIE	0.019962	1	20	33
-CTKL	0.019962	1	20	48
-CVDG	0.019962	1	20	24
-CVTP	0.019962	1	20	35
-CYTT	0.019962	1	20	38
-CSRG	0.020474	2	39	62
-CADS	0.021013	1	19	27
-CCTE	0.021013	1	19	29
-CDFP	0.021013	1	19	20
-CDGT	0.021013	1	19	29
-CEAL	0.021013	1	19	47
-CFPL	0.021013	1	19	52
-CGQS	0.021013	1	19	32
-CGRT	0.021013	1	19	57
-CLDK	0.021013	1	19	23
-CLDY	0.021013	1	19	38
-CLRN	0.021013	1	19	41
-CLTP	0.021013	1	19	53
-CNSS	0.021013	2	38	41
-CPVP	0.021013	1	19	56
-CRTL	0.021013	2	38	83
-CVPI	0.021013	1	19	13
-CWST	0.021013	1	19	38
-CERS	0.021581	2	37	55
-CSNR	0.021581	2	37	58
-CVLG	0.021581	2	37	57
-CVST	0.021581	2	37	69
-CESM	0.02218	1	18	31
-CEVD	0.02218	1	18	6
-CFEA	0.02218	1	18	11
-CFTN	0.02218	1	18	7
-CGNV	0.02218	1	18	21
-CGYV	0.02218	1	18	22
-CIEL	0.02218	1	18	18

-CKSG	0.02218	1	18	27
-CKYV	0.02218	1	18	27
-CLGP	0.02218	1	18	27
-CLGV	0.02218	1	18	50
-CLPG	0.02218	1	18	19
-CNSN	0.02218	1	18	31
-CPLL	0.02218	2	36	83
-CPLR	0.02218	1	18	77
-CPTG	0.02218	1	18	34
-CQSA	0.02218	1	18	25
-CSDT	0.02218	1	18	24
-CSNE	0.02218	1	18	15
-CSTE	0.02218	1	18	23
-CSTK	0.02218	1	18	35
-CSTT	0.02218	2	36	81
-CSYR	0.02218	1	18	64
-CTPT	0.02218	1	18	34
-CTSW	0.02218	1	18	47
-CVSI	0.02218	1	18	41
-CYST	0.02218	1	18	45
-CETL	0.022814	2	35	38
-CLTR	0.022814	2	35	115
-CMSV	0.022814	2	35	40
-CSVD	0.023061	16	277	162
-CDDM	0.023485	1	17	16
-CDKL	0.023485	1	17	36
-CEEV	0.023485	1	17	14
-CFSA	0.023485	1	17	16
-CGPR	0.023485	1	17	51
-CIET	0.023485	1	17	15
-CISD	0.023485	1	17	28
-CKTG	0.023485	1	17	21
-CLAV	0.023485	1	17	41
-CLDM	0.023485	1	17	17
-CLET	0.023485	1	17	63
-CLGA	0.023485	1	17	46
-CLGM	0.023485	1	17	30
-CMES	0.023485	1	17	14
-CNAT	0.023485	1	17	22
-CNGL	0.023485	1	17	22
-CNSR	0.023485	2	34	52
-CPKL	0.023485	1	17	35
-CPLK	0.023485	2	34	36
-CPSR	0.023485	2	34	82
-CQTV	0.023485	1	17	23
-CSAT	0.023485	1	17	49
-CSDV	0.023485	2	34	43
-CSKG	0.023485	1	17	44
-CVKL	0.023485	1	17	40
-CVKV	0.023485	1	17	39
-CVRN	0.023485	1	17	36
-CVRT	0.023485	1	17	51
-CVSG	0.023485	1	17	44
-CYEV	0.023485	1	17	18
-CETV	0.024197	2	33	37
-CSTV	0.024197	2	33	65
-CCES	0.024953	1	16	26

-CCLD	0.024953	1	16	40
-CDAS	0.024953	1	16	26
-CDKG	0.024953	1	16	10
-CESF	0.024953	1	16	36
-CFTG	0.024953	1	16	31
-CGKV	0.024953	1	16	26
-CGSN	0.024953	1	16	38
-CIPT	0.024953	1	16	33
-CKFT	0.024953	1	16	22
-CKTE	0.024953	1	16	20
-CLDF	0.024953	1	16	19
-CLDS	0.024953	2	32	53
-CLGL	0.024953	2	32	85
-CMNV	0.024953	1	16	30
-CMPA	0.024953	1	16	25
-CMTP	0.024953	1	16	16
-CNTF	0.024953	1	16	25
-CPML	0.024953	1	16	26
-CQRK	0.024953	1	16	22
-CQSV	0.024953	1	16	21
-CRDT	0.024953	1	16	23
-CRKL	0.024953	1	16	61
-CRNR	0.024953	1	16	42
-CTGQ	0.024953	1	16	15
-CTPL	0.024953	1	16	38
-CVGM	0.024953	1	16	29
-CVPN	0.024953	1	16	27
-CVQE	0.024953	1	16	15
-CVSE	0.025758	2	31	48
-CAKN	0.026616	1	15	41
-CDDT	0.026616	1	15	8
-CDKF	0.026616	1	15	9
-CEAM	0.026616	1	15	30
-CEFD	0.026616	1	15	11
-CESW	0.026616	1	15	15
-CFPE	0.026616	1	15	12
-CGTF	0.026616	1	15	33
-CHSG	0.026616	1	15	30
-CISK	0.026616	1	15	28
-CKNR	0.026616	1	15	19
-CKPN	0.026616	1	15	25
-CLEP	0.026616	1	15	30
-CLNM	0.026616	1	15	30
-CLYT	0.026616	1	15	33
-CMGV	0.026616	1	15	34
-CNSA	0.026616	2	30	39
-CPFS	0.026616	1	15	22
-CPTF	0.026616	1	15	32
-CPVG	0.026616	1	15	40
-CRPR	0.026616	1	15	58
-CSAK	0.026616	1	15	31
-CSDC	0.026616	1	15	15
-CSNI	0.026616	1	15	13
-CSPT	0.026616	2	30	69
-CTKV	0.026616	1	15	27
-CTMG	0.026616	1	15	17
-CTNI	0.026616	1	15	27

-CTYR	0.026616	1	15	49
-CVAL	0.026616	1	15	37
-CYIK	0.026616	1	15	11
-CGLT	0.027534	2	29	47
-CKLL	0.027534	2	29	65
-CSKS	0.027534	2	29	67
-CTTG	0.027534	2	29	50
-CLTV	0.027854	3	43	78
-CALD	0.028518	1	14	36
-CAME	0.028518	1	14	18
-CASP	0.028518	1	14	52
-CDCL	0.028518	1	14	25
-CDEE	0.028518	1	14	5
-CDEQ	0.028518	1	14	11
-CDGG	0.028518	1	14	17
-CDLD	0.028518	1	14	18
-CEKS	0.028518	1	14	17
-CETC	0.028518	1	14	13
-CGGV	0.028518	1	14	23
-CGHS	0.028518	1	14	25
-CGKF	0.028518	1	14	20
-CGKR	0.028518	1	14	53
-CGSM	0.028518	1	14	33
-CIEG	0.028518	1	14	15
-CIGR	0.028518	1	14	35
-CIKT	0.028518	1	14	19
-CISI	0.028518	2	28	51
-CIVK	0.028518	1	14	19
-CKGI	0.028518	1	14	12
-CKML	0.028518	1	14	29
-CKPS	0.028518	1	14	37
-CKTP	0.028518	1	14	27
-CKTT	0.028518	1	14	23
-CLEA	0.028518	1	14	17
-CLEM	0.028518	1	14	25
-CLHT	0.028518	1	14	24
-CLKN	0.028518	1	14	26
-CMKS	0.028518	1	14	16
-CMSP	0.028518	1	14	31
-CNEF	0.028518	1	14	16
-CNMK	0.028518	1	14	15
-CNML	0.028518	1	14	41
-CNQN	0.028518	1	14	26
-CNSD	0.028518	1	14	22
-CNTG	0.028518	1	14	41
-CPDE	0.028518	1	14	16
-CPDG	0.028518	1	14	24
-CPGY	0.028518	1	14	10
-CPIG	0.028518	1	14	34
-CPNE	0.028518	1	14	16
-CPRE	0.028518	1	14	36
-CQDV	0.028518	1	14	14
-CRAL	0.028518	2	28	62
-CRTA	0.028518	1	14	55
-CSHT	0.028518	1	14	34
-CTRE	0.028518	1	14	31
-CTRN	0.028518	1	14	46

-CVRG	0.028518	1	14	27
-CVRH	0.028518	1	14	26
-CVSR	0.028518	2	28	54
-CVTD	0.028518	1	14	22
-CPRS	0.028518	3	42	96
-CPLT	0.029574	2	27	55
-CSES	0.029574	4	54	37
-CSNS	0.029943	3	40	70
-CAAS	0.030711	1	13	43
-CAKM	0.030711	1	13	17
-CANR	0.030711	1	13	43
-CASC	0.030711	1	13	34
-CASG	0.030711	1	13	38
-CATG	0.030711	1	13	38
-CCPL	0.030711	1	13	27
-CCWA	0.030711	1	13	13
-CDGE	0.030711	1	13	15
-CDNS	0.030711	1	13	31
-CEGC	0.030711	2	26	26
-CELE	0.030711	1	13	22
-CERV	0.030711	1	13	12
-CESE	0.030711	1	13	20
-CESG	0.030711	1	13	29
-CETT	0.030711	1	13	33
-CFDM	0.030711	1	13	15
-CFQE	0.030711	1	13	14
-CGSG	0.030711	1	13	39
-CGTE	0.030711	1	13	16
-CHLK	0.030711	1	13	32
-CHSE	0.030711	1	13	25
-CKAR	0.030711	1	13	28
-CKGR	0.030711	1	13	19
-CKLE	0.030711	1	13	23
-CKNT	0.030711	1	13	20
-CKPL	0.030711	1	13	24
-CKRK	0.030711	1	13	27
-CKSN	0.030711	1	13	28
-CLNP	0.030711	1	13	41
-CLNS	0.030711	2	26	33
-CLPV	0.030711	2	26	56
-CLSF	0.030711	1	13	36
-CLSP	0.030711	1	13	60
-CLTD	0.030711	1	13	42
-CLTK	0.030711	2	26	25
-CMAS	0.030711	1	13	29
-CPAT	0.030711	1	13	45
-CPGA	0.030711	1	13	19
-CPTR	0.030711	2	26	41
-CPVV	0.030711	1	13	51
-CQDR	0.030711	1	13	16
-CQMK	0.030711	1	13	10
-CQSN	0.030711	1	13	19
-CRDQ	0.030711	1	13	11
-CRGV	0.030711	1	13	40
-CRLK	0.030711	1	13	39
-CSAP	0.030711	1	13	30
-CSHK	0.030711	1	13	16

-CSKM	0.030711	1	13	19
-CSKR	0.030711	1	13	54
-CSQG	0.030711	1	13	30
-CTDK	0.030711	1	13	16
-CTEI	0.030711	1	13	26
-CTEV	0.030711	1	13	26
-CTGL	0.030711	1	13	36
-CTTN	0.030711	1	13	29
-CVAG	0.030711	1	13	19
-CVEA	0.030711	1	13	24
-CVER	0.030711	1	13	35
-CVPL	0.030711	1	13	47
-CVVK	0.030711	1	13	28
-CYEE	0.030711	1	13	14
-CYEG	0.030711	1	13	24
-CYSF	0.030711	1	13	14
-CYSP	0.030711	1	13	25
-CYTL	0.030711	1	13	45
-CSSR	0.031313	4	51	105
-CREL	0.031519	3	38	46
-CATL	0.03194	2	25	56
-CFSN	0.03194	2	25	31
-CFST	0.03194	2	25	31
-CLSK	0.03194	2	25	57
-CREV	0.03194	2	25	42
-CTDM	0.03194	2	25	18
-CTDS	0.03194	2	25	43
-CYSL	0.03194	2	25	66
-CAKP	0.03327	1	12	27
-CANE	0.03327	1	12	12
-CCKK	0.03327	1	12	18
-CDFR	0.03327	1	12	39
-CDFT	0.03327	1	12	11
-CDGN	0.03327	1	12	12
-CDHF	0.03327	1	12	16
-CDIP	0.03327	1	12	18
-CDKY	0.03327	1	12	13
-CDLG	0.03327	1	12	15
-CDNV	0.03327	1	12	12
-CDNY	0.03327	1	12	24
-CELK	0.03327	1	12	18
-CERG	0.03327	1	12	27
-CERI	0.03327	1	12	13
-CETK	0.03327	1	12	13
-CFGR	0.03327	1	12	32
-CFGV	0.03327	1	12	26
-CGGE	0.03327	1	12	8
-CGMD	0.03327	1	12	15
-CGMS	0.03327	1	12	26
-CGRF	0.03327	1	12	18
-CGTY	0.03327	1	12	27
-CHFS	0.03327	1	12	32
-CHSP	0.03327	2	24	46
-CIGE	0.03327	1	12	11
-CIGP	0.03327	1	12	39
-CIRT	0.03327	2	24	44
-CITG	0.03327	1	12	11

-CKAN	0.03327	1	12	15
-CKDE	0.03327	1	12	21
-CKGM	0.03327	1	12	20
-CKLD	0.03327	1	12	25
-CKLK	0.03327	1	12	40
-CKRM	0.03327	1	12	29
-CKRR	0.03327	1	12	44
-CKVR	0.03327	2	24	34
-CKYL	0.03327	1	12	14
-CLAT	0.03327	1	12	44
-CLEH	0.03327	1	12	17
-CLFI	0.03327	1	12	17
-CLGI	0.03327	1	12	23
-CLNE	0.03327	1	12	23
-CMNA	0.03327	1	12	10
-CMTA	0.03327	1	12	13
-CMTV	0.03327	1	12	24
-CNEV	0.03327	1	12	25
-CNIE	0.03327	1	12	8
-CNST	0.03327	1	12	37
-CNTE	0.03327	1	12	30
-CPKE	0.03327	1	12	21
-CPMA	0.03327	1	12	19
-CQCL	0.03327	1	12	26
-CQDS	0.03327	1	12	17
-CQGT	0.03327	1	12	24
-CQLK	0.03327	1	12	13
-CQRT	0.03327	1	12	34
-CRAE	0.03327	1	12	28
-CRNA	0.03327	1	12	40
-CRND	0.03327	1	12	15
-CRNE	0.03327	1	12	39
-CSAF	0.03327	1	12	30
-CSHR	0.03327	1	12	36
-CSKI	0.03327	1	12	36
-CTKW	0.03327	1	12	31
-CTPI	0.03327	1	12	21
-CTRL	0.03327	2	24	100
-CTRT	0.03327	2	24	52
-CTYK	0.03327	1	12	25
-CVEM	0.03327	1	12	23
-CVGV	0.03327	2	24	27
-CVRA	0.03327	2	24	77
-CVSP	0.03327	1	12	49
-CVTA	0.03327	1	12	38
-CVYP	0.03327	1	12	36
-CVYS	0.03327	1	12	45
-CWEN	0.03327	1	12	2
-CWPG	0.03327	1	12	12
-CYAT	0.03327	1	12	20
-CYCA	0.03327	1	12	22
-CYQL	0.03327	1	12	40
-CYSI	0.03327	1	12	17
-CGVL	0.03327	3	36	71
-CSSG	0.03327	3	36	77
-CSTS	0.03327	3	36	117
-CTTV	0.034221	3	35	48

-CART	0.034717	2	23	64
-CIKS	0.034717	2	23	39
-CPRR	0.034717	2	23	66
-CPSE	0.034717	2	23	38
-CPTE	0.034717	2	23	21
-CSRK	0.034717	2	23	51
-CTPR	0.034717	2	23	45
-CVQG	0.034717	2	23	31
-CAGV	0.036295	1	11	25
-CAPE	0.036295	1	11	17
-CARE	0.036295	1	11	20
-CAYG	0.036295	1	11	27
-CCDE	0.036295	1	11	12
-CCDT	0.036295	1	11	11
-CDFA	0.036295	1	11	17
-CDGI	0.036295	1	11	24
-CDGR	0.036295	1	11	29
-CDHT	0.036295	1	11	20
-CDKI	0.036295	1	11	11
-CDSE	0.036295	1	11	18
-CDSS	0.036295	4	44	53
-CECA	0.036295	1	11	11
-CEFK	0.036295	1	11	15
-CEGR	0.036295	1	11	23
-CERD	0.036295	1	11	22
-CERY	0.036295	1	11	16
-CESK	0.036295	2	22	34
-CETS	0.036295	1	11	18
-CEYE	0.036295	1	11	12
-CEYV	0.036295	1	11	15
-CFDV	0.036295	1	11	18
-CFKT	0.036295	1	11	8
-CGAM	0.036295	1	11	12
-CGDK	0.036295	1	11	21
-CGFD	0.036295	1	11	19
-CGFS	0.036295	1	11	26
-CGGD	0.036295	1	11	11
-CGLN	0.036295	1	11	48
-CGTQ	0.036295	1	11	28
-CGTV	0.036295	1	11	28
-CIRK	0.036295	1	11	33
-CKCY	0.036295	1	11	18
-CKNG	0.036295	2	22	21
-CKPR	0.036295	1	11	26
-CKQK	0.036295	1	11	16
-CKRG	0.036295	1	11	31
-CLKR	0.036295	2	22	50
-CLKV	0.036295	1	11	47
-CLNI	0.036295	1	11	19
-CLRY	0.036295	1	11	51
-CLTI	0.036295	2	22	36
-CMDT	0.036295	1	11	14
-CMPK	0.036295	1	11	26
-CMPV	0.036295	1	11	28
-CNDE	0.036295	1	11	19
-CNTD	0.036295	2	22	15
-CNTV	0.036295	1	11	41

-CNVY	0.036295	1	11	25
-CPFA	0.036295	1	11	34
-CPLD	0.036295	1	11	24
-CPTA	0.036295	1	11	27
-CQAL	0.036295	1	11	30
-CQAS	0.036295	1	11	28
-CQKL	0.036295	1	11	22
-CQRL	0.036295	1	11	30
-CRCE	0.036295	1	11	24
-CRHG	0.036295	1	11	37
-CRRG	0.036295	2	22	52
-CRSN	0.036295	1	11	48
-CRTT	0.036295	2	22	48
-CRWS	0.036295	1	11	46
-CRWT	0.036295	1	11	17
-CRYD	0.036295	1	11	39
-CTEG	0.036295	1	11	27
-CTER	0.036295	1	11	34
-CTES	0.036295	3	33	46
-CTGE	0.036295	1	11	18
-CTGK	0.036295	1	11	29
-CTHT	0.036295	1	11	29
-CTKG	0.036295	1	11	26
-CTKK	0.036295	1	11	32
-CTNC	0.036295	1	11	36
-CTNL	0.036295	2	22	42
-CTRK	0.036295	1	11	39
-CTSD	0.036295	2	22	32
-CVAD	0.036295	1	11	22
-CVAP	0.036295	1	11	36
-CVEK	0.036295	1	11	19
-CVEV	0.036295	1	11	17
-CVKA	0.036295	1	11	14
-CVLI	0.036295	1	11	42
-CVMG	0.036295	1	11	40
-CVRM	0.036295	1	11	37
-CYAI	0.036295	1	11	17
-CYEL	0.036295	1	11	17
-CYGA	0.036295	1	11	22
-CYSR	0.036295	1	11	30
-CYTS	0.036295	2	22	46
-CGPS	0.037429	3	32	57
-CSTA	0.037429	3	32	61
-CDSV	0.038023	2	21	38
-CDVT	0.038023	2	21	58
-CGLD	0.038023	2	21	43
-CLNK	0.038023	2	21	25
-CPQK	0.038023	2	21	15
-CRPL	0.038023	2	21	49
-CRPV	0.038023	2	21	38
-CSQS	0.038023	2	21	51
-CSTL	0.038023	4	42	113
-CYET	0.038023	2	21	24
-CYSN	0.038023	2	21	26
-CEGV	0.038637	3	31	32
-CLLK	0.038637	3	31	73
-CLTG	0.038637	3	31	38

-CPSL	0.039142	5	51	95
-CAAG	0.039925	1	10	16
-CAEG	0.039925	1	10	10
-CAHN	0.039925	1	10	32
-CALI	0.039925	1	10	37
-CANI	0.039925	1	10	7
-CARM	0.039925	1	10	30
-CASE	0.039925	1	10	29
-CCEN	0.039925	1	10	17
-CCGE	0.039925	1	10	23
-CCSI	0.039925	1	10	27
-CCTT	0.039925	1	10	27
-CDGV	0.039925	1	10	31
-CDHG	0.039925	1	10	6
-CDLF	0.039925	1	10	14
-CDME	0.039925	2	20	17
-CDNM	0.039925	1	10	27
-CDNN	0.039925	1	10	24
-CDPV	0.039925	2	20	37
-CDSF	0.039925	1	10	25
-CEAV	0.039925	1	10	11
-CEER	0.039925	1	10	12
-CEEY	0.039925	1	10	12
-CEFP	0.039925	1	10	13
-CEGL	0.039925	1	10	32
-CENR	0.039925	1	10	21
-CEQC	0.039925	1	10	12
-CERM	0.039925	2	20	25
-CERQ	0.039925	1	10	14
-CESA	0.039925	1	10	24
-CFPH	0.039925	1	10	12
-CFTK	0.039925	1	10	12
-CGGS	0.039925	2	20	40
-CGKL	0.039925	2	20	44
-CGTN	0.039925	1	10	20
-CGVD	0.039925	1	10	21
-CHGD	0.039925	1	10	9
-CHSS	0.039925	2	20	35
-CHST	0.039925	1	10	51
-CHTE	0.039925	1	10	20
-CIPR	0.039925	1	10	24
-CIRQ	0.039925	1	10	22
-CISG	0.039925	2	20	27
-CKGS	0.039925	1	10	23
-CKKC	0.039925	1	10	12
-CKNS	0.039925	1	10	21
-CKPT	0.039925	1	10	17
-CLAK	0.039925	2	20	34
-CLAR	0.039925	3	30	59
-CLES	0.039925	2	20	48
-CLEY	0.039925	1	10	17
-CLFD	0.039925	1	10	22
-CLKD	0.039925	1	10	12
-CLKK	0.039925	1	10	19
-CLNV	0.039925	2	20	31
-CLPL	0.039925	2	20	116
-CLYA	0.039925	1	10	30

-CMEF	0.039925	1	10	15
-CMRS	0.039925	2	20	48
-CMSE	0.039925	2	20	34
-CMTE	0.039925	1	10	20
-CMTS	0.039925	2	20	43
-CNAW	0.039925	1	10	17
-CNDS	0.039925	1	10	23
-CNEL	0.039925	1	10	32
-CNLK	0.039925	2	20	40
-CNRF	0.039925	1	10	18
-CNRG	0.039925	1	10	36
-CNSF	0.039925	1	10	41
-CPAW	0.039925	1	10	27
-CPGQ	0.039925	1	10	10
-CPHE	0.039925	1	10	16
-CPMS	0.039925	1	10	45
-CPNS	0.039925	3	30	34
-CPQT	0.039925	1	10	24
-CPQV	0.039925	1	10	24
-CPRQ	0.039925	1	10	29
-CPYS	0.039925	1	10	34
-CQET	0.039925	1	10	16
-CQNM	0.039925	1	10	12
-CQTR	0.039925	1	10	40
-CRAK	0.039925	2	20	46
-CRAQ	0.039925	1	10	30
-CRDN	0.039925	1	10	23
-CREI	0.039925	1	10	14
-CRKR	0.039925	2	20	44
-CRKS	0.039925	2	20	55
-CRNY	0.039925	1	10	21
-CRTI	0.039925	1	10	19
-CSEE	0.039925	1	10	15
-CSKD	0.039925	2	20	18
-CSKP	0.039925	1	10	35
-CSNA	0.039925	1	10	32
-CSRN	0.039925	2	20	60
-CSVK	0.039925	2	20	42
-CTAH	0.039925	1	10	18
-CTCG	0.039925	1	10	14
-CTGD	0.039925	1	10	6
-CTNG	0.039925	1	10	20
-CTQN	0.039925	1	10	26
-CTSN	0.039925	2	20	40
-CTWV	0.039925	1	10	33
-CVAV	0.039925	2	20	20
-CVDN	0.039925	1	10	19
-CVEG	0.039925	1	10	15
-CVHN	0.039925	1	10	23
-CVHS	0.039925	1	10	50
-CVSY	0.039925	1	10	32
-CWGV	0.039925	1	10	23
-CWTK	0.039925	1	10	9
-CWTR	0.039925	1	10	32
-CYHF	0.039925	1	10	11
-CYLE	0.039925	1	10	29
-CYRE	0.039925	1	10	25

-CYRM	0.039925	1	10	24
-CYTH	0.039925	1	10	14
-CYWR	0.039925	1	10	28
-CELT	0.041301	3	29	36
-CLKL	0.041301	3	29	68
-CQTE	0.041301	3	29	17
-CSGL	0.041301	3	29	77
-CSKV	0.041301	3	29	77
-CTSK	0.041301	3	29	40
-CEES	0.042026	2	19	15
-CESD	0.042026	2	19	21
-CGEE	0.042026	2	19	13
-CGLE	0.042026	2	19	43
-CISR	0.042026	2	19	33
-CLAE	0.042026	2	19	19
-CLAN	0.042026	2	19	60
-CLQV	0.042026	2	19	36
-CLSI	0.042026	2	19	36
-CMSL	0.042026	4	38	64
-CNKT	0.042026	2	19	19
-CPPK	0.042026	2	19	18
-CPRV	0.042026	2	19	55
-CQDL	0.042026	2	19	32
-CRAS	0.042026	2	19	51
-CRTE	0.042026	2	19	25
-CSGE	0.042026	2	19	41
-CSLK	0.042026	2	19	64
-CSRI	0.042026	2	19	47
-CSSW	0.042026	2	19	51
-CVAN	0.042026	2	19	25
-CVGA	0.042026	2	19	20
-CVPK	0.042026	2	19	21
-CVRD	0.042026	2	19	30
-CLRV	0.042473	5	47	74
-CLGS	0.042776	3	28	71
-CLPP	0.042776	3	28	30
-CPTT	0.042776	3	28	42
-CTGT	0.042776	3	28	40
-CAAV	0.044361	1	9	30
-CADT	0.044361	1	9	31
-CAFD	0.044361	1	9	23
-CAHS	0.044361	1	9	27
-CAIR	0.044361	1	9	38
-CAKA	0.044361	1	9	24
-CAKG	0.044361	2	18	27
-CALE	0.044361	1	9	29
-CANV	0.044361	1	9	12
-CAQL	0.044361	2	18	42
-CAQN	0.044361	1	9	12
-CARP	0.044361	1	9	43
-CAVG	0.044361	1	9	22
-CCTA	0.044361	1	9	5
-CDAI	0.044361	1	9	22
-CDCD	0.044361	2	18	8
-CDDE	0.044361	1	9	14
-CDGS	0.044361	2	18	39
-CDKT	0.044361	1	9	21

-CDYR	0.044361	1	9	30
-CEAI	0.044361	1	9	13
-CEDP	0.044361	1	9	14
-CEDR	0.044361	1	9	8
-CEFL	0.044361	1	9	19
-CEFS	0.044361	1	9	23
-CEHN	0.044361	1	9	17
-CEIG	0.044361	1	9	11
-CEKQ	0.044361	1	9	5
-CEKR	0.044361	1	9	21
-CEMA	0.044361	1	9	16
-CENH	0.044361	2	18	20
-CEPC	0.044361	1	9	15
-CEPF	0.044361	1	9	13
-CFEL	0.044361	1	9	20
-CFGT	0.044361	1	9	17
-CFPR	0.044361	1	9	26
-CFPT	0.044361	1	9	16
-CFTV	0.044361	1	9	26
-CFVN	0.044361	1	9	23
-CFVP	0.044361	1	9	22
-CGAA	0.044361	1	9	12
-CGAQ	0.044361	1	9	19
-CGEA	0.044361	1	9	16
-CGEY	0.044361	1	9	12
-CGGI	0.044361	2	18	17
-CGMV	0.044361	1	9	16
-CGND	0.044361	1	9	11
-CHEF	0.044361	1	9	11
-CHIE	0.044361	1	9	10
-CHPL	0.044361	1	9	21
-CHRE	0.044361	1	9	29
-CHTP	0.044361	1	9	19
-CIGG	0.044361	1	9	16
-CIHD	0.044361	1	9	20
-CIHR	0.044361	1	9	18
-CIRN	0.044361	1	9	14
-CITE	0.044361	2	18	11
-CIYK	0.044361	1	9	31
-CKAM	0.044361	1	9	12
-CKAV	0.044361	1	9	19
-CKCV	0.044361	1	9	11
-CKDV	0.044361	2	18	14
-CKKI	0.044361	1	9	12
-CKMK	0.044361	1	9	11
-CKPG	0.044361	1	9	16
-CKRQ	0.044361	1	9	14
-CKVV	0.044361	1	9	25
-CLDG	0.044361	1	9	17
-CLDN	0.044361	1	9	20
-CLGD	0.044361	1	9	20
-CLNC	0.044361	1	9	17
-CLPE	0.044361	2	18	32
-CLPH	0.044361	2	18	13
-CLQG	0.044361	1	9	26
-CLTH	0.044361	1	9	35
-CLVK	0.044361	1	9	18

-CMDG	0.044361	1	9	15
-CMEV	0.044361	1	9	15
-CMGG	0.044361	1	9	13
-CMRQ	0.044361	1	9	30
-CMSN	0.044361	1	9	20
-CNAN	0.044361	1	9	19
-CNEY	0.044361	1	9	9
-CNIK	0.044361	1	9	8
-CNNS	0.044361	2	18	41
-CNRD	0.044361	2	18	19
-CNSP	0.044361	1	9	31
-CNSW	0.044361	1	9	27
-CNTC	0.044361	1	9	23
-CNTT	0.044361	1	9	24
-CPCY	0.044361	1	9	7
-CPEC	0.044361	1	9	4
-CPMV	0.044361	1	9	36
-CPQY	0.044361	1	9	7
-CPTW	0.044361	1	9	26
-CPTY	0.044361	1	9	12
-CPYG	0.044361	1	9	13
-CQGL	0.044361	1	9	16
-CQMI	0.044361	1	9	9
-CQNH	0.044361	1	9	19
-CRDC	0.044361	1	9	22
-CREY	0.044361	1	9	17
-CRKM	0.044361	1	9	17
-CRKT	0.044361	2	18	44
-CRMG	0.044361	1	9	40
-CRPP	0.044361	1	9	39
-CRQR	0.044361	1	9	47
-CRTV	0.044361	2	18	44
-CRVI	0.044361	1	9	40
-CSAR	0.044361	2	18	79
-CSDW	0.044361	1	9	13
-CSGH	0.044361	1	9	25
-CSHS	0.044361	2	18	46
-CSKF	0.044361	1	9	17
-CSKW	0.044361	1	9	26
-CSLD	0.044361	2	18	47
-CSLY	0.044361	1	9	30
-CSPH	0.044361	1	9	19
-CSPY	0.044361	1	9	31
-CSTY	0.044361	1	9	35
-CSVG	0.044361	2	18	36
-CSWT	0.044361	1	9	24
-CSYY	0.044361	1	9	41
-CTED	0.044361	2	18	29
-CTGG	0.044361	1	9	19
-CTKT	0.044361	1	9	25
-CTPK	0.044361	1	9	23
-CTRI	0.044361	1	9	31
-CTTD	0.044361	1	9	12
-CTTL	0.044361	2	18	83
-CTTW	0.044361	1	9	39
-CTVK	0.044361	2	18	24
-CVEE	0.044361	1	9	16

-CVGG	0.044361	1	9	28
-CVKC	0.044361	1	9	12
-CVNC	0.044361	1	9	14
-CVNF	0.044361	1	9	11
-CVQI	0.044361	1	9	12
-CVYV	0.044361	1	9	17
-CWSG	0.044361	1	9	18
-CYAA	0.044361	1	9	13
-CYDQ	0.044361	1	9	11
-CYES	0.044361	1	9	25
-CYGL	0.044361	1	9	31
-CYGV	0.044361	1	9	18
-CYRG	0.044361	1	9	14
-CYVK	0.044361	1	9	38
-CFSS	0.044361	3	27	37
-CKAL	0.044361	3	27	38
-CNGS	0.044361	3	27	43
-CRRD	0.044361	3	27	46
-CRSR	0.044361	3	27	77
-CSET	0.044361	3	27	52
-CDLR	0.045369	5	44	54
-CSDS	0.045628	4	35	54
-CARS	0.046067	3	26	78
-CDGL	0.046067	3	26	46
-CESL	0.046067	3	26	42
-CLSA	0.046067	3	26	97
-CRTS	0.046067	3	26	71
-CTRS	0.046424	5	43	94
-CAKV	0.04697	2	17	39
-CATV	0.04697	2	17	57
-CDEL	0.04697	2	17	24
-CDFN	0.04697	2	17	21
-CDGM	0.04697	2	17	22
-CDST	0.04697	2	17	42
-CDTL	0.04697	4	34	46
-CESH	0.04697	2	17	32
-CGLA	0.04697	2	17	51
-CGSE	0.04697	2	17	24
-CGSR	0.04697	4	34	50
-CITR	0.04697	2	17	74
-CKLG	0.04697	2	17	33
-CKVT	0.04697	2	17	24
-CLEL	0.04697	2	17	37
-CLFE	0.04697	2	17	31
-CLRT	0.04697	2	17	70
-CPKV	0.04697	2	17	27
-CPVK	0.04697	2	17	26
-CRFE	0.04697	2	17	35
-CRSP	0.04697	2	17	56
-CSCT	0.04697	2	17	43
-CTAP	0.04697	2	17	41
-CTKA	0.04697	2	17	22
-CTTK	0.04697	2	17	14
-CVKR	0.04697	2	17	40
-CYAS	0.04697	2	17	38
-CYGS	0.04697	2	17	41
-CYNV	0.04697	2	17	16

-CPRT	0.047909	3	25	65
-CRDR	0.047909	3	25	43
-CVLR	0.048688	5	41	83
-CAKL	0.049906	2	16	34
-CALG	0.049906	1	8	37
-CAMP	0.049906	2	16	20
-CAYL	0.049906	1	8	37
-CCDM	0.049906	1	8	2
-CCGL	0.049906	1	8	35
-CCKG	0.049906	1	8	13
-CCQA	0.049906	1	8	12
-CCST	0.049906	1	8	35
-CCTG	0.049906	1	8	26
-CDAL	0.049906	3	24	30
-CDGK	0.049906	1	8	19
-CDKH	0.049906	1	8	5
-CDLY	0.049906	1	8	33
-CDMS	0.049906	2	16	25
-CDNE	0.049906	1	8	16
-CDQG	0.049906	1	8	16
-CDQT	0.049906	1	8	11
-CDRE	0.049906	1	8	18
-CDRN	0.049906	1	8	19
-CDSI	0.049906	2	16	39
-CDTE	0.049906	1	8	12
-CDYM	0.049906	1	8	20
-CDYQ	0.049906	1	8	6
-CEAS	0.049906	1	8	27
-CECE	0.049906	1	8	15
-CEFG	0.049906	1	8	11
-CEGD	0.049906	2	16	3
-CEGQ	0.049906	1	8	23
-CEHE	0.049906	1	8	13
-CEHT	0.049906	1	8	22
-CEKP	0.049906	1	8	12
-CEMG	0.049906	1	8	13
-CENT	0.049906	1	8	19
-CENW	0.049906	1	8	16
-CEPV	0.049906	1	8	25
-CERA	0.049906	1	8	29
-CERE	0.049906	1	8	22
-CERP	0.049906	1	8	22
-CESR	0.049906	4	32	39
-CFAC	0.049906	1	8	25
-CFAP	0.049906	1	8	10
-CFEV	0.049906	1	8	11
-CFFR	0.049906	1	8	11
-CFGL	0.049906	1	8	28
-CFHQ	0.049906	1	8	12
-CFMT	0.049906	1	8	14
-CFND	0.049906	1	8	11
-CFRS	0.049906	1	8	44
-CFSC	0.049906	1	8	16
-CFSI	0.049906	1	8	12
-CGAF	0.049906	1	8	16
-CGAV	0.049906	1	8	55
-CGCA	0.049906	1	8	10

-CGGR	0.049906	1	8	33
-CGHC	0.049906	1	8	12
-CGKG	0.049906	1	8	15
-CGMA	0.049906	1	8	18
-CGMT	0.049906	1	8	23
-CGNN	0.049906	1	8	22
-CGPA	0.049906	2	16	34
-CGPD	0.049906	1	8	12
-CGQA	0.049906	1	8	14
-CGRN	0.049906	1	8	30
-CGRQ	0.049906	1	8	14
-CGSW	0.049906	1	8	28
-CGSY	0.049906	1	8	21
-CGTI	0.049906	1	8	33
-CGTS	0.049906	3	24	52
-CGVI	0.049906	1	8	25
-CHCD	0.049906	1	8	11
-CHDT	0.049906	1	8	30
-CHEA	0.049906	1	8	14
-CHED	0.049906	1	8	3
-CHEN	0.049906	1	8	16
-CHKG	0.049906	1	8	13
-CHKS	0.049906	1	8	31
-CHLR	0.049906	1	8	75
-CHMR	0.049906	1	8	12
-CHPT	0.049906	1	8	10
-CHRN	0.049906	1	8	9
-CHTN	0.049906	1	8	22
-CIAR	0.049906	2	16	33
-CIDT	0.049906	1	8	15
-CIEK	0.049906	2	16	26
-CIHG	0.049906	1	8	17
-CIPD	0.049906	1	8	10
-CIRC	0.049906	1	8	8
-CIST	0.049906	2	16	21
-CKCD	0.049906	1	8	18
-CKCK	0.049906	1	8	19
-CKDN	0.049906	1	8	10
-CKEC	0.049906	1	8	10
-CKEE	0.049906	1	8	5
-CKEL	0.049906	1	8	27
-CKEV	0.049906	1	8	10
-CKGL	0.049906	2	16	22
-CKHN	0.049906	1	8	22
-CKKS	0.049906	1	8	20
-CKLN	0.049906	2	16	36
-CKMD	0.049906	1	8	10
-CKNE	0.049906	1	8	20
-CKSR	0.049906	2	16	62
-CLAS	0.049906	3	24	70
-CLCE	0.049906	1	8	21
-CLCG	0.049906	1	8	22
-CLEE	0.049906	2	16	23
-CLGF	0.049906	1	8	34
-CLGY	0.049906	1	8	15
-CLHA	0.049906	1	8	51
-CLKP	0.049906	1	8	28

-CLKQ	0.049906	1	8	13
-CLLY	0.049906	1	8	44
-CLNF	0.049906	1	8	23
-CLPY	0.049906	1	8	21
-CLQD	0.049906	2	16	19
-CLYN	0.049906	1	8	24
-CMAR	0.049906	1	8	22
-CMCG	0.049906	1	8	23
-CMKI	0.049906	1	8	14
-CMLR	0.049906	1	8	60
-CMNK	0.049906	1	8	11
-CMPT	0.049906	1	8	28
-CMRC	0.049906	1	8	17
-CMSD	0.049906	1	8	14
-CMSR	0.049906	1	8	31
-CMSS	0.049906	2	16	34
-CNFG	0.049906	1	8	16
-CNFR	0.049906	1	8	20
-CNGF	0.049906	1	8	15
-CNHK	0.049906	1	8	20
-CNKC	0.049906	1	8	12
-CNKH	0.049906	1	8	9
-CNNT	0.049906	1	8	26
-CNRP	0.049906	2	16	20
-CNSC	0.049906	1	8	24
-CNSL	0.049906	3	24	63
-CNTA	0.049906	1	8	13
-CNVD	0.049906	1	8	16
-CNYE	0.049906	1	8	25
-CNYG	0.049906	1	8	10
-CPAI	0.049906	1	8	20
-CPCI	0.049906	1	8	17
-CPDI	0.049906	1	8	9
-CPDL	0.049906	1	8	35
-CPDQ	0.049906	1	8	8
-CPEK	0.049906	1	8	8
-CPFK	0.049906	1	8	17
-CPGF	0.049906	1	8	26
-CPKW	0.049906	1	8	18
-CPLE	0.049906	3	24	38
-CPNN	0.049906	1	8	18
-CPPT	0.049906	1	8	27
-CPQG	0.049906	1	8	17
-CPRI	0.049906	1	8	22
-CPRK	0.049906	2	16	34
-CPSV	0.049906	3	24	61
-CPTK	0.049906	1	8	22
-CPVR	0.049906	3	24	78
-CQDG	0.049906	1	8	8
-CQGV	0.049906	1	8	20
-CQLY	0.049906	1	8	18
-CQSD	0.049906	1	8	18
-CQSG	0.049906	1	8	16
-CQTT	0.049906	1	8	14
-CQVE	0.049906	1	8	11
-CQYL	0.049906	1	8	19
-CRAA	0.049906	1	8	22

-CRAV	0.049906	2	16	34
-CRAW	0.049906	1	8	49
-CRDM	0.049906	1	8	16
-CREH	0.049906	1	8	14
-CRGG	0.049906	1	8	18
-CRGT	0.049906	1	8	33
-CRKE	0.049906	1	8	24
-CRKG	0.049906	1	8	29
-CRKP	0.049906	1	8	38
-CRLG	0.049906	2	16	60
-CRPI	0.049906	1	8	13
-CRPY	0.049906	1	8	27
-CRQL	0.049906	1	8	36
-CRQT	0.049906	1	8	37
-CRVK	0.049906	2	16	33
-CSDE	0.049906	3	24	25
-CSGW	0.049906	1	8	28
-CSLF	0.049906	1	8	35
-CSLP	0.049906	4	32	83
-CSMV	0.049906	2	16	40
-CSPR	0.049906	2	16	73
-CSQR	0.049906	1	8	27
-CSRF	0.049906	2	16	50
-CSWQ	0.049906	1	8	15
-CSYP	0.049906	1	8	34
-CSYV	0.049906	3	24	34
-CTAW	0.049906	1	8	20
-CTFT	0.049906	1	8	27
-CTMK	0.049906	1	8	16
-CTPY	0.049906	1	8	16
-CTQG	0.049906	2	16	21
-CTRG	0.049906	2	16	39
-CTRP	0.049906	2	16	33
-CTVW	0.049906	1	8	25
-CVAE	0.049906	2	16	30
-CVEC	0.049906	1	8	8
-CVGN	0.049906	2	16	28
-CVKP	0.049906	2	16	33
-CVKS	0.049906	2	16	42
-CVND	0.049906	1	8	12
-CVQN	0.049906	1	8	16
-CWDL	0.049906	1	8	23
-CWSE	0.049906	1	8	18
-CWSH	0.049906	1	8	17
-CYAR	0.049906	1	8	30
-CYDI	0.049906	1	8	11
-CYGN	0.049906	1	8	11
-CYGT	0.049906	1	8	18
-CYHN	0.049906	1	8	15
-CYNL	0.049906	1	8	38
-CYNS	0.049906	1	8	28
-CYPL	0.049906	3	24	49
-CYTA	0.049906	1	8	22
-CYTD	0.049906	1	8	12
-CYTN	0.049906	2	16	24
-CYTP	0.049906	1	8	18
-CYTR	0.049906	2	16	52

-CPSS	0.051754	7	54	82
-CHSI	0.052076	3	23	16
-CKLT	0.052076	3	23	57
-CLGQ	0.052076	3	23	31
-CLPN	0.052076	3	23	44
-CPSM	0.052076	3	23	32
-CRPS	0.052076	3	23	65
-CSSK	0.052076	3	23	64
-CADG	0.053233	2	15	25
-CARK	0.053233	2	15	50
-CDGP	0.053233	2	15	26
-CDRM	0.053233	2	15	17
-CDVN	0.053233	2	15	22
-CGEL	0.053233	2	15	18
-CGLG	0.053233	2	15	39
-CGVV	0.053233	2	15	47
-CHEE	0.053233	2	15	13
-CKLR	0.053233	2	15	71
-CKSC	0.053233	2	15	20
-CLDV	0.053233	2	15	31
-CLHG	0.053233	2	15	49
-CLLD	0.053233	2	15	50
-CLSM	0.053233	2	15	70
-CMIG	0.053233	2	15	17
-CNAL	0.053233	2	15	22
-CNLP	0.053233	2	15	48
-CNNV	0.053233	2	15	19
-CPKR	0.053233	2	15	30
-CRDY	0.053233	2	15	27
-CRER	0.053233	2	15	46
-CSAC	0.053233	2	15	27
-CSPD	0.053233	2	15	26
-CTDL	0.053233	4	30	54
-CTEP	0.053233	2	15	31
-CTFK	0.053233	2	15	21
-CTHK	0.053233	2	15	18
-CTKR	0.053233	2	15	41
-CTNN	0.053233	2	15	40
-CVGE	0.053233	2	15	21
-CVNV	0.053233	2	15	24
-CWDS	0.053233	2	15	21
-CWSV	0.053233	2	15	53
-CVDS	0.053952	5	37	38
-CAKK	0.054443	3	22	36
-CEGS	0.054443	3	22	17
-CGTL	0.054443	3	22	69
-CLRR	0.054443	3	22	88
-CNES	0.054443	3	22	31
-CSAN	0.054443	3	22	42
-CSAV	0.054443	3	22	63
-CSYS	0.054443	3	22	59
-CTSE	0.054443	3	22	49
-CVRS	0.054443	3	22	71
-CYSV	0.054443	3	22	51
-CDML	0.055068	4	29	33
-CHES	0.055451	5	36	48
-CAAR	0.057035	1	7	25

-CAFG	0.057035	1	7	11
-CAFH	0.057035	1	7	20
-CAFV	0.057035	1	7	27
-CAKY	0.057035	1	7	20
-CANA	0.057035	1	7	21
-CANY	0.057035	1	7	9
-CAQY	0.057035	1	7	17
-CARV	0.057035	2	14	53
-CASH	0.057035	1	7	30
-CATY	0.057035	1	7	24
-CAVK	0.057035	1	7	35
-CCEV	0.057035	1	7	12
-CCSK	0.057035	1	7	19
-CCSY	0.057035	1	7	18
-CCVY	0.057035	1	7	11
-CCYS	0.057035	1	7	21
-CDDS	0.057035	2	14	35
-CDGF	0.057035	1	7	18
-CDHS	0.057035	1	7	28
-CDKA	0.057035	1	7	21
-CDKV	0.057035	1	7	12
-CDNW	0.057035	1	7	12
-CDPL	0.057035	4	28	37
-CDQR	0.057035	1	7	19
-CDQS	0.057035	2	14	12
-CDQV	0.057035	1	7	11
-CDRY	0.057035	1	7	8
-CDSW	0.057035	1	7	19
-CDTI	0.057035	1	7	31
-CDTQ	0.057035	1	7	10
-CDWL	0.057035	1	7	18
-CDYG	0.057035	1	7	15
-CEAG	0.057035	1	7	6
-CECD	0.057035	1	7	9
-CEDV	0.057035	1	7	23
-CEEP	0.057035	1	7	15
-CEKE	0.057035	1	7	7
-CEKM	0.057035	1	7	13
-CENN	0.057035	1	7	9
-CEPS	0.057035	1	7	19
-CESQ	0.057035	1	7	14
-CETR	0.057035	1	7	16
-CEVP	0.057035	2	14	46
-CEWG	0.057035	1	7	8
-CFCE	0.057035	1	7	8
-CFCV	0.057035	1	7	17
-CFDS	0.057035	1	7	12
-CFHG	0.057035	1	7	10
-CFID	0.057035	1	7	10
-CFPI	0.057035	1	7	27
-CFPV	0.057035	1	7	16
-CFRR	0.057035	1	7	44
-CFSP	0.057035	1	7	26
-CFYR	0.057035	1	7	23
-CFYV	0.057035	1	7	20
-CGAW	0.057035	1	7	28
-CGDL	0.057035	2	14	30

-CGDV	0.057035	1	7	11
-CGER	0.057035	1	7	19
-CGFT	0.057035	1	7	15
-CGGT	0.057035	2	14	24
-CGKC	0.057035	2	14	12
-CGLC	0.057035	1	7	35
-CGNM	0.057035	2	14	28
-CGPC	0.057035	1	7	14
-CGTA	0.057035	2	14	18
-CGYE	0.057035	1	7	16
-CGYL	0.057035	1	7	35
-CHAE	0.057035	1	7	12
-CHCL	0.057035	1	7	28
-CHEL	0.057035	1	7	24
-CHFG	0.057035	1	7	12
-CHGT	0.057035	2	14	22
-CHPR	0.057035	1	7	21
-CHQV	0.057035	1	7	19
-CHSD	0.057035	1	7	22
-CHTL	0.057035	2	14	36
-CHTT	0.057035	1	7	47
-CHVK	0.057035	1	7	11
-CIEP	0.057035	1	7	18
-CIHN	0.057035	1	7	6
-CIKL	0.057035	1	7	19
-CINT	0.057035	1	7	19
-CIPK	0.057035	1	7	22
-CIQA	0.057035	1	7	3
-CIQG	0.057035	2	14	19
-CISY	0.057035	1	7	14
-CITA	0.057035	1	7	27
-CITP	0.057035	1	7	23
-CIYR	0.057035	1	7	27
-CKDI	0.057035	1	7	16
-CKGV	0.057035	2	14	24
-CKIR	0.057035	1	7	23
-CKKF	0.057035	1	7	9
-CKKR	0.057035	1	7	12
-CKPE	0.057035	1	7	6
-CKPY	0.057035	1	7	14
-CKQF	0.057035	1	7	9
-CKQL	0.057035	1	7	12
-CKRF	0.057035	1	7	13
-CKRI	0.057035	1	7	16
-CKSP	0.057035	1	7	19
-CKVY	0.057035	1	7	11
-CLEF	0.057035	1	7	16
-CLEQ	0.057035	1	7	11
-CLFK	0.057035	1	7	12
-CLFN	0.057035	1	7	22
-CLKF	0.057035	1	7	26
-CLKY	0.057035	1	7	14
-CLQI	0.057035	1	7	25
-CLQS	0.057035	2	14	48
-CLQY	0.057035	1	7	24
-CLRF	0.057035	1	7	31
-CLSQ	0.057035	2	14	51

-CMCP	0.057035	1	7	16
-CMDS	0.057035	2	14	16
-CMFT	0.057035	1	7	18
-CMGA	0.057035	1	7	14
-CMLI	0.057035	1	7	22
-CMPR	0.057035	1	7	32
-CMSG	0.057035	1	7	12
-CMTT	0.057035	2	14	21
-CMVG	0.057035	1	7	9
-CNAI	0.057035	1	7	12
-CNAV	0.057035	1	7	24
-CNDG	0.057035	1	7	16
-CNEK	0.057035	1	7	21
-CNGA	0.057035	1	7	6
-CNGP	0.057035	1	7	20
-CNGT	0.057035	1	7	34
-CNHF	0.057035	1	7	14
-CNHI	0.057035	1	7	12
-CNIG	0.057035	1	7	16
-CNLE	0.057035	1	7	9
-CNLV	0.057035	2	14	63
-CNPN	0.057035	1	7	23
-CNRY	0.057035	1	7	32
-CNSQ	0.057035	1	7	23
-CNWT	0.057035	1	7	14
-CNYS	0.057035	1	7	18
-CPAF	0.057035	1	7	24
-CPCN	0.057035	1	7	18
-CPEG	0.057035	2	14	18
-CPEM	0.057035	1	7	16
-CPGG	0.057035	1	7	24
-CPGN	0.057035	1	7	17
-CPHI	0.057035	1	7	10
-CPIE	0.057035	1	7	20
-CPIP	0.057035	2	14	17
-CPIR	0.057035	2	14	36
-CPKD	0.057035	1	7	25
-CPKK	0.057035	1	7	18
-CPKN	0.057035	1	7	16
-CPNF	0.057035	1	7	13
-CPNV	0.057035	2	14	17
-CPPH	0.057035	1	7	20
-CPRN	0.057035	2	14	23
-CPRY	0.057035	1	7	50
-CPSP	0.057035	2	14	51
-CPVE	0.057035	1	7	13
-CQAC	0.057035	1	7	31
-CQEL	0.057035	1	7	8
-CQGD	0.057035	1	7	9
-CQGP	0.057035	1	7	11
-CQLL	0.057035	2	14	36
-CQMT	0.057035	1	7	10
-CQNG	0.057035	1	7	12
-CQNS	0.057035	1	7	20
-CQRM	0.057035	1	7	15
-CQRN	0.057035	1	7	23
-CQRP	0.057035	1	7	13

-CQTN	0.057035	1	7	20
-CQTS	0.057035	2	14	36
-CQVG	0.057035	1	7	14
-CQYR	0.057035	1	7	28
-CRAH	0.057035	1	7	20
-CRDI	0.057035	1	7	14
-CREM	0.057035	1	7	19
-CREQ	0.057035	1	7	11
-CRFG	0.057035	2	14	25
-CRFL	0.057035	1	7	49
-CRGD	0.057035	2	14	12
-CRGE	0.057035	1	7	22
-CRHI	0.057035	1	7	23
-CRHT	0.057035	1	7	30
-CRME	0.057035	1	7	11
-CRPG	0.057035	1	7	34
-CRQD	0.057035	1	7	13
-CRQE	0.057035	1	7	12
-CRQI	0.057035	1	7	24
-CRSG	0.057035	4	28	67
-CRTY	0.057035	1	7	39
-CRWF	0.057035	1	7	25
-CRYE	0.057035	1	7	14
-CSAD	0.057035	2	14	30
-CSAI	0.057035	2	14	30
-CSDD	0.057035	2	14	13
-CSEM	0.057035	1	7	12
-CSFH	0.057035	1	7	22
-CSFK	0.057035	1	7	21
-CSFN	0.057035	1	7	8
-CSGD	0.057035	2	14	28
-CSHD	0.057035	1	7	16
-CSHN	0.057035	1	7	22
-CSIK	0.057035	1	7	29
-CSME	0.057035	1	7	24
-CSPW	0.057035	1	7	21
-CSRT	0.057035	4	28	85
-CTEA	0.057035	1	7	3
-CTET	0.057035	2	14	16
-CTGF	0.057035	1	7	24
-CTGI	0.057035	1	7	24
-CTHN	0.057035	1	7	23
-CTHW	0.057035	1	7	26
-CTKI	0.057035	1	7	24
-CTLI	0.057035	1	7	46
-CTNK	0.057035	1	7	18
-CTYG	0.057035	1	7	19
-CVAT	0.057035	1	7	35
-CVGT	0.057035	2	14	33
-CVNK	0.057035	1	7	19
-CVQT	0.057035	1	7	18
-CVRF	0.057035	2	14	25
-CVSA	0.057035	2	14	19
-CVSN	0.057035	2	14	27
-CWAT	0.057035	1	7	17
-CWPT	0.057035	1	7	19
-CWRT	0.057035	1	7	31

-CWSR	0.057035	1	7	28
-CYCR	0.057035	1	7	28
-CYDT	0.057035	1	7	16
-CYGC	0.057035	1	7	11
-CYGI	0.057035	1	7	9
-CYKS	0.057035	2	14	40
-CYRY	0.057035	1	7	31
-CYSY	0.057035	1	7	18
-CYTI	0.057035	1	7	10
-CYYS	0.057035	1	7	20
-CDVI	0.057035	3	21	33
-CELD	0.057035	3	21	31
-CKAS	0.057035	3	21	37
-CLAI	0.057035	3	21	34
-CNKP	0.057035	3	21	27
-CPSG	0.057035	3	21	27
-CRSW	0.057035	3	21	67
-CRTR	0.057035	3	21	111
-CSNV	0.057035	3	21	45
-CTSI	0.057035	5	35	56
-CVSK	0.057035	3	21	45
-CSAL	0.058223	7	48	77
-CRRS	0.058426	6	41	105
-CYSS	0.059147	4	27	42
-CDGA	0.059887	3	20	19
-CEDQ	0.059887	3	20	11
-CERL	0.059887	3	20	28
-CKVP	0.059887	3	20	25
-CNKR	0.059887	3	20	32
-CSKT	0.059887	3	20	40
-CSRR	0.059887	3	20	96
-CTRV	0.059887	3	20	64
-CTSP	0.059887	6	40	43
-CVGK	0.059887	3	20	18
-CSLV	0.060492	5	33	72
-CLPR	0.060755	7	46	72
-CSLT	0.061422	6	39	92
-CAET	0.061422	2	13	28
-CAYT	0.061422	2	13	27
-CCPS	0.061422	2	13	23
-CDDR	0.061422	2	13	31
-CDLT	0.061422	4	26	37
-CDRG	0.061422	2	13	20
-CDTG	0.061422	2	13	34
-CDWN	0.061422	2	13	16
-CEMR	0.061422	2	13	17
-CERN	0.061422	2	13	29
-CGIE	0.061422	2	13	30
-CGQE	0.061422	2	13	13
-CGTP	0.061422	2	13	38
-CHAL	0.061422	2	13	64
-CHLV	0.061422	2	13	34
-CHSL	0.061422	2	13	63
-CKAT	0.061422	2	13	15
-CKRL	0.061422	2	13	39
-CKSM	0.061422	2	13	21
-CKST	0.061422	2	13	33

-CLGG	0.061422	2	13	40
-CLKH	0.061422	2	13	44
-CLNG	0.061422	2	13	31
-CLNL	0.061422	4	26	60
-CLSY	0.061422	2	13	25
-CLTL	0.061422	8	52	128
-CNKS	0.061422	2	13	24
-CNLG	0.061422	2	13	36
-CPDM	0.061422	2	13	17
-CPFD	0.061422	2	13	7
-CPGV	0.061422	2	13	49
-CPKS	0.061422	2	13	35
-CPNI	0.061422	2	13	18
-CPRD	0.061422	2	13	23
-CPSN	0.061422	2	13	33
-CPYT	0.061422	2	13	28
-CQGS	0.061422	2	13	20
-CQKT	0.061422	2	13	29
-CQTF	0.061422	2	13	16
-CQTL	0.061422	2	13	35
-CRNG	0.061422	2	13	47
-CRYN	0.061422	2	13	35
-CSGG	0.061422	2	13	37
-CSMN	0.061422	2	13	23
-CTCD	0.061422	2	13	14
-CTFE	0.061422	4	26	27
-CTLL	0.061422	2	13	102
-CTNR	0.061422	4	26	54
-CTTC	0.061422	2	13	39
-CTWT	0.061422	2	13	39
-CVRK	0.061422	2	13	36
-CVTR	0.061422	2	13	30
-CDSL	0.063039	3	19	58
-CEYD	0.063039	3	19	10
-CLHE	0.063039	3	19	39
-CLHS	0.063039	3	19	66
-CNDR	0.063039	3	19	25
-CPLP	0.063039	3	19	59
-CPSA	0.063039	3	19	37
-CRLN	0.063039	3	19	52
-CTTA	0.063039	3	19	21
-CVAR	0.063039	3	19	59
-CVNS	0.063039	3	19	59
-CEDS	0.063879	4	25	28
-CEVL	0.063879	4	25	58
-CPPS	0.063879	4	25	47
-CRVL	0.063879	4	25	95
-CSTG	0.063879	4	25	56
-CVSV	0.063879	4	25	69
-CLPT	0.064743	6	37	63
-CAAE	0.066541	1	6	19
-CADE	0.066541	1	6	21
-CADF	0.066541	1	6	22
-CADW	0.066541	1	6	6
-CAEF	0.066541	1	6	17
-CAEQ	0.066541	1	6	7
-CAGI	0.066541	1	6	17

-CAGR	0.066541	1	6	33
-CAHG	0.066541	1	6	15
-CAHP	0.066541	1	6	32
-CAHV	0.066541	1	6	21
-CAKR	0.066541	2	12	30
-CANL	0.066541	1	6	28
-CATP	0.066541	1	6	28
-CATR	0.066541	2	12	43
-CAVD	0.066541	1	6	29
-CAYR	0.066541	1	6	29
-CCAD	0.066541	1	6	15
-CCAM	0.066541	1	6	8
-CCAV	0.066541	1	6	29
-CCED	0.066541	2	12	8
-CCEL	0.066541	1	6	15
-CCEP	0.066541	1	6	9
-CCET	0.066541	1	6	14
-CCFF	0.066541	1	6	6
-CCGT	0.066541	1	6	26
-CCKI	0.066541	1	6	16
-CCKM	0.066541	1	6	15
-CCKV	0.066541	1	6	8
-CCLK	0.066541	1	6	6
-CCNK	0.066541	1	6	13
-CCNN	0.066541	1	6	9
-CCNV	0.066541	1	6	19
-CCPG	0.066541	1	6	13
-CCPK	0.066541	1	6	8
-CCPR	0.066541	1	6	22
-CCPV	0.066541	1	6	15
-CCQL	0.066541	1	6	21
-CCRL	0.066541	1	6	36
-CCTI	0.066541	1	6	11
-CCTL	0.066541	1	6	49
-CCTV	0.066541	1	6	33
-CDAC	0.066541	1	6	4
-CDAE	0.066541	1	6	7
-CDCR	0.066541	1	6	24
-CDFD	0.066541	1	6	12
-CDHM	0.066541	1	6	19
-CDMH	0.066541	1	6	21
-CDMR	0.066541	2	12	21
-CDMV	0.066541	1	6	18
-CDMY	0.066541	1	6	6
-CDNQ	0.066541	1	6	10
-CDPP	0.066541	1	6	17
-CDQF	0.066541	2	12	18
-CDQQ	0.066541	1	6	12
-CDRK	0.066541	2	12	30
-CECV	0.066541	2	12	19
-CEDK	0.066541	1	6	12
-CEDL	0.066541	1	6	15
-CEED	0.066541	1	6	10
-CEHR	0.066541	1	6	19
-CELG	0.066541	1	6	24
-CEPQ	0.066541	1	6	8
-CEPR	0.066541	2	12	30

-CEPT	0.066541	1	6	18
-CEQW	0.066541	1	6	10
-CESP	0.066541	1	6	24
-CEST	0.066541	4	24	36
-CEVE	0.066541	1	6	16
-CEVG	0.066541	1	6	20
-CEYW	0.066541	1	6	8
-CFAK	0.066541	1	6	14
-CFCD	0.066541	1	6	14
-CFCT	0.066541	1	6	15
-CFCY	0.066541	1	6	4
-CFDN	0.066541	1	6	16
-CFDR	0.066541	2	12	13
-CFEH	0.066541	1	6	11
-CFEP	0.066541	1	6	9
-CFKA	0.066541	1	6	9
-CFKV	0.066541	1	6	17
-CFLR	0.066541	2	12	41
-CFMG	0.066541	1	6	10
-CFNG	0.066541	1	6	19
-CFPP	0.066541	1	6	8
-CFQQ	0.066541	1	6	7
-CFRK	0.066541	1	6	12
-CFRV	0.066541	1	6	33
-CFSE	0.066541	2	12	19
-CFTA	0.066541	2	12	30
-CFVI	0.066541	1	6	6
-CGAP	0.066541	1	6	20
-CGAR	0.066541	1	6	43
-CGCG	0.066541	1	6	14
-CGCH	0.066541	1	6	10
-CGDG	0.066541	1	6	11
-CGEH	0.066541	1	6	5
-CGET	0.066541	1	6	27
-CGFR	0.066541	1	6	24
-CGGN	0.066541	1	6	14
-CGHN	0.066541	1	6	10
-CGKA	0.066541	1	6	7
-CGKD	0.066541	1	6	9
-CGKI	0.066541	1	6	20
-CGLH	0.066541	1	6	27
-CGLI	0.066541	2	12	44
-CGLP	0.066541	2	12	40
-CGMK	0.066541	1	6	17
-CGNA	0.066541	1	6	13
-CGNQ	0.066541	1	6	25
-CGPM	0.066541	1	6	30
-CGRE	0.066541	1	6	14
-CGRH	0.066541	1	6	26
-CGRM	0.066541	1	6	28
-CGRW	0.066541	1	6	29
-CGSC	0.066541	2	12	17
-CGST	0.066541	4	24	59
-CGTH	0.066541	1	6	11
-CGTR	0.066541	1	6	52
-CGTW	0.066541	1	6	24
-CHEP	0.066541	1	6	12

-CHFC	0.066541	1	6	12
-CHGY	0.066541	1	6	13
-CHID	0.066541	1	6	14
-CHLE	0.066541	1	6	22
-CHSM	0.066541	2	12	21
-CHTC	0.066541	1	6	15
-CHTD	0.066541	2	12	27
-CIAL	0.066541	2	12	43
-CIAT	0.066541	2	12	44
-CIEE	0.066541	1	6	11
-CIGN	0.066541	1	6	22
-CIIK	0.066541	1	6	9
-CIKC	0.066541	1	6	6
-CILK	0.066541	1	6	23
-CIMK	0.066541	1	6	25
-CINI	0.066541	1	6	8
-CIQE	0.066541	1	6	13
-CIRE	0.066541	1	6	14
-CISP	0.066541	1	6	14
-CISV	0.066541	2	12	36
-CITN	0.066541	1	6	17
-CIWK	0.066541	1	6	4
-CKAD	0.066541	1	6	7
-CKAG	0.066541	1	6	25
-CKEQ	0.066541	1	6	11
-CKES	0.066541	1	6	7
-CKFK	0.066541	1	6	16
-CKFP	0.066541	1	6	6
-CKGG	0.066541	1	6	18
-CKGT	0.066541	2	12	14
-CKHW	0.066541	1	6	15
-CKKA	0.066541	1	6	8
-CKKD	0.066541	1	6	7
-CKKQ	0.066541	1	6	5
-CKKV	0.066541	1	6	19
-CKLW	0.066541	1	6	16
-CKMC	0.066541	1	6	3
-CKPH	0.066541	1	6	4
-CKPW	0.066541	1	6	14
-CKQD	0.066541	2	12	14
-CKQE	0.066541	1	6	13
-CKQS	0.066541	1	6	8
-CKSW	0.066541	1	6	13
-CKVE	0.066541	1	6	18
-CKWS	0.066541	1	6	11
-CKWT	0.066541	1	6	18
-CKYK	0.066541	1	6	6
-CLDD	0.066541	1	6	20
-CLEW	0.066541	1	6	14
-CLPC	0.066541	2	12	27
-CLPI	0.066541	1	6	31
-CLQE	0.066541	1	6	4
-CLQL	0.066541	2	12	50
-CLRE	0.066541	2	12	30
-CLRH	0.066541	1	6	41
-CLTA	0.066541	2	12	51
-CLYD	0.066541	1	6	31

-CLYK	0.066541	2	12	42
-CLYP	0.066541	1	6	42
-CLYV	0.066541	1	6	32
-CMAG	0.066541	1	6	18
-CMAQ	0.066541	1	6	10
-CMDK	0.066541	1	6	10
-CMDV	0.066541	1	6	2
-CMET	0.066541	1	6	20
-CMGK	0.066541	1	6	20
-CMKL	0.066541	1	6	20
-CMKR	0.066541	1	6	13
-CMKY	0.066541	1	6	17
-CMLD	0.066541	1	6	33
-CMLK	0.066541	1	6	22
-CMNL	0.066541	2	12	32
-CMPD	0.066541	1	6	9
-CMSM	0.066541	2	12	27
-CNEE	0.066541	1	6	10
-CNGG	0.066541	2	12	17
-CNGK	0.066541	1	6	10
-CNGM	0.066541	1	6	8
-CNHD	0.066541	1	6	10
-CNHE	0.066541	2	12	18
-CNHG	0.066541	1	6	17
-CNHV	0.066541	1	6	15
-CNKD	0.066541	1	6	8
-CNND	0.066541	1	6	8
-CNNH	0.066541	1	6	8
-CNNK	0.066541	1	6	10
-CNNW	0.066541	1	6	19
-CNPF	0.066541	1	6	13
-CNQR	0.066541	1	6	14
-CNRS	0.066541	4	24	38
-CNSI	0.066541	2	12	17
-CNTL	0.066541	1	6	32
-CNVG	0.066541	2	12	16
-CNVI	0.066541	2	12	14
-CNWK	0.066541	1	6	16
-CNWS	0.066541	1	6	16
-CPAR	0.066541	2	12	39
-CPDP	0.066541	1	6	14
-CPET	0.066541	2	12	27
-CPEV	0.066541	1	6	9
-CPFE	0.066541	1	6	17
-CPFL	0.066541	1	6	35
-CPFQ	0.066541	1	6	5
-CPHA	0.066541	1	6	21
-CPHL	0.066541	1	6	30
-CPKP	0.066541	1	6	26
-CPLG	0.066541	4	24	63
-CPLW	0.066541	1	6	23
-CPMP	0.066541	1	6	36
-CPMR	0.066541	1	6	37
-CPNA	0.066541	1	6	17
-CPNP	0.066541	1	6	13
-CPPG	0.066541	1	6	19
-CPPY	0.066541	1	6	15

-CPQI	0.066541	1	6	17
-CQAR	0.066541	1	6	14
-CQCG	0.066541	1	6	8
-CQDA	0.066541	2	12	18
-CQFV	0.066541	1	6	14
-CQGA	0.066541	1	6	14
-CQGI	0.066541	1	6	9
-CQGR	0.066541	1	6	18
-CQGW	0.066541	1	6	14
-CQGY	0.066541	1	6	7
-CQHR	0.066541	1	6	21
-CQIE	0.066541	1	6	12
-CQKN	0.066541	1	6	10
-CQKR	0.066541	2	12	16
-CQKS	0.066541	1	6	11
-CQLD	0.066541	1	6	12
-CQLR	0.066541	2	12	30
-CQNE	0.066541	1	6	10
-CQNI	0.066541	1	6	11
-CQNL	0.066541	1	6	19
-CQNV	0.066541	1	6	10
-CQQL	0.066541	1	6	11
-CQQN	0.066541	1	6	13
-CQTG	0.066541	1	6	19
-CRAC	0.066541	1	6	24
-CRDE	0.066541	1	6	22
-CRDS	0.066541	2	12	34
-CREA	0.066541	1	6	16
-CREF	0.066541	1	6	21
-CRFT	0.066541	1	6	26
-CRKA	0.066541	2	12	42
-CRKF	0.066541	1	6	19
-CRKK	0.066541	1	6	17
-CRKQ	0.066541	1	6	23
-CRLR	0.066541	4	24	142
-CRLV	0.066541	2	12	95
-CRMA	0.066541	1	6	14
-CRPF	0.066541	1	6	25
-CRPN	0.066541	1	6	32
-CRQA	0.066541	1	6	15
-CRQS	0.066541	2	12	41
-CRVH	0.066541	2	12	22
-CRVY	0.066541	1	6	29
-CRWN	0.066541	1	6	19
-CRWP	0.066541	1	6	12
-CRYY	0.066541	1	6	22
-CSCK	0.066541	1	6	20
-CSFE	0.066541	1	6	8
-CSFP	0.066541	2	12	20
-CSGC	0.066541	1	6	13
-CSGN	0.066541	2	12	31
-CSHV	0.066541	1	6	34
-CSHY	0.066541	1	6	18
-CSKK	0.066541	1	6	29
-CSLW	0.066541	1	6	62
-CSNC	0.066541	1	6	27
-CSNK	0.066541	2	12	18

-CSPA	0.066541	2	12	47
-CSPC	0.066541	1	6	19
-CSQF	0.066541	1	6	13
-CSWG	0.066541	1	6	17
-CSYL	0.066541	1	6	64
-CSYT	0.066541	2	12	71
-CTAN	0.066541	2	12	30
-CTDN	0.066541	1	6	19
-CTHD	0.066541	1	6	17
-CTHV	0.066541	2	12	33
-CTNS	0.066541	2	12	39
-CTNT	0.066541	2	12	32
-CTPE	0.066541	2	12	34
-CTPG	0.066541	1	6	21
-CTPH	0.066541	1	6	17
-CTRC	0.066541	2	12	28
-CTRD	0.066541	2	12	24
-CTRF	0.066541	2	12	40
-CTYN	0.066541	1	6	15
-CVCK	0.066541	1	6	13
-CVDF	0.066541	1	6	12
-CVDW	0.066541	1	6	16
-CVFF	0.066541	1	6	11
-CVHV	0.066541	1	6	24
-CVKH	0.066541	1	6	15
-CVKK	0.066541	2	12	33
-CVKM	0.066541	1	6	17
-CVLK	0.066541	4	24	33
-CVQK	0.066541	1	6	34
-CVRQ	0.066541	2	12	43
-CVSD	0.066541	1	6	19
-CVTE	0.066541	2	12	46
-CVTW	0.066541	1	6	36
-CVYD	0.066541	1	6	15
-CVYK	0.066541	1	6	15
-CWEH	0.066541	1	6	11
-CWET	0.066541	1	6	17
-CWKG	0.066541	1	6	10
-CWNE	0.066541	1	6	9
-CWNI	0.066541	1	6	4
-CWRR	0.066541	1	6	26
-CWRS	0.066541	1	6	33
-CWSA	0.066541	2	12	28
-CWSD	0.066541	1	6	9
-CWSM	0.066541	1	6	27
-CWTA	0.066541	1	6	16
-CWTT	0.066541	1	6	37
-CYAP	0.066541	2	12	14
-CYDS	0.066541	1	6	12
-CYER	0.066541	1	6	19
-CYIG	0.066541	2	12	24
-CYKE	0.066541	1	6	3
-CYLD	0.066541	1	6	29
-CYLG	0.066541	2	12	43
-CYLK	0.066541	1	6	48
-CYMN	0.066541	1	6	14
-CYMT	0.066541	1	6	21

-CYNN	0.066541	1	6	12
-CYQN	0.066541	1	6	14
-CYSG	0.066541	2	12	39
-CDIK	0.066541	3	18	21
-CGSD	0.066541	3	18	29
-CIGL	0.066541	3	18	29
-CKTR	0.066541	3	18	47
-CLER	0.066541	3	18	39
-CLRL	0.066541	6	36	130
-CLSR	0.066541	5	30	95
-CLTS	0.066541	5	30	96
-CNSV	0.066541	3	18	62
-CRYT	0.066541	3	18	37
-CSSI	0.066541	6	36	101
-CSTR	0.066541	6	36	96
-CSVR	0.066541	6	36	68
-CTLD	0.066541	3	18	45
-CVEL	0.066541	3	18	33
-CVNN	0.066541	3	18	30
-CYPD	0.066541	3	18	32
-CSRL	0.067796	9	53	124
-CTVR	0.068164	7	41	82
-CVGL	0.068442	6	35	48
-CDRS	0.068835	5	29	64
-CDEA	0.069434	4	23	17
-CSEL	0.069434	8	46	99
-CSLN	0.069434	4	23	62
-CSVP	0.069434	4	23	63
-CYVR	0.069434	4	23	58
-CGLR	0.070455	3	17	72
-CKDR	0.070455	3	17	26
-CKTS	0.070455	3	17	50
-CLLG	0.070455	3	17	61
-CSGV	0.070455	3	17	41
-CSMD	0.070455	3	17	25
-CSNL	0.070455	3	17	61
-CTDR	0.070455	3	17	34
-CTRR	0.070455	3	17	92
-CWES	0.070455	3	17	22
-CGSS	0.071294	5	28	75
-CKSL	0.071294	5	28	58
-CAGT	0.07259	2	11	25
-CAMG	0.07259	2	11	23
-CANS	0.07259	2	11	44
-CANT	0.07259	2	11	21
-CASD	0.07259	2	11	26
-CCHE	0.07259	2	11	4
-CCNS	0.07259	2	11	22
-CCVW	0.07259	2	11	33
-CDAR	0.07259	2	11	37
-CDEY	0.07259	2	11	27
-CDFC	0.07259	2	11	24
-CDMT	0.07259	2	11	26
-CDTV	0.07259	2	11	22
-CDWV	0.07259	2	11	16
-CDYL	0.07259	2	11	15
-CELR	0.07259	6	33	63

-CERH	0.07259	2	11	19
-CESC	0.07259	2	11	19
-CESY	0.07259	2	11	21
-CFSM	0.07259	2	11	27
-CGAS	0.07259	2	11	32
-CGFK	0.07259	2	11	12
-CGGP	0.07259	2	11	18
-CGPL	0.07259	2	11	32
-CGRI	0.07259	2	11	31
-CGVP	0.07259	2	11	38
-CHGS	0.07259	2	11	21
-CHPA	0.07259	2	11	20
-CIEN	0.07259	2	11	11
-CKRV	0.07259	2	11	28
-CLCR	0.07259	2	11	43
-CLED	0.07259	2	11	16
-CLGE	0.07259	2	11	30
-CLKA	0.07259	2	11	29
-CLKI	0.07259	2	11	20
-CLNN	0.07259	2	11	31
-CMPL	0.07259	2	11	41
-CMTD	0.07259	2	11	24
-CMTG	0.07259	2	11	15
-CMTL	0.07259	2	11	45
-CMVE	0.07259	2	11	14
-CNLH	0.07259	2	11	31
-CNSM	0.07259	2	11	21
-CPDN	0.07259	2	11	22
-CPDV	0.07259	2	11	18
-CPHR	0.07259	2	11	42
-CPNL	0.07259	2	11	48
-CPTV	0.07259	2	11	44
-CPWT	0.07259	2	11	15
-CPYR	0.07259	2	11	34
-CQFR	0.07259	2	11	23
-CQSF	0.07259	2	11	19
-CRET	0.07259	2	11	45
-CRNS	0.07259	2	11	44
-CRSL	0.07259	8	44	124
-CSFA	0.07259	2	11	25
-CSTP	0.07259	4	22	71
-CTAK	0.07259	2	11	18
-CTGN	0.07259	2	11	35
-CTGV	0.07259	2	11	31
-CTQE	0.07259	2	11	9
-CVAC	0.07259	2	11	21
-CVDE	0.07259	2	11	17
-CVEP	0.07259	2	11	27
-CVGI	0.07259	2	11	20
-CVGP	0.07259	2	11	11
-CYLH	0.07259	2	11	24
-CYPV	0.07259	2	11	22
-CYRV	0.07259	2	11	33
-CYTV	0.07259	2	11	28
-CVSL	0.073934	5	27	80
-CDLL	0.074278	8	43	101
-CDRT	0.074859	3	16	34

-CDYS	0.074859	3	16	18
-CFVV	0.074859	3	16	28
-CKSH	0.074859	3	16	30
-CKTL	0.074859	3	16	17
-CLEV	0.074859	3	16	34
-CLLE	0.074859	3	16	39
-CMVR	0.074859	3	16	31
-CPES	0.074859	3	16	18
-CPLV	0.074859	3	16	66
-CPSI	0.074859	3	16	33
-CQVR	0.074859	3	16	32
-CRPA	0.074859	3	16	41
-CSPL	0.074859	3	16	57
-CSYK	0.074859	3	16	34
-CTDT	0.074859	3	16	32
-CTFG	0.074859	3	16	19
-CTKY	0.074859	3	16	11
-CVTG	0.074859	3	16	46
-CVVP	0.074859	3	16	40
-CSIR	0.075533	7	37	73
-CSLL	0.075533	7	37	150
-CALT	0.076047	4	21	73
-CIRV	0.076047	4	21	42
-CRRR	0.076047	4	21	94
-CSQK	0.076047	4	21	39
-CTTE	0.076047	4	21	47
-CAAC	0.079849	1	5	10
-CAEC	0.079849	1	5	7
-CAEE	0.079849	2	10	18
-CAEK	0.079849	1	5	1
-CAFA	0.079849	1	5	12
-CAFE	0.079849	1	5	15
-CAFP	0.079849	1	5	24
-CAGA	0.079849	1	5	16
-CAHC	0.079849	1	5	11
-CAKC	0.079849	1	5	9
-CAKI	0.079849	1	5	10
-CAMD	0.079849	1	5	10
-CAMW	0.079849	1	5	10
-CANG	0.079849	1	5	16
-CAQF	0.079849	1	5	9
-CAQR	0.079849	1	5	22
-CARC	0.079849	3	15	41
-CARR	0.079849	2	10	65
-CASK	0.079849	2	10	49
-CAVR	0.079849	4	20	53
-CAWR	0.079849	1	5	24
-CAYK	0.079849	1	5	19
-CCAG	0.079849	1	5	3
-CCAK	0.079849	2	10	12
-CCAY	0.079849	1	5	14
-CCEK	0.079849	1	5	19
-CCER	0.079849	2	10	28
-CCKL	0.079849	1	5	22
-CCKS	0.079849	1	5	25
-CCNA	0.079849	1	5	18
-CCNL	0.079849	1	5	37

-CCPN	0.079849	2	10	11
-CCQK	0.079849	1	5	22
-CCRP	0.079849	1	5	36
-CCSR	0.079849	2	10	28
-CCVG	0.079849	1	5	11
-CCYE	0.079849	1	5	3
-CCYQ	0.079849	2	10	20
-CCYR	0.079849	1	5	13
-CDAK	0.079849	1	5	11
-CDCN	0.079849	1	5	17
-CDCP	0.079849	1	5	10
-CDCT	0.079849	1	5	23
-CDDC	0.079849	1	5	6
-CDDD	0.079849	1	5	8
-CDDI	0.079849	1	5	16
-CDDK	0.079849	1	5	9
-CDEP	0.079849	1	5	6
-CDER	0.079849	3	15	20
-CDET	0.079849	1	5	17
-CDEW	0.079849	1	5	6
-CDHD	0.079849	1	5	9
-CDHK	0.079849	1	5	9
-CDHY	0.079849	1	5	15
-CDKC	0.079849	1	5	19
-CDKM	0.079849	2	10	13
-CDMA	0.079849	1	5	15
-CDMG	0.079849	1	5	16
-CDPT	0.079849	3	15	21
-CDQI	0.079849	1	5	18
-CDRQ	0.079849	1	5	13
-CDRW	0.079849	2	10	15
-CDTM	0.079849	1	5	17
-CDWE	0.079849	1	5	7
-CDWM	0.079849	1	5	11
-CDYA	0.079849	1	5	16
-CDYE	0.079849	1	5	14
-CDYT	0.079849	1	5	18
-CEAD	0.079849	1	5	11
-CEAT	0.079849	1	5	15
-CECT	0.079849	1	5	22
-CEDG	0.079849	1	5	10
-CEFY	0.079849	1	5	7
-CEGN	0.079849	1	5	10
-CEGT	0.079849	1	5	8
-CEHL	0.079849	2	10	17
-CEKN	0.079849	1	5	11
-CELI	0.079849	1	5	17
-CEMN	0.079849	1	5	11
-CEMT	0.079849	1	5	15
-CEMV	0.079849	1	5	6
-CENV	0.079849	1	5	3
-CEPA	0.079849	1	5	16
-CEPG	0.079849	1	5	7
-CEQY	0.079849	1	5	23
-CERC	0.079849	1	5	10
-CETD	0.079849	3	15	25
-CETF	0.079849	1	5	22

-CETG	0.079849	2	10	25
-CETH	0.079849	1	5	19
-CETY	0.079849	1	5	4
-CEYG	0.079849	2	10	30
-CFAE	0.079849	1	5	12
-CFAQ	0.079849	1	5	7
-CFAT	0.079849	1	5	24
-CFDA	0.079849	1	5	13
-CFEI	0.079849	1	5	4
-CFGD	0.079849	1	5	11
-CFGE	0.079849	1	5	3
-CFGM	0.079849	1	5	13
-CFHP	0.079849	1	5	13
-CFKG	0.079849	1	5	11
-CFKK	0.079849	1	5	2
-CFLT	0.079849	2	10	41
-CFNS	0.079849	1	5	17
-CFPN	0.079849	1	5	22
-CFQI	0.079849	1	5	5
-CFRL	0.079849	1	5	33
-CFRQ	0.079849	1	5	9
-CFSK	0.079849	2	10	22
-CFTH	0.079849	1	5	8
-CFTS	0.079849	4	20	38
-CFVK	0.079849	1	5	6
-CGAE	0.079849	1	5	11
-CGCS	0.079849	1	5	19
-CGDF	0.079849	1	5	8
-CGDH	0.079849	1	5	25
-CGDQ	0.079849	1	5	8
-CGEN	0.079849	1	5	17
-CGFA	0.079849	1	5	6
-CGKM	0.079849	2	10	31
-CGKT	0.079849	1	5	22
-CGMR	0.079849	2	10	42
-CGNR	0.079849	1	5	17
-CGPF	0.079849	1	5	30
-CGPG	0.079849	1	5	20
-CGQV	0.079849	1	5	11
-CGRG	0.079849	1	5	49
-CGRV	0.079849	2	10	55
-CGTT	0.079849	3	15	50
-CGYP	0.079849	1	5	20
-CGYS	0.079849	1	5	25
-CHAV	0.079849	1	5	23
-CHDK	0.079849	1	5	14
-CHDS	0.079849	1	5	29
-CHDV	0.079849	1	5	18
-CHEI	0.079849	1	5	5
-CHEV	0.079849	1	5	11
-CHGE	0.079849	1	5	6
-CHGF	0.079849	1	5	20
-CHGG	0.079849	1	5	8
-CHGI	0.079849	1	5	13
-CHKA	0.079849	1	5	24
-CHKF	0.079849	1	5	4
-CHKT	0.079849	1	5	15

-CHLG	0.079849	3	15	41
-CHLI	0.079849	1	5	23
-CHMD	0.079849	1	5	8
-CHMP	0.079849	1	5	18
-CHNV	0.079849	1	5	13
-CHPV	0.079849	1	5	19
-CHQN	0.079849	1	5	1
-CHQS	0.079849	1	5	17
-CHSN	0.079849	1	5	30
-CHTI	0.079849	1	5	16
-CHWA	0.079849	1	5	12
-CHYP	0.079849	1	5	7
-CIAE	0.079849	1	5	18
-CIAH	0.079849	1	5	15
-CIDS	0.079849	2	10	27
-CIER	0.079849	1	5	26
-CIEV	0.079849	1	5	5
-CIFH	0.079849	1	5	15
-CIGC	0.079849	1	5	6
-CIKE	0.079849	1	5	8
-CIKV	0.079849	1	5	16
-CINN	0.079849	1	5	15
-CIPI	0.079849	1	5	12
-CIQS	0.079849	1	5	19
-CIRM	0.079849	2	10	29
-CIRY	0.079849	1	5	9
-CISL	0.079849	4	20	48
-CITD	0.079849	1	5	10
-CIYE	0.079849	2	10	7
-CKAI	0.079849	1	5	18
-CKCF	0.079849	1	5	3
-CKDF	0.079849	1	5	13
-CKDG	0.079849	1	5	17
-CKDP	0.079849	1	5	12
-CKFA	0.079849	1	5	20
-CKHR	0.079849	1	5	6
-CKKN	0.079849	1	5	8
-CKKP	0.079849	1	5	17
-CKLV	0.079849	1	5	23
-CKME	0.079849	1	5	7
-CKMQ	0.079849	1	5	15
-CKMT	0.079849	1	5	17
-CKNN	0.079849	1	5	14
-CKPM	0.079849	1	5	16
-CKPP	0.079849	1	5	9
-CKQT	0.079849	1	5	16
-CKRA	0.079849	1	5	24
-CKRC	0.079849	2	10	21
-CKSA	0.079849	1	5	28
-CKSD	0.079849	1	5	25
-CKSF	0.079849	1	5	17
-CKTV	0.079849	1	5	27
-CKWG	0.079849	1	5	13
-CKWL	0.079849	1	5	20
-CKYD	0.079849	1	5	7
-CKYH	0.079849	1	5	19
-CKYR	0.079849	1	5	10

-CLAA	0.079849	1	5	33
-CLAL	0.079849	9	45	87
-CLEI	0.079849	1	5	14
-CLEK	0.079849	1	5	15
-CLGW	0.079849	1	5	33
-CLHD	0.079849	1	5	20
-CLLW	0.079849	1	5	75
-CLNH	0.079849	1	5	31
-CLPQ	0.079849	1	5	21
-CLTW	0.079849	2	10	50
-CLVE	0.079849	3	15	38
-CLVF	0.079849	1	5	23
-CLWP	0.079849	1	5	27
-CLWV	0.079849	1	5	24
-CMAK	0.079849	1	5	16
-CMAY	0.079849	1	5	11
-CMDD	0.079849	2	10	10
-CMDM	0.079849	2	10	23
-CMEL	0.079849	2	10	17
-CMFE	0.079849	1	5	12
-CMGF	0.079849	1	5	12
-CMGN	0.079849	1	5	13
-CMHN	0.079849	1	5	17
-CMHV	0.079849	1	5	20
-CMIE	0.079849	2	10	10
-CMKT	0.079849	1	5	20
-CMLF	0.079849	1	5	13
-CMLW	0.079849	1	5	21
-CMMD	0.079849	2	10	22
-CMMF	0.079849	1	5	11
-CMNM	0.079849	1	5	14
-CMQL	0.079849	1	5	20
-CMRA	0.079849	1	5	21
-CMRE	0.079849	1	5	18
-CMRK	0.079849	1	5	20
-CMSA	0.079849	2	10	19
-CMTF	0.079849	1	5	7
-CMVK	0.079849	1	5	10
-CNAC	0.079849	3	15	25
-CNAE	0.079849	1	5	10
-CNAM	0.079849	1	5	12
-CNDA	0.079849	1	5	19
-CNDF	0.079849	1	5	5
-CNDK	0.079849	2	10	13
-CNDY	0.079849	1	5	10
-CNEC	0.079849	1	5	7
-CNEI	0.079849	1	5	11
-CNEQ	0.079849	1	5	5
-CNGE	0.079849	1	5	16
-CNGR	0.079849	10	50	72
-CNGY	0.079849	2	10	29
-CNHS	0.079849	3	15	38
-CNKM	0.079849	1	5	26
-CNMV	0.079849	1	5	26
-CNNG	0.079849	1	5	29
-CNNN	0.079849	1	5	14
-CNPE	0.079849	1	5	10

-CNPG	0.079849	1	5	16
-CNPL	0.079849	2	10	35
-CNPP	0.079849	1	5	9
-CNPS	0.079849	1	5	45
-CNQY	0.079849	1	5	8
-CNRI	0.079849	1	5	17
-CNRK	0.079849	1	5	19
-CNSG	0.079849	2	10	28
-CNTK	0.079849	2	10	7
-CNYD	0.079849	1	5	9
-CNYL	0.079849	2	10	29
-CNYV	0.079849	1	5	28
-CPAA	0.079849	1	5	23
-CPAG	0.079849	2	10	23
-CPAK	0.079849	1	5	28
-CPAP	0.079849	1	5	21
-CPDC	0.079849	1	5	11
-CPDK	0.079849	1	5	9
-CPDR	0.079849	2	10	26
-CPEF	0.079849	1	5	17
-CPEH	0.079849	1	5	7
-CPFP	0.079849	1	5	24
-CPFR	0.079849	2	10	38
-CPGT	0.079849	1	5	37
-CPHG	0.079849	1	5	18
-CPHT	0.079849	1	5	30
-CPHY	0.079849	1	5	7
-CPIN	0.079849	1	5	19
-CPKC	0.079849	1	5	25
-CPMD	0.079849	2	10	15
-CPMN	0.079849	1	5	18
-CPMT	0.079849	1	5	18
-CPMY	0.079849	1	5	9
-CPPM	0.079849	3	15	17
-CPQC	0.079849	1	5	16
-CPQE	0.079849	1	5	9
-CPSK	0.079849	3	15	39
-CPST	0.079849	4	20	60
-CPTI	0.079849	1	5	15
-CPTM	0.079849	1	5	31
-CPTP	0.079849	2	10	30
-CPVW	0.079849	1	5	20
-CPYD	0.079849	1	5	20
-CQAM	0.079849	1	5	3
-CQAT	0.079849	1	5	7
-CQAV	0.079849	1	5	13
-CQCS	0.079849	1	5	16
-CQDP	0.079849	2	10	12
-CQEE	0.079849	1	5	3
-CQEY	0.079849	1	5	8
-CQIG	0.079849	1	5	6
-CQKE	0.079849	1	5	9
-CQMD	0.079849	1	5	7
-CQMN	0.079849	1	5	14
-CQMP	0.079849	2	10	10
-CQMS	0.079849	1	5	26
-CQNA	0.079849	1	5	7

-CQNR	0.079849	1	5	34
-CQQS	0.079849	1	5	12
-CQQW	0.079849	1	5	10
-CQRA	0.079849	1	5	19
-CQRQ	0.079849	1	5	23
-CQRV	0.079849	1	5	35
-CQRY	0.079849	1	5	22
-CQSI	0.079849	1	5	17
-CQSL	0.079849	2	10	41
-CQSY	0.079849	2	10	11
-CQYF	0.079849	1	5	7
-CQYS	0.079849	2	10	22
-CRAD	0.079849	3	15	44
-CREW	0.079849	1	5	12
-CRGS	0.079849	3	15	48
-CRHF	0.079849	1	5	16
-CRHV	0.079849	1	5	22
-CRHY	0.079849	1	5	19
-CRLW	0.079849	1	5	34
-CRMK	0.079849	1	5	20
-CRNH	0.079849	2	10	24
-CRNI	0.079849	1	5	20
-CRQN	0.079849	1	5	11
-CRTH	0.079849	1	5	24
-CRVE	0.079849	1	5	18
-CRVG	0.079849	2	10	33
-CRVV	0.079849	6	30	51
-CSAW	0.079849	1	5	22
-CSAY	0.079849	1	5	33
-CSDF	0.079849	1	5	24
-CSDR	0.079849	3	15	47
-CSDY	0.079849	2	10	19
-CSEK	0.079849	1	5	16
-CSEP	0.079849	2	10	27
-CSFG	0.079849	1	5	27
-CSFR	0.079849	2	10	22
-CSGT	0.079849	3	15	53
-CSHI	0.079849	1	5	28
-CSKC	0.079849	2	10	14
-CSMH	0.079849	1	5	15
-CSMP	0.079849	3	15	36
-CSNG	0.079849	4	20	40
-CSNH	0.079849	1	5	16
-CSNW	0.079849	1	5	35
-CSPG	0.079849	1	5	38
-CTAE	0.079849	1	5	27
-CTAI	0.079849	1	5	15
-CTDY	0.079849	1	5	11
-CTEK	0.079849	1	5	10
-CTFD	0.079849	2	10	11
-CTGP	0.079849	1	5	15
-CTGY	0.079849	1	5	13
-CTHA	0.079849	1	5	16
-CTKC	0.079849	1	5	5
-CTKD	0.079849	1	5	9
-CTME	0.079849	3	15	22
-CTND	0.079849	2	10	14

-CTNP	0.079849	1	5	15
-CTNQ	0.079849	1	5	16
-CTNW	0.079849	1	5	29
-CTPD	0.079849	2	10	10
-CTQS	0.079849	1	5	42
-CTTI	0.079849	3	15	38
-CTTY	0.079849	1	5	16
-CTVE	0.079849	2	10	30
-CTYH	0.079849	1	5	12
-CVAA	0.079849	1	5	20
-CVCG	0.079849	1	5	17
-CVDD	0.079849	1	5	14
-CVDM	0.079849	1	5	23
-CVDQ	0.079849	1	5	11
-CVDV	0.079849	4	20	21
-CVEN	0.079849	1	5	17
-CVEW	0.079849	1	5	17
-CVEY	0.079849	1	5	7
-CVGD	0.079849	1	5	15
-CVHG	0.079849	2	10	24
-CVHP	0.079849	1	5	32
-CVHT	0.079849	1	5	13
-CVLD	0.079849	2	10	24
-CVQA	0.079849	1	5	20
-CVQD	0.079849	1	5	18
-CVQV	0.079849	1	5	47
-CVQW	0.079849	1	5	16
-CVQY	0.079849	1	5	17
-CVRI	0.079849	1	5	33
-CVRV	0.079849	4	20	69
-CVWT	0.079849	1	5	17
-CVYN	0.079849	2	10	15
-CWDY	0.079849	1	5	8
-CWFG	0.079849	1	5	20
-CWGG	0.079849	1	5	4
-CWGN	0.079849	1	5	9
-CWGS	0.079849	1	5	18
-CWHS	0.079849	1	5	15
-CWKD	0.079849	2	10	15
-CWLD	0.079849	1	5	30
-CWLP	0.079849	1	5	25
-CWNR	0.079849	1	5	21
-CWNT	0.079849	1	5	22
-CWPE	0.079849	2	10	11
-CWQD	0.079849	1	5	15
-CWQE	0.079849	2	10	4
-CWQS	0.079849	1	5	16
-CWQT	0.079849	1	5	17
-CWRL	0.079849	1	5	36
-CWSK	0.079849	1	5	19
-CWTE	0.079849	1	5	13
-CWTH	0.079849	1	5	13
-CYAE	0.079849	2	10	15
-CYCK	0.079849	1	5	13
-CYDC	0.079849	1	5	6
-CYDR	0.079849	5	25	25
-CYEM	0.079849	1	5	9

-CYEP	0.079849	2	10	26
-CYHE	0.079849	1	5	6
-CYKI	0.079849	1	5	11
-CYKM	0.079849	1	5	14
-CYLY	0.079849	1	5	13
-CYNA	0.079849	1	5	19
-CYNR	0.079849	1	5	29
-CYPE	0.079849	1	5	6
-CYQK	0.079849	1	5	13
-CYQT	0.079849	1	5	15
-CYRT	0.079849	1	5	40
-CYSQ	0.079849	2	10	40
-CYTE	0.079849	1	5	10
-CYWS	0.079849	1	5	14
-CYYP	0.079849	1	5	22
-CLLV	0.081897	8	39	95
-CRDL	0.081897	8	39	62
-CNDM	0.082603	6	29	17
-CPAL	0.082603	6	29	61
-CLSV	0.082862	11	53	98
-CLDQ	0.083176	5	24	45
-CSSY	0.083176	5	24	45
-CTTS	0.083176	5	24	70
-CVPT	0.083176	5	24	54
-CASV	0.084052	4	19	47
-CGSA	0.084052	4	19	38
-CSEV	0.084052	4	19	35
-CSVI	0.084052	4	19	48
-CTAS	0.084052	4	19	54
-CAPL	0.085553	3	14	49
-CDNF	0.085553	3	14	16
-CECL	0.085553	3	14	26
-CESN	0.085553	6	28	29
-CFDD	0.085553	3	14	15
-CGVR	0.085553	3	14	22
-CKNA	0.085553	3	14	18
-CLQK	0.085553	3	14	20
-CLYS	0.085553	3	14	59
-CMRP	0.085553	3	14	25
-CNLT	0.085553	3	14	54
-CNPR	0.085553	3	14	27
-CPAS	0.085553	3	14	38
-CPLS	0.085553	6	28	82
-CPRP	0.085553	3	14	37
-CRRV	0.085553	6	28	101
-CRSM	0.085553	3	14	57
-CRTK	0.085553	3	14	47
-CRYS	0.085553	3	14	32
-CSFD	0.085553	3	14	24
-CSGP	0.085553	3	14	27
-CSTW	0.085553	3	14	28
-CTAT	0.085553	3	14	43
-CTLK	0.085553	3	14	47
-CYIP	0.085553	3	14	26
-CDNL	0.086793	5	23	33
-CRST	0.086793	5	23	86
-CSRS	0.086793	15	69	153

-CSSM	0.086793	5	23	69
-CAEA	0.088721	2	9	14
-CAFR	0.088721	2	9	41
-CAGS	0.088721	4	18	35
-CAQD	0.088721	2	9	11
-CATI	0.088721	2	9	33
-CAWS	0.088721	2	9	44
-CAWT	0.088721	2	9	24
-CCAN	0.088721	2	9	18
-CCDS	0.088721	2	9	12
-CDAM	0.088721	2	9	19
-CDFG	0.088721	2	9	14
-CDLK	0.088721	2	9	22
-CDSG	0.088721	4	18	48
-CDTT	0.088721	2	9	17
-CDVV	0.088721	4	18	34
-CEAP	0.088721	2	9	17
-CEIE	0.088721	2	9	9
-CEYL	0.088721	2	9	19
-CEYS	0.088721	2	9	26
-CEYT	0.088721	2	9	16
-CFLK	0.088721	2	9	24
-CFPA	0.088721	2	9	13
-CGGM	0.088721	2	9	22
-CGMG	0.088721	2	9	19
-CGNS	0.088721	2	9	20
-CGPT	0.088721	2	9	28
-CHPE	0.088721	2	9	8
-CHSR	0.088721	2	9	36
-CIDD	0.088721	2	9	17
-CISC	0.088721	2	9	8
-CKGA	0.088721	2	9	10
-CKGF	0.088721	2	9	12
-CKKM	0.088721	2	9	10
-CKMI	0.088721	2	9	14
-CKNL	0.088721	2	9	26
-CKPI	0.088721	2	9	17
-CKSI	0.088721	4	18	20
-CKSY	0.088721	2	9	23
-CLAD	0.088721	2	9	21
-CLAY	0.088721	2	9	35
-CLDC	0.088721	2	9	16
-CLNA	0.088721	2	9	29
-CLRG	0.088721	4	18	63
-CMDP	0.088721	2	9	9
-CMDR	0.088721	2	9	15
-CMGS	0.088721	2	9	16
-CMLV	0.088721	2	9	32
-CMRI	0.088721	2	9	15
-CNAG	0.088721	2	9	18
-CNAR	0.088721	2	9	36
-CNCT	0.088721	2	9	31
-CNPT	0.088721	2	9	29
-CNSE	0.088721	2	9	18
-CNTP	0.088721	4	18	26
-CNTY	0.088721	2	9	20
-CPFG	0.088721	2	9	13

-CPPR	0.088721	2	9	51
-CQSE	0.088721	2	9	19
-CRRN	0.088721	2	9	30
-CRSI	0.088721	4	18	38
-CRTC	0.088721	2	9	25
-CSCL	0.088721	2	9	76
-CSEQ	0.088721	2	9	11
-CSKE	0.088721	2	9	22
-CSLE	0.088721	4	18	77
-CSPP	0.088721	2	9	22
-CSWE	0.088721	4	18	22
-CTKE	0.088721	2	9	22
-CTKH	0.088721	2	9	23
-CTRA	0.088721	2	9	21
-CTWL	0.088721	2	9	31
-CTYL	0.088721	4	18	59
-CVEI	0.088721	2	9	20
-CVFV	0.088721	2	9	23
-CVKN	0.088721	2	9	15
-CVLW	0.088721	2	9	45
-CVQL	0.088721	2	9	28
-CVRY	0.088721	2	9	26
-CYAD	0.088721	2	9	8
-CYGQ	0.088721	2	9	12
-CYMP	0.088721	2	9	34
-CYPH	0.088721	2	9	9
-CYPI	0.088721	2	9	20
-CYPN	0.088721	2	9	31
-CYRC	0.088721	2	9	17
-CYSE	0.088721	4	18	20
-CYTG	0.088721	2	9	17
-CLKS	0.088721	6	27	63
-CLRD	0.088721	6	27	26
-CSDL	0.088721	6	27	39
-CSRA	0.088721	6	27	62
-CSRV	0.088721	6	27	90
-CSSN	0.088721	6	27	45
-CSSL	0.089627	11	49	119
-CSLR	0.08983	9	40	127
-CFSR	0.090152	7	31	69
-CLRA	0.090738	5	22	64
-CSEC	0.091394	19	83	46
-CADA	0.092134	3	13	8
-CAEW	0.092134	3	13	16
-CAKD	0.092134	3	13	31
-CDVG	0.092134	3	13	21
-CDVP	0.092134	3	13	23
-CEAK	0.092134	3	13	22
-CFQT	0.092134	3	13	16
-CHGQ	0.092134	3	13	14
-CHLA	0.092134	3	13	33
-CHVE	0.092134	3	13	16
-CIYV	0.092134	3	13	14
-CLSW	0.092134	3	13	43
-CLYL	0.092134	3	13	77
-CLYR	0.092134	3	13	60
-CNFS	0.092134	3	13	22

-CNVR	0.092134	6	26	62
-CPNG	0.092134	3	13	42
-CQDT	0.092134	3	13	22
-CQTP	0.092134	3	13	29
-CRDG	0.092134	3	13	32
-CSGR	0.092134	6	26	77
-CSHG	0.092134	3	13	37
-CTFR	0.092134	3	13	47
-CTHR	0.092134	3	13	38
-CWSL	0.092134	3	13	58
-CYDL	0.092134	3	13	27
-CSER	0.093157	7	30	50
-CASL	0.09394	8	34	74
-CDSA	0.09394	4	17	37
-CMTR	0.09394	4	17	42
-CSYI	0.09394	4	17	36
-CTAL	0.09394	4	17	48
-CVRP	0.09394	4	17	53
-CLLR	0.094558	9	38	135
-CGYR	0.095058	5	21	42
-CRTD	0.095058	5	21	35
-CSFS	0.095058	5	21	28
-CTLG	0.095058	5	21	62
-CPRG	0.095819	6	25	39
-CLSN	0.096787	8	33	34
-CTLR	0.097114	9	37	98
-CVRL	0.097593	11	45	104
-CAAA	0.099811	1	4	9
-CAAH	0.099811	1	4	14
-CAAN	0.099811	1	4	11
-CACK	0.099811	1	4	15
-CACL	0.099811	2	8	37
-CADR	0.099811	1	4	31
-CAEI	0.099811	2	8	13
-CAEM	0.099811	1	4	18
-CAES	0.099811	3	12	32
-CAGC	0.099811	1	4	17
-CAGD	0.099811	1	4	4
-CAGE	0.099811	2	8	30
-CAGG	0.099811	1	4	22
-CAGH	0.099811	1	4	13
-CAGP	0.099811	1	4	14
-CAHE	0.099811	1	4	4
-CAIK	0.099811	1	4	20
-CAKE	0.099811	1	4	8
-CAKQ	0.099811	2	8	20
-CALN	0.099811	1	4	33
-CAMN	0.099811	2	8	23
-CAMR	0.099811	3	12	39
-CANK	0.099811	1	4	24
-CAPA	0.099811	1	4	14
-CAPI	0.099811	1	4	7
-CAPW	0.099811	1	4	17
-CAQK	0.099811	2	8	18
-CARG	0.099811	4	16	33
-CARQ	0.099811	1	4	37
-CARY	0.099811	1	4	19

-CASN	0.099811	4	16	33
-CATA	0.099811	1	4	12
-CATE	0.099811	3	12	21
-CATF	0.099811	1	4	19
-CATN	0.099811	1	4	13
-CATT	0.099811	2	8	46
-CATW	0.099811	1	4	21
-CAWK	0.099811	1	4	7
-CAWW	0.099811	1	4	15
-CAYE	0.099811	1	4	13
-CAYV	0.099811	3	12	24
-CCAR	0.099811	3	12	31
-CCAT	0.099811	1	4	23
-CCCN	0.099811	1	4	5
-CCCS	0.099811	2	8	24
-CCCT	0.099811	1	4	10
-CCDD	0.099811	1	4	7
-CCDK	0.099811	1	4	10
-CCFE	0.099811	1	4	20
-CCFL	0.099811	1	4	13
-CCGC	0.099811	1	4	22
-CCGN	0.099811	1	4	20
-CCGY	0.099811	1	4	12
-CCHW	0.099811	1	4	14
-CCKE	0.099811	1	4	15
-CCKR	0.099811	1	4	16
-CCME	0.099811	1	4	9
-CCNT	0.099811	1	4	15
-CCPE	0.099811	2	8	10
-CCPF	0.099811	1	4	17
-CCPW	0.099811	1	4	6
-CCQC	0.099811	1	4	21
-CCQV	0.099811	1	4	11
-CCRT	0.099811	3	12	37
-CCRW	0.099811	1	4	19
-CCSC	0.099811	1	4	6
-CCSE	0.099811	1	4	22
-CCSG	0.099811	1	4	29
-CCSN	0.099811	1	4	15
-CCTD	0.099811	1	4	15
-CCTY	0.099811	1	4	8
-CCYK	0.099811	1	4	6
-CCYP	0.099811	1	4	12
-CDAH	0.099811	1	4	8
-CDAY	0.099811	1	4	20
-CDCH	0.099811	1	4	20
-CDCY	0.099811	1	4	6
-CDDF	0.099811	1	4	9
-CDES	0.099811	1	4	11
-CDFK	0.099811	1	4	6
-CDFS	0.099811	3	12	32
-CDHN	0.099811	2	8	10
-CDIE	0.099811	1	4	13
-CDIG	0.099811	2	8	12
-CDIR	0.099811	3	12	39
-CDKN	0.099811	1	4	12
-CDKR	0.099811	1	4	19

-CDLV	0.099811	4	16	39
-CDMF	0.099811	1	4	16
-CDNH	0.099811	1	4	13
-CDNR	0.099811	1	4	27
-CDPE	0.099811	1	4	18
-CDPG	0.099811	1	4	14
-CDPK	0.099811	2	8	9
-CDPM	0.099811	1	4	8
-CDQD	0.099811	2	8	2
-CDQK	0.099811	1	4	5
-CDQN	0.099811	1	4	19
-CDSH	0.099811	1	4	24
-CDSR	0.099811	3	12	43
-CDTD	0.099811	2	8	14
-CDTF	0.099811	1	4	15
-CDTR	0.099811	2	8	42
-CDVE	0.099811	1	4	13
-CDVR	0.099811	6	24	61
-CDWA	0.099811	1	4	8
-CDYN	0.099811	2	8	11
-CEAE	0.099811	1	4	11
-CEAW	0.099811	1	4	11
-CECR	0.099811	1	4	13
-CEEC	0.099811	1	4	2
-CEEE	0.099811	1	4	4
-CEFH	0.099811	2	8	12
-CEFT	0.099811	1	4	15
-CEGF	0.099811	1	4	10
-CEHI	0.099811	1	4	3
-CEKA	0.099811	1	4	8
-CEKH	0.099811	1	4	5
-CEKV	0.099811	1	4	8
-CEKW	0.099811	1	4	4
-CELA	0.099811	3	12	32
-CELF	0.099811	1	4	9
-CELN	0.099811	2	8	21
-CELW	0.099811	1	4	17
-CENK	0.099811	1	4	7
-CENL	0.099811	1	4	23
-CEPD	0.099811	1	4	14
-CEPP	0.099811	1	4	10
-CEPW	0.099811	1	4	15
-CEPY	0.099811	1	4	10
-CERT	0.099811	2	8	36
-CERW	0.099811	1	4	19
-CETW	0.099811	1	4	7
-CEVK	0.099811	1	4	4
-CEVN	0.099811	1	4	19
-CEVY	0.099811	1	4	14
-CEWD	0.099811	1	4	3
-CEWN	0.099811	1	4	12
-CFAL	0.099811	2	8	36
-CFAS	0.099811	1	4	15
-CFDK	0.099811	1	4	4
-CFDT	0.099811	3	12	18
-CFET	0.099811	1	4	14
-CFFE	0.099811	1	4	11

-CFFV	0.099811	1	4	22
-CFGN	0.099811	1	4	22
-CFHF	0.099811	1	4	5
-CFIG	0.099811	1	4	8
-CFIY	0.099811	1	4	11
-CFKL	0.099811	1	4	19
-CFKR	0.099811	1	4	15
-CFLL	0.099811	2	8	64
-CFLN	0.099811	1	4	33
-CFNE	0.099811	1	4	11
-CFPC	0.099811	1	4	10
-CFPK	0.099811	1	4	10
-CFPQ	0.099811	1	4	6
-CFQA	0.099811	1	4	8
-CFQG	0.099811	1	4	14
-CFQV	0.099811	1	4	9
-CFRD	0.099811	1	4	13
-CFRP	0.099811	1	4	32
-CFSL	0.099811	2	8	45
-CFTR	0.099811	1	4	26
-CFVG	0.099811	3	12	22
-CFVH	0.099811	1	4	12
-CGAN	0.099811	1	4	9
-CGCD	0.099811	1	4	9
-CGCL	0.099811	2	8	34
-CGCM	0.099811	1	4	5
-CGDA	0.099811	1	4	8
-CGDC	0.099811	1	4	12
-CGDE	0.099811	1	4	15
-CGDN	0.099811	1	4	2
-CGDS	0.099811	1	4	20
-CGDW	0.099811	1	4	19
-CGEM	0.099811	1	4	16
-CGEP	0.099811	1	4	14
-CGFP	0.099811	1	4	10
-CGGQ	0.099811	1	4	12
-CGHH	0.099811	1	4	14
-CGHQ	0.099811	1	4	12
-CGID	0.099811	1	4	9
-CGIP	0.099811	1	4	18
-CGKK	0.099811	1	4	11
-CGKY	0.099811	2	8	19
-CGLL	0.099811	8	32	71
-CGLY	0.099811	1	4	16
-CGME	0.099811	2	8	18
-CGMH	0.099811	1	4	11
-CGML	0.099811	1	4	31
-CGNC	0.099811	1	4	17
-CGNH	0.099811	1	4	12
-CGQK	0.099811	1	4	10
-CGQN	0.099811	1	4	7
-CGQT	0.099811	1	4	9
-CGQW	0.099811	1	4	5
-CGVE	0.099811	1	4	16
-CGYD	0.099811	1	4	8
-CGYH	0.099811	1	4	21
-CGYY	0.099811	1	4	12

-CHAG	0.099811	1	4	11
-CHAN	0.099811	3	12	16
-CHAY	0.099811	2	8	7
-CHCE	0.099811	1	4	8
-CHCK	0.099811	1	4	8
-CHCR	0.099811	1	4	26
-CHDI	0.099811	3	12	19
-CHEG	0.099811	1	4	12
-CHEM	0.099811	1	4	8
-CHET	0.099811	1	4	26
-CHFP	0.099811	1	4	8
-CHFV	0.099811	1	4	18
-CHGP	0.099811	2	8	18
-CHHD	0.099811	1	4	11
-CHIK	0.099811	1	4	11
-CHKL	0.099811	1	4	8
-CHKR	0.099811	1	4	18
-CHNA	0.099811	1	4	17
-CHND	0.099811	1	4	15
-CHNN	0.099811	1	4	10
-CHNR	0.099811	1	4	18
-CHQG	0.099811	1	4	8
-CHRF	0.099811	1	4	15
-CHRK	0.099811	1	4	23
-CHRP	0.099811	2	8	32
-CHRR	0.099811	2	8	35
-CHRS	0.099811	4	16	34
-CHRW	0.099811	1	4	13
-CHSC	0.099811	2	8	26
-CHSK	0.099811	2	8	13
-CHTG	0.099811	1	4	12
-CHTQ	0.099811	1	4	27
-CHTS	0.099811	3	12	49
-CHTY	0.099811	1	4	15
-CHYC	0.099811	1	4	6
-CIAG	0.099811	2	8	15
-CIDY	0.099811	1	4	9
-CIEA	0.099811	1	4	9
-CIGT	0.099811	1	4	12
-CIKA	0.099811	1	4	10
-CIKF	0.099811	1	4	7
-CIKN	0.099811	1	4	4
-CILR	0.099811	4	16	59
-CILW	0.099811	1	4	23
-CINF	0.099811	1	4	14
-CINL	0.099811	3	12	30
-CIPW	0.099811	1	4	9
-CIQK	0.099811	1	4	6
-CIQL	0.099811	1	4	21
-CIQY	0.099811	1	4	14
-CIRI	0.099811	1	4	31
-CIRW	0.099811	1	4	24
-CISA	0.099811	2	8	11
-CISW	0.099811	1	4	18
-CITI	0.099811	1	4	14
-CITK	0.099811	3	12	24
-CIYL	0.099811	1	4	40

-CKAF	0.099811	2	8	6
-CKCP	0.099811	1	4	6
-CKCQ	0.099811	1	4	1
-CKCW	0.099811	1	4	14
-CKDH	0.099811	2	8	12
-CKEG	0.099811	1	4	6
-CKEM	0.099811	1	4	2
-CKEP	0.099811	1	4	7
-CKFC	0.099811	1	4	15
-CKFG	0.099811	1	4	9
-CKFH	0.099811	1	4	8
-CKFN	0.099811	1	4	10
-CKFR	0.099811	1	4	29
-CKGC	0.099811	1	4	10
-CKHF	0.099811	1	4	12
-CKHS	0.099811	2	8	11
-CKIE	0.099811	1	4	4
-CKIG	0.099811	1	4	12
-CKMS	0.099811	1	4	18
-CKNF	0.099811	1	4	10
-CKNQ	0.099811	1	4	9
-CKNW	0.099811	1	4	9
-CKPF	0.099811	1	4	9
-CKPK	0.099811	1	4	11
-CKQC	0.099811	1	4	8
-CKQG	0.099811	1	4	8
-CKQI	0.099811	1	4	7
-CKRD	0.099811	1	4	9
-CKRE	0.099811	2	8	8
-CKRS	0.099811	4	16	59
-CKSQ	0.099811	1	4	10
-CKTC	0.099811	1	4	20
-CKTF	0.099811	1	4	14
-CKTQ	0.099811	1	4	14
-CKTW	0.099811	1	4	5
-CKVI	0.099811	1	4	17
-CLAC	0.099811	2	8	9
-CLAG	0.099811	4	16	29
-CLAH	0.099811	1	4	15
-CLCK	0.099811	1	4	16
-CLDR	0.099811	4	16	50
-CLEC	0.099811	1	4	16
-CLEG	0.099811	2	8	22
-CLFP	0.099811	1	4	36
-CLFS	0.099811	3	12	28
-CLGH	0.099811	2	8	17
-CLKE	0.099811	1	4	13
-CLKG	0.099811	4	16	31
-CLMD	0.099811	1	4	28
-CLNY	0.099811	2	8	27
-CLPF	0.099811	1	4	37
-CLPM	0.099811	2	8	33
-CLRI	0.099811	3	12	55
-CLRW	0.099811	3	12	56
-CLSH	0.099811	2	8	37
-CLTY	0.099811	2	8	39
-CLVW	0.099811	1	4	46

-CLWG	0.099811	1	4	39
-CLYH	0.099811	1	4	11
-CMDQ	0.099811	2	8	17
-CMEN	0.099811	1	4	6
-CMER	0.099811	2	8	12
-CMGI	0.099811	2	8	17
-CMHA	0.099811	1	4	6
-CMHK	0.099811	1	4	8
-CMHL	0.099811	1	4	22
-CMKA	0.099811	1	4	7
-CMKK	0.099811	1	4	6
-CMKQ	0.099811	1	4	21
-CMLH	0.099811	1	4	5
-CMMK	0.099811	1	4	11
-CMMR	0.099811	2	8	20
-CMNP	0.099811	2	8	8
-CMNS	0.099811	1	4	12
-CMPE	0.099811	2	8	13
-CMPM	0.099811	1	4	17
-CMPN	0.099811	1	4	20
-CMPQ	0.099811	1	4	15
-CMQT	0.099811	1	4	20
-CMQV	0.099811	1	4	14
-CMQW	0.099811	1	4	5
-CMRR	0.099811	1	4	31
-CMTC	0.099811	1	4	8
-CMTK	0.099811	2	8	26
-CMYD	0.099811	1	4	11
-CMYL	0.099811	1	4	24
-CNAA	0.099811	1	4	10
-CNAD	0.099811	1	4	10
-CNAF	0.099811	1	4	14
-CNAQ	0.099811	1	4	12
-CNCN	0.099811	1	4	9
-CNEP	0.099811	1	4	11
-CNFV	0.099811	1	4	19
-CNGW	0.099811	1	4	7
-CNHY	0.099811	1	4	7
-CNID	0.099811	2	8	16
-CNIR	0.099811	2	8	30
-CNKG	0.099811	1	4	17
-CNKI	0.099811	1	4	12
-CNKL	0.099811	3	12	40
-CNKV	0.099811	1	4	14
-CNMD	0.099811	1	4	11
-CNMG	0.099811	1	4	14
-CNMS	0.099811	1	4	33
-CNMT	0.099811	1	4	25
-CNNC	0.099811	1	4	9
-CNNF	0.099811	1	4	8
-CNPA	0.099811	2	8	25
-CNPD	0.099811	1	4	16
-CNPK	0.099811	1	4	6
-CNPQ	0.099811	1	4	10
-CNQG	0.099811	1	4	11
-CNQS	0.099811	3	12	24
-CNQW	0.099811	1	4	12

-CNRV	0.099811	3	12	39
-CNTW	0.099811	1	4	16
-CNVK	0.099811	2	8	15
-CNWM	0.099811	1	4	5
-CNWQ	0.099811	1	4	16
-CNYA	0.099811	1	4	15
-CNYM	0.099811	1	4	12
-CPAC	0.099811	2	8	18
-CPCE	0.099811	1	4	12
-CPCK	0.099811	1	4	20
-CPCT	0.099811	2	8	18
-CPCV	0.099811	2	8	22
-CPDF	0.099811	1	4	7
-CPDW	0.099811	1	4	12
-CPEE	0.099811	2	8	12
-CPGD	0.099811	2	8	10
-CPGM	0.099811	2	8	36
-CPHC	0.099811	1	4	8
-CPHV	0.099811	1	4	23
-CPID	0.099811	1	4	7
-CPIW	0.099811	1	4	16
-CPMW	0.099811	1	4	23
-CPNH	0.099811	2	8	26
-CPNM	0.099811	3	12	27
-CPNR	0.099811	2	8	30
-CPNT	0.099811	1	4	18
-CPNY	0.099811	1	4	13
-CPPL	0.099811	2	8	37
-CPPV	0.099811	2	8	38
-CPQS	0.099811	1	4	16
-CPSH	0.099811	1	4	27
-CPTC	0.099811	2	8	19
-CPTN	0.099811	2	8	26
-CPVH	0.099811	1	4	17
-CPVI	0.099811	1	4	21
-CPVN	0.099811	1	4	19
-CPWE	0.099811	1	4	5
-CQAH	0.099811	1	4	8
-CQAN	0.099811	1	4	5
-CQAQ	0.099811	1	4	22
-CQAY	0.099811	1	4	9
-CQCT	0.099811	1	4	8
-CQDE	0.099811	1	4	9
-CQER	0.099811	2	8	20
-CQFA	0.099811	1	4	10
-CQFS	0.099811	2	8	21
-CQFW	0.099811	1	4	7
-CQGQ	0.099811	1	4	18
-CQHL	0.099811	1	4	14
-CQHV	0.099811	1	4	8
-CQIH	0.099811	1	4	23
-CQIR	0.099811	1	4	17
-CQKD	0.099811	1	4	3
-CQKM	0.099811	1	4	16
-CQLI	0.099811	1	4	23
-CQLN	0.099811	1	4	14
-CQME	0.099811	1	4	6

-CQQI	0.099811	1	4	3
-CQQV	0.099811	1	4	16
-CQRG	0.099811	1	4	14
-CQRI	0.099811	1	4	13
-CQSH	0.099811	1	4	10
-CQST	0.099811	4	16	31
-CQVD	0.099811	1	4	20
-CQVW	0.099811	1	4	9
-CQWL	0.099811	1	4	13
-CQWS	0.099811	1	4	19
-CQWT	0.099811	1	4	9
-CRCL	0.099811	2	8	37
-CRDF	0.099811	1	4	13
-CREG	0.099811	1	4	21
-CREK	0.099811	1	4	8
-CRGC	0.099811	3	12	19
-CRGW	0.099811	1	4	21
-CRGY	0.099811	1	4	18
-CRIP	0.099811	1	4	23
-CRKD	0.099811	2	8	15
-CRLD	0.099811	1	4	25
-CRLY	0.099811	1	4	28
-CRNM	0.099811	2	8	36
-CRNT	0.099811	2	8	30
-CRPH	0.099811	1	4	23
-CRPK	0.099811	1	4	24
-CRRE	0.099811	2	8	34
-CRRF	0.099811	1	4	39
-CRRK	0.099811	3	12	50
-CRSH	0.099811	2	8	31
-CRSY	0.099811	2	8	24
-CRVW	0.099811	2	8	49
-CRWL	0.099811	1	4	28
-CSAQ	0.099811	1	4	29
-CSCY	0.099811	1	4	17
-CSFF	0.099811	1	4	13
-CSFV	0.099811	2	8	36
-CSGA	0.099811	3	12	27
-CSGF	0.099811	1	4	21
-CSGK	0.099811	2	8	21
-CSID	0.099811	2	8	30
-CSKY	0.099811	1	4	19
-CSMG	0.099811	1	4	27
-CSPI	0.099811	2	8	24
-CSQE	0.099811	1	4	17
-CSQT	0.099811	2	8	28
-CSRW	0.099811	2	8	55
-CSSE	0.099811	3	12	50
-CSTD	0.099811	3	12	27
-CSWS	0.099811	3	12	63
-CSYH	0.099811	2	8	14
-CTAA	0.099811	2	8	19
-CTAG	0.099811	2	8	35
-CTDD	0.099811	2	8	18
-CTEW	0.099811	2	8	20
-CTKM	0.099811	1	4	17
-CTKQ	0.099811	1	4	14

-CTLE	0.099811	3	12	42
-CTMY	0.099811	1	4	19
-CTNH	0.099811	1	4	16
-CTPP	0.099811	2	8	22
-CTQC	0.099811	1	4	13
-CTQL	0.099811	2	8	33
-CTRW	0.099811	1	4	44
-CTTR	0.099811	6	24	56
-CTYC	0.099811	2	8	25
-CTYF	0.099811	1	4	6
-CTYI	0.099811	2	8	31
-CVAY	0.099811	1	4	12
-CVCW	0.099811	1	4	16
-CVDI	0.099811	1	4	10
-CVDT	0.099811	3	12	35
-CVEF	0.099811	2	8	23
-CVEH	0.099811	1	4	23
-CVEQ	0.099811	1	4	18
-CVFE	0.099811	1	4	16
-CVFK	0.099811	1	4	27
-CVGY	0.099811	1	4	13
-CVHW	0.099811	1	4	22
-CVNE	0.099811	1	4	20
-CVPA	0.099811	2	8	25
-CVQP	0.099811	1	4	17
-CVRC	0.099811	1	4	20
-CVSQ	0.099811	2	8	54
-CVSW	0.099811	3	12	42
-CVVW	0.099811	1	4	26
-CVWK	0.099811	1	4	20
-CVWL	0.099811	1	4	32
-CVWR	0.099811	1	4	35
-CVWW	0.099811	1	4	14
-CVYE	0.099811	1	4	10
-CVYG	0.099811	1	4	9
-CWAP	0.099811	1	4	10
-CWAR	0.099811	1	4	11
-CWCD	0.099811	1	4	11
-CWDI	0.099811	1	4	7
-CWDT	0.099811	1	4	9
-CWEG	0.099811	1	4	4
-CWEP	0.099811	1	4	7
-CWGE	0.099811	1	4	9
-CWGY	0.099811	1	4	9
-CWKA	0.099811	1	4	7
-CWKC	0.099811	1	4	11
-CWKE	0.099811	1	4	6
-CWKH	0.099811	1	4	9
-CWKN	0.099811	1	4	9
-CWKS	0.099811	1	4	15
-CWKY	0.099811	1	4	10
-CWLE	0.099811	1	4	26
-CWLG	0.099811	1	4	23
-CWLH	0.099811	1	4	9
-CWLK	0.099811	1	4	12
-CWNA	0.099811	1	4	17
-CWNG	0.099811	1	4	10

-CWPL	0.099811	1	4	42
-CWPR	0.099811	1	4	22
-CWQA	0.099811	1	4	5
-CWQN	0.099811	1	4	7
-CWTD	0.099811	1	4	13
-CWWK	0.099811	1	4	14
-CWWY	0.099811	1	4	8
-CWYM	0.099811	2	8	8
-CYAH	0.099811	1	4	11
-CYAK	0.099811	2	8	25
-CYAN	0.099811	1	4	19
-CYAW	0.099811	1	4	16
-CYDA	0.099811	2	8	13
-CYDW	0.099811	1	4	9
-CYEF	0.099811	1	4	12
-CYEH	0.099811	3	12	19
-CYEW	0.099811	1	4	11
-CYFT	0.099811	1	4	11
-CYGG	0.099811	1	4	20
-CYGK	0.099811	1	4	5
-CYHK	0.099811	1	4	11
-CYIE	0.099811	1	4	8
-CYIH	0.099811	1	4	8
-CYIW	0.099811	1	4	15
-CYKD	0.099811	1	4	13
-CYKF	0.099811	1	4	11
-CYKQ	0.099811	1	4	9
-CYKR	0.099811	1	4	22
-CYKT	0.099811	1	4	11
-CYLW	0.099811	1	4	28
-CYME	0.099811	1	4	12
-CYNK	0.099811	1	4	16
-CYPS	0.099811	1	4	36
-CYPT	0.099811	1	4	18
-CYPY	0.099811	1	4	16
-CYRD	0.099811	1	4	19
-CYSW	0.099811	1	4	17
-CYTC	0.099811	1	4	5
-CYTK	0.099811	2	8	12
-CYWE	0.099811	1	4	3
-CYYG	0.099811	1	4	28
-CYYH	0.099811	1	4	12
-CYYT	0.099811	1	4	6
-CGNL	0.099811	5	20	30
-CIRS	0.099811	5	20	60
-CQSR	0.099811	5	20	50
-CTSR	0.10303	8	31	107
-CLSD	0.10351	7	27	35
-CDCG	0.10506	5	19	21
-CDSP	0.10506	5	19	39
-CGAL	0.10506	5	19	38
-CGSV	0.10506	5	19	30
-CIDV	0.10506	5	19	24
-CQVL	0.10506	5	19	39
-CSDG	0.10506	5	19	36
-CKLS	0.10568	9	34	71
-CCAS	0.10647	4	15	48

-CGNI	0.10647	4	15	22
-CNLR	0.10647	4	15	72
-CPGS	0.10647	4	15	44
-CPRA	0.10647	4	15	31
-CSDK	0.10647	4	15	28
-CSDM	0.10647	4	15	29
-CTEL	0.10647	4	15	43
-CTHL	0.10647	4	15	37
-CYPR	0.10647	4	15	29
-CADL	0.10889	3	11	34
-CAQV	0.10889	3	11	40
-CATK	0.10889	3	11	38
-CAVE	0.10889	3	11	18
-CCDP	0.10889	3	11	11
-CCRS	0.10889	3	11	57
-CFRT	0.10889	3	11	41
-CGTG	0.10889	3	11	30
-CLAW	0.10889	3	11	44
-CLMP	0.10889	3	11	43
-CLPD	0.10889	3	11	12
-CMSK	0.10889	3	11	36
-CPSQ	0.10889	3	11	31
-CPSW	0.10889	3	11	35
-CPVT	0.10889	3	11	37
-CRCD	0.10889	3	11	25
-CRGL	0.10889	6	22	72
-CRGN	0.10889	3	11	34
-CRLE	0.10889	3	11	56
-CRNL	0.10889	3	11	52
-CRPT	0.10889	3	11	48
-CRSD	0.10889	3	11	45
-CRTN	0.10889	6	22	58
-CSAA	0.10889	3	11	38
-CSCV	0.10889	3	11	42
-CSFL	0.10889	3	11	43
-CSQW	0.10889	3	11	9
-CSRE	0.10889	3	11	46
-CTAV	0.10889	3	11	39
-CTFL	0.10889	3	11	36
-CTKP	0.10889	3	11	32
-CTTT	0.10889	3	11	44
-CVPE	0.10889	3	11	38
-CVTK	0.10889	3	11	27
-CVYL	0.10889	3	11	38
-CWTS	0.10889	3	11	37
-CLSG	0.1109	5	18	62
-CRVP	0.1109	5	18	54
-CAST	0.11179	7	25	57
-CSMS	0.11207	16	57	68
-CASS	0.11261	11	39	84
-CAAT	0.11407	2	7	44
-CAPR	0.11407	2	7	40
-CAQE	0.11407	2	7	16
-CAQG	0.11407	2	7	9
-CARD	0.11407	2	7	17
-CARF	0.11407	2	7	29
-CCPD	0.11407	2	7	18

-CCRI	0.11407	2	7	19
-CCSL	0.11407	4	14	45
-CCVR	0.11407	4	14	50
-CDAD	0.11407	2	7	10
-CDAV	0.11407	2	7	23
-CDDA	0.11407	2	7	11
-CDGW	0.11407	2	7	6
-CDHR	0.11407	4	14	38
-CDLN	0.11407	4	14	28
-CDPN	0.11407	2	7	28
-CDPY	0.11407	2	7	10
-CDRF	0.11407	2	7	10
-CDWS	0.11407	2	7	34
-CEGG	0.11407	2	7	10
-CEGY	0.11407	2	7	7
-CENA	0.11407	2	7	6
-CFAG	0.11407	2	7	13
-CFGS	0.11407	2	7	19
-CFLG	0.11407	2	7	29
-CFPG	0.11407	2	7	26
-CFRA	0.11407	2	7	25
-CFYS	0.11407	2	7	30
-CGFF	0.11407	2	7	12
-CGFV	0.11407	2	7	36
-CGGG	0.11407	2	7	18
-CGNT	0.11407	2	7	23
-CGNW	0.11407	2	7	18
-CGPN	0.11407	2	7	23
-CGRS	0.11407	4	14	75
-CHDQ	0.11407	2	7	23
-CHPN	0.11407	2	7	19
-CHSF	0.11407	2	7	25
-CHTR	0.11407	2	7	21
-CIAV	0.11407	2	7	19
-CIFR	0.11407	2	7	23
-CIGQ	0.11407	2	7	19
-CIGV	0.11407	4	14	26
-CIPG	0.11407	2	7	15
-CIRR	0.11407	4	14	65
-CITL	0.11407	2	7	28
-CIVP	0.11407	4	14	19
-CKDL	0.11407	2	7	11
-CKDQ	0.11407	2	7	6
-CLDL	0.11407	8	28	68
-CLDT	0.11407	4	14	21
-CLFL	0.11407	2	7	52
-CLLI	0.11407	4	14	49
-CLPW	0.11407	2	7	46
-CLRC	0.11407	2	7	33
-CLVP	0.11407	4	14	45
-CMAP	0.11407	2	7	13
-CMHR	0.11407	2	7	30
-CMHT	0.11407	2	7	32
-CMPI	0.11407	2	7	13
-CMTI	0.11407	2	7	6
-CMVL	0.11407	2	7	29
-CMYE	0.11407	2	7	19

-CNAS	0.11407	4	14	35
-CNDQ	0.11407	2	7	8
-CNGH	0.11407	2	7	10
-CNKY	0.11407	2	7	15
-CNLD	0.11407	2	7	22
-CNTN	0.11407	2	7	25
-CPAN	0.11407	2	7	16
-CPIK	0.11407	2	7	18
-CPQL	0.11407	2	7	34
-CPRF	0.11407	2	7	27
-CPVD	0.11407	2	7	18
-CPYF	0.11407	2	7	3
-CQDI	0.11407	2	7	14
-CQGK	0.11407	2	7	17
-CQIP	0.11407	2	7	11
-CREP	0.11407	2	7	22
-CRGK	0.11407	2	7	32
-CRGR	0.11407	2	7	38
-CRMD	0.11407	2	7	24
-CRNN	0.11407	2	7	45
-CRWV	0.11407	2	7	31
-CSCD	0.11407	2	7	12
-CSEG	0.11407	2	7	22
-CSMA	0.11407	4	14	35
-CSRP	0.11407	4	14	64
-CSVW	0.11407	2	7	29
-CSYE	0.11407	2	7	17
-CSYG	0.11407	2	7	23
-CTDP	0.11407	4	14	22
-CTGA	0.11407	2	7	13
-CTGR	0.11407	2	7	40
-CTQV	0.11407	2	7	28
-CVKG	0.11407	2	7	24
-CVNH	0.11407	2	7	11
-CVYH	0.11407	2	7	16
-CWCK	0.11407	2	7	9
-CWDK	0.11407	2	7	8
-CWLT	0.11407	2	7	23
-CWNS	0.11407	2	7	12
-CWRV	0.11407	2	7	45
-CYDV	0.11407	2	7	18
-CYEN	0.11407	2	7	26
-CYGD	0.11407	2	7	17
-CYGF	0.11407	2	7	5
-CYQR	0.11407	2	7	24
-CYYL	0.11407	2	7	20
-CRSA	0.11407	12	42	71
-CSGS	0.11407	6	21	85
-CVLL	0.11591	9	31	128
-CDAT	0.11645	7	24	34
-CQSS	0.11645	7	24	54
-CDPR	0.11743	5	17	21
-CNRL	0.11743	5	17	64
-CRTP	0.11743	5	17	54
-CTSL	0.11743	15	51	96
-CTVG	0.11743	5	17	32
-CACP	0.11977	3	10	24

-CAIP	0.11977	3	10	13
-CATS	0.11977	3	10	78
-CDAA	0.11977	3	10	13
-CELH	0.11977	3	10	17
-CEVW	0.11977	3	10	20
-CFSG	0.11977	3	10	27
-CFSH	0.11977	3	10	19
-CFVR	0.11977	3	10	26
-CGRP	0.11977	3	10	35
-CHKV	0.11977	3	10	22
-CHRV	0.11977	3	10	35
-CIRH	0.11977	3	10	26
-CIRL	0.11977	3	10	35
-CIRP	0.11977	3	10	38
-CLHV	0.11977	3	10	37
-CLVI	0.11977	3	10	47
-CMRT	0.11977	3	10	38
-CNAY	0.11977	3	10	28
-CNMR	0.11977	3	10	31
-CNRA	0.11977	3	10	31
-CPRC	0.11977	3	10	27
-CQRS	0.11977	6	20	43
-CRAN	0.11977	3	10	28
-CRED	0.11977	3	10	17
-CRKV	0.11977	3	10	23
-CRSE	0.11977	3	10	62
-CSMY	0.11977	3	10	35
-CSWP	0.11977	3	10	32
-CTMI	0.11977	3	10	23
-CVCE	0.11977	3	10	21
-CVET	0.11977	3	10	23
-CVNP	0.11977	3	10	23
-CWEL	0.11977	3	10	27
-CWPS	0.11977	3	10	30
-CWTP	0.11977	3	10	18
-CYRK	0.11977	3	10	38
-CDLS	0.12041	19	63	79
-CDRL	0.12151	7	23	60
-CMKN	0.12151	7	23	25
-CSST	0.12284	12	39	122
-CISS	0.12284	4	13	48
-CKAA	0.12284	4	13	34
-CLGR	0.12284	8	26	61
-CMAV	0.12284	4	13	32
-CNLL	0.12284	4	13	53
-CQSM	0.12284	4	13	19
-CRMS	0.12284	4	13	37
-CRYR	0.12284	4	13	56
-CSEA	0.12284	4	13	21
-CSGQ	0.12284	4	13	29
-CSNF	0.12284	4	13	23
-CSRC	0.12284	4	13	46
-CSTC	0.12284	4	13	26
-CCSV	0.12476	5	16	36
-CELY	0.12476	5	16	13
-CGSI	0.12476	5	16	33
-CMNI	0.12476	5	16	26

-CVNL	0.12476	5	16	44
-CASM	0.12608	6	19	39
-CDRP	0.12608	6	19	49
-CKSK	0.12608	6	19	23
-CSSP	0.12608	6	19	81
-CTFV	0.12608	6	19	21
-CRVR	0.12703	7	22	80
-CVMP	0.12703	7	22	41
-CDHL	0.12879	10	31	41
-CAAD	0.13308	1	3	18
-CACE	0.13308	1	3	7
-CADC	0.13308	1	3	11
-CADQ	0.13308	1	3	10
-CAFC	0.13308	1	3	8
-CAFK	0.13308	1	3	15
-CAFL	0.13308	1	3	20
-CAFN	0.13308	2	6	18
-CAFY	0.13308	1	3	12
-CAHD	0.13308	2	6	7
-CAHF	0.13308	1	3	11
-CAHK	0.13308	1	3	7
-CAKF	0.13308	1	3	14
-CALV	0.13308	8	24	70
-CAMI	0.13308	1	3	12
-CAMT	0.13308	2	6	22
-CANC	0.13308	1	3	12
-CANF	0.13308	1	3	17
-CANQ	0.13308	1	3	17
-CAPG	0.13308	2	6	20
-CAPK	0.13308	1	3	14
-CAPP	0.13308	1	3	19
-CASW	0.13308	2	6	34
-CATC	0.13308	1	3	23
-CAVW	0.13308	1	3	17
-CAWA	0.13308	1	3	10
-CAWG	0.13308	1	3	16
-CAWI	0.13308	1	3	7
-CAWL	0.13308	1	3	22
-CAWY	0.13308	1	3	12
-CCAE	0.13308	2	6	18
-CCCP	0.13308	1	3	2
-CCDG	0.13308	1	3	15
-CCDW	0.13308	1	3	7
-CCEF	0.13308	1	3	6
-CCEH	0.13308	2	6	21
-CCEQ	0.13308	1	3	10
-CCFY	0.13308	1	3	11
-CCGF	0.13308	1	3	9
-CCGG	0.13308	1	3	12
-CCGK	0.13308	2	6	30
-CCGM	0.13308	1	3	7
-CCHH	0.13308	1	3	8
-CCHT	0.13308	2	6	11
-CCKD	0.13308	1	3	8
-CCKN	0.13308	1	3	9
-CCKP	0.13308	1	3	22
-CCLV	0.13308	2	6	34

-CCMK	0.13308	1	3	12
-CCMV	0.13308	1	3	14
-CCMW	0.13308	1	3	15
-CCNQ	0.13308	1	3	12
-CCPP	0.13308	1	3	8
-CCPT	0.13308	2	6	28
-CCPY	0.13308	1	3	12
-CCQT	0.13308	1	3	18
-CCQY	0.13308	1	3	2
-CCRE	0.13308	1	3	12
-CCRN	0.13308	1	3	22
-CCSH	0.13308	1	3	8
-CCSP	0.13308	1	3	15
-CCTK	0.13308	1	3	27
-CCTR	0.13308	1	3	26
-CCWN	0.13308	1	3	8
-CCWR	0.13308	1	3	13
-CCYD	0.13308	1	3	12
-CCYT	0.13308	1	3	5
-CDCA	0.13308	1	3	8
-CDCE	0.13308	1	3	6
-CDDG	0.13308	2	6	11
-CDDN	0.13308	1	3	11
-CDEH	0.13308	1	3	6
-CDEV	0.13308	2	6	17
-CDID	0.13308	1	3	7
-CDIH	0.13308	1	3	7
-CDKD	0.13308	1	3	9
-CDMK	0.13308	1	3	12
-CDMN	0.13308	1	3	7
-CDMQ	0.13308	1	3	15
-CDMW	0.13308	1	3	6
-CDNA	0.13308	1	3	8
-CDNC	0.13308	1	3	6
-CDNK	0.13308	2	6	17
-CDNT	0.13308	1	3	14
-CDQC	0.13308	1	3	7
-CDQL	0.13308	1	3	23
-CDQP	0.13308	1	3	12
-CDTP	0.13308	2	6	11
-CDYD	0.13308	1	3	14
-CDYK	0.13308	2	6	11
-CEAA	0.13308	2	6	7
-CEAH	0.13308	1	3	12
-CEAY	0.13308	1	3	12
-CECN	0.13308	1	3	4
-CECQ	0.13308	1	3	6
-CECS	0.13308	2	6	16
-CEDH	0.13308	2	6	18
-CEDI	0.13308	1	3	7
-CEDW	0.13308	1	3	7
-CEEA	0.13308	1	3	3
-CEEH	0.13308	1	3	6
-CEEM	0.13308	1	3	6
-CEFN	0.13308	1	3	9
-CEGA	0.13308	1	3	10
-CEGI	0.13308	1	3	14

-CEHK	0.13308	1	3	11
-CEHM	0.13308	1	3	11
-CEHV	0.13308	2	6	12
-CEMP	0.13308	1	3	12
-CEMQ	0.13308	1	3	9
-CENC	0.13308	1	3	9
-CEND	0.13308	2	6	10
-CENF	0.13308	1	3	10
-CENG	0.13308	1	3	26
-CENM	0.13308	1	3	14
-CENQ	0.13308	1	3	5
-CEQA	0.13308	1	3	11
-CEQG	0.13308	1	3	7
-CEQI	0.13308	1	3	3
-CEQS	0.13308	1	3	19
-CEQT	0.13308	1	3	7
-CERF	0.13308	1	3	12
-CESI	0.13308	2	6	12
-CETA	0.13308	2	6	11
-CETE	0.13308	1	3	14
-CETI	0.13308	1	3	4
-CEVT	0.13308	2	6	22
-CEWE	0.13308	1	3	3
-CEYA	0.13308	1	3	8
-CEYP	0.13308	1	3	24
-CEYQ	0.13308	1	3	8
-CEYY	0.13308	1	3	12
-CFAA	0.13308	1	3	28
-CFAD	0.13308	1	3	17
-CFAF	0.13308	1	3	15
-CFAH	0.13308	1	3	10
-CFAM	0.13308	2	6	17
-CFCH	0.13308	1	3	4
-CFCL	0.13308	2	6	19
-CFCS	0.13308	1	3	9
-CFDF	0.13308	1	3	6
-CFDP	0.13308	1	3	5
-CFDQ	0.13308	1	3	3
-CFFG	0.13308	1	3	12
-CFFK	0.13308	1	3	12
-CFFL	0.13308	1	3	22
-CFHC	0.13308	1	3	8
-CFHE	0.13308	1	3	9
-CFHV	0.13308	2	6	21
-CFIP	0.13308	2	6	10
-CFKD	0.13308	1	3	9
-CFKP	0.13308	1	3	6
-CFLW	0.13308	1	3	31
-CFLY	0.13308	1	3	5
-CFMD	0.13308	1	3	4
-CFMH	0.13308	1	3	6
-CFMK	0.13308	1	3	10
-CFMN	0.13308	1	3	6
-CFMP	0.13308	1	3	19
-CFNF	0.13308	1	3	4
-CFNL	0.13308	2	6	32
-CFNM	0.13308	1	3	19

-CFNN	0.13308	1	3	4
-CFNQ	0.13308	1	3	10
-CFNT	0.13308	1	3	12
-CFPM	0.13308	1	3	21
-CFQM	0.13308	1	3	5
-CFRF	0.13308	1	3	23
-CFRI	0.13308	1	3	6
-CFRM	0.13308	1	3	17
-CFRN	0.13308	2	6	35
-CFSF	0.13308	1	3	17
-CFTD	0.13308	1	3	2
-CFVW	0.13308	1	3	4
-CFWA	0.13308	1	3	11
-CFWP	0.13308	1	3	22
-CFWR	0.13308	1	3	15
-CFYE	0.13308	1	3	13
-CGAC	0.13308	1	3	15
-CGCI	0.13308	1	3	14
-CGCP	0.13308	2	6	7
-CGCV	0.13308	2	6	24
-CGDD	0.13308	1	3	7
-CGEG	0.13308	2	6	17
-CGEK	0.13308	1	3	19
-CGEQ	0.13308	1	3	5
-CGFG	0.13308	2	6	11
-CGGK	0.13308	2	6	14
-CGHY	0.13308	1	3	9
-CGIN	0.13308	2	6	7
-CGIR	0.13308	2	6	33
-CGKE	0.13308	1	3	15
-CGKQ	0.13308	1	3	6
-CGLW	0.13308	1	3	30
-CGMC	0.13308	2	6	10
-CGMP	0.13308	2	6	29
-CGMQ	0.13308	1	3	11
-CGNG	0.13308	2	6	10
-CGNY	0.13308	1	3	8
-CGPE	0.13308	2	6	18
-CGPK	0.13308	1	3	11
-CGPV	0.13308	2	6	20
-CGQH	0.13308	2	6	7
-CGRK	0.13308	2	6	21
-CGTC	0.13308	1	3	10
-CGTM	0.13308	2	6	45
-CGWA	0.13308	1	3	12
-CGWE	0.13308	1	3	9
-CGWK	0.13308	1	3	13
-CGWT	0.13308	1	3	16
-CGYF	0.13308	1	3	9
-CGYI	0.13308	1	3	10
-CGYM	0.13308	1	3	11
-CHAP	0.13308	1	3	16
-CHAW	0.13308	1	3	15
-CHDE	0.13308	1	3	8
-CHDF	0.13308	2	6	5
-CHEY	0.13308	1	3	8
-CHFA	0.13308	1	3	6

-CHFL	0.13308	2	6	18
-CHHI	0.13308	1	3	9
-CHIG	0.13308	1	3	17
-CHIR	0.13308	1	3	21
-CHKE	0.13308	1	3	8
-CHMI	0.13308	1	3	10
-CHMT	0.13308	1	3	14
-CHMV	0.13308	1	3	10
-CHNM	0.13308	1	3	9
-CHNT	0.13308	1	3	44
-CHNY	0.13308	1	3	11
-CHPG	0.13308	1	3	18
-CHQY	0.13308	2	6	11
-CHRI	0.13308	1	3	11
-CHSA	0.13308	2	6	24
-CHSV	0.13308	1	3	13
-CHSW	0.13308	1	3	12
-CHTF	0.13308	1	3	7
-CHTW	0.13308	1	3	23
-CHYK	0.13308	1	3	12
-CHYS	0.13308	1	3	23
-CHYT	0.13308	1	3	8
-CIAK	0.13308	2	6	18
-CIAY	0.13308	1	3	9
-CICG	0.13308	1	3	9
-CICH	0.13308	1	3	19
-CIDE	0.13308	1	3	11
-CIDI	0.13308	1	3	9
-CIDK	0.13308	1	3	5
-CIDM	0.13308	1	3	13
-CIDR	0.13308	4	12	16
-CIFL	0.13308	1	3	18
-CIFV	0.13308	1	3	17
-CIGF	0.13308	1	3	10
-CIGM	0.13308	1	3	16
-CIHK	0.13308	1	3	8
-CIHW	0.13308	1	3	10
-CIKD	0.13308	1	3	9
-CIKH	0.13308	1	3	9
-CIKR	0.13308	1	3	21
-CIKW	0.13308	1	3	4
-CIKY	0.13308	1	3	11
-CIMG	0.13308	1	3	6
-CINA	0.13308	1	3	17
-CINH	0.13308	1	3	8
-CINV	0.13308	1	3	18
-CINY	0.13308	1	3	6
-CIPA	0.13308	2	6	24
-CIPP	0.13308	1	3	9
-CIPV	0.13308	2	6	15
-CIQD	0.13308	2	6	10
-CIQQ	0.13308	1	3	10
-CIQT	0.13308	1	3	3
-CISQ	0.13308	2	6	22
-CIWS	0.13308	1	3	31
-CIWV	0.13308	1	3	11
-CIYD	0.13308	1	3	4

-CIYG	0.13308	1	3	19
-CIYH	0.13308	1	3	16
-CKAH	0.13308	1	3	7
-CKAK	0.13308	2	6	16
-CKAP	0.13308	1	3	19
-CKCA	0.13308	1	3	22
-CKCG	0.13308	1	3	7
-CKCH	0.13308	1	3	5
-CKED	0.13308	2	6	6
-CKEY	0.13308	1	3	6
-CKGP	0.13308	1	3	7
-CKGQ	0.13308	1	3	5
-CKGY	0.13308	1	3	13
-CKHG	0.13308	1	3	5
-CKHI	0.13308	1	3	10
-CKHK	0.13308	1	3	11
-CKIK	0.13308	1	3	18
-CKKE	0.13308	1	3	4
-CKKG	0.13308	2	6	5
-CKKH	0.13308	1	3	4
-CKLY	0.13308	1	3	25
-CKMG	0.13308	1	3	10
-CKMN	0.13308	1	3	7
-CKNC	0.13308	1	3	6
-CKND	0.13308	1	3	7
-CKNH	0.13308	1	3	6
-CKNM	0.13308	1	3	10
-CKNP	0.13308	1	3	9
-CKQA	0.13308	1	3	9
-CKQM	0.13308	1	3	11
-CKQV	0.13308	2	6	16
-CKRH	0.13308	1	3	14
-CKSE	0.13308	2	6	15
-CKTH	0.13308	1	3	11
-CKTN	0.13308	1	3	10
-CKTY	0.13308	1	3	11
-CKVK	0.13308	1	3	11
-CKWM	0.13308	1	3	8
-CKWV	0.13308	1	3	12
-CKYE	0.13308	1	3	12
-CLCH	0.13308	1	3	30
-CLCN	0.13308	1	3	17
-CLCW	0.13308	2	6	14
-CLDP	0.13308	2	6	35
-CLDW	0.13308	1	3	10
-CLGC	0.13308	1	3	12
-CLHI	0.13308	1	3	17
-CLHP	0.13308	1	3	24
-CLHR	0.13308	2	6	47
-CLHW	0.13308	1	3	22
-CLID	0.13308	1	3	12
-CLKW	0.13308	1	3	9
-CLMG	0.13308	1	3	16
-CLMK	0.13308	1	3	19
-CLQF	0.13308	1	3	17
-CLWC	0.13308	1	3	17
-CLWK	0.13308	1	3	15

-CLYF	0.13308	1	3	20
-CMAD	0.13308	2	6	9
-CMAH	0.13308	1	3	7
-CMAI	0.13308	2	6	20
-CMAN	0.13308	1	3	9
-CMCE	0.13308	1	3	4
-CMCL	0.13308	2	6	21
-CMDI	0.13308	1	3	13
-CMDN	0.13308	1	3	6
-CMED	0.13308	1	3	4
-CMEE	0.13308	2	6	11
-CMEP	0.13308	1	3	4
-CMFR	0.13308	1	3	11
-CMFV	0.13308	2	6	21
-CMGC	0.13308	1	3	5
-CMGE	0.13308	1	3	15
-CMGM	0.13308	1	3	19
-CMGP	0.13308	1	3	16
-CMGQ	0.13308	1	3	11
-CMGT	0.13308	1	3	12
-CMHD	0.13308	1	3	6
-CMHI	0.13308	1	3	12
-CMHM	0.13308	1	3	15
-CMIH	0.13308	1	3	6
-CMIW	0.13308	1	3	12
-CMKG	0.13308	1	3	17
-CMKM	0.13308	1	3	8
-CMLE	0.13308	1	3	20
-CMLG	0.13308	1	3	23
-CMMA	0.13308	1	3	12
-CMMG	0.13308	1	3	10
-CMMH	0.13308	1	3	2
-CMNT	0.13308	1	3	6
-CMPP	0.13308	1	3	19
-CMPY	0.13308	1	3	16
-CMQA	0.13308	1	3	10
-CMQR	0.13308	1	3	10
-CMRH	0.13308	1	3	11
-CMRL	0.13308	4	12	51
-CMRY	0.13308	1	3	16
-CMSI	0.13308	1	3	10
-CMSQ	0.13308	1	3	20
-CMTH	0.13308	1	3	11
-CMTN	0.13308	1	3	31
-CMTW	0.13308	1	3	20
-CMTY	0.13308	1	3	18
-CMVD	0.13308	1	3	5
-CMVF	0.13308	1	3	6
-CMVI	0.13308	1	3	22
-CMWE	0.13308	1	3	8
-CMYG	0.13308	2	6	12
-CNCG	0.13308	1	3	7
-CNCK	0.13308	1	3	13
-CNCV	0.13308	1	3	21
-CNDP	0.13308	2	6	15
-CNEW	0.13308	2	6	15
-CNFA	0.13308	1	3	7

-CNFH	0.13308	1	3	11
-CNFL	0.13308	1	3	24
-CNFT	0.13308	2	6	20
-CNGN	0.13308	1	3	15
-CNGQ	0.13308	1	3	12
-CNHN	0.13308	1	3	8
-CNHR	0.13308	2	6	40
-CNHW	0.13308	2	6	10
-CNKN	0.13308	1	3	11
-CNKW	0.13308	1	3	9
-CNLF	0.13308	1	3	16
-CNLW	0.13308	1	3	23
-CNLY	0.13308	1	3	13
-CNME	0.13308	1	3	25
-CNMN	0.13308	1	3	3
-CNMW	0.13308	1	3	8
-CNNM	0.13308	1	3	15
-CNPV	0.13308	1	3	12
-CNQA	0.13308	2	6	16
-CNQP	0.13308	1	3	12
-CNRC	0.13308	1	3	23
-CNRN	0.13308	2	6	37
-CNTH	0.13308	1	3	13
-CNTI	0.13308	1	3	14
-CNWH	0.13308	1	3	7
-CNWL	0.13308	1	3	19
-CNWP	0.13308	1	3	23
-CNWW	0.13308	1	3	8
-CNYF	0.13308	1	3	5
-CNYH	0.13308	2	6	15
-CNYK	0.13308	2	6	20
-CPAH	0.13308	1	3	9
-CPAY	0.13308	2	6	24
-CPCW	0.13308	1	3	19
-CPDA	0.13308	1	3	17
-CPDS	0.13308	1	3	24
-CPDT	0.13308	1	3	25
-CPED	0.13308	2	6	6
-CPEI	0.13308	1	3	16
-CPEP	0.13308	1	3	19
-CPEW	0.13308	1	3	6
-CPEY	0.13308	1	3	10
-CPFI	0.13308	1	3	15
-CPFN	0.13308	1	3	8
-CPHD	0.13308	1	3	5
-CPHH	0.13308	1	3	9
-CPHM	0.13308	1	3	21
-CPHN	0.13308	1	3	17
-CPHW	0.13308	1	3	15
-CPKA	0.13308	1	3	17
-CPKG	0.13308	2	6	21
-CPKH	0.13308	2	6	18
-CPMK	0.13308	1	3	6
-CPPN	0.13308	1	3	24
-CPPQ	0.13308	2	6	19
-CPQA	0.13308	1	3	9
-CPQF	0.13308	1	3	5

-CPQW	0.13308	1	3	15
-CPSY	0.13308	4	12	20
-CPTD	0.13308	4	12	25
-CPTH	0.13308	1	3	22
-CPWL	0.13308	1	3	33
-CPWP	0.13308	2	6	33
-CPYC	0.13308	1	3	26
-CPYN	0.13308	1	3	21
-CPYV	0.13308	2	6	29
-CPYY	0.13308	1	3	11
-CQAD	0.13308	1	3	3
-CQAI	0.13308	1	3	17
-CQAP	0.13308	1	3	16
-CQCA	0.13308	1	3	18
-CQDD	0.13308	1	3	8
-CQDK	0.13308	1	3	10
-CQEQ	0.13308	1	3	6
-CQEW	0.13308	1	3	7
-CQFG	0.13308	2	6	16
-CQGC	0.13308	1	3	2
-CQGE	0.13308	2	6	10
-CQGF	0.13308	1	3	3
-CQGM	0.13308	1	3	7
-CQHD	0.13308	1	3	20
-CQHT	0.13308	1	3	15
-CQID	0.13308	1	3	11
-CQKP	0.13308	1	3	4
-CQLH	0.13308	1	3	11
-CQLP	0.13308	1	3	25
-CQLW	0.13308	1	3	10
-CQMF	0.13308	1	3	18
-CQMG	0.13308	1	3	10
-CQMH	0.13308	1	3	17
-CQML	0.13308	2	6	17
-CQMV	0.13308	1	3	5
-CQMW	0.13308	1	3	9
-CQNT	0.13308	2	6	9
-CQNY	0.13308	1	3	13
-CQPA	0.13308	1	3	7
-CQPL	0.13308	1	3	17
-CQPS	0.13308	1	3	12
-CQRD	0.13308	1	3	17
-CQSW	0.13308	1	3	19
-CQTW	0.13308	1	3	20
-CQVI	0.13308	1	3	6
-CQVT	0.13308	2	6	22
-CQVY	0.13308	1	3	11
-CQWG	0.13308	1	3	15
-CQYD	0.13308	1	3	8
-CQYH	0.13308	1	3	13
-CQYP	0.13308	1	3	9
-CRAI	0.13308	1	3	30
-CRAM	0.13308	1	3	21
-CRCW	0.13308	1	3	16
-CRDA	0.13308	4	12	33
-CRDK	0.13308	1	3	16
-CREE	0.13308	1	3	8

-CRFD	0.13308	1	3	18
-CRFN	0.13308	1	3	22
-CRGQ	0.13308	2	6	18
-CRKN	0.13308	1	3	10
-CRKY	0.13308	2	6	17
-CRLF	0.13308	1	3	36
-CRMP	0.13308	1	3	31
-CRMV	0.13308	1	3	30
-CRMW	0.13308	1	3	22
-CRNC	0.13308	1	3	20
-CRNF	0.13308	1	3	17
-CRNQ	0.13308	1	3	21
-CRPC	0.13308	2	6	26
-CRQY	0.13308	1	3	16
-CRRT	0.13308	4	12	55
-CRRY	0.13308	1	3	32
-CRSV	0.13308	13	39	108
-CRWC	0.13308	1	3	20
-CRWK	0.13308	1	3	22
-CRWQ	0.13308	1	3	6
-CRYG	0.13308	2	6	34
-CSAE	0.13308	2	6	26
-CSDH	0.13308	4	12	22
-CSEF	0.13308	2	6	13
-CSEN	0.13308	2	6	17
-CSEW	0.13308	1	3	15
-CSFC	0.13308	1	3	14
-CSHC	0.13308	1	3	14
-CSHM	0.13308	1	3	10
-CSHP	0.13308	2	6	26
-CSIH	0.13308	2	6	13
-CSLI	0.13308	4	12	42
-CSND	0.13308	1	3	15
-CSPE	0.13308	1	3	30
-CSPQ	0.13308	1	3	32
-CSRY	0.13308	2	6	38
-CSSF	0.13308	4	12	35
-CSWC	0.13308	1	3	11
-CSWD	0.13308	1	3	8
-CSWF	0.13308	1	3	26
-CSWL	0.13308	1	3	51
-CSWV	0.13308	1	3	27
-CSWW	0.13308	1	3	16
-CSYW	0.13308	1	3	18
-CTDE	0.13308	1	3	5
-CTDW	0.13308	1	3	5
-CTEE	0.13308	1	3	14
-CTEQ	0.13308	1	3	9
-CTFW	0.13308	1	3	36
-CTGW	0.13308	1	3	23
-CTHG	0.13308	1	3	24
-CTIP	0.13308	4	12	37
-CTLH	0.13308	4	12	63
-CTMW	0.13308	1	3	30
-CTNA	0.13308	2	6	21
-CTNE	0.13308	2	6	18
-CTPN	0.13308	2	6	25

-CTQF	0.13308	1	3	13
-CTQK	0.13308	1	3	10
-CTQT	0.13308	2	6	35
-CTTP	0.13308	2	6	25
-CTVD	0.13308	2	6	12
-CVCD	0.13308	1	3	13
-CVCH	0.13308	1	3	10
-CVDY	0.13308	2	6	13
-CVFR	0.13308	2	6	16
-CVGF	0.13308	1	3	16
-CVHE	0.13308	1	3	17
-CVHI	0.13308	1	3	19
-CVHK	0.13308	2	6	26
-CVHR	0.13308	1	3	50
-CVIK	0.13308	1	3	23
-CVKD	0.13308	2	6	10
-CVKE	0.13308	1	3	11
-CVKI	0.13308	2	6	6
-CVNY	0.13308	1	3	11
-CVPC	0.13308	1	3	15
-CVRW	0.13308	1	3	34
-CVTY	0.13308	1	3	23
-CVWC	0.13308	1	3	7
-CVWI	0.13308	1	3	8
-CVWQ	0.13308	1	3	2
-CVWV	0.13308	1	3	21
-CWAA	0.13308	1	3	9
-CWAK	0.13308	1	3	7
-CWAL	0.13308	1	3	22
-CWAN	0.13308	1	3	7
-CWCE	0.13308	1	3	9
-CWDG	0.13308	1	3	10
-CWDH	0.13308	1	3	6
-CWDM	0.13308	1	3	4
-CWDR	0.13308	2	6	10
-CWEM	0.13308	1	3	5
-CWER	0.13308	1	3	12
-CWFN	0.13308	1	3	7
-CWFQ	0.13308	1	3	7
-CWFS	0.13308	1	3	16
-CWGC	0.13308	1	3	17
-CWGD	0.13308	1	3	3
-CWGI	0.13308	1	3	11
-CWGL	0.13308	1	3	22
-CWHN	0.13308	1	3	4
-CWID	0.13308	1	3	5
-CWIG	0.13308	1	3	16
-CWIP	0.13308	1	3	6
-CWKT	0.13308	2	6	13
-CWLR	0.13308	1	3	46
-CWNL	0.13308	1	3	13
-CWPC	0.13308	1	3	11
-CWPH	0.13308	1	3	7
-CWQC	0.13308	1	3	22
-CWQR	0.13308	1	3	16
-CWRK	0.13308	1	3	15
-CWSW	0.13308	2	6	20

-CWVD	0.13308	1	3	5
-CWVG	0.13308	1	3	23
-CWVI	0.13308	1	3	8
-CWVK	0.13308	1	3	11
-CWWR	0.13308	1	3	14
-CWYH	0.13308	1	3	5
-CWYS	0.13308	1	3	13
-CWYV	0.13308	1	3	17
-CYAF	0.13308	1	3	10
-CYAG	0.13308	2	6	15
-CYCD	0.13308	1	3	7
-CYCL	0.13308	2	6	31
-CYCN	0.13308	1	3	7
-CYCP	0.13308	1	3	25
-CYCV	0.13308	1	3	22
-CYDF	0.13308	1	3	20
-CYDG	0.13308	1	3	10
-CYDN	0.13308	1	3	9
-CYEK	0.13308	1	3	6
-CYFE	0.13308	1	3	8
-CYGE	0.13308	1	3	5
-CYGM	0.13308	1	3	26
-CYGY	0.13308	2	6	16
-CYKC	0.13308	1	3	11
-CYKG	0.13308	1	3	16
-CYKN	0.13308	1	3	17
-CYKP	0.13308	1	3	28
-CYKV	0.13308	1	3	14
-CYMK	0.13308	1	3	16
-CYMS	0.13308	2	6	34
-CYNW	0.13308	1	3	8
-CYPC	0.13308	1	3	9
-CYPF	0.13308	1	3	14
-CYPG	0.13308	2	6	18
-CYPP	0.13308	1	3	14
-CYQG	0.13308	2	6	11
-CYQP	0.13308	1	3	6
-CYQV	0.13308	1	3	7
-CYRN	0.13308	1	3	27
-CYWG	0.13308	1	3	10
-CYWM	0.13308	1	3	12
-CYWT	0.13308	1	3	29
-CAEL	0.13308	3	9	24
-CAFT	0.13308	3	9	19
-CATD	0.13308	3	9	36
-CDSY	0.13308	3	9	27
-CDTW	0.13308	3	9	18
-CDVH	0.13308	5	15	7
-CENE	0.13308	3	9	16
-CESV	0.13308	6	18	46
-CFDL	0.13308	3	9	17
-CGCR	0.13308	3	9	34
-CGQL	0.13308	3	9	35
-CGSP	0.13308	3	9	29
-CKRT	0.13308	5	15	21
-CKTK	0.13308	3	9	15
-CKVG	0.13308	3	9	12

-CLFR	0.13308	3	9	43
-CLNQ	0.13308	3	9	39
-CLRM	0.13308	6	18	74
-CLYE	0.13308	3	9	28
-CMNG	0.13308	3	9	10
-CMST	0.13308	3	9	29
-CNKA	0.13308	6	18	14
-CNLI	0.13308	3	9	27
-CNVN	0.13308	3	9	28
-CNVP	0.13308	3	9	30
-CPRM	0.13308	6	18	45
-CPSF	0.13308	3	9	31
-CRHS	0.13308	3	9	44
-CRPD	0.13308	3	9	28
-CRSQ	0.13308	3	9	33
-CRYL	0.13308	3	9	56
-CSGY	0.13308	5	15	30
-CSVE	0.13308	6	18	42
-CTDG	0.13308	6	18	29
-CTRH	0.13308	3	9	32
-CTRM	0.13308	3	9	29
-CTST	0.13308	9	27	63
-CTSY	0.13308	6	18	58
-CTWR	0.13308	3	9	45
-CVPD	0.13308	3	9	22
-CVSS	0.13308	20	60	122
-CYFR	0.13308	3	9	25
-CYKA	0.13308	3	9	22
-CYRL	0.13308	3	9	62
-CYRQ	0.13308	3	9	19
-CYSA	0.13308	3	9	19
-CSVL	0.13688	12	35	93
-CVTT	0.13767	10	29	42
-CHDR	0.13974	7	20	26
-CGAD	0.14091	6	17	9
-CRAR	0.14091	6	17	91
-CSGM	0.14091	6	17	34
-CDCS	0.14259	5	14	23
-CESS	0.14259	5	14	25
-CHGL	0.14259	5	14	43
-CLVR	0.14259	10	28	66
-CMLT	0.14259	5	14	36
-CCDL	0.14518	4	11	32
-CDTN	0.14518	4	11	36
-CEKD	0.14518	4	11	16
-CFER	0.14518	4	11	15
-CGLS	0.14518	8	22	79
-CHPS	0.14518	4	11	37
-CKDT	0.14518	4	11	10
-CLAP	0.14518	4	11	45
-CLPK	0.14518	4	11	33
-CLQT	0.14518	4	11	50
-CQES	0.14518	4	11	11
-CRDW	0.14518	4	11	26
-CSAS	0.14518	8	22	85
-CSVH	0.14518	4	11	36
-CTEC	0.14518	4	11	10

-CYVL	0.14518	4	11	56
-CLVL	0.14639	11	30	93
-CSHE	0.14709	7	19	32
-CALR	0.14787	10	27	106
-CAGL	0.14972	3	8	26
-CAPS	0.14972	3	8	52
-CAYP	0.14972	3	8	33
-CCID	0.14972	3	8	13
-CCIR	0.14972	3	8	27
-CCKT	0.14972	3	8	22
-CCSW	0.14972	3	8	20
-CDEF	0.14972	3	8	14
-CDVD	0.14972	3	8	19
-CFLP	0.14972	3	8	42
-CGDT	0.14972	3	8	16
-CGPW	0.14972	3	8	19
-CGQG	0.14972	3	8	10
-CGVQ	0.14972	3	8	17
-CHTA	0.14972	3	8	9
-CITQ	0.14972	6	16	30
-CKDS	0.14972	3	8	23
-CKLI	0.14972	3	8	30
-CKVL	0.14972	3	8	36
-CLME	0.14972	3	8	20
-CMAT	0.14972	3	8	21
-CMCA	0.14972	3	8	17
-CNNR	0.14972	3	8	23
-CPAD	0.14972	3	8	23
-CRAP	0.14972	3	8	33
-CRCR	0.14972	3	8	39
-CRFP	0.14972	3	8	38
-CRRH	0.14972	3	8	26
-CRRM	0.14972	3	8	60
-CSAG	0.14972	3	8	31
-CSEI	0.14972	6	16	16
-CSKQ	0.14972	3	8	32
-CSPK	0.14972	6	16	29
-CSRH	0.14972	3	8	40
-CSSD	0.14972	6	16	40
-CSYF	0.14972	3	8	23
-CTDQ	0.14972	3	8	21
-CTPS	0.14972	3	8	57
-CTSH	0.14972	3	8	35
-CVFP	0.14972	3	8	14
-CVKT	0.14972	3	8	25
-CVLP	0.14972	6	16	70
-CVPS	0.14972	3	8	53
-CYPA	0.14972	3	8	15
-CSSV	0.15107	14	37	95
-CTYS	0.15209	8	21	72
-CEAR	0.15356	5	13	36
-CQRE	0.15356	5	13	10
-CRPE	0.15356	5	13	34
-CSFT	0.15356	5	13	46
-CSVT	0.15356	10	26	47
-CTDH	0.15356	5	13	15
-CTRQ	0.15356	5	13	41

-CYVP	0.15356	5	13	24
-CALL	0.15526	7	18	103
-CHTV	0.15526	7	18	31
-CTSM	0.15526	7	18	62
-CADK	0.1597	2	5	23
-CAEY	0.1597	2	5	16
-CAND	0.1597	2	5	11
-CASY	0.1597	2	5	21
-CAVP	0.1597	4	10	26
-CAYI	0.1597	2	5	11
-CCCA	0.1597	2	5	10
-CCDV	0.1597	4	10	16
-CCEC	0.1597	2	5	15
-CCFR	0.1597	2	5	22
-CCKA	0.1597	2	5	11
-CCLF	0.1597	2	5	10
-CCLG	0.1597	2	5	29
-CCQS	0.1597	2	5	27
-CCRC	0.1597	2	5	16
-CCSS	0.1597	6	15	69
-CCWQ	0.1597	2	5	4
-CCYG	0.1597	2	5	14
-CDAG	0.1597	4	10	14
-CDFM	0.1597	2	5	11
-CDGH	0.1597	2	5	12
-CDGQ	0.1597	4	10	10
-CDKK	0.1597	2	5	15
-CDLA	0.1597	8	20	28
-CDPF	0.1597	4	10	15
-CDSN	0.1597	4	10	35
-CDVL	0.1597	8	20	40
-CDYV	0.1597	4	10	13
-CECG	0.1597	4	10	25
-CECI	0.1597	2	5	13
-CEEL	0.1597	2	5	10
-CEFR	0.1597	4	10	27
-CEGK	0.1597	2	5	18
-CEKG	0.1597	2	5	8
-CEPL	0.1597	2	5	27
-CEWA	0.1597	2	5	19
-CEYI	0.1597	2	5	12
-CFAV	0.1597	2	5	10
-CFAY	0.1597	2	5	9
-CFCA	0.1597	2	5	9
-CFGA	0.1597	2	5	9
-CFGK	0.1597	2	5	8
-CFMA	0.1597	2	5	11
-CFPD	0.1597	2	5	13
-CFSD	0.1597	4	10	14
-CFVL	0.1597	2	5	40
-CGAI	0.1597	4	10	34
-CGCE	0.1597	2	5	13
-CGDR	0.1597	2	5	41
-CGEV	0.1597	2	5	16
-CGGA	0.1597	2	5	24
-CGHA	0.1597	2	5	10
-CGHD	0.1597	2	5	12

-CGKH	0.1597	2	5	21
-CGQR	0.1597	2	5	25
-CGRL	0.1597	4	10	39
-CGSH	0.1597	2	5	28
-CGSQ	0.1597	2	5	31
-CGVF	0.1597	2	5	20
-CGWD	0.1597	2	5	7
-CHDP	0.1597	2	5	11
-CHEQ	0.1597	2	5	9
-CHGA	0.1597	2	5	6
-CHGC	0.1597	2	5	6
-CHNC	0.1597	2	5	13
-CHQL	0.1597	2	5	31
-CHRM	0.1597	2	5	21
-CHYG	0.1597	2	5	14
-CIFS	0.1597	2	5	31
-CISE	0.1597	2	5	20
-CITW	0.1597	2	5	18
-CIWR	0.1597	2	5	21
-CIYM	0.1597	2	5	4
-CKDM	0.1597	2	5	9
-CKGW	0.1597	2	5	4
-CKLF	0.1597	2	5	26
-CKMF	0.1597	2	5	6
-CKWP	0.1597	2	5	6
-CLCL	0.1597	2	5	65
-CLGN	0.1597	2	5	21
-CLHM	0.1597	6	15	19
-CLIP	0.1597	2	5	43
-CLKM	0.1597	2	5	17
-CLND	0.1597	2	5	20
-CLQR	0.1597	2	5	25
-CLYI	0.1597	2	5	22
-CMAW	0.1597	2	5	13
-CMEQ	0.1597	2	5	15
-CMHS	0.1597	2	5	26
-CMKP	0.1597	2	5	7
-CNAH	0.1597	2	5	14
-CNCS	0.1597	2	5	23
-CNDD	0.1597	2	5	14
-CNFE	0.1597	2	5	4
-CNKF	0.1597	2	5	10
-CNKK	0.1597	2	5	21
-CNLN	0.1597	2	5	45
-CNPH	0.1597	2	5	18
-CNRQ	0.1597	2	5	19
-CNRR	0.1597	2	5	45
-CNRW	0.1597	2	5	17
-CNYN	0.1597	2	5	5
-CPEA	0.1597	2	5	8
-CPGR	0.1597	2	5	19
-CPIY	0.1597	2	5	10
-CPKY	0.1597	2	5	17
-CPLY	0.1597	2	5	25
-CPNK	0.1597	2	5	16
-CPPA	0.1597	2	5	12
-CPPI	0.1597	2	5	20

-CPVY	0.1597	2	5	10
-CQAA	0.1597	2	5	18
-CQCV	0.1597	4	10	19
-CQEP	0.1597	2	5	18
-CQLT	0.1597	2	5	36
-CQSQ	0.1597	2	5	11
-CRAG	0.1597	4	10	46
-CRAY	0.1597	4	10	30
-CRCI	0.1597	2	5	15
-CREN	0.1597	2	5	19
-CRGA	0.1597	4	10	28
-CRHD	0.1597	2	5	20
-CRHN	0.1597	2	5	20
-CRLP	0.1597	4	10	62
-CRMN	0.1597	2	5	14
-CRMT	0.1597	2	5	22
-CRRI	0.1597	2	5	27
-CSAM	0.1597	4	10	25
-CSCE	0.1597	2	5	13
-CSDN	0.1597	2	5	19
-CSNM	0.1597	4	10	26
-CSNN	0.1597	2	5	17
-CSQC	0.1597	2	5	9
-CTHI	0.1597	2	5	11
-CTHS	0.1597	2	5	36
-CVDC	0.1597	2	5	9
-CVGH	0.1597	2	5	21
-CVIR	0.1597	8	20	46
-CVMK	0.1597	2	5	14
-CVPG	0.1597	4	10	21
-CWAS	0.1597	2	5	28
-CWCA	0.1597	4	10	12
-CWCR	0.1597	2	5	14
-CWLI	0.1597	2	5	23
-CWPD	0.1597	2	5	11
-CWQV	0.1597	2	5	16
-CWRA	0.1597	4	10	16
-CWTL	0.1597	2	5	23
-CYEA	0.1597	2	5	7
-CYEI	0.1597	2	5	9
-CYGH	0.1597	2	5	4
-CYMR	0.1597	2	5	25
-CYNI	0.1597	2	5	10
-CYRP	0.1597	2	5	34
-CYSH	0.1597	2	5	20
-CYTY	0.1597	2	5	14
-CYVW	0.1597	2	5	20
-CYVY	0.1597	2	5	19
-CLIR	0.1644	7	17	47
-CKRP	0.16635	5	12	24
-CMRD	0.16635	5	12	23
-CPEQ	0.16635	5	12	20
-CPYE	0.16635	5	12	13
-CRRP	0.16635	5	12	56
-CSML	0.16635	5	12	40
-CSRM	0.16635	5	12	58
-CTIR	0.16635	5	12	43

-CYLV	0.16635	5	12	61
-CACT	0.17111	3	7	24
-CALF	0.17111	3	7	34
-CASF	0.17111	3	7	44
-CDPI	0.17111	3	7	17
-CDSM	0.17111	6	14	19
-CEKL	0.17111	3	7	21
-CEML	0.17111	3	7	29
-CEPN	0.17111	3	7	32
-CFEK	0.17111	3	7	3
-CFRY	0.17111	3	7	34
-CFTI	0.17111	3	7	8
-CGCT	0.17111	3	7	18
-CHGR	0.17111	3	7	19
-CHPP	0.17111	3	7	16
-CIDP	0.17111	3	7	10
-CKVH	0.17111	3	7	15
-CLMR	0.17111	3	7	57
-CMLP	0.17111	3	7	18
-CMVH	0.17111	3	7	16
-CNER	0.17111	3	7	19
-CNNL	0.17111	3	7	27
-CPHS	0.17111	3	7	24
-CQGN	0.17111	3	7	11
-CQRF	0.17111	3	7	14
-CQTD	0.17111	3	7	14
-CRAT	0.17111	3	7	51
-CRML	0.17111	3	7	52
-CRRL	0.17111	6	14	92
-CRYP	0.17111	3	7	42
-CSYC	0.17111	3	7	13
-CTAY	0.17111	3	7	10
-CTFP	0.17111	3	7	32
-CVHF	0.17111	3	7	16
-CVTL	0.17111	12	28	65
-CYAY	0.17111	3	7	19
-CYVE	0.17111	3	7	19
-CSDA	0.17467	7	16	23
-CYRS	0.17467	7	16	53
-CAIE	0.17744	4	9	20
-CASI	0.17744	4	9	39
-CDLP	0.17744	8	18	41
-CETP	0.17744	4	9	11
-CIPS	0.17744	4	9	17
-CLQC	0.17744	4	9	28
-CLRP	0.17744	4	9	72
-CPLA	0.17744	4	9	44
-CPLI	0.17744	4	9	35
-CQCP	0.17744	4	9	15
-CRDV	0.17744	4	9	29
-CRFR	0.17744	4	9	50
-CRHQ	0.17744	4	9	17
-CRNV	0.17744	4	9	42
-CRQV	0.17744	4	9	25
-CRRC	0.17744	4	9	26
-CVHL	0.17744	4	9	50
-CYSC	0.17744	4	9	10

-CTSV	0.17744	12	27	88
-CRSS	0.17839	21	47	118
-CARN	0.18148	5	11	32
-CDGY	0.18148	5	11	12
-CEDT	0.18148	5	11	16
-CHNL	0.18148	5	11	29
-CPAM	0.18148	5	11	17
-CQRR	0.18148	5	11	41
-CQVP	0.18148	5	11	21
-CSNT	0.18148	5	11	51
-CVLE	0.18148	5	11	37
-CGVA	0.18427	6	13	30
-CSDI	0.18427	6	13	28
-CTSA	0.18427	6	13	43
-CVES	0.18427	6	13	38
-CVGR	0.18427	6	13	62
-CTDA	0.18631	7	15	43
-CALK	0.18912	9	19	61
-CLDA	0.18912	9	19	21
-CLLL	0.19012	10	21	144
-CLSC	0.19012	10	21	31
-CSHL	0.19012	10	21	71
-CVVR	0.19012	10	21	33
-CLSS	0.19423	18	37	92
-CLLT	0.19475	20	41	108
-CSMR	0.19962	9	18	83
-CAAF	0.19962	1	2	10
-CAAI	0.19962	2	4	22
-CAAP	0.19962	1	2	22
-CAAW	0.19962	1	2	16
-CAAY	0.19962	1	2	17
-CACD	0.19962	1	2	10
-CACN	0.19962	1	2	9
-CADD	0.19962	2	4	23
-CADI	0.19962	1	2	15
-CAER	0.19962	2	4	30
-CAEV	0.19962	2	4	16
-CAFQ	0.19962	2	4	11
-CAFS	0.19962	2	4	25
-CAGK	0.19962	2	4	13
-CAGN	0.19962	1	2	11
-CAGY	0.19962	1	2	12
-CAHA	0.19962	1	2	13
-CAHM	0.19962	1	2	12
-CAHR	0.19962	2	4	31
-CAHT	0.19962	3	6	29
-CAHY	0.19962	1	2	12
-CAKH	0.19962	1	2	10
-CAKW	0.19962	1	2	16
-CAMK	0.19962	1	2	14
-CAMY	0.19962	1	2	4
-CANM	0.19962	2	4	19
-CANW	0.19962	1	2	22
-CAPF	0.19962	1	2	13
-CAPN	0.19962	2	4	21
-CAPV	0.19962	1	2	24
-CAPY	0.19962	1	2	15

-CAQS	0.19962	1	2	22
-CAQW	0.19962	1	2	15
-CARH	0.19962	2	4	10
-CATQ	0.19962	1	2	21
-CAVY	0.19962	1	2	11
-CAWD	0.19962	1	2	17
-CAWE	0.19962	1	2	8
-CAWP	0.19962	1	2	12
-CAYA	0.19962	2	4	12
-CAYD	0.19962	2	4	17
-CCAL	0.19962	4	8	35
-CCAP	0.19962	1	2	10
-CCCL	0.19962	1	2	17
-CCCV	0.19962	2	4	10
-CCEI	0.19962	2	4	9
-CCEW	0.19962	1	2	9
-CCEY	0.19962	1	2	7
-CCFA	0.19962	2	4	6
-CCFC	0.19962	3	6	14
-CCFP	0.19962	1	2	7
-CCFS	0.19962	2	4	19
-CCGA	0.19962	3	6	10
-CCGD	0.19962	1	2	1
-CCGS	0.19962	3	6	38
-CCGV	0.19962	4	8	19
-CCHA	0.19962	2	4	7
-CCHF	0.19962	1	2	8
-CCHK	0.19962	2	4	15
-CCHN	0.19962	1	2	17
-CCHY	0.19962	1	2	9
-CCIP	0.19962	1	2	7
-CCKF	0.19962	3	6	12
-CCKH	0.19962	1	2	9
-CCKQ	0.19962	1	2	16
-CCLI	0.19962	2	4	14
-CCLT	0.19962	4	8	37
-CCMD	0.19962	1	2	2
-CCMY	0.19962	1	2	11
-CCND	0.19962	1	2	3
-CCNE	0.19962	1	2	5
-CCNH	0.19962	1	2	5
-CCPI	0.19962	1	2	13
-CCQD	0.19962	1	2	6
-CCQP	0.19962	1	2	7
-CCRG	0.19962	1	2	12
-CCRH	0.19962	1	2	15
-CCRR	0.19962	2	4	40
-CCSA	0.19962	2	4	24
-CCTC	0.19962	1	2	10
-CCTF	0.19962	1	2	4
-CCTP	0.19962	1	2	21
-CCTS	0.19962	3	6	37
-CCTW	0.19962	1	2	23
-CCVD	0.19962	1	2	21
-CCWC	0.19962	1	2	6
-CCWE	0.19962	1	2	4
-CCWG	0.19962	1	2	7

-CCWI	0.19962	1	2	3
-CCYC	0.19962	1	2	8
-CCYW	0.19962	1	2	7
-CDAF	0.19962	1	2	19
-CDAP	0.19962	2	4	15
-CDCK	0.19962	1	2	2
-CDEC	0.19962	1	2	7
-CDED	0.19962	1	2	6
-CDEI	0.19962	1	2	2
-CDEK	0.19962	2	4	6
-CDEM	0.19962	1	2	7
-CDFF	0.19962	1	2	4
-CDFL	0.19962	2	4	18
-CDFV	0.19962	1	2	8
-CDHE	0.19962	1	2	10
-CDIW	0.19962	1	2	6
-CDKE	0.19962	2	4	16
-CDKQ	0.19962	1	2	7
-CDKW	0.19962	1	2	7
-CDMC	0.19962	1	2	7
-CDMD	0.19962	1	2	4
-CDPC	0.19962	1	2	16
-CDPW	0.19962	1	2	16
-CDQH	0.19962	1	2	15
-CDRI	0.19962	3	6	15
-CDSQ	0.19962	2	4	15
-CDTY	0.19962	1	2	11
-CDVY	0.19962	1	2	10
-CDWQ	0.19962	1	2	10
-CEAC	0.19962	1	2	6
-CECH	0.19962	1	2	5
-CECK	0.19962	1	2	8
-CECW	0.19962	1	2	4
-CECY	0.19962	1	2	8
-CEDY	0.19962	1	2	3
-CEEK	0.19962	1	2	4
-CEEN	0.19962	1	2	6
-CEET	0.19962	1	2	3
-CEFC	0.19962	1	2	4
-CEFE	0.19962	1	2	4
-CEFI	0.19962	2	4	5
-CEFW	0.19962	1	2	7
-CEGH	0.19962	1	2	1
-CEGP	0.19962	1	2	5
-CEHA	0.19962	1	2	2
-CEHD	0.19962	1	2	7
-CEHF	0.19962	1	2	3
-CEHG	0.19962	1	2	3
-CEHP	0.19962	1	2	7
-CEHS	0.19962	1	2	19
-CEHW	0.19962	1	2	5
-CEIK	0.19962	2	4	7
-CEIR	0.19962	3	6	19
-CEIW	0.19962	1	2	7
-CEKC	0.19962	1	2	5
-CEKF	0.19962	1	2	5
-CEKT	0.19962	1	2	5

-CEMF	0.19962	1	2	16
-CEMH	0.19962	1	2	1
-CEMW	0.19962	1	2	9
-CEMY	0.19962	1	2	2
-CENI	0.19962	1	2	6
-CENP	0.19962	2	4	11
-CEPH	0.19962	1	2	14
-CEPM	0.19962	1	2	15
-CEQK	0.19962	1	2	3
-CEQN	0.19962	1	2	9
-CETN	0.19962	2	4	11
-CEWK	0.19962	1	2	5
-CEWR	0.19962	1	2	28
-CEWV	0.19962	1	2	12
-CEYF	0.19962	1	2	2
-CEYN	0.19962	2	4	8
-CFAI	0.19962	1	2	13
-CFAN	0.19962	1	2	18
-CFCC	0.19962	1	2	3
-CFCG	0.19962	1	2	7
-CFCW	0.19962	1	2	12
-CFDC	0.19962	1	2	3
-CFDE	0.19962	1	2	6
-CFDY	0.19962	1	2	3
-CFED	0.19962	1	2	1
-CFEE	0.19962	2	4	9
-CFEM	0.19962	1	2	4
-CFEN	0.19962	2	4	17
-CFEQ	0.19962	1	2	8
-CFEY	0.19962	1	2	11
-CFFA	0.19962	1	2	15
-CFFH	0.19962	1	2	12
-CFFI	0.19962	1	2	13
-CFFP	0.19962	1	2	16
-CFFS	0.19962	1	2	13
-CFGC	0.19962	1	2	5
-CFGF	0.19962	1	2	12
-CFGI	0.19962	1	2	16
-CFGP	0.19962	1	2	7
-CFGQ	0.19962	1	2	6
-CFGY	0.19962	1	2	8
-CFHR	0.19962	1	2	12
-CFHS	0.19962	1	2	15
-CFHY	0.19962	1	2	1
-CFII	0.19962	1	2	1
-CFIR	0.19962	1	2	18
-CFKF	0.19962	2	4	10
-CFKN	0.19962	1	2	8
-CFKW	0.19962	2	4	21
-CFLE	0.19962	4	8	7
-CFLH	0.19962	3	6	38
-CFLV	0.19962	1	2	37
-CFML	0.19962	1	2	26
-CFNA	0.19962	1	2	12
-CFNH	0.19962	1	2	8
-CFNV	0.19962	1	2	7
-CFNW	0.19962	2	4	3

-CFNY	0.19962	1	2	2
-CFPS	0.19962	2	4	33
-CFQC	0.19962	1	2	17
-CFQK	0.19962	2	4	14
-CFQN	0.19962	1	2	4
-CFQR	0.19962	2	4	28
-CFQS	0.19962	1	2	7
-CFQW	0.19962	1	2	17
-CFQY	0.19962	1	2	3
-CFRG	0.19962	2	4	35
-CFSW	0.19962	1	2	20
-CFTT	0.19962	2	4	17
-CFTY	0.19962	1	2	7
-CFVD	0.19962	2	4	13
-CFVE	0.19962	1	2	9
-CFVF	0.19962	1	2	9
-CFVY	0.19962	1	2	3
-CFWD	0.19962	1	2	5
-CFWI	0.19962	1	2	11
-CFWL	0.19962	1	2	17
-CFWN	0.19962	1	2	14
-CFWQ	0.19962	1	2	11
-CFWS	0.19962	2	4	9
-CFYK	0.19962	1	2	7
-CFYN	0.19962	1	2	7
-CFYT	0.19962	3	6	16
-CGAT	0.19962	3	6	23
-CGCK	0.19962	1	2	17
-CGCN	0.19962	2	4	6
-CGCQ	0.19962	1	2	10
-CGDM	0.19962	1	2	24
-CGED	0.19962	3	6	11
-CGEI	0.19962	1	2	6
-CGEW	0.19962	2	4	8
-CGFH	0.19962	1	2	2
-CGFI	0.19962	1	2	8
-CGGF	0.19962	1	2	11
-CGGH	0.19962	1	2	8
-CGGL	0.19962	6	12	36
-CGGY	0.19962	2	4	6
-CGHG	0.19962	2	4	29
-CGHI	0.19962	1	2	13
-CGHM	0.19962	1	2	18
-CGHR	0.19962	2	4	26
-CGHT	0.19962	1	2	16
-CGIK	0.19962	1	2	14
-CGIW	0.19962	2	4	6
-CGKP	0.19962	2	4	8
-CGLK	0.19962	1	2	23
-CGMI	0.19962	1	2	8
-CGMN	0.19962	3	6	23
-CGNK	0.19962	1	2	9
-CGPY	0.19962	1	2	11
-CGQM	0.19962	1	2	8
-CGQP	0.19962	1	2	7
-CGRD	0.19962	2	4	23
-CGVH	0.19962	2	4	14

-CGWL	0.19962	1	2	15
-CGWQ	0.19962	1	2	14
-CGWS	0.19962	2	4	18
-CGWV	0.19962	1	2	12
-CGWY	0.19962	1	2	10
-CGYA	0.19962	1	2	18
-CGYG	0.19962	1	2	14
-CGYK	0.19962	1	2	14
-CHAF	0.19962	1	2	12
-CHAH	0.19962	1	2	7
-CHAK	0.19962	1	2	8
-CHAR	0.19962	2	4	24
-CHAT	0.19962	1	2	14
-CHCH	0.19962	1	2	10
-CHCP	0.19962	1	2	12
-CHCW	0.19962	1	2	9
-CHCY	0.19962	1	2	10
-CHDC	0.19962	1	2	11
-CHDN	0.19962	1	2	3
-CHEC	0.19962	1	2	10
-CHEH	0.19962	1	2	4
-CHEW	0.19962	1	2	5
-CHFE	0.19962	1	2	2
-CHFR	0.19962	1	2	13
-CHFW	0.19962	1	2	7
-CHGN	0.19962	1	2	19
-CHGV	0.19962	3	6	16
-CHGW	0.19962	1	2	12
-CHHE	0.19962	1	2	8
-CHHN	0.19962	1	2	7
-CHHT	0.19962	2	4	20
-CHKK	0.19962	2	4	15
-CHKP	0.19962	1	2	10
-CHKQ	0.19962	1	2	10
-CHLD	0.19962	4	8	26
-CHMA	0.19962	1	2	10
-CHMY	0.19962	1	2	5
-CHPC	0.19962	1	2	15
-CHPH	0.19962	2	4	16
-CHPK	0.19962	1	2	17
-CHPY	0.19962	2	4	13
-CHQA	0.19962	1	2	9
-CHQD	0.19962	1	2	6
-CHQF	0.19962	1	2	9
-CHQH	0.19962	1	2	8
-CHQI	0.19962	1	2	5
-CHQK	0.19962	1	2	8
-CHQR	0.19962	3	6	28
-CHSH	0.19962	2	4	7
-CHSY	0.19962	2	4	15
-CHVG	0.19962	1	2	23
-CHWC	0.19962	1	2	3
-CHWD	0.19962	1	2	8
-CHWE	0.19962	1	2	5
-CHWF	0.19962	1	2	2
-CHWG	0.19962	1	2	4
-CHWH	0.19962	1	2	3

-CHWL	0.19962	1	2	22
-CHWN	0.19962	1	2	14
-CHWP	0.19962	1	2	13
-CHWS	0.19962	1	2	21
-CHWT	0.19962	1	2	16
-CHWV	0.19962	2	4	13
-CHYE	0.19962	2	4	16
-CHYL	0.19962	1	2	33
-CHYR	0.19962	1	2	20
-CIAA	0.19962	2	4	19
-CIAD	0.19962	1	2	11
-CIAI	0.19962	2	4	16
-CIDN	0.19962	2	4	12
-CIDW	0.19962	1	2	6
-CIEI	0.19962	1	2	5
-CIEM	0.19962	1	2	10
-CIFD	0.19962	1	2	9
-CIFG	0.19962	1	2	10
-CIFK	0.19962	1	2	8
-CIFY	0.19962	1	2	3
-CIGA	0.19962	4	8	14
-CIGD	0.19962	2	4	11
-CIGH	0.19962	1	2	8
-CIGK	0.19962	1	2	9
-CIGS	0.19962	4	8	38
-CIGY	0.19962	1	2	7
-CIHE	0.19962	2	4	10
-CIHI	0.19962	1	2	7
-CIHT	0.19962	1	2	6
-CIHY	0.19962	1	2	2
-CIKM	0.19962	2	4	15
-CIKQ	0.19962	1	2	7
-CILD	0.19962	2	4	26
-CIME	0.19962	1	2	4
-CIMW	0.19962	1	2	4
-CIMY	0.19962	1	2	10
-CIND	0.19962	1	2	9
-CING	0.19962	1	2	10
-CINK	0.19962	2	4	15
-CINR	0.19962	1	2	25
-CINS	0.19962	3	6	29
-CINW	0.19962	1	2	4
-CIPE	0.19962	1	2	14
-CIPY	0.19962	1	2	7
-CIQF	0.19962	1	2	13
-CIQN	0.19962	2	4	10
-CIQV	0.19962	1	2	6
-CIQW	0.19962	1	2	11
-CITT	0.19962	1	2	22
-CIWA	0.19962	1	2	6
-CIWD	0.19962	1	2	8
-CIWG	0.19962	1	2	9
-CIWP	0.19962	1	2	8
-CIWT	0.19962	1	2	9
-CIYC	0.19962	1	2	14
-CIYN	0.19962	1	2	5
-CIYQ	0.19962	1	2	18

-CIYT	0.19962	2	4	15
-CIYY	0.19962	1	2	4
-CKAQ	0.19962	4	8	20
-CKAW	0.19962	1	2	13
-CKCC	0.19962	1	2	18
-CKCI	0.19962	1	2	6
-CKEF	0.19962	1	2	5
-CKEH	0.19962	2	4	4
-CKEI	0.19962	1	2	10
-CKEK	0.19962	1	2	4
-CKEN	0.19962	1	2	7
-CKET	0.19962	2	4	10
-CKFL	0.19962	2	4	30
-CKFY	0.19962	1	2	9
-CKGD	0.19962	1	2	7
-CKGH	0.19962	1	2	4
-CKGK	0.19962	1	2	7
-CKHE	0.19962	1	2	6
-CKHP	0.19962	1	2	7
-CKHQ	0.19962	1	2	3
-CKHT	0.19962	1	2	9
-CKHV	0.19962	1	2	13
-CKII	0.19962	1	2	12
-CKIN	0.19962	1	2	10
-CKIP	0.19962	1	2	11
-CKIY	0.19962	1	2	15
-CKKT	0.19962	1	2	5
-CKKY	0.19962	2	4	9
-CKMA	0.19962	1	2	8
-CKMH	0.19962	1	2	9
-CKMV	0.19962	1	2	13
-CKNY	0.19962	3	6	11
-CKQN	0.19962	1	2	7
-CKQW	0.19962	1	2	13
-CKRY	0.19962	1	2	20
-CKTD	0.19962	2	4	19
-CKTI	0.19962	1	2	12
-CKVF	0.19962	1	2	8
-CKVN	0.19962	1	2	8
-CKVQ	0.19962	3	6	16
-CKVW	0.19962	1	2	20
-CKWN	0.19962	2	4	18
-CKWW	0.19962	1	2	1
-CKYA	0.19962	1	2	9
-CKYF	0.19962	2	4	8
-CKYM	0.19962	1	2	14
-CLAF	0.19962	4	8	46
-CLCD	0.19962	1	2	6
-CLCI	0.19962	1	2	18
-CLCV	0.19962	3	6	30
-CLCY	0.19962	1	2	10
-CLDE	0.19962	3	6	13
-CLFA	0.19962	1	2	19
-CLHF	0.19962	1	2	18
-CLHH	0.19962	2	4	21
-CLHL	0.19962	6	12	81
-CLHY	0.19962	1	2	19

-CLIW	0.19962	1	2	17
-CLKC	0.19962	2	4	20
-CLLH	0.19962	4	8	33
-CLMI	0.19962	2	4	24
-CLMW	0.19962	1	2	28
-CLMY	0.19962	1	2	11
-CLNW	0.19962	2	4	33
-CLPA	0.19962	7	14	47
-CLQW	0.19962	1	2	14
-CLRQ	0.19962	7	14	35
-CLVD	0.19962	1	2	20
-CLWE	0.19962	1	2	8
-CLWN	0.19962	1	2	19
-CLWS	0.19962	3	6	51
-CLYM	0.19962	2	4	25
-CLYW	0.19962	1	2	28
-CMAC	0.19962	1	2	4
-CMAF	0.19962	1	2	9
-CMAL	0.19962	4	8	20
-CMAM	0.19962	1	2	8
-CMCD	0.19962	1	2	12
-CMCV	0.19962	2	4	8
-CMDH	0.19962	1	2	10
-CMEK	0.19962	1	2	7
-CMFG	0.19962	1	2	5
-CMFQ	0.19962	2	4	10
-CMFW	0.19962	1	2	8
-CMGH	0.19962	2	4	5
-CMGR	0.19962	1	2	18
-CMHP	0.19962	1	2	17
-CMHW	0.19962	2	4	10
-CMHY	0.19962	1	2	12
-CMKC	0.19962	1	2	6
-CMKD	0.19962	1	2	8
-CMLY	0.19962	2	4	16
-CMML	0.19962	2	4	15
-CMMP	0.19962	1	2	14
-CMMT	0.19962	3	6	39
-CMNC	0.19962	1	2	6
-CMND	0.19962	1	2	6
-CMNH	0.19962	1	2	5
-CMNN	0.19962	1	2	11
-CMPC	0.19962	2	4	22
-CMPF	0.19962	1	2	19
-CMPH	0.19962	1	2	7
-CMPS	0.19962	4	8	41
-CMQD	0.19962	1	2	2
-CMQE	0.19962	2	4	6
-CMQG	0.19962	1	2	9
-CMQI	0.19962	1	2	3
-CMQK	0.19962	1	2	7
-CMRV	0.19962	2	4	17
-CMRW	0.19962	1	2	19
-CMSH	0.19962	1	2	12
-CMVW	0.19962	1	2	8
-CMVY	0.19962	1	2	11
-CMWA	0.19962	1	2	12

-CMWD	0.19962	1	2	8
-CMWF	0.19962	1	2	10
-CMWK	0.19962	1	2	11
-CMWS	0.19962	1	2	15
-CMWT	0.19962	1	2	6
-CMWV	0.19962	1	2	12
-CMYP	0.19962	1	2	20
-CMYQ	0.19962	1	2	14
-CNCH	0.19962	2	4	2
-CNCI	0.19962	2	4	6
-CNCY	0.19962	1	2	11
-CNDN	0.19962	1	2	7
-CNDT	0.19962	1	2	11
-CNDV	0.19962	1	2	18
-CNDW	0.19962	1	2	6
-CNEA	0.19962	1	2	9
-CNEH	0.19962	1	2	8
-CNET	0.19962	1	2	2
-CNFF	0.19962	1	2	13
-CNFI	0.19962	2	4	18
-CNFP	0.19962	1	2	7
-CNFW	0.19962	1	2	5
-CNGD	0.19962	1	2	6
-CNHC	0.19962	1	2	8
-CNHM	0.19962	1	2	16
-CNHP	0.19962	2	4	13
-CNHQ	0.19962	1	2	10
-CNHT	0.19962	1	2	13
-CNIH	0.19962	1	2	4
-CNKQ	0.19962	1	2	14
-CNMP	0.19962	2	4	11
-CNMY	0.19962	1	2	10
-CNNP	0.19962	1	2	15
-CNNQ	0.19962	1	2	9
-CNNY	0.19962	1	2	9
-CNPC	0.19962	1	2	9
-CNPM	0.19962	1	2	17
-CNQC	0.19962	1	2	14
-CNQE	0.19962	1	2	2
-CNQF	0.19962	1	2	8
-CNQK	0.19962	1	2	8
-CNQM	0.19962	1	2	8
-CNQV	0.19962	1	2	17
-CNSH	0.19962	3	6	21
-CNSY	0.19962	2	4	22
-CNTM	0.19962	1	2	21
-CNTQ	0.19962	1	2	25
-CNVE	0.19962	2	4	21
-CNVH	0.19962	6	12	21
-CNWA	0.19962	1	2	6
-CNWD	0.19962	1	2	4
-CNWE	0.19962	1	2	7
-CNWG	0.19962	1	2	11
-CNWN	0.19962	1	2	4
-CNWR	0.19962	2	4	28
-CNWY	0.19962	1	2	9
-CNYI	0.19962	1	2	16

-CNYP	0.19962	1	2	14
-CNYR	0.19962	2	4	28
-CPAV	0.19962	1	2	26
-CPCR	0.19962	2	4	56
-CPCS	0.19962	3	6	29
-CPEN	0.19962	4	8	16
-CPFC	0.19962	1	2	10
-CPFH	0.19962	1	2	8
-CPGC	0.19962	1	2	11
-CPGH	0.19962	1	2	7
-CPGI	0.19962	2	4	20
-CPGK	0.19962	2	4	8
-CPGP	0.19962	2	4	16
-CPGW	0.19962	3	6	23
-CPHP	0.19962	3	6	15
-CPIH	0.19962	1	2	15
-CPKF	0.19962	2	4	8
-CPKM	0.19962	1	2	19
-CPKQ	0.19962	1	2	10
-CPMG	0.19962	1	2	7
-CPNC	0.19962	1	2	8
-CPNW	0.19962	2	4	14
-CPPD	0.19962	2	4	8
-CPPE	0.19962	2	4	12
-CPPF	0.19962	2	4	21
-CPQP	0.19962	1	2	13
-CPVL	0.19962	12	24	57
-CPWF	0.19962	1	2	8
-CPWI	0.19962	1	2	10
-CPWK	0.19962	1	2	18
-CPWR	0.19962	1	2	35
-CPWS	0.19962	1	2	27
-CPWV	0.19962	1	2	15
-CPWW	0.19962	1	2	13
-CPWY	0.19962	1	2	4
-CPYA	0.19962	2	4	26
-CPYW	0.19962	1	2	23
-CQAG	0.19962	2	4	11
-CQAW	0.19962	1	2	19
-CQCE	0.19962	1	2	7
-CQCN	0.19962	1	2	15
-CQCQ	0.19962	1	2	15
-CQCR	0.19962	3	6	13
-CQDH	0.19962	1	2	4
-CQDY	0.19962	1	2	4
-CQED	0.19962	1	2	7
-CQEH	0.19962	1	2	7
-CQEK	0.19962	1	2	11
-CQEM	0.19962	2	4	5
-CQEN	0.19962	1	2	12
-CQEV	0.19962	1	2	3
-CQFC	0.19962	1	2	16
-CQFD	0.19962	3	6	3
-CQFE	0.19962	1	2	7
-CQFK	0.19962	1	2	11
-CQFP	0.19962	1	2	5
-CQGH	0.19962	1	2	6

-CQHM	0.19962	1	2	7
-CQIK	0.19962	1	2	11
-CQKF	0.19962	1	2	4
-CQKG	0.19962	1	2	5
-CQKI	0.19962	1	2	1
-CQKK	0.19962	1	2	4
-CQKY	0.19962	1	2	6
-CQMA	0.19962	1	2	19
-CQMR	0.19962	2	4	16
-CQNP	0.19962	1	2	10
-CQNQ	0.19962	2	4	13
-CQPY	0.19962	1	2	9
-CQQC	0.19962	1	2	7
-CQQD	0.19962	1	2	12
-CQQT	0.19962	1	2	10
-CQRC	0.19962	2	4	9
-CQSC	0.19962	1	2	9
-CQTC	0.19962	1	2	7
-CQTI	0.19962	2	4	16
-CQTY	0.19962	1	2	12
-CQVK	0.19962	4	8	12
-CQVN	0.19962	1	2	5
-CQWP	0.19962	2	4	11
-CQWV	0.19962	1	2	5
-CQYC	0.19962	1	2	4
-CQYK	0.19962	1	2	2
-CQYT	0.19962	1	2	15
-CRCN	0.19962	1	2	7
-CRCS	0.19962	6	12	31
-CRCT	0.19962	3	6	35
-CRCY	0.19962	1	2	9
-CRFI	0.19962	1	2	18
-CRFY	0.19962	1	2	15
-CRGH	0.19962	1	2	1
-CRGP	0.19962	1	2	23
-CRHH	0.19962	3	6	27
-CRHR	0.19962	3	6	50
-CRID	0.19962	1	2	18
-CRIE	0.19962	2	4	22
-CRIG	0.19962	1	2	28
-CRIK	0.19962	1	2	11
-CRIR	0.19962	6	12	58
-CRIW	0.19962	1	2	23
-CRKH	0.19962	1	2	15
-CRKI	0.19962	1	2	24
-CRLT	0.19962	3	6	73
-CRMH	0.19962	1	2	13
-CRMY	0.19962	2	4	17
-CRNP	0.19962	1	2	26
-CRPQ	0.19962	1	2	24
-CRQC	0.19962	1	2	11
-CRQG	0.19962	3	6	23
-CRQK	0.19962	2	4	12
-CRQQ	0.19962	1	2	20
-CRQW	0.19962	1	2	10
-CRRW	0.19962	1	2	35
-CRSF	0.19962	2	4	25

-CRTF	0.19962	1	2	18
-CRTM	0.19962	2	4	39
-CRTW	0.19962	2	4	20
-CRVD	0.19962	3	6	21
-CRWE	0.19962	2	4	13
-CRWG	0.19962	1	2	13
-CRWM	0.19962	1	2	14
-CRWR	0.19962	2	4	34
-CRWY	0.19962	1	2	24
-CRYF	0.19962	1	2	15
-CRYV	0.19962	4	8	29
-CSDQ	0.19962	2	4	10
-CSEY	0.19962	3	6	10
-CSFQ	0.19962	1	2	27
-CSFW	0.19962	1	2	22
-CSFY	0.19962	1	2	12
-CSHF	0.19962	2	4	32
-CSHH	0.19962	1	2	16
-CSHW	0.19962	1	2	9
-CSKA	0.19962	4	8	36
-CSKH	0.19962	1	2	19
-CSLH	0.19962	4	8	59
-CSMK	0.19962	3	6	27
-CSMT	0.19962	4	8	49
-CSNP	0.19962	2	4	27
-CSNY	0.19962	2	4	20
-CSPS	0.19962	4	8	61
-CSQY	0.19962	1	2	14
-CSRQ	0.19962	2	4	31
-CSWI	0.19962	1	2	8
-CSWN	0.19962	1	2	21
-CSWY	0.19962	1	2	21
-CSYQ	0.19962	2	4	14
-CTAC	0.19962	2	4	12
-CTCI	0.19962	3	6	20
-CTCW	0.19962	1	2	16
-CTDC	0.19962	1	2	19
-CTDI	0.19962	2	4	11
-CTDV	0.19962	8	16	33
-CTEN	0.19962	2	4	19
-CTFF	0.19962	1	2	13
-CTGC	0.19962	1	2	22
-CTGM	0.19962	3	6	15
-CTHE	0.19962	2	4	21
-CTHF	0.19962	2	4	16
-CTHY	0.19962	1	2	10
-CTKN	0.19962	1	2	11
-CTPM	0.19962	1	2	35
-CTQH	0.19962	1	2	4
-CTQI	0.19962	2	4	4
-CTQQ	0.19962	1	2	7
-CTQW	0.19962	1	2	13
-CTTQ	0.19962	1	2	30
-CTVH	0.19962	3	6	35
-CTWA	0.19962	1	2	8
-CTWC	0.19962	1	2	16
-CTWE	0.19962	1	2	13

-CTWK	0.19962	2	4	15
-CTYA	0.19962	2	4	16
-CTYE	0.19962	1	2	17
-CTYW	0.19962	1	2	11
-CVCP	0.19962	3	6	17
-CVCR	0.19962	1	2	28
-CVED	0.19962	2	4	24
-CVFD	0.19962	1	2	12
-CVGC	0.19962	1	2	14
-CVGW	0.19962	2	4	17
-CVHD	0.19962	1	2	10
-CVKQ	0.19962	1	2	5
-CVME	0.19962	2	4	18
-CVMW	0.19962	1	2	16
-CVNI	0.19962	1	2	12
-CVNT	0.19962	1	2	21
-CVPH	0.19962	1	2	24
-CVPM	0.19962	1	2	14
-CVPW	0.19962	1	2	16
-CVTH	0.19962	3	6	32
-CVWD	0.19962	1	2	17
-CVWE	0.19962	1	2	4
-CVWG	0.19962	1	2	14
-CVWH	0.19962	1	2	12
-CVYT	0.19962	2	4	21
-CVYY	0.19962	1	2	9
-CWAE	0.19962	1	2	7
-CWAG	0.19962	2	4	6
-CWAI	0.19962	1	2	14
-CWAW	0.19962	1	2	10
-CWCS	0.19962	2	4	16
-CWDE	0.19962	1	2	1
-CWDQ	0.19962	1	2	2
-CWEC	0.19962	1	2	6
-CWEV	0.19962	1	2	9
-CWEW	0.19962	1	2	4
-CWFR	0.19962	1	2	11
-CWGA	0.19962	1	2	8
-CWGP	0.19962	1	2	1
-CWGR	0.19962	2	4	8
-CWGT	0.19962	1	2	11
-CWGW	0.19962	1	2	9
-CWHC	0.19962	1	2	9
-CWHW	0.19962	1	2	1
-CWHY	0.19962	1	2	16
-CWKI	0.19962	1	2	5
-CWKP	0.19962	1	2	6
-CWLN	0.19962	1	2	10
-CWLS	0.19962	11	22	47
-CWLY	0.19962	1	2	11
-CWME	0.19962	1	2	14
-CWMG	0.19962	1	2	9
-CWMK	0.19962	1	2	17
-CWML	0.19962	1	2	8
-CWMT	0.19962	1	2	18
-CWMV	0.19962	1	2	14
-CWNF	0.19962	1	2	10

-CWNK	0.19962	1	2	11
-CWNV	0.19962	1	2	6
-CWPA	0.19962	1	2	8
-CWPI	0.19962	1	2	8
-CWPK	0.19962	1	2	6
-CWPM	0.19962	2	4	10
-CWQG	0.19962	1	2	13
-CWQM	0.19962	1	2	3
-CWRC	0.19962	1	2	15
-CWRD	0.19962	1	2	12
-CWRE	0.19962	1	2	4
-CWRQ	0.19962	1	2	10
-CWSI	0.19962	1	2	16
-CWSN	0.19962	1	2	17
-CWSQ	0.19962	1	2	15
-CWSS	0.19962	6	12	29
-CWTF	0.19962	2	4	13
-CWTI	0.19962	1	2	25
-CWTV	0.19962	1	2	33
-CWVA	0.19962	1	2	20
-CWVN	0.19962	1	2	13
-CWWG	0.19962	1	2	15
-CWWL	0.19962	1	2	15
-CWWM	0.19962	1	2	4
-CWWP	0.19962	1	2	10
-CWWS	0.19962	1	2	26
-CWYA	0.19962	1	2	13
-CWYI	0.19962	1	2	3
-CWYK	0.19962	1	2	10
-CWYL	0.19962	1	2	22
-CYAQ	0.19962	1	2	18
-CYDD	0.19962	1	2	5
-CYEC	0.19962	1	2	5
-CYFD	0.19962	1	2	6
-CYFH	0.19962	1	2	6
-CYFL	0.19962	1	2	18
-CYFN	0.19962	1	2	16
-CYFS	0.19962	1	2	4
-CYGP	0.19962	2	4	22
-CYGR	0.19962	2	4	33
-CYGW	0.19962	1	2	8
-CYHG	0.19962	2	4	4
-CYHQ	0.19962	2	4	8
-CYHT	0.19962	1	2	13
-CYMV	0.19962	1	2	24
-CYMY	0.19962	1	2	15
-CYNF	0.19962	1	2	6
-CYNG	0.19962	1	2	23
-CYNT	0.19962	1	2	14
-CYQA	0.19962	1	2	3
-CYQF	0.19962	1	2	19
-CYQM	0.19962	1	2	14
-CYQS	0.19962	2	4	21
-CYQW	0.19962	1	2	11
-CYRW	0.19962	1	2	6
-CYSM	0.19962	1	2	20
-CYVV	0.19962	4	8	40

-CYWA	0.19962	1	2	8
-CYWH	0.19962	1	2	7
-CYWQ	0.19962	1	2	11
-CYYA	0.19962	2	4	12
-CYYD	0.19962	1	2	6
-CYYE	0.19962	1	2	17
-CYYK	0.19962	1	2	13
-CYYW	0.19962	1	2	4
-CGVT	0.19962	5	10	20
-CSQN	0.19962	5	10	41
-CVVD	0.19962	5	10	14
-CSSS	0.20606	32	62	114
-CIVR	0.20913	11	21	46
-CPQR	0.21293	8	15	25
-CTCR	0.21498	7	13	50
-CCHL	0.21777	6	11	12
-CDRD	0.21777	6	11	19
-CHDL	0.21777	6	11	41
-CHRL	0.21777	6	11	46
-CQYA	0.21777	6	11	12
-CTYV	0.21777	6	11	34
-CCLN	0.2218	5	9	26
-CCSM	0.2218	5	9	23
-CGRA	0.2218	5	9	35
-CLVY	0.2218	5	9	21
-CMRG	0.2218	5	9	25
-CRMR	0.2218	5	9	79
-CVDP	0.2218	5	9	15
-CNSK	0.22358	14	25	34
-CRRA	0.22458	9	16	44
-CSYN	0.22458	9	16	30
-CTML	0.22458	9	16	54
-CVMR	0.22458	9	16	38
-CDDH	0.22814	4	7	11
-CDNG	0.22814	4	7	15
-CEVH	0.22814	4	7	13
-CGDP	0.22814	4	7	20
-CHLN	0.22814	4	7	32
-CHSQ	0.22814	4	7	26
-CHVD	0.22814	4	7	16
-CHVH	0.22814	4	7	20
-CIHL	0.22814	8	14	23
-CILE	0.22814	4	7	20
-CLDH	0.22814	4	7	13
-CLML	0.22814	4	7	89
-CMCS	0.22814	4	7	16
-CRDP	0.22814	4	7	28
-CRRQ	0.22814	4	7	58
-CSEH	0.22814	4	7	18
-CTEF	0.22814	4	7	25
-CVCL	0.22814	4	7	32
-CPSD	0.23114	11	19	27
-CAAL	0.23289	7	12	55
-CMYA	0.23289	7	12	21
-CSIP	0.23289	7	12	45
-CPKT	0.23485	10	17	21
-CACR	0.23955	6	10	42

-CADV	0.23955	3	5	11
-CAEN	0.23955	3	5	14
-CAYS	0.23955	3	5	31
-CCEE	0.23955	3	5	2
-CCHP	0.23955	3	5	15
-CCPA	0.23955	3	5	13
-CCVH	0.23955	3	5	15
-CDGC	0.23955	3	5	13
-CDSC	0.23955	3	5	8
-CELP	0.23955	6	10	41
-CELV	0.23955	3	5	30
-CEQV	0.23955	3	5	15
-CEVI	0.23955	3	5	23
-CFDG	0.23955	3	5	14
-CFTE	0.23955	3	5	19
-CGTK	0.23955	3	5	14
-CHHP	0.23955	3	5	21
-CHHS	0.23955	3	5	32
-CHKY	0.23955	3	5	11
-CHNG	0.23955	3	5	15
-CHRT	0.23955	3	5	30
-CIEH	0.23955	3	5	14
-CKNV	0.23955	3	5	10
-CLEN	0.23955	3	5	22
-CLQA	0.23955	3	5	32
-CMCR	0.23955	3	5	20
-CMEH	0.23955	3	5	9
-CNGI	0.23955	3	5	11
-CNNA	0.23955	3	5	9
-CNWV	0.23955	3	5	22
-CPCD	0.23955	3	5	6
-CPYL	0.23955	3	5	56
-CQAK	0.23955	3	5	13
-CQLV	0.23955	3	5	18
-CQNC	0.23955	3	5	9
-CRVF	0.23955	3	5	14
-CSCP	0.23955	3	5	23
-CSTH	0.23955	3	5	37
-CTCA	0.23955	6	10	20
-CTCT	0.23955	3	5	27
-CVDH	0.23955	3	5	20
-CVWS	0.23955	3	5	28
-CWFL	0.23955	6	10	27
-CWLA	0.23955	3	5	27
-CWLL	0.23955	3	5	39
-CWVV	0.23955	3	5	18
-CYAV	0.23955	3	5	21
-CYCS	0.23955	3	5	13
-CYEQ	0.23955	3	5	10
-CYNQ	0.23955	3	5	11
-CYRR	0.23955	3	5	48
-CELS	0.24165	23	38	68
-CASA	0.24398	11	18	38
-CELL	0.24398	11	18	53
-CVAS	0.24781	18	29	46
-CHLP	0.24953	5	8	25
-CHMS	0.24953	5	8	21

-CPVS	0.24953	5	8	48
-CQAE	0.24953	5	8	17
-CRVN	0.24953	5	8	40
-CVDL	0.24953	10	16	39
-CYVH	0.24953	5	8	27
-CLVG	0.25216	12	19	28
-CALP	0.25407	7	11	60
-CIDL	0.25407	7	11	36
-CLRK	0.25407	7	11	56
-CVSC	0.25407	7	11	24
-CYLT	0.25407	7	11	29
-CACS	0.25552	32	50	84
-CTCV	0.25666	9	14	35
-CVTI	0.25666	9	14	20
-CADH	0.26616	2	3	13
-CAEP	0.26616	2	3	23
-CAGF	0.26616	2	3	13
-CALW	0.26616	2	3	25
-CALY	0.26616	4	6	36
-CAQA	0.26616	2	3	20
-CARI	0.26616	2	3	21
-CARW	0.26616	2	3	15
-CAWC	0.26616	2	3	6
-CAYN	0.26616	4	6	30
-CAYY	0.26616	2	3	16
-CCAW	0.26616	2	3	7
-CCCD	0.26616	2	3	10
-CCDI	0.26616	2	3	4
-CCFI	0.26616	2	3	4
-CCFT	0.26616	2	3	22
-CCFV	0.26616	2	3	6
-CCGP	0.26616	2	3	14
-CCGR	0.26616	2	3	28
-CCHG	0.26616	2	3	15
-CCHQ	0.26616	2	3	9
-CCHR	0.26616	2	3	19
-CCHS	0.26616	2	3	25
-CCLE	0.26616	2	3	22
-CCLY	0.26616	2	3	28
-CCMG	0.26616	2	3	13
-CCNC	0.26616	2	3	12
-CCPM	0.26616	2	3	33
-CCRF	0.26616	2	3	19
-CCSQ	0.26616	2	3	7
-CCWD	0.26616	2	3	2
-CDAQ	0.26616	2	3	13
-CDDY	0.26616	2	3	11
-CDHV	0.26616	4	6	34
-CDKS	0.26616	2	3	20
-CDLE	0.26616	2	3	16
-CDND	0.26616	2	3	15
-CDQM	0.26616	2	3	6
-CDWG	0.26616	2	3	4
-CDYP	0.26616	2	3	8
-CEAQ	0.26616	2	3	5
-CECC	0.26616	2	3	20
-CEDN	0.26616	4	6	13

-CEIN	0.26616	2	3	7
-CENS	0.26616	2	3	23
-CEQL	0.26616	2	3	15
-CEWL	0.26616	2	3	11
-CFEG	0.26616	2	3	8
-CFGH	0.26616	2	3	10
-CFMI	0.26616	2	3	9
-CFMS	0.26616	2	3	17
-CFNR	0.26616	2	3	28
-CFQH	0.26616	2	3	9
-CFRC	0.26616	2	3	12
-CFTC	0.26616	2	3	8
-CFTL	0.26616	4	6	37
-CFTW	0.26616	2	3	10
-CGAY	0.26616	2	3	8
-CGCF	0.26616	2	3	8
-CGCY	0.26616	2	3	7
-CGDY	0.26616	2	3	10
-CGNE	0.26616	2	3	8
-CGNF	0.26616	4	6	16
-CGPH	0.26616	2	3	20
-CGQC	0.26616	2	3	11
-CGQI	0.26616	2	3	14
-CGWR	0.26616	2	3	19
-CHCI	0.26616	2	3	4
-CHCV	0.26616	2	3	7
-CHHY	0.26616	2	3	14
-CHLW	0.26616	2	3	13
-CHRQ	0.26616	4	6	22
-CHYV	0.26616	2	3	14
-CIAP	0.26616	2	3	22
-CICK	0.26616	2	3	7
-CIFT	0.26616	2	3	16
-CIHS	0.26616	2	3	14
-CIIP	0.26616	2	3	7
-CILP	0.26616	2	3	16
-CIPN	0.26616	2	3	19
-CIQI	0.26616	2	3	16
-CKAE	0.26616	2	3	11
-CKAY	0.26616	2	3	10
-CKER	0.26616	2	3	5
-CKFD	0.26616	2	3	18
-CKGE	0.26616	2	3	5
-CKHC	0.26616	2	3	7
-CKKK	0.26616	2	3	7
-CKKL	0.26616	2	3	21
-CKMR	0.26616	2	3	33
-CKWD	0.26616	2	3	5
-CKYP	0.26616	2	3	19
-CLFH	0.26616	2	3	17
-CLLF	0.26616	2	3	39
-CLMN	0.26616	2	3	26
-CLQP	0.26616	2	3	18
-CLWA	0.26616	2	3	20
-CLWL	0.26616	4	6	57
-CLWT	0.26616	2	3	36
-CLYY	0.26616	2	3	24

-CMAE	0.26616	2	3	13
-CMDA	0.26616	2	3	14
-CMDL	0.26616	2	3	12
-CMEG	0.26616	2	3	18
-CMFI	0.26616	2	3	11
-CMFS	0.26616	4	6	18
-CMIP	0.26616	2	3	12
-CMKE	0.26616	2	3	7
-CMRN	0.26616	4	6	17
-CMSY	0.26616	2	3	12
-CNCL	0.26616	2	3	19
-CNFK	0.26616	2	3	16
-CNFN	0.26616	2	3	8
-CNIP	0.26616	8	12	15
-CNPI	0.26616	2	3	8
-CNPW	0.26616	2	3	12
-CNYT	0.26616	2	3	15
-CPCL	0.26616	4	6	37
-CPDD	0.26616	2	3	10
-CPDY	0.26616	2	3	5
-CPIQ	0.26616	2	3	13
-CPMH	0.26616	2	3	11
-CQEG	0.26616	2	3	11
-CQKV	0.26616	2	3	12
-CQND	0.26616	2	3	6
-CQTA	0.26616	4	6	13
-CQTQ	0.26616	2	3	10
-CQYN	0.26616	2	3	8
-CRCG	0.26616	2	3	13
-CRFC	0.26616	2	3	19
-CRGF	0.26616	2	3	19
-CRGI	0.26616	4	6	21
-CRGM	0.26616	2	3	12
-CRKW	0.26616	2	3	24
-CRPM	0.26616	2	3	21
-CRWD	0.26616	2	3	10
-CRYH	0.26616	2	3	15
-CRYK	0.26616	2	3	17
-CSAH	0.26616	2	3	26
-CSQA	0.26616	2	3	20
-CSSA	0.26616	16	24	62
-CSTF	0.26616	2	3	29
-CSTQ	0.26616	2	3	43
-CSVY	0.26616	4	6	24
-CSWK	0.26616	2	3	26
-CTCE	0.26616	2	3	21
-CTEH	0.26616	2	3	15
-CTFH	0.26616	2	3	20
-CTFN	0.26616	2	3	6
-CTFY	0.26616	2	3	11
-CTMN	0.26616	2	3	20
-CTNM	0.26616	2	3	14
-CTPW	0.26616	2	3	27
-CTQA	0.26616	2	3	12
-CTQD	0.26616	2	3	10
-CTQR	0.26616	4	6	31
-CTSQ	0.26616	2	3	41

-CTWD	0.26616	2	3	5
-CTWF	0.26616	2	3	9
-CTYT	0.26616	4	6	16
-CVAK	0.26616	8	12	27
-CVFN	0.26616	2	3	15
-CVFS	0.26616	4	6	39
-CVKF	0.26616	2	3	4
-CVKW	0.26616	4	6	24
-CVNW	0.26616	2	3	24
-CVPQ	0.26616	2	3	23
-CVPY	0.26616	2	3	13
-CVYF	0.26616	2	3	21
-CVYI	0.26616	2	3	14
-CVYR	0.26616	2	3	21
-CWED	0.26616	2	3	7
-CWHL	0.26616	2	3	17
-CWHT	0.26616	2	3	21
-CWIL	0.26616	2	3	15
-CWMR	0.26616	2	3	18
-CWNP	0.26616	2	3	14
-CWQK	0.26616	2	3	17
-CWRP	0.26616	2	3	16
-CWRW	0.26616	2	3	14
-CWTC	0.26616	4	6	7
-CWYD	0.26616	2	3	25
-CWYE	0.26616	2	3	12
-CWYR	0.26616	2	3	12
-CYCF	0.26616	2	3	9
-CYCI	0.26616	2	3	18
-CYDE	0.26616	2	3	8
-CYDM	0.26616	2	3	8
-CYDY	0.26616	2	3	14
-CYHI	0.26616	2	3	15
-CYHM	0.26616	2	3	11
-CYMD	0.26616	2	3	14
-CYPK	0.26616	2	3	20
-CYPW	0.26616	2	3	14
-CYTM	0.26616	2	3	15
-CYTQ	0.26616	2	3	16
-CYTW	0.26616	2	3	21
-CYVT	0.26616	4	6	31
-CAMV	0.26616	10	15	13
-CTAD	0.26616	6	9	24
-CTVP	0.26616	6	9	52
-CTYD	0.26616	6	9	22
-CDRR	0.27149	17	25	61
-CRVT	0.27317	13	19	43
-CEVV	0.27448	11	16	20
-CCLP	0.2764	9	13	36
-CYLR	0.27947	7	10	35
-CHVL	0.28182	12	17	86
-CTSS	0.2828	34	48	99
-CALH	0.28518	5	7	45
-CDRC	0.28518	5	7	20
-CGPP	0.28518	5	7	37
-CLCP	0.28518	5	7	47
-CTMD	0.28518	5	7	18

-CTMT	0.28664	28	39	65
-CDRV	0.29036	8	11	28
-CPLN	0.29036	8	11	47
-CRLL	0.29036	16	22	130
-CSYD	0.29036	8	11	21
-CVPV	0.29036	8	11	32
-CHLL	0.29418	14	19	60
-CGVS	0.29943	9	12	38
-CACA	0.29943	3	4	15
-CAID	0.29943	3	4	16
-CAYM	0.29943	6	8	27
-CCRK	0.29943	3	4	21
-CDGD	0.29943	3	4	15
-CDKP	0.29943	3	4	9
-CDVW	0.29943	3	4	10
-CFTF	0.29943	3	4	26
-CGHV	0.29943	3	4	24
-CGPI	0.29943	3	4	34
-CGRC	0.29943	3	4	30
-CGTD	0.29943	3	4	7
-CHLH	0.29943	3	4	34
-CHTK	0.29943	3	4	27
-CILY	0.29943	3	4	22
-CKAC	0.29943	3	4	14
-CKRN	0.29943	3	4	12
-CLQN	0.29943	3	4	36
-CMEM	0.29943	3	4	12
-CMLL	0.29943	3	4	36
-CMSW	0.29943	3	4	25
-CMYH	0.29943	3	4	12
-CNRM	0.29943	3	4	30
-CPME	0.29943	3	4	15
-CPND	0.29943	3	4	9
-CPPC	0.29943	3	4	18
-CQFI	0.29943	3	4	11
-CRHP	0.29943	3	4	33
-CRPW	0.29943	3	4	26
-CSCG	0.29943	3	4	12
-CSED	0.29943	6	8	31
-CTEY	0.29943	3	4	9
-CTFI	0.29943	3	4	22
-CTWS	0.29943	3	4	38
-CVAW	0.29943	3	4	30
-CVLH	0.29943	3	4	15
-CYCT	0.29943	3	4	14
-CYKL	0.29943	3	4	26
-CYLL	0.29943	12	16	68
-CYLN	0.29943	3	4	30
-CYRH	0.29943	3	4	20
-CMNR	0.30711	10	13	33
-CSIG	0.30711	10	13	37
-CRDD	0.31052	7	9	22
-CACV	0.3194	4	5	9
-CAVH	0.3194	4	5	37
-CCLL	0.3194	4	5	41
-CCMT	0.3194	4	5	29
-CCRV	0.3194	4	5	21

-CDHC	0.3194	4	5	18
-CDWD	0.3194	4	5	9
-CEGE	0.3194	4	5	3
-CFES	0.3194	8	10	19
-CFSQ	0.3194	4	5	17
-CHDA	0.3194	4	5	17
-CHRG	0.3194	4	5	16
-CHVI	0.3194	4	5	20
-CHVP	0.3194	8	10	40
-CICR	0.3194	4	5	11
-CILL	0.3194	4	5	45
-CIRG	0.3194	4	5	34
-CMIR	0.3194	4	5	22
-CMTQ	0.3194	4	5	16
-CNDL	0.3194	4	5	19
-CPLF	0.3194	4	5	28
-CRCA	0.3194	8	10	42
-CRLH	0.3194	4	5	25
-CSCN	0.3194	4	5	28
-CYVD	0.3194	4	5	19
-CLLP	0.32439	13	16	67
-CDSD	0.32666	9	11	23
-CVQS	0.32666	9	11	27
-CCLR	0.3327	5	6	66
-CDRA	0.3327	10	12	47
-CHHR	0.3327	5	6	20
-CHLT	0.3327	5	6	51
-CICY	0.3327	5	6	5
-CIPC	0.3327	5	6	10
-CIVE	0.3327	5	6	17
-CLHN	0.3327	5	6	25
-CNED	0.3327	5	6	13
-CPPP	0.3327	5	6	15
-CVIP	0.3327	5	6	27
-CVNA	0.3327	5	6	31
-CACC	0.34221	6	7	9
-CCIE	0.34221	6	7	14
-CDCI	0.34221	6	7	6
-CHNS	0.34221	6	7	25
-CKIL	0.34221	6	7	25
-CPVA	0.34221	6	7	40
-CTMP	0.34221	6	7	31
-CYSD	0.34221	6	7	18
-CCVP	0.34934	7	8	30
-CDLC	0.34934	7	8	20
-CDPS	0.34934	14	16	31
-CGRR	0.34934	7	8	31
-CRHA	0.34934	7	8	27
-CGIL	0.35488	8	9	26
-CILI	0.35488	8	9	32
-CTPC	0.35488	8	9	10
-CDHP	0.35932	9	10	20
-CSTM	0.35932	9	10	25
-CSCR	0.36295	10	11	47
-CSMI	0.36295	10	11	23
-CTMR	0.36295	10	11	50
-CLMT	0.37263	14	15	35

-CDDL	0.37576	16	17	41
-CNLA	0.38189	22	23	47
-CDVA	0.39925	9	9	13
-CPSC	0.39925	9	9	15
-CAAK	0.39925	2	2	16
-CAAM	0.39925	2	2	40
-CAAQ	0.39925	1	1	18
-CACG	0.39925	2	2	12
-CACH	0.39925	2	2	17
-CACW	0.39925	2	2	17
-CACY	0.39925	1	1	6
-CADM	0.39925	1	1	6
-CADN	0.39925	1	1	16
-CADY	0.39925	1	1	11
-CAED	0.39925	1	1	8
-CAFF	0.39925	2	2	7
-CAFI	0.39925	2	2	11
-CAFW	0.39925	1	1	11
-CAGM	0.39925	2	2	20
-CAGQ	0.39925	2	2	11
-CAGW	0.39925	2	2	19
-CAHH	0.39925	1	1	4
-CAHL	0.39925	3	3	28
-CAHQ	0.39925	1	1	9
-CAIW	0.39925	1	1	15
-CANH	0.39925	1	1	9
-CANN	0.39925	1	1	11
-CANP	0.39925	1	1	14
-CAPC	0.39925	1	1	9
-CAPD	0.39925	2	2	10
-CAPH	0.39925	2	2	6
-CAPM	0.39925	1	1	20
-CAPQ	0.39925	1	1	12
-CAPT	0.39925	2	2	30
-CAQC	0.39925	1	1	6
-CAQH	0.39925	1	1	10
-CAQI	0.39925	3	3	4
-CAQM	0.39925	1	1	16
-CAQP	0.39925	1	1	9
-CAQQ	0.39925	1	1	5
-CAQT	0.39925	1	1	26
-CASQ	0.39925	3	3	37
-CATH	0.39925	1	1	16
-CAVN	0.39925	15	15	34
-CAWH	0.39925	1	1	10
-CAWV	0.39925	1	1	19
-CAYC	0.39925	1	1	9
-CAYF	0.39925	2	2	13
-CAYH	0.39925	3	3	11
-CAYQ	0.39925	1	1	9
-CAYW	0.39925	1	1	16
-CCAA	0.39925	1	1	23
-CCAC	0.39925	1	1	9
-CCAH	0.39925	1	1	5
-CCCE	0.39925	1	1	7
-CCCG	0.39925	1	1	4
-CCCH	0.39925	2	2	9

-CCCI	0.39925	1	1	2
-CCCK	0.39925	1	1	12
-CCCW	0.39925	1	1	9
-CCCY	0.39925	2	2	9
-CCDA	0.39925	6	6	11
-CCDC	0.39925	1	1	5
-CCDF	0.39925	2	2	2
-CCDN	0.39925	1	1	17
-CCEA	0.39925	1	1	4
-CCEG	0.39925	1	1	7
-CCEM	0.39925	1	1	2
-CCFD	0.39925	1	1	1
-CCFG	0.39925	1	1	13
-CCFH	0.39925	1	1	5
-CCFK	0.39925	2	2	8
-CCFN	0.39925	1	1	3
-CCFW	0.39925	1	1	15
-CCGH	0.39925	1	1	7
-CCGI	0.39925	1	1	6
-CCGQ	0.39925	2	2	6
-CCGW	0.39925	1	1	14
-CCHM	0.39925	1	1	3
-CCHV	0.39925	3	3	11
-CCIK	0.39925	1	1	8
-CCIW	0.39925	1	1	7
-CCKC	0.39925	1	1	8
-CCKW	0.39925	1	1	7
-CCKY	0.39925	1	1	7
-CCLW	0.39925	1	1	9
-CCMF	0.39925	2	2	9
-CCMI	0.39925	2	2	6
-CCMN	0.39925	1	1	4
-CCNF	0.39925	1	1	1
-CCNG	0.39925	1	1	5
-CCNI	0.39925	1	1	6
-CCNM	0.39925	1	1	7
-CCNR	0.39925	3	3	16
-CCNW	0.39925	1	1	4
-CCNY	0.39925	1	1	1
-CCPH	0.39925	1	1	7
-CCQE	0.39925	1	1	3
-CCQF	0.39925	1	1	5
-CCQG	0.39925	1	1	8
-CCQH	0.39925	2	2	15
-CCQN	0.39925	1	1	6
-CCQR	0.39925	2	2	15
-CCQW	0.39925	1	1	7
-CCRY	0.39925	2	2	21
-CCSD	0.39925	4	4	13
-CCSF	0.39925	1	1	20
-CCTH	0.39925	1	1	7
-CCVE	0.39925	1	1	7
-CCVI	0.39925	6	6	15
-CCWF	0.39925	1	1	4
-CCWK	0.39925	1	1	6
-CCWL	0.39925	1	1	7
-CCWM	0.39925	1	1	7

-CCWP	0.39925	1	1	7
-CCWS	0.39925	1	1	18
-CCWT	0.39925	1	1	5
-CCWV	0.39925	1	1	5
-CCWW	0.39925	1	1	5
-CCWY	0.39925	1	1	16
-CCYH	0.39925	1	1	2
-CCYI	0.39925	1	1	7
-CCYN	0.39925	1	1	8
-CDAW	0.39925	2	2	13
-CDCC	0.39925	1	1	6
-CDCW	0.39925	1	1	5
-CDDP	0.39925	4	4	10
-CDDW	0.39925	2	2	7
-CDEG	0.39925	1	1	6
-CDEN	0.39925	2	2	12
-CDFE	0.39925	1	1	2
-CDFI	0.39925	1	1	1
-CDFW	0.39925	1	1	10
-CDFY	0.39925	1	1	6
-CDHI	0.39925	1	1	3
-CDHW	0.39925	1	1	13
-CDIY	0.39925	1	1	5
-CDLH	0.39925	6	6	24
-CDLW	0.39925	1	1	20
-CDMI	0.39925	2	2	3
-CDMP	0.39925	2	2	18
-CDNI	0.39925	1	1	7
-CDPH	0.39925	3	3	11
-CDQA	0.39925	1	1	8
-CDQE	0.39925	1	1	2
-CDTA	0.39925	1	1	6
-CDTK	0.39925	3	3	14
-CDVK	0.39925	2	2	17
-CDWC	0.39925	1	1	1
-CDWF	0.39925	1	1	2
-CDWH	0.39925	1	1	6
-CDWI	0.39925	1	1	5
-CDWK	0.39925	1	1	10
-CDWP	0.39925	2	2	11
-CDWT	0.39925	2	2	18
-CDWY	0.39925	1	1	8
-CDYC	0.39925	6	6	7
-CDYF	0.39925	1	1	16
-CDYH	0.39925	1	1	14
-CDYI	0.39925	1	1	8
-CDYW	0.39925	1	1	2
-CDYY	0.39925	2	2	9
-CEAF	0.39925	1	1	6
-CECM	0.39925	3	3	9
-CECP	0.39925	3	3	13
-CEDC	0.39925	1	1	6
-CEDE	0.39925	1	1	1
-CEDF	0.39925	1	1	5
-CEDM	0.39925	1	1	4
-CEEF	0.39925	2	2	5
-CEEG	0.39925	1	1	7

-CEEI	0.39925	1	1	3
-CEEQ	0.39925	1	1	4
-CEFA	0.39925	1	1	6
-CEFF	0.39925	1	1	6
-CEFQ	0.39925	1	1	4
-CEFV	0.39925	1	1	5
-CEGM	0.39925	1	1	2
-CEGW	0.39925	1	1	5
-CEHC	0.39925	1	1	4
-CEHH	0.39925	1	1	3
-CEHQ	0.39925	1	1	5
-CEHY	0.39925	1	1	6
-CEID	0.39925	1	1	5
-CEKI	0.39925	1	1	4
-CEKK	0.39925	1	1	2
-CEKY	0.39925	1	1	5
-CELQ	0.39925	2	2	20
-CEME	0.39925	1	1	6
-CEMK	0.39925	1	1	2
-CEMS	0.39925	2	2	17
-CENY	0.39925	2	2	8
-CEPE	0.39925	1	1	12
-CEPI	0.39925	1	1	5
-CEPK	0.39925	1	1	11
-CEQD	0.39925	1	1	6
-CEQE	0.39925	2	2	9
-CEQF	0.39925	1	1	4
-CEQH	0.39925	1	1	9
-CEQM	0.39925	1	1	7
-CEQP	0.39925	1	1	8
-CEQQ	0.39925	1	1	4
-CETM	0.39925	1	1	23
-CETQ	0.39925	1	1	8
-CEVF	0.39925	1	1	8
-CEVQ	0.39925	1	1	15
-CEWC	0.39925	1	1	7
-CEWF	0.39925	1	1	4
-CEWH	0.39925	1	1	2
-CEWI	0.39925	1	1	2
-CEWP	0.39925	2	2	20
-CEWS	0.39925	3	3	5
-CEWT	0.39925	2	2	18
-CEWW	0.39925	1	1	3
-CEWY	0.39925	1	1	8
-CEYC	0.39925	1	1	12
-CEYK	0.39925	1	1	5
-CEYR	0.39925	3	3	12
-CFAR	0.39925	2	2	17
-CFAW	0.39925	1	1	9
-CFCF	0.39925	1	1	8
-CFCK	0.39925	2	2	6
-CFCN	0.39925	1	1	8
-CFDH	0.39925	1	1	9
-CFDI	0.39925	1	1	9
-CFDW	0.39925	1	1	3
-CFEC	0.39925	1	1	5
-CFEF	0.39925	1	1	3

-CFEW	0.39925	1	1	2
-CFFC	0.39925	1	1	9
-CFFD	0.39925	1	1	5
-CFFF	0.39925	1	1	7
-CFFN	0.39925	1	1	9
-CFFQ	0.39925	1	1	3
-CFFT	0.39925	2	2	13
-CFFW	0.39925	1	1	2
-CFFY	0.39925	1	1	4
-CFGG	0.39925	3	3	15
-CFHA	0.39925	1	1	14
-CFHD	0.39925	1	1	11
-CFHH	0.39925	1	1	5
-CFHI	0.39925	1	1	5
-CFHK	0.39925	1	1	3
-CFHL	0.39925	1	1	17
-CFHM	0.39925	1	1	15
-CFHN	0.39925	1	1	8
-CFHT	0.39925	1	1	12
-CFIE	0.39925	1	1	11
-CFIH	0.39925	1	1	3
-CFIK	0.39925	1	1	8
-CFIW	0.39925	1	1	11
-CFKC	0.39925	1	1	6
-CFKE	0.39925	1	1	4
-CFKH	0.39925	1	1	6
-CFKI	0.39925	1	1	4
-CFKM	0.39925	1	1	7
-CFKQ	0.39925	2	2	3
-CFKY	0.39925	1	1	11
-CFLA	0.39925	3	3	26
-CFLD	0.39925	1	1	12
-CFLF	0.39925	2	2	8
-CFLI	0.39925	2	2	11
-CFME	0.39925	2	2	14
-CFMF	0.39925	1	1	4
-CFMR	0.39925	2	2	21
-CFMV	0.39925	1	1	15
-CFMW	0.39925	1	1	5
-CFNC	0.39925	1	1	9
-CFNI	0.39925	1	1	15
-CFNK	0.39925	2	2	8
-CFNP	0.39925	1	1	11
-CFPF	0.39925	1	1	15
-CFPW	0.39925	1	1	3
-CFQD	0.39925	1	1	6
-CFQF	0.39925	1	1	6
-CFRH	0.39925	2	2	16
-CFSY	0.39925	1	1	13
-CFTP	0.39925	1	1	16
-CFVT	0.39925	3	3	12
-CFWC	0.39925	1	1	9
-CFWE	0.39925	1	1	4
-CFWF	0.39925	1	1	3
-CFWG	0.39925	1	1	5
-CFWH	0.39925	1	1	5
-CFWK	0.39925	1	1	3

-CFWM	0.39925	1	1	11
-CFWT	0.39925	2	2	21
-CFWV	0.39925	1	1	9
-CFWW	0.39925	1	1	5
-CFWY	0.39925	1	1	5
-CFYA	0.39925	1	1	8
-CFYC	0.39925	1	1	3
-CFYD	0.39925	1	1	6
-CFYF	0.39925	1	1	6
-CFYG	0.39925	1	1	6
-CFYH	0.39925	1	1	9
-CFYI	0.39925	1	1	17
-CFYL	0.39925	2	2	21
-CFYP	0.39925	1	1	7
-CFYW	0.39925	1	1	8
-CGAG	0.39925	3	3	8
-CGAH	0.39925	1	1	7
-CGCW	0.39925	1	1	8
-CGDI	0.39925	2	2	8
-CGEC	0.39925	1	1	12
-CGEF	0.39925	2	2	24
-CGFC	0.39925	1	1	7
-CGFE	0.39925	1	1	7
-CGFL	0.39925	2	2	22
-CGFM	0.39925	2	2	11
-CGFN	0.39925	1	1	2
-CGFW	0.39925	1	1	6
-CGFY	0.39925	1	1	8
-CGGC	0.39925	1	1	5
-CGGW	0.39925	1	1	18
-CGHE	0.39925	1	1	8
-CGHF	0.39925	1	1	1
-CGHW	0.39925	1	1	2
-CGKN	0.39925	2	2	13
-CGMF	0.39925	1	1	10
-CGPQ	0.39925	1	1	7
-CGQD	0.39925	1	1	8
-CGQF	0.39925	1	1	6
-CGQQ	0.39925	1	1	10
-CGQY	0.39925	1	1	5
-CGVN	0.39925	2	2	11
-CGWC	0.39925	1	1	11
-CGWF	0.39925	1	1	9
-CGWH	0.39925	1	1	6
-CGWI	0.39925	1	1	6
-CGWM	0.39925	1	1	6
-CGWN	0.39925	2	2	6
-CGWP	0.39925	1	1	23
-CGWW	0.39925	1	1	7
-CGYC	0.39925	2	2	5
-CGYQ	0.39925	1	1	13
-CGYT	0.39925	4	4	23
-CGYW	0.39925	1	1	6
-CHAC	0.39925	2	2	18
-CHAM	0.39925	1	1	17
-CHAS	0.39925	4	4	33
-CHCA	0.39925	1	1	5

-CHCC	0.39925	1	1	6
-CHCF	0.39925	1	1	6
-CHCG	0.39925	1	1	4
-CHCN	0.39925	1	1	9
-CHCS	0.39925	2	2	14
-CHCT	0.39925	1	1	16
-CHDD	0.39925	2	2	6
-CHDG	0.39925	1	1	7
-CHDM	0.39925	1	1	19
-CHDW	0.39925	2	2	11
-CHEK	0.39925	1	1	8
-CHFD	0.39925	1	1	7
-CHFF	0.39925	1	1	3
-CHFN	0.39925	1	1	3
-CHFQ	0.39925	1	1	8
-CHFT	0.39925	2	2	9
-CHFY	0.39925	1	1	5
-CHGM	0.39925	3	3	4
-CHHC	0.39925	1	1	6
-CHHF	0.39925	1	1	3
-CHHK	0.39925	1	1	4
-CHHM	0.39925	1	1	12
-CHHW	0.39925	1	1	3
-CHIH	0.39925	2	2	14
-CHIP	0.39925	1	1	3
-CHIW	0.39925	1	1	10
-CHKC	0.39925	2	2	3
-CHKD	0.39925	1	1	2
-CHKI	0.39925	1	1	10
-CHKM	0.39925	1	1	3
-CHKN	0.39925	2	2	8
-CHKW	0.39925	1	1	9
-CHLF	0.39925	2	2	15
-CHLY	0.39925	1	1	12
-CHME	0.39925	1	1	10
-CHMF	0.39925	1	1	8
-CHMG	0.39925	3	3	17
-CHMK	0.39925	1	1	15
-CHML	0.39925	2	2	32
-CHMN	0.39925	1	1	9
-CHMQ	0.39925	1	1	14
-CHMW	0.39925	1	1	2
-CHNE	0.39925	2	2	15
-CHNF	0.39925	1	1	4
-CHNH	0.39925	1	1	9
-CHNI	0.39925	1	1	9
-CHNK	0.39925	1	1	18
-CHNQ	0.39925	1	1	15
-CHNW	0.39925	1	1	5
-CHPD	0.39925	2	2	7
-CHPF	0.39925	1	1	11
-CHPI	0.39925	2	2	23
-CHPM	0.39925	1	1	12
-CHPW	0.39925	1	1	13
-CHQC	0.39925	1	1	6
-CHQE	0.39925	2	2	17
-CHQM	0.39925	1	1	16

-CHQP	0.39925	1	1	4
-CHQQ	0.39925	1	1	9
-CHQT	0.39925	2	2	13
-CHQW	0.39925	1	1	13
-CHRC	0.39925	2	2	22
-CHRD	0.39925	8	8	10
-CHRY	0.39925	1	1	14
-CHTH	0.39925	1	1	12
-CHTM	0.39925	2	2	28
-CHVF	0.39925	1	1	13
-CHVN	0.39925	2	2	14
-CHWK	0.39925	1	1	6
-CHWQ	0.39925	1	1	5
-CHWW	0.39925	1	1	7
-CHWY	0.39925	1	1	4
-CHYA	0.39925	2	2	17
-CHYD	0.39925	1	1	16
-CHYF	0.39925	1	1	3
-CHYI	0.39925	1	1	7
-CHYM	0.39925	1	1	13
-CHYN	0.39925	1	1	11
-CHYQ	0.39925	1	1	3
-CHYW	0.39925	1	1	2
-CHYY	0.39925	1	1	1
-CIAF	0.39925	1	1	2
-CIAN	0.39925	1	1	8
-CIAW	0.39925	1	1	11
-CICD	0.39925	1	1	1
-CICE	0.39925	1	1	4
-CICI	0.39925	2	2	11
-CICP	0.39925	1	1	9
-CICW	0.39925	1	1	7
-CIDF	0.39925	1	1	17
-CIEC	0.39925	1	1	6
-CIED	0.39925	1	1	3
-CIEF	0.39925	1	1	2
-CIEQ	0.39925	1	1	15
-CIEW	0.39925	1	1	5
-CIEY	0.39925	1	1	7
-CIFA	0.39925	2	2	10
-CIFE	0.39925	1	1	3
-CIFF	0.39925	1	1	12
-CIFI	0.39925	1	1	7
-CIFN	0.39925	2	2	5
-CIFP	0.39925	1	1	5
-CIFW	0.39925	1	1	8
-CIGW	0.39925	1	1	10
-CIHF	0.39925	1	1	9
-CIHM	0.39925	3	3	7
-CIHP	0.39925	2	2	10
-CIHQ	0.39925	1	1	9
-CIHV	0.39925	3	3	11
-CIIR	0.39925	4	4	15
-CIKG	0.39925	1	1	13
-CIKI	0.39925	1	1	4
-CIKK	0.39925	2	2	10
-CIMI	0.39925	1	1	2

-CINC	0.39925	1	1	5
-CINE	0.39925	2	2	8
-CINP	0.39925	1	1	5
-CIPH	0.39925	2	2	17
-CIPQ	0.39925	1	1	7
-CIQH	0.39925	1	1	10
-CIQP	0.39925	1	1	8
-CIQR	0.39925	1	1	14
-CIRD	0.39925	2	2	8
-CIRF	0.39925	1	1	11
-CISF	0.39925	4	4	23
-CISN	0.39925	2	2	16
-CITY	0.39925	1	1	28
-CIVW	0.39925	1	1	5
-CIWC	0.39925	1	1	1
-CIWE	0.39925	1	1	6
-CIWF	0.39925	1	1	13
-CIWH	0.39925	1	1	2
-CIWI	0.39925	1	1	6
-CIWM	0.39925	1	1	4
-CIWN	0.39925	1	1	14
-CIWW	0.39925	1	1	9
-CIWY	0.39925	1	1	9
-CIYA	0.39925	1	1	11
-CIYF	0.39925	1	1	4
-CIYI	0.39925	1	1	7
-CIYP	0.39925	1	1	1
-CIYS	0.39925	1	1	21
-CIYW	0.39925	1	1	6
-CKCE	0.39925	1	1	5
-CKCN	0.39925	2	2	9
-CKCS	0.39925	2	2	8
-CKCT	0.39925	1	1	9
-CKDC	0.39925	1	1	4
-CKDD	0.39925	1	1	7
-CKDK	0.39925	1	1	3
-CKDW	0.39925	1	1	14
-CKDY	0.39925	1	1	2
-CKEA	0.39925	1	1	2
-CKEW	0.39925	1	1	4
-CKFE	0.39925	2	2	7
-CKFF	0.39925	1	1	2
-CKFI	0.39925	1	1	3
-CKFS	0.39925	1	1	26
-CKFV	0.39925	1	1	13
-CKFW	0.39925	2	2	6
-CKGN	0.39925	3	3	16
-CKHD	0.39925	1	1	2
-CKHH	0.39925	1	1	7
-CKHL	0.39925	1	1	18
-CKHM	0.39925	1	1	13
-CKHY	0.39925	1	1	5
-CKIF	0.39925	1	1	3
-CKIW	0.39925	1	1	5
-CKKW	0.39925	1	1	6
-CKLA	0.39925	12	12	24
-CKMW	0.39925	1	1	3

-CKMY	0.39925	1	1	14
-CKNI	0.39925	1	1	3
-CKNK	0.39925	1	1	12
-CKPA	0.39925	2	2	15
-CKPC	0.39925	2	2	7
-CKPQ	0.39925	1	1	13
-CKPV	0.39925	1	1	19
-CKQH	0.39925	1	1	5
-CKQP	0.39925	1	1	2
-CKQQ	0.39925	1	1	8
-CKQR	0.39925	1	1	20
-CKQY	0.39925	1	1	4
-CKRW	0.39925	2	2	13
-CKTA	0.39925	12	12	23
-CKTM	0.39925	1	1	16
-CKVD	0.39925	2	2	7
-CKWA	0.39925	1	1	19
-CKWC	0.39925	1	1	11
-CKWE	0.39925	1	1	8
-CKWF	0.39925	1	1	5
-CKWI	0.39925	1	1	2
-CKWK	0.39925	1	1	12
-CKWQ	0.39925	2	2	3
-CKWR	0.39925	1	1	8
-CKWY	0.39925	1	1	7
-CKYC	0.39925	1	1	18
-CKYG	0.39925	1	1	8
-CKYI	0.39925	1	1	3
-CKYN	0.39925	2	2	7
-CKYQ	0.39925	1	1	5
-CKYW	0.39925	1	1	12
-CKYY	0.39925	1	1	15
-CLFF	0.39925	1	1	16
-CLFG	0.39925	2	2	9
-CLFV	0.39925	4	4	21
-CLFW	0.39925	1	1	21
-CLFY	0.39925	1	1	11
-CLHC	0.39925	1	1	17
-CLHK	0.39925	2	2	16
-CLIE	0.39925	2	2	5
-CLMH	0.39925	3	3	8
-CLWD	0.39925	2	2	19
-CLWH	0.39925	1	1	27
-CLWM	0.39925	1	1	31
-CLWR	0.39925	2	2	31
-CLWW	0.39925	1	1	17
-CLWY	0.39925	1	1	7
-CLYQ	0.39925	2	2	18
-CMAA	0.39925	1	1	10
-CMCC	0.39925	1	1	2
-CMCI	0.39925	1	1	4
-CMCK	0.39925	1	1	2
-CMCN	0.39925	1	1	8
-CMCT	0.39925	1	1	7
-CMCW	0.39925	1	1	5
-CMCY	0.39925	1	1	14
-CMDC	0.39925	1	1	1

-CMDE	0.39925	1	1	1
-CMDF	0.39925	1	1	4
-CMDW	0.39925	1	1	2
-CMDY	0.39925	1	1	8
-CMEA	0.39925	1	1	6
-CMEC	0.39925	1	1	8
-CMEI	0.39925	1	1	8
-CMEW	0.39925	1	1	10
-CMEY	0.39925	1	1	16
-CMFC	0.39925	1	1	3
-CMFD	0.39925	1	1	3
-CMFF	0.39925	1	1	4
-CMFH	0.39925	1	1	5
-CMFK	0.39925	1	1	10
-CMFL	0.39925	2	2	31
-CMFN	0.39925	3	3	13
-CMFP	0.39925	1	1	6
-CMFY	0.39925	1	1	12
-CMGD	0.39925	2	2	8
-CMGW	0.39925	1	1	7
-CMHC	0.39925	1	1	8
-CMHF	0.39925	1	1	6
-CMHG	0.39925	1	1	11
-CMHH	0.39925	1	1	2
-CMHQ	0.39925	1	1	7
-CMKF	0.39925	1	1	5
-CMKH	0.39925	1	1	3
-CMKV	0.39925	1	1	10
-CMKW	0.39925	2	2	11
-CMME	0.39925	1	1	2
-CMMI	0.39925	1	1	8
-CMMN	0.39925	1	1	9
-CMMV	0.39925	1	1	11
-CMMW	0.39925	1	1	2
-CMMY	0.39925	1	1	5
-CMNF	0.39925	2	2	7
-CMNQ	0.39925	2	2	10
-CMNY	0.39925	1	1	10
-CMPG	0.39925	1	1	5
-CMPW	0.39925	1	1	17
-CMQC	0.39925	1	1	9
-CMQF	0.39925	1	1	8
-CMQH	0.39925	1	1	6
-CMQM	0.39925	1	1	4
-CMQN	0.39925	1	1	9
-CMQP	0.39925	1	1	2
-CMQQ	0.39925	1	1	6
-CMQY	0.39925	1	1	4
-CMRF	0.39925	1	1	11
-CMRM	0.39925	1	1	7
-CMSC	0.39925	3	3	17
-CMSF	0.39925	1	1	14
-CMTM	0.39925	3	3	21
-CMVN	0.39925	7	7	16
-CMVP	0.39925	2	2	19
-CMWC	0.39925	1	1	7
-CMWG	0.39925	1	1	12

-CMWH	0.39925	1	1	10
-CMWI	0.39925	1	1	9
-CMWL	0.39925	1	1	16
-CMWM	0.39925	1	1	8
-CMWN	0.39925	1	1	11
-CMWP	0.39925	1	1	17
-CMWQ	0.39925	4	4	7
-CMWW	0.39925	1	1	3
-CMWY	0.39925	1	1	6
-CMYC	0.39925	1	1	9
-CMYF	0.39925	1	1	3
-CMYM	0.39925	1	1	8
-CMYN	0.39925	1	1	12
-CMYR	0.39925	2	2	11
-CMYT	0.39925	2	2	14
-CMYW	0.39925	1	1	5
-CMYY	0.39925	1	1	11
-CNAK	0.39925	2	2	16
-CNAP	0.39925	2	2	10
-CNCA	0.39925	1	1	6
-CNCC	0.39925	1	1	5
-CNCD	0.39925	1	1	4
-CNCP	0.39925	3	3	6
-CNCR	0.39925	2	2	10
-CNCW	0.39925	1	1	6
-CNDC	0.39925	1	1	5
-CNDH	0.39925	4	4	6
-CNDI	0.39925	1	1	6
-CNEG	0.39925	2	2	9
-CNEN	0.39925	1	1	8
-CNFD	0.39925	1	1	2
-CNFQ	0.39925	1	1	5
-CNFY	0.39925	1	1	3
-CNGC	0.39925	1	1	10
-CNHH	0.39925	1	1	7
-CNIW	0.39925	1	1	8
-CNKE	0.39925	1	1	7
-CNMA	0.39925	2	2	15
-CNMH	0.39925	1	1	11
-CNMI	0.39925	1	1	5
-CNNE	0.39925	1	1	8
-CNNI	0.39925	1	1	6
-CNPY	0.39925	2	2	11
-CNQH	0.39925	1	1	5
-CNQI	0.39925	1	1	9
-CNQL	0.39925	1	1	30
-CNQQ	0.39925	1	1	6
-CNQT	0.39925	1	1	10
-CNRE	0.39925	1	1	23
-CNRH	0.39925	1	1	22
-CNVW	0.39925	1	1	17
-CNWC	0.39925	1	1	6
-CNWF	0.39925	1	1	4
-CNWI	0.39925	1	1	4
-CNYC	0.39925	1	1	6
-CNYQ	0.39925	1	1	3
-CNYW	0.39925	1	1	3

-CNYY	0.39925	1	1	17
-CPAQ	0.39925	1	1	7
-CPCC	0.39925	1	1	11
-CPCG	0.39925	3	3	16
-CPDH	0.39925	4	4	16
-CPFF	0.39925	1	1	6
-CPFT	0.39925	1	1	18
-CPFW	0.39925	1	1	12
-CPFY	0.39925	2	2	13
-CPHF	0.39925	1	1	6
-CPHQ	0.39925	1	1	16
-CPKI	0.39925	1	1	13
-CPLH	0.39925	1	1	26
-CPMF	0.39925	1	1	13
-CPMQ	0.39925	1	1	11
-CPPW	0.39925	1	1	4
-CPQD	0.39925	1	1	4
-CPQH	0.39925	2	2	15
-CPQM	0.39925	1	1	7
-CPQN	0.39925	1	1	11
-CPQQ	0.39925	1	1	12
-CPRH	0.39925	2	2	24
-CPRW	0.39925	1	1	18
-CPWA	0.39925	1	1	18
-CPWC	0.39925	1	1	2
-CPWD	0.39925	2	2	11
-CPWG	0.39925	1	1	18
-CPWH	0.39925	1	1	7
-CPWM	0.39925	2	2	18
-CPWN	0.39925	1	1	13
-CPYH	0.39925	1	1	11
-CPYI	0.39925	1	1	10
-CQAF	0.39925	1	1	12
-CQCC	0.39925	1	1	8
-CQCD	0.39925	1	1	2
-CQCH	0.39925	1	1	7
-CQCI	0.39925	3	3	10
-CQCK	0.39925	1	1	4
-CQCW	0.39925	1	1	3
-CQCY	0.39925	1	1	11
-CQDC	0.39925	1	1	3
-CQDF	0.39925	1	1	6
-CQDM	0.39925	1	1	6
-CQDN	0.39925	1	1	8
-CQDW	0.39925	1	1	5
-CQEA	0.39925	1	1	3
-CQEC	0.39925	1	1	1
-CQEF	0.39925	1	1	4
-CQEI	0.39925	1	1	7
-CQFF	0.39925	1	1	9
-CQFH	0.39925	1	1	5
-CQFL	0.39925	1	1	10
-CQFN	0.39925	1	1	6
-CQFQ	0.39925	1	1	14
-CQFT	0.39925	2	2	12
-CQFY	0.39925	1	1	11
-CQHA	0.39925	1	1	3

-CQHC	0.39925	1	1	4
-CQHE	0.39925	1	1	2
-CQHF	0.39925	1	1	4
-CQHG	0.39925	1	1	7
-CQHH	0.39925	2	2	6
-CQHI	0.39925	1	1	5
-CQHK	0.39925	1	1	6
-CQHN	0.39925	1	1	4
-CQHP	0.39925	1	1	6
-CQHQ	0.39925	1	1	11
-CQHW	0.39925	1	1	10
-CQHY	0.39925	1	1	10
-CQIW	0.39925	1	1	5
-CQKA	0.39925	1	1	8
-CQKC	0.39925	1	1	10
-CQKH	0.39925	2	2	2
-CQKQ	0.39925	1	1	6
-CQKW	0.39925	1	1	2
-CQLF	0.39925	2	2	16
-CQMY	0.39925	1	1	6
-CQNF	0.39925	1	1	2
-CQNK	0.39925	1	1	22
-CQNN	0.39925	1	1	3
-CQNW	0.39925	1	1	3
-CQPC	0.39925	1	1	2
-CQPD	0.39925	1	1	2
-CQPE	0.39925	1	1	4
-CQPF	0.39925	1	1	9
-CQPG	0.39925	1	1	6
-CQPH	0.39925	1	1	7
-CQPI	0.39925	1	1	17
-CQPK	0.39925	1	1	4
-CQPM	0.39925	1	1	7
-CQPN	0.39925	2	2	12
-CQPP	0.39925	1	1	10
-CQPQ	0.39925	1	1	11
-CQPR	0.39925	1	1	21
-CQPT	0.39925	1	1	11
-CQPV	0.39925	1	1	12
-CQPW	0.39925	1	1	12
-CQQA	0.39925	2	2	14
-CQQE	0.39925	1	1	1
-CQQF	0.39925	1	1	10
-CQQH	0.39925	1	1	10
-CQQK	0.39925	1	1	6
-CQQM	0.39925	1	1	4
-CQQP	0.39925	1	1	6
-CQQQ	0.39925	1	1	1
-CQQY	0.39925	1	1	3
-CQRW	0.39925	1	1	7
-CQSK	0.39925	1	1	8
-CQTH	0.39925	1	1	11
-CQTM	0.39925	1	1	40
-CQVF	0.39925	2	2	8
-CQVH	0.39925	1	1	6
-CQWA	0.39925	1	1	10
-CQWC	0.39925	1	1	2

-CQWD	0.39925	2	2	3
-CQWE	0.39925	1	1	5
-CQWF	0.39925	1	1	8
-CQWH	0.39925	1	1	5
-CQWI	0.39925	1	1	1
-CQWK	0.39925	1	1	7
-CQWM	0.39925	1	1	3
-CQWN	0.39925	1	1	10
-CQWQ	0.39925	2	2	11
-CQWR	0.39925	1	1	8
-CQWW	0.39925	1	1	5
-CQWY	0.39925	1	1	13
-CQYE	0.39925	1	1	3
-CQYM	0.39925	1	1	5
-CQYQ	0.39925	1	1	4
-CQYW	0.39925	1	1	3
-CQYY	0.39925	1	1	4
-CRAF	0.39925	1	1	16
-CRCH	0.39925	2	2	12
-CRCK	0.39925	2	2	15
-CRFA	0.39925	6	6	25
-CRFH	0.39925	2	2	15
-CRFV	0.39925	2	2	35
-CRHK	0.39925	2	2	32
-CRHM	0.39925	2	2	39
-CRHW	0.39925	1	1	7
-CRIY	0.39925	2	2	6
-CRKC	0.39925	1	1	12
-CRLI	0.39925	12	12	36
-CRMI	0.39925	6	6	19
-CRNK	0.39925	1	1	19
-CRNW	0.39925	1	1	10
-CRQF	0.39925	1	1	18
-CRQH	0.39925	1	1	13
-CRQP	0.39925	1	1	10
-CRSC	0.39925	15	15	40
-CRWA	0.39925	2	2	24
-CRWH	0.39925	1	1	15
-CRWI	0.39925	1	1	21
-CRYA	0.39925	6	6	32
-CRYI	0.39925	1	1	7
-CRYM	0.39925	1	1	26
-CRYW	0.39925	1	1	18
-CSCI	0.39925	2	2	12
-CSCW	0.39925	1	1	12
-CSFI	0.39925	1	1	16
-CSHQ	0.39925	2	2	21
-CSIW	0.39925	1	1	18
-CSMW	0.39925	1	1	17
-CSNQ	0.39925	1	1	9
-CSPM	0.39925	1	1	39
-CSQH	0.39925	2	2	21
-CSQL	0.39925	6	6	26
-CSQM	0.39925	2	2	25
-CSQP	0.39925	1	1	9
-CSSH	0.39925	4	4	19
-CSWA	0.39925	2	2	14

-CSWM	0.39925	1	1	11
-CSWR	0.39925	2	2	44
-CSYM	0.39925	2	2	30
-CTCK	0.39925	1	1	12
-CTCL	0.39925	8	8	44
-CTCN	0.39925	2	2	19
-CTCY	0.39925	2	2	19
-CTEM	0.39925	2	2	10
-CTHC	0.39925	2	2	23
-CTHH	0.39925	4	4	8
-CTHM	0.39925	1	1	11
-CTHP	0.39925	2	2	25
-CTIK	0.39925	2	2	18
-CTIW	0.39925	1	1	17
-CTLW	0.39925	1	1	47
-CTNY	0.39925	1	1	14
-CTPQ	0.39925	1	1	16
-CTQP	0.39925	1	1	7
-CTQY	0.39925	1	1	15
-CTRY	0.39925	2	2	24
-CTWH	0.39925	1	1	9
-CTWI	0.39925	1	1	4
-CTWN	0.39925	1	1	18
-CTWW	0.39925	1	1	6
-CTWY	0.39925	1	1	8
-CTYP	0.39925	1	1	21
-CTYY	0.39925	1	1	7
-CVDK	0.39925	1	1	2
-CVFH	0.39925	1	1	7
-CVFL	0.39925	2	2	48
-CVFQ	0.39925	1	1	20
-CVFW	0.39925	1	1	24
-CVFY	0.39925	1	1	12
-CVGQ	0.39925	2	2	11
-CVHC	0.39925	2	2	16
-CVHH	0.39925	1	1	8
-CVHY	0.39925	1	1	11
-CVKY	0.39925	3	3	14
-CVLY	0.39925	7	7	40
-CVNG	0.39925	4	4	21
-CVNM	0.39925	8	8	13
-CVNQ	0.39925	1	1	14
-CVQF	0.39925	1	1	10
-CVQH	0.39925	1	1	6
-CVQQ	0.39925	1	1	11
-CVSH	0.39925	2	2	12
-CVWF	0.39925	1	1	18
-CVWN	0.39925	1	1	17
-CVWY	0.39925	1	1	7
-CVYA	0.39925	1	1	26
-CVYC	0.39925	1	1	15
-CVYQ	0.39925	1	1	16
-CVYW	0.39925	1	1	15
-CWAC	0.39925	1	1	11
-CWAD	0.39925	2	2	6
-CWAF	0.39925	1	1	6
-CWAH	0.39925	1	1	3

-CWAQ	0.39925	6	6	3
-CWAV	0.39925	2	2	10
-CWAY	0.39925	2	2	11
-CWCC	0.39925	1	1	5
-CWCF	0.39925	1	1	6
-CWCG	0.39925	1	1	9
-CWCI	0.39925	1	1	3
-CWCN	0.39925	1	1	5
-CWCT	0.39925	1	1	19
-CWCW	0.39925	1	1	2
-CWCY	0.39925	1	1	3
-CWDA	0.39925	1	1	2
-CWDC	0.39925	1	1	5
-CWDD	0.39925	1	1	5
-CWDF	0.39925	1	1	7
-CWDN	0.39925	1	1	7
-CWDP	0.39925	1	1	3
-CWDV	0.39925	1	1	4
-CWDW	0.39925	1	1	2
-CWEE	0.39925	1	1	1
-CWEF	0.39925	2	2	9
-CWEI	0.39925	1	1	4
-CWEK	0.39925	1	1	6
-CWEQ	0.39925	1	1	3
-CWEY	0.39925	1	1	10
-CWFA	0.39925	1	1	9
-CWFC	0.39925	1	1	9
-CWFD	0.39925	1	1	12
-CWFE	0.39925	1	1	6
-CWFF	0.39925	1	1	3
-CWFI	0.39925	1	1	8
-CWFK	0.39925	2	2	5
-CWFP	0.39925	1	1	14
-CWFT	0.39925	1	1	10
-CWFV	0.39925	1	1	9
-CWFW	0.39925	1	1	4
-CWFY	0.39925	1	1	5
-CWGF	0.39925	1	1	4
-CWGH	0.39925	1	1	1
-CWGK	0.39925	1	1	6
-CWGM	0.39925	2	2	10
-CWGQ	0.39925	1	1	1
-CWHA	0.39925	1	1	9
-CWHD	0.39925	1	1	4
-CWHE	0.39925	1	1	4
-CWHF	0.39925	1	1	1
-CWHG	0.39925	1	1	16
-CWHH	0.39925	1	1	6
-CWHK	0.39925	1	1	8
-CWHM	0.39925	1	1	22
-CWHP	0.39925	2	2	13
-CWHR	0.39925	2	2	16
-CWIE	0.39925	1	1	9
-CWIH	0.39925	1	1	8
-CWII	0.39925	1	1	3
-CWIK	0.39925	1	1	7
-CWIN	0.39925	1	1	1

-CWIR	0.39925	1	1	13
-CWIW	0.39925	1	1	3
-CWIY	0.39925	1	1	11
-CWKF	0.39925	1	1	2
-CWKK	0.39925	1	1	4
-CWKL	0.39925	2	2	8
-CWKM	0.39925	1	1	7
-CWKQ	0.39925	1	1	2
-CWKR	0.39925	1	1	16
-CWKV	0.39925	1	1	4
-CWKW	0.39925	1	1	7
-CWLF	0.39925	1	1	17
-CWLV	0.39925	2	2	47
-CWLW	0.39925	1	1	13
-CWMA	0.39925	1	1	5
-CWMC	0.39925	2	2	6
-CWMD	0.39925	1	1	5
-CWMF	0.39925	1	1	2
-CWMH	0.39925	1	1	2
-CWMI	0.39925	1	1	7
-CWMS	0.39925	3	3	15
-CWMW	0.39925	1	1	9
-CWMY	0.39925	1	1	6
-CWNC	0.39925	1	1	3
-CWND	0.39925	1	1	7
-CWNH	0.39925	1	1	11
-CWNM	0.39925	1	1	1
-CWNN	0.39925	1	1	6
-CWNQ	0.39925	1	1	8
-CWNW	0.39925	1	1	5
-CWNY	0.39925	1	1	6
-CWPF	0.39925	1	1	6
-CWPP	0.39925	1	1	5
-CWPV	0.39925	1	1	15
-CWPW	0.39925	1	1	13
-CWPY	0.39925	1	1	20
-CWQF	0.39925	1	1	3
-CWQH	0.39925	1	1	4
-CWQI	0.39925	1	1	5
-CWQL	0.39925	1	1	13
-CWQP	0.39925	1	1	5
-CWQW	0.39925	1	1	8
-CWQY	0.39925	1	1	21
-CWRF	0.39925	1	1	8
-CWRG	0.39925	1	1	20
-CWRI	0.39925	1	1	8
-CWRM	0.39925	2	2	28
-CWRN	0.39925	2	2	15
-CWRY	0.39925	2	2	9
-CWSF	0.39925	1	1	12
-CWSY	0.39925	2	2	15
-CWTM	0.39925	2	2	19
-CWTN	0.39925	1	1	9
-CWTW	0.39925	1	1	9
-CWTY	0.39925	1	1	6
-CWVE	0.39925	1	1	7
-CWVF	0.39925	1	1	9

-CWVH	0.39925	1	1	6
-CWVL	0.39925	2	2	18
-CWVP	0.39925	1	1	11
-CWVR	0.39925	2	2	23
-CWVW	0.39925	1	1	13
-CWVY	0.39925	1	1	4
-CWWA	0.39925	2	2	6
-CWWC	0.39925	1	1	7
-CWWD	0.39925	1	1	2
-CWWE	0.39925	1	1	3
-CWWF	0.39925	1	1	19
-CWWH	0.39925	1	1	11
-CWWI	0.39925	1	1	4
-CWWN	0.39925	1	1	2
-CWWQ	0.39925	1	1	12
-CWWT	0.39925	1	1	15
-CWWV	0.39925	1	1	5
-CWWW	0.39925	1	1	4
-CWYC	0.39925	1	1	4
-CWYF	0.39925	1	1	10
-CWYG	0.39925	2	2	8
-CWYN	0.39925	1	1	7
-CWYP	0.39925	2	2	12
-CWYT	0.39925	1	1	12
-CWYW	0.39925	1	1	6
-CWYY	0.39925	1	1	4
-CYAC	0.39925	1	1	3
-CYAM	0.39925	1	1	12
-CYCC	0.39925	2	2	8
-CYCE	0.39925	1	1	9
-CYCH	0.39925	1	1	10
-CYCW	0.39925	1	1	7
-CYCY	0.39925	1	1	2
-CYDH	0.39925	3	3	24
-CYDK	0.39925	1	1	15
-CYDP	0.39925	3	3	13
-CYEY	0.39925	1	1	4
-CYFA	0.39925	1	1	5
-CYFC	0.39925	1	1	11
-CYFF	0.39925	1	1	6
-CYFG	0.39925	1	1	5
-CYFI	0.39925	1	1	2
-CYFK	0.39925	1	1	14
-CYFM	0.39925	1	1	6
-CYFP	0.39925	1	1	10
-CYFQ	0.39925	2	2	10
-CYFV	0.39925	1	1	15
-CYFW	0.39925	1	1	5
-CYFY	0.39925	1	1	7
-CYHC	0.39925	1	1	10
-CYHD	0.39925	2	2	6
-CYHR	0.39925	1	1	26
-CYHW	0.39925	1	1	13
-CYHY	0.39925	1	1	5
-CYID	0.39925	1	1	10
-CYIN	0.39925	1	1	22
-CYIR	0.39925	2	2	29

-CYKH	0.39925	1	1	8
-CYKK	0.39925	1	1	13
-CYKW	0.39925	1	1	2
-CYLF	0.39925	1	1	10
-CYMC	0.39925	1	1	6
-CYMF	0.39925	1	1	13
-CYMG	0.39925	1	1	9
-CYMH	0.39925	1	1	23
-CYMI	0.39925	2	2	9
-CYMM	0.39925	1	1	7
-CYMW	0.39925	1	1	8
-CYNC	0.39925	2	2	5
-CYNE	0.39925	1	1	4
-CYNM	0.39925	2	2	16
-CYNP	0.39925	1	1	6
-CYNY	0.39925	1	1	13
-CYQC	0.39925	1	1	7
-CYQD	0.39925	1	1	7
-CYQE	0.39925	2	2	9
-CYQH	0.39925	1	1	12
-CYQQ	0.39925	1	1	8
-CYQY	0.39925	1	1	4
-CYRF	0.39925	1	1	12
-CYRI	0.39925	1	1	13
-CYSK	0.39925	1	1	18
-CYTF	0.39925	1	1	13
-CYVG	0.39925	2	2	16
-CYVI	0.39925	2	2	13
-CYVN	0.39925	1	1	13
-CYVQ	0.39925	2	2	11
-CYWC	0.39925	1	1	6
-CYWD	0.39925	1	1	2
-CYWF	0.39925	1	1	5
-CYWI	0.39925	1	1	9
-CYWK	0.39925	1	1	7
-CYWL	0.39925	1	1	20
-CYWN	0.39925	1	1	6
-CYWW	0.39925	1	1	4
-CYWY	0.39925	1	1	1
-CYYC	0.39925	1	1	12
-CYYF	0.39925	1	1	1
-CYYI	0.39925	1	1	16
-CYYM	0.39925	2	2	13
-CYYN	0.39925	1	1	11
-CYYQ	0.39925	1	1	11
-CYYR	0.39925	3	3	21
-CYYY	0.39925	1	1	3
-CEDA	0.39925	5	5	13
-CIES	0.39925	5	5	10
-CIPM	0.39925	5	5	17
-CNEM	0.39925	5	5	14
-CQDQ	0.39925	5	5	16
-CRHC	0.39925	5	5	15
-CTPA	0.39925	5	5	23
-CTVY	0.39925	5	5	29
-CWEA	0.39925	5	5	4
-CTLT	0.4146	54	52	83

-CAVL	0.41826	22	21	59
-CVTV	0.42026	40	38	53
-CFLS	0.43076	41	38	44
-CSVF	0.43917	22	20	37
-CFVA	0.44361	10	9	16
-CLAM	0.44361	10	9	27
-CSCS	0.44361	10	9	45
-CVRR	0.45628	16	14	83
-CSSQ	0.46067	15	13	57
-CDPD	0.46579	7	6	18
-CWPQ	0.46579	7	6	16
-CGIT	0.47184	13	11	29
-CVTN	0.47909	6	5	32
-CGSL	0.48604	28	23	84
-CEAN	0.49906	5	4	15
-CFMC	0.49906	5	4	11
-CGKS	0.49906	10	8	25
-CGMY	0.49906	5	4	13
-CGYN	0.49906	5	4	19
-CIHA	0.49906	5	4	19
-CIMR	0.49906	10	8	19
-CQLA	0.49906	5	4	23
-CRCP	0.49906	5	4	19
-CSCF	0.49906	5	4	11
-CSSC	0.49906	20	16	49
-CVCI	0.49906	5	4	21
-CAML	0.50813	14	11	31
-CGVG	0.50813	14	11	23
-CVVE	0.50813	28	22	31
-CLAQ	0.51015	23	18	29
-CEMI	0.51332	9	7	9
-CRFS	0.51332	9	7	43
-CMVV	0.51902	13	10	31
-CARA	0.53233	4	3	25
-CCAF	0.53233	8	6	15
-CCAQ	0.53233	4	3	8
-CCDR	0.53233	4	3	13
-CDDQ	0.53233	4	3	11
-CDQY	0.53233	4	3	7
-CDVQ	0.53233	4	3	11
-CGKW	0.53233	4	3	19
-CGLF	0.53233	4	3	29
-CHDY	0.53233	4	3	9
-CHER	0.53233	4	3	25
-CHVV	0.53233	4	3	30
-CIDH	0.53233	8	6	10
-CKDA	0.53233	4	3	14
-CKMP	0.53233	4	3	5
-CKYS	0.53233	4	3	16
-CLTF	0.53233	4	3	28
-CMHE	0.53233	4	3	9
-CMQS	0.53233	4	3	27
-CPAE	0.53233	4	3	24
-CQMC	0.53233	4	3	14
-CRHL	0.53233	8	6	41
-CSCH	0.53233	4	3	20
-CTCH	0.53233	4	3	12

-CTSF	0.53233	4	3	41
-CHRA	0.54443	15	11	41
-CVML	0.54564	41	30	43
-CVDA	0.54896	11	8	13
-CAIG	0.55894	28	20	20
-CCYV	0.55894	7	5	19
-CHRH	0.55894	7	5	32
-CIRA	0.55894	7	5	23
-CMWR	0.55894	7	5	23
-CYWV	0.55894	7	5	14
-CSKN	0.5656	17	12	20
-CMMS	0.57669	13	9	37
-CCVL	0.58351	19	13	38
-CSQI	0.59887	9	6	8
-CACI	0.59887	3	2	9
-CAEH	0.59887	3	2	7
-CCDH	0.59887	3	2	5
-CCHD	0.59887	3	2	8
-CCML	0.59887	3	2	22
-CCQQ	0.59887	3	2	3
-CCRQ	0.59887	6	4	18
-CCYM	0.59887	3	2	7
-CDHH	0.59887	6	4	10
-CDPA	0.59887	6	4	18
-CEDD	0.59887	3	2	3
-CEIH	0.59887	3	2	9
-CFQL	0.59887	3	2	13
-CFRE	0.59887	3	2	17
-CFVQ	0.59887	3	2	18
-CGCC	0.59887	3	2	10
-CGHL	0.59887	3	2	39
-CGWG	0.59887	3	2	20
-CHCQ	0.59887	3	2	13
-CHDH	0.59887	3	2	6
-CHGH	0.59887	3	2	9
-CHVW	0.59887	3	2	20
-CIDG	0.59887	3	2	9
-CIKP	0.59887	3	2	16
-CIVD	0.59887	12	8	21
-CIWL	0.59887	3	2	12
-CKCR	0.59887	3	2	11
-CKID	0.59887	3	2	6
-CKYT	0.59887	3	2	13
-CLFC	0.59887	3	2	11
-CMLN	0.59887	3	2	20
-CMYS	0.59887	3	2	27
-CNCE	0.59887	6	4	23
-CPVF	0.59887	3	2	19
-CPYM	0.59887	3	2	19
-CQQR	0.59887	6	4	27
-CQVQ	0.59887	3	2	14
-CRIH	0.59887	3	2	16
-CRYQ	0.59887	6	4	19
-CSHA	0.59887	6	4	24
-CSQD	0.59887	3	2	10
-CTTH	0.59887	3	2	22
-CTWG	0.59887	3	2	18

-CVAH	0.59887	3	2	11
-CVQR	0.59887	6	4	22
-CWCL	0.59887	3	2	24
-CWCV	0.59887	3	2	4
-CWQQ	0.59887	3	2	2
-CWVT	0.59887	3	2	16
-CYCG	0.59887	3	2	5
-CYLI	0.59887	6	4	40
-CYND	0.59887	3	2	10
-CNHA	0.62739	11	7	17
-CSCA	0.62739	11	7	36
-CTID	0.62739	11	7	10
-CRIL	0.6341	27	17	51
-CSVV	0.6341	54	34	65
-CADP	0.63879	8	5	17
-CNHL	0.63879	8	5	21
-CWSC	0.63879	8	5	15
-CYRA	0.63879	8	5	32
-CAHI	0.64877	13	8	35
-CIAS	0.6559	23	14	45
-CCRM	0.66541	5	3	38
-CDLI	0.66541	5	3	25
-CDRH	0.66541	5	3	9
-CGIG	0.66541	5	3	27
-CGVY	0.66541	5	3	10
-CHLS	0.66541	10	6	49
-CHVR	0.66541	10	6	43
-CIMP	0.66541	5	3	12
-CKVA	0.66541	10	6	17
-CLIY	0.66541	5	3	5
-CNCF	0.66541	5	3	13
-CPGE	0.66541	5	3	20
-CQMM	0.66541	5	3	7
-CTLY	0.66541	5	3	40
-CTNF	0.66541	5	3	10
-CWSP	0.66541	5	3	28
-CWTG	0.66541	5	3	15
-CYHH	0.66541	5	3	14
-CYLP	0.66541	15	9	38
-CLCA	0.68442	60	35	25
-CCMA	0.69868	14	8	14
-CCTN	0.69868	7	4	16
-CDMM	0.69868	7	4	3
-CDTC	0.69868	7	4	27
-CDTH	0.69868	7	4	17
-CHGK	0.69868	7	4	11
-CPYP	0.69868	7	4	14
-CSPF	0.69868	7	4	32
-CNVT	0.70455	30	17	30
-CITV	0.70977	16	9	36
-CAVI	0.71864	9	5	20
-CDII	0.71864	9	5	8
-CGAK	0.71864	9	5	17
-CMYV	0.71864	9	5	25
-CTFS	0.74146	13	7	36
-CLMV	0.75413	17	9	41
-CFIC	0.7622	21	11	7

-CATM	0.79849	4	2	24
-CAWF	0.79849	2	1	6
-CCAI	0.79849	2	1	3
-CCCC	0.79849	2	1	6
-CCCR	0.79849	4	2	15
-CCDQ	0.79849	4	2	8
-CCDY	0.79849	2	1	13
-CCHC	0.79849	2	1	5
-CCHI	0.79849	2	1	7
-CCLH	0.79849	6	3	19
-CCMP	0.79849	2	1	14
-CCNP	0.79849	2	1	27
-CCPC	0.79849	2	1	9
-CCQI	0.79849	2	1	13
-CCRD	0.79849	4	2	24
-CCVK	0.79849	2	1	10
-CCVQ	0.79849	14	7	7
-CCWH	0.79849	2	1	11
-CCYF	0.79849	2	1	11
-CCYY	0.79849	2	1	11
-CDCF	0.79849	2	1	9
-CDCM	0.79849	2	1	5
-CDCV	0.79849	2	1	15
-CDFH	0.79849	2	1	7
-CDNP	0.79849	2	1	10
-CDPQ	0.79849	2	1	3
-CDQW	0.79849	4	2	7
-CDWR	0.79849	2	1	15
-CDWW	0.79849	2	1	7
-CEIP	0.79849	6	3	7
-CEMD	0.79849	2	1	6
-CEWQ	0.79849	2	1	3
-CEYH	0.79849	2	1	6
-CFCI	0.79849	2	1	6
-CFCP	0.79849	2	1	8
-CFCR	0.79849	2	1	8
-CFFM	0.79849	2	1	12
-CFHW	0.79849	2	1	8
-CFMY	0.79849	2	1	5
-CFPY	0.79849	2	1	8
-CFQP	0.79849	2	1	8
-CFRW	0.79849	2	1	13
-CFTM	0.79849	6	3	13
-CFYM	0.79849	2	1	5
-CFYQ	0.79849	2	1	5
-CFYY	0.79849	2	1	10
-CGHK	0.79849	2	1	19
-CGHP	0.79849	2	1	11
-CGIH	0.79849	2	1	7
-CGII	0.79849	2	1	8
-CGIY	0.79849	2	1	11
-CGMW	0.79849	2	1	22
-CGNP	0.79849	2	1	20
-CGVW	0.79849	2	1	19
-CHAA	0.79849	2	1	9
-CHAD	0.79849	2	1	18
-CHAI	0.79849	2	1	13

-CHAQ	0.79849	2	1	9
-CHFH	0.79849	2	1	4
-CHFI	0.79849	2	1	4
-CHFK	0.79849	2	1	3
-CHHQ	0.79849	4	2	8
-CHIY	0.79849	2	1	16
-CHKH	0.79849	2	1	9
-CHMC	0.79849	2	1	9
-CHNP	0.79849	2	1	7
-CHVY	0.79849	2	1	10
-CHWI	0.79849	2	1	6
-CHWM	0.79849	2	1	17
-CHWR	0.79849	2	1	18
-CIAC	0.79849	2	1	5
-CIDC	0.79849	2	1	3
-CIHC	0.79849	2	1	8
-CIIW	0.79849	4	2	18
-CIMD	0.79849	2	1	3
-CISH	0.79849	2	1	17
-CIVY	0.79849	6	3	13
-CKCL	0.79849	2	1	13
-CKFM	0.79849	2	1	10
-CKHA	0.79849	2	1	8
-CKIH	0.79849	2	1	7
-CKPD	0.79849	2	1	6
-CKWH	0.79849	2	1	12
-CLCT	0.79849	4	2	32
-CLQH	0.79849	2	1	14
-CLWF	0.79849	2	1	10
-CLYC	0.79849	2	1	15
-CMCH	0.79849	2	1	8
-CMIK	0.79849	2	1	8
-CMNE	0.79849	2	1	7
-CMNW	0.79849	2	1	10
-CMYI	0.79849	2	1	11
-CNFC	0.79849	2	1	24
-CNQD	0.79849	2	1	5
-CPCH	0.79849	2	1	18
-CPFV	0.79849	2	1	11
-CPHK	0.79849	2	1	11
-CPMI	0.79849	2	1	17
-CPVQ	0.79849	2	1	18
-CPWQ	0.79849	4	2	3
-CPYQ	0.79849	2	1	12
-CQHS	0.79849	2	1	10
-CQLE	0.79849	2	1	9
-CQLG	0.79849	6	3	20
-CQMQ	0.79849	2	1	18
-CQYG	0.79849	4	2	7
-CQYI	0.79849	2	1	4
-CQYV	0.79849	4	2	27
-CRCC	0.79849	2	1	10
-CRCV	0.79849	6	3	39
-CRFF	0.79849	2	1	9
-CRFK	0.79849	2	1	18
-CRFW	0.79849	2	1	23
-CRHE	0.79849	2	1	14

-CRMQ	0.79849	4	2	15
-CRTQ	0.79849	2	1	51
-CRWW	0.79849	2	1	17
-CRYC	0.79849	4	2	13
-CSCC	0.79849	4	2	19
-CSWH	0.79849	2	1	12
-CSYA	0.79849	8	4	28
-CTCP	0.79849	2	1	18
-CTHQ	0.79849	2	1	18
-CTPF	0.79849	6	3	16
-CTWP	0.79849	4	2	18
-CTWQ	0.79849	2	1	14
-CTYQ	0.79849	2	1	4
-CVFA	0.79849	2	1	23
-CVFG	0.79849	2	1	10
-CVFI	0.79849	2	1	15
-CVHA	0.79849	4	2	16
-CVMD	0.79849	2	1	11
-CVPP	0.79849	2	1	20
-CVYM	0.79849	8	4	19
-CWCH	0.79849	2	1	1
-CWCP	0.79849	4	2	17
-CWFH	0.79849	2	1	7
-CWFM	0.79849	2	1	11
-CWHI	0.79849	2	1	12
-CWHQ	0.79849	2	1	2
-CWHV	0.79849	2	1	7
-CWIF	0.79849	6	3	8
-CWIQ	0.79849	2	1	1
-CWMN	0.79849	2	1	5
-CWMP	0.79849	2	1	18
-CWPN	0.79849	2	1	16
-CWVQ	0.79849	4	2	8
-CYHA	0.79849	6	3	11
-CYHV	0.79849	2	1	19
-CYIQ	0.79849	4	2	14
-CYIY	0.79849	2	1	13
-CYKY	0.79849	2	1	6
-CYMA	0.79849	2	1	19
-CYMQ	0.79849	2	1	9
-CYNH	0.79849	2	1	5
-CYPM	0.79849	2	1	14
-CYVF	0.79849	2	1	16
-CYWP	0.79849	2	1	12
-CYYV	0.79849	2	1	16
-CCYL	0.79849	10	5	37
-CQGG	0.79849	10	5	11
-CSVN	0.81845	41	20	40
-CQLS	0.8195	39	19	46
-CKVS	0.82511	31	15	19
-CILG	0.83479	23	11	22
-CLIK	0.8484	17	8	23
-CHVT	0.86503	13	6	19
-CQVV	0.88721	20	9	9
-CDHQ	0.93157	7	3	15
-CEVA	0.93157	28	12	27
-CIML	0.93157	7	3	12

-CTKF	0.93157	7	3	16
-CVCN	0.93157	7	3	21
-CYHL	0.93157	7	3	38
-CQCF	0.94821	19	8	11
-CTCS	0.98276	32	13	39
-CFTQ	0.99811	5	2	12
-CGMM	0.99811	10	4	9
-CGRY	0.99811	5	2	26
-CHHL	0.99811	10	4	17
-CIMH	0.99811	5	2	10
-CKFQ	0.99811	5	2	18
-CLVH	0.99811	5	2	20
-CPNQ	0.99811	5	2	15
-CREC	0.99811	5	2	12
-CRQM	0.99811	5	2	7
-CWMM	0.99811	5	2	7
-CYHP	0.99811	5	2	15
-CYII	0.99811	5	2	10
-CNVL	1.0188	74	29	51
-CVWM	1.0266	18	7	15
-CVVY	1.0314	62	24	26
-CKLC	1.038	13	5	13
-CTTF	1.038	52	20	36
-CDHA	1.0647	8	3	16
-CGES	1.0647	8	3	23
-CIMN	1.0647	8	3	10
-CPCP	1.0647	8	3	17
-CVHQ	1.0647	8	3	13
-CSLA	1.0647	112	42	65
-CINQ	1.1312	17	6	18
-CISM	1.1363	37	13	23
-CRLA	1.1603	93	32	69
-CKIS	1.1767	56	19	14
-CAMA	1.1977	18	6	14
-CKLH	1.1977	9	3	15
-CMVT	1.1977	9	3	19
-CPCQ	1.1977	9	3	10
-CPIT	1.1977	9	3	23
-CTLV	1.1977	99	33	78
-CTVI	1.1977	39	13	28
-CVCY	1.1977	9	3	7
-CAHW	1.1977	3	1	15
-CAMH	1.1977	3	1	13
-CAWN	1.1977	3	1	8
-CCIM	1.1977	3	1	9
-CCMH	1.1977	3	1	10
-CCPQ	1.1977	3	1	5
-CDCQ	1.1977	3	1	4
-CDIN	1.1977	3	1	6
-CDVF	1.1977	3	1	11
-CDVS	1.1977	21	7	51
-CEEW	1.1977	6	2	10
-CEFM	1.1977	3	1	10
-CFGW	1.1977	3	1	11
-CGVC	1.1977	3	1	12
-CHHV	1.1977	3	1	14
-CHPQ	1.1977	3	1	18

-CIDQ	1.1977	3	1	14
-CIHH	1.1977	3	1	16
-CLWI	1.1977	3	1	11
-CMFA	1.1977	3	1	6
-CMGY	1.1977	3	1	3
-CMID	1.1977	3	1	13
-CNMF	1.1977	3	1	9
-CNMQ	1.1977	3	1	10
-CQRH	1.1977	3	1	14
-CRCF	1.1977	3	1	7
-CRFQ	1.1977	3	1	7
-CSQQ	1.1977	3	1	13
-CVFT	1.1977	3	1	20
-CVMH	1.1977	3	1	11
-CVMI	1.1977	15	5	15
-CVWP	1.1977	3	1	13
-CWRH	1.1977	3	1	12
-CYQI	1.1977	3	1	5
-CKIA	1.2548	44	14	10
-CTAM	1.2643	19	6	14
-CYLA	1.3206	43	13	57
-CLQQ	1.3574	17	5	11
-CCRA	1.3974	7	2	34
-CHCM	1.3974	7	2	2
-CPCA	1.3974	7	2	12
-CTGH	1.3974	7	2	26
-CVCA	1.3974	28	8	27
-CYPQ	1.3974	7	2	17
-CQVA	1.4259	50	14	10
-CTAQ	1.4259	25	7	25
-CDAN	1.4289	68	19	32
-CVSF	1.4449	76	21	22
-CIIE	1.4639	11	3	5
-CKIT	1.4639	11	3	13
-CTCC	1.4639	11	3	14
-CWAM	1.4639	11	3	13
-CLMF	1.4829	78	21	38
-CICL	1.4972	15	4	16
-CITH	1.4972	15	4	11
-CLLN	1.4972	90	24	41
-CNRT	1.4972	15	4	39
-CVSM	1.5048	98	26	46
-CNLS	1.5083	136	36	81
-CSMF	1.5171	57	15	35
-CIDA	1.5171	19	5	24
-CQTK	1.5471	31	8	29
-CKLM	1.5694	114	29	28
-CDFQ	1.597	4	1	20
-CECF	1.597	8	2	9
-CFIQ	1.597	4	1	2
-CHVQ	1.597	8	2	15
-CILH	1.597	24	6	21
-CPIA	1.597	28	7	17
-CQQG	1.597	4	1	6
-CRDH	1.597	4	1	7
-CRMF	1.597	8	2	13
-CTLF	1.597	24	6	16

-CTMH	1.597	4	1	14
-CVPF	1.597	24	6	10
-CVWA	1.597	4	1	22
-CYLQ	1.597	8	2	28
-CYML	1.597	4	1	26
-CVCT	1.6502	62	15	10
-CCVF	1.6635	50	12	15
-CVQC	1.6768	21	5	13
-CTMV	1.6838	97	23	38
-CGVK	1.6968	68	16	21
-CFVS	1.7153	116	27	15
-CEWM	1.7301	13	3	14
-CGIV	1.7301	130	30	37
-CCIG	1.7744	40	9	17
-CLTC	1.7754	209	47	35
-CDLQ	1.7966	9	2	12
-CLHQ	1.8631	14	3	14
-CWLC	1.8631	28	6	12
-CDIL	1.9004	119	25	28
-CAVT	1.9164	48	10	18
-CTLN	1.9297	145	30	58
-CKMM	1.9392	34	7	5
-CCQM	1.9519	44	9	17
-CDIV	1.9838	159	32	13
-CGIF	1.9962	15	3	11
-CVHM	1.9962	60	12	24
-CDIT	1.9962	5	1	10
-CEMC	1.9962	5	1	2
-CEYM	1.9962	5	1	20
-CFIL	1.9962	5	1	14
-CICC	1.9962	5	1	7
-CNCQ	1.9962	10	2	13
-CTDF	1.9962	5	1	10
-CVTQ	1.9962	10	2	23
-CWYQ	1.9962	5	1	12
-CQLM	2.0136	116	23	20
-CVVG	2.0483	118	23	18
-CVLV	2.0647	181	35	63
-CMLS	2.0898	335	64	52
-CHLQ	2.096	21	4	9
-CRCQ	2.1293	16	3	16
-CCVA	2.1339	155	29	19
-CAVF	2.1959	33	6	21
-CTFA	2.1959	33	6	14
-CPIS	2.2503	62	11	26
-CFIM	2.2624	17	3	2
-CAWQ	2.2846	103	18	18
-CMVM	2.2957	46	8	18
-CCMS	2.3384	41	7	17
-CMMC	2.3456	47	8	6
-CYIL	2.3755	119	20	31
-CHHG	2.3955	6	1	10
-CHHH	2.3955	6	1	10
-CIFM	2.3955	6	1	12
-CMCF	2.3955	6	1	9
-CMYK	2.3955	6	1	5
-CNVF	2.3955	12	2	9

-CPTQ	2.3955	6	1	18
-CQCM	2.3955	6	1	5
-CRII	2.3955	12	2	20
-CVFM	2.3955	6	1	10
-CVIW	2.3955	30	5	11
-CGLQ	2.4525	43	7	22
-CPYK	2.462	37	6	23
-CIAM	2.4753	31	5	14
-CVQM	2.5175	227	36	18
-CLIG	2.5701	103	16	34
-CDVM	2.6105	85	13	18
-CNVM	2.6616	100	15	17
-CIMV	2.706	122	18	12
-CSLS	2.707	617	91	156
-CRVC	2.7253	157	23	35
-CTYM	2.7377	48	7	12
-CMMM	2.7504	62	9	8
-CILC	2.7519	193	28	18
-CFIS	2.7774	160	23	27
-CTWM	2.7947	49	7	17
-CCMR	2.7947	7	1	15
-CEQR	2.7947	14	2	9
-CFIN	2.7947	7	1	16
-CIAQ	2.7947	35	5	8
-CTFQ	2.7947	7	1	17
-CYIF	2.7947	7	1	8
-CAMF	2.8137	148	21	26
-CSIY	2.8746	36	5	12
-CTAF	2.8834	130	18	15
-CITS	2.8945	116	16	33
-CMLA	2.8945	58	8	23
-CNMC	2.8945	29	4	15
-CTIE	2.8945	58	8	16
-CVCF	2.8945	116	16	16
-CVVH	2.9345	147	20	32
-CIIA	2.9356	125	17	24
-CLCF	2.9399	81	11	7
-CVMA	2.9516	207	28	24
-CVLF	2.9544	185	25	28
-CTIG	2.9943	15	2	17
-CWIV	2.9943	15	2	6
-CIMC	3.0442	61	8	4
-CVVI	3.0757	208	27	17
-CCLA	3.0822	193	25	41
-CIVV	3.0942	93	12	28
-CEIS	3.0942	124	16	30
-CFVC	3.1214	172	22	16
-CCYA	3.194	8	1	3
-CFIF	3.194	8	1	4
-CHFM	3.194	32	4	7
-CHLC	3.194	56	7	14
-CILV	3.194	112	14	30
-CNVV	3.194	96	12	26
-CSIQ	3.2161	145	18	25
-CIVA	3.2409	138	17	20
-CLVV	3.308	406	49	84
-CICV	3.321	183	22	15

-CYVA	3.327	25	3	21
-CIVL	3.3366	234	28	57
-CAIF	3.3651	59	7	6
-CVIM	3.3714	152	18	19
-CRIM	3.3754	93	11	13
-CVMQ	3.3754	93	11	17
-CEIV	3.3936	153	18	14
-CELM	3.3936	34	4	4
-CMVA	3.4069	128	15	10
-CNIT	3.4089	111	13	17
-CVVC	3.4185	137	16	32
-CLYG	3.4335	43	5	27
-CDIA	3.5134	132	15	23
-CWTQ	3.5134	44	5	10
-CRLS	3.5172	555	63	107
-CTCF	3.5267	53	6	8
-CAIC	3.5267	159	18	15
-CNCM	3.5362	62	7	9
-CGIS	3.5533	178	20	21
-CHHA	3.5932	9	1	11
-CHIN	3.5932	9	1	9
-CHYH	3.5932	9	1	18
-CKIM	3.5932	36	4	9
-CCVV	3.6671	248	27	33
-CIVI	3.7263	56	6	6
-CYIS	3.7529	141	15	19
-CEIF	3.7529	47	5	8
-CICT	3.7928	57	6	16
-CVIC	3.811	105	11	11
-CTCM	3.8594	29	3	11
-CICM	3.9562	109	11	7
-CIVQ	3.9592	119	12	26
-CLMA	3.9605	248	25	15
-CHIT	3.9714	189	19	25
-CKVC	3.9925	30	3	4
-CHMH	3.9925	10	1	11
-CPCF	3.9925	20	2	8
-CQIY	3.9925	20	2	4
-CTMF	3.9925	80	8	12
-CLLA	4.0205	574	57	81
-CVMV	4.0232	262	26	28
-CHIC	4.0257	242	24	11
-CQIS	4.059	61	6	20
-CCVS	4.1004	380	37	32
-CFIT	4.1065	72	7	5
-CYIT	4.2206	148	14	18
-CAIA	4.232	212	20	35
-CTVF	4.232	212	20	20
-CSIT	4.2438	287	27	43
-CRIF	4.303	97	9	6
-CEIC	4.3119	54	5	5
-CAVA	4.3149	281	26	38
-CTMC	4.3168	173	16	13
-CTMA	4.3212	184	17	36
-CAIM	4.3303	141	13	13
-CLVM	4.3667	175	16	44
-CVCV	4.3769	296	27	25

-CCCQ	4.3917	33	3	1
-CMIN	4.3917	99	9	9
-CIMM	4.3917	22	2	5
-CLIH	4.3917	11	1	17
-CELC	4.4117	221	20	25
-CVVQ	4.4449	167	15	15
-CNIF	4.4804	101	9	10
-CTIH	4.4982	169	15	18
-CWVC	4.5137	407	36	29
-CICF	4.5145	147	13	7
-CIVM	4.5248	34	3	12
-CHIA	4.5514	57	5	6
-CVVN	4.576	298	26	15
-CPMC	4.5913	161	14	28
-CKLQ	4.5913	23	2	21
-CQFM	4.5913	23	2	13
-CTIQ	4.5913	299	26	23
-CMVS	4.608	277	24	39
-CYVS	4.6612	467	40	55
-CIVG	4.6769	328	28	28
-CCLS	4.6849	751	64	88
-CKIC	4.7111	59	5	10
-CVVF	4.7184	130	11	26
-CLVT	4.7377	356	30	56
-CEVC	4.7602	155	13	13
-CMVC	4.7909	108	9	5
-CCIH	4.7909	12	1	5
-CPIV	4.7909	96	8	19
-CLVN	4.8575	146	12	20
-CIIV	4.8908	49	4	11
-CITF	4.8908	98	8	16
-CNVQ	4.8998	135	11	23
-CVIF	4.8998	135	11	11
-CNIL	4.9011	356	29	18
-CRMC	4.9092	332	27	25
-CLIF	4.9138	160	13	21
-CFCM	4.9204	456	37	17
-CTVN	4.9388	334	27	24
-CIFQ	4.9906	25	2	14
-CMIC	4.9906	25	2	8
-CALA	5.0191	264	21	37
-CRVM	5.0191	176	14	34
-CEIL	5.0305	315	25	23
-CRVA	5.0806	649	51	52
-CVLC	5.0845	433	34	44
-CIID	5.0904	51	4	18
-CFVM	5.1176	141	11	10
-CAMS	5.1197	218	17	36
-CVVM	5.1482	245	19	16
-CNLM	5.1583	323	25	31
-CCIS	5.1902	156	12	11
-CIMT	5.1902	273	21	21
-CIPF	5.1902	13	1	7
-CMIY	5.1902	13	1	5
-CVTM	5.2288	406	31	40
-CMVQ	5.2301	262	20	16
-CEII	5.29	159	12	14

-CVVL	5.2953	504	38	98
-CMLC	5.31	133	10	16
-CFLC	5.3233	40	3	14
-CVVV	5.3732	323	24	40
-CIII	5.3898	162	12	16
-CCMM	5.3898	54	4	16
-CMCQ	5.3898	54	4	5
-CVCS	5.412	244	18	25
-CLIM	5.4231	163	12	16
-CHVA	5.4297	340	25	35
-CILA	5.4359	177	13	28
-CIVT	5.4373	286	21	40
-CTIF	5.4397	109	8	9
-CTQM	5.4397	109	8	20
-CIIN	5.4754	96	7	9
-CAIQ	5.4896	110	8	14
-CTVL	5.4896	550	40	110
-CPLQ	5.5324	97	7	41
-CQLC	5.5395	111	8	17
-CSVA	5.5599	376	27	68
-CNVA	5.5609	390	28	34
-CTVT	5.569	544	39	47
-CCVT	5.5894	196	14	23
-CAIH	5.5894	28	2	20
-CIMQ	5.5894	14	1	11
-CVIE	5.6054	351	25	41
-CTIC	5.6094	281	20	20
-CVIS	5.6351	494	35	53
-CYLS	5.641	438	31	82
-CLIT	5.6427	212	15	19
-CSVQ	5.6643	454	32	52
-CAVC	5.675	199	14	33
-CDVC	5.6892	57	4	14
-CQIM	5.6892	57	4	6
-CILT	5.7276	373	26	47
-CVLA	5.7373	388	27	29
-CAVV	5.7538	245	17	50
-CSIC	5.7773	246	17	31
-CVVA	5.7811	362	25	45
-CNVS	5.8052	538	37	39
-CVAM	5.8478	249	17	32
-CYVM	5.8845	339	23	28
-CIVH	5.8889	59	4	8
-CTMM	5.8889	118	8	23
-CLFQ	5.9316	208	14	17
-CSFM	5.9677	284	19	15
-CVTS	5.9887	555	37	52
-CIIQ	5.9887	15	1	18
-CIIY	5.9887	30	2	8
-CILF	6.025	166	11	28
-CVCM	6.0307	287	19	26
-CVFC	6.0885	61	4	9
-CSMC	6.0937	580	38	41
-CLCS	6.1046	474	31	28
-CLLS	6.1187	1977	129	121
-CHIM	6.1339	169	11	10
-CLVA	6.1408	323	21	31

-CMIV	6.1484	77	5	24
-CVLT	6.1505	570	37	38
-CGIM	6.1883	62	4	8
-CTIV	6.1957	419	27	39
-CLTQ	6.2105	140	9	43
-CTIL	6.2198	592	38	72
-CYIA	6.247	266	17	22
-CSVC	6.2756	503	32	28
-CLLC	6.2881	441	28	37
-CIIT	6.3153	174	11	24
-CLVS	6.3225	966	61	88
-CIVS	6.3265	412	26	57
-CLCM	6.3547	382	24	43
-CDIQ	6.3879	16	1	11
-CGFQ	6.3879	16	1	13
-CNFM	6.3879	128	8	14
-CTFC	6.3879	16	1	6
-CHVS	6.3879	336	21	34
-CSIM	6.4349	274	17	20
-CWIT	6.4678	81	5	14
-CCIT	6.502	114	7	10
-CEVS	6.5035	619	38	21
-CTIY	6.521	49	3	10
-CVMT	6.5476	410	25	63
-CCVC	6.5523	279	17	19
-CEIY	6.5876	33	2	6
-CWVS	6.5876	363	22	14
-CICA	6.6009	248	15	14
-CAVS	6.6075	331	20	41
-CAIV	6.6161	116	7	27
-CLIS	6.6508	683	41	65
-CNIN	6.6541	100	6	6
-CWIC	6.6541	50	3	5
-CVLM	6.7346	641	38	54
-CLVC	6.7565	220	13	28
-CYLC	6.7587	237	14	18
-CAMQ	6.7872	17	1	17
-CAWM	6.7872	17	1	10
-CEMM	6.7872	68	4	6
-CGIA	6.7872	34	2	9
-CSVS	6.8014	1431	84	101
-CVMS	6.8053	375	22	44
-CQIA	6.8442	120	7	19
-CMIS	6.862	275	16	19
-CTVC	6.9203	156	9	27
-CEIT	6.9469	87	5	9
-CLVQ	7.0302	405	23	38
-CSIF	7.0329	229	13	12
-CMFM	7.0866	142	8	14
-CVMM	7.0866	355	20	20
-CILS	7.116	909	51	93
-CIVF	7.1199	107	6	13
-CDIF	7.1864	18	1	1
-CTMS	7.1969	685	38	61
-CVVS	7.1989	577	32	61
-CFMM	7.2663	273	15	22
-CVMN	7.3195	55	3	19

-CTLM	7.394	463	25	29
-CTII	7.4859	150	8	27
-CDIS	7.5058	94	5	23
-CKIV	7.5058	94	5	19
-CIVC	7.5191	113	6	18
-CLLM	7.5221	829	44	57
-CMLQ	7.5387	321	17	25
-CTIA	7.5635	341	18	40
-CTIS	7.5749	702	37	34
-CRIS	7.63	688	36	57
-CDIM	7.6471	249	13	20
-CPVM	7.6583	211	11	22
-CCCF	7.7187	58	3	5
-CILQ	7.7419	446	23	24
-CQIL	7.7454	194	10	26
-CRLC	7.752	466	24	29
-CALC	7.7699	253	13	17
-CTVA	7.7758	409	21	36
-CCIQ	7.8138	137	7	15
-CLLQ	7.8231	725	37	38
-CIIG	7.8252	196	10	7
-CCTM	7.8518	295	15	28
-CTIN	7.8904	1502	76	43
-CPCM	7.9123	218	11	13
-CTLS	7.935	1113	56	83
-CHVC	7.9849	260	13	34
-CRIN	7.9849	120	6	15
-CVMC	7.9849	420	21	26
-CVIH	7.9849	80	4	9
-CKVM	8.0156	261	13	19
-CVLN	8.0229	844	42	65
-CCIA	8.0515	121	6	18
-CVCQ	8.0847	81	4	6
-CALS	8.091	1297	64	103
-CVAF	8.118	183	9	18
-CTIM	8.132	387	19	19
-CVIN	8.1446	102	5	15
-CLMS	8.1535	919	45	63
-CRIC	8.1999	267	13	22
-CLQM	8.2289	371	18	30
-CLIA	8.239	227	11	32
-CYIM	8.2511	62	3	15
-CPMM	8.2753	228	11	23
-CMIA	8.2843	166	8	16
-CKIQ	8.3271	146	7	12
-CPFM	8.3842	21	1	11
-CEIA	8.4412	148	7	11
-CRVS	8.4495	1164	55	79
-CVVT	8.606	388	18	41
-CCIL	8.6237	324	15	22
-CTVV	8.6272	497	23	31
-CFCQ	8.6503	65	3	4
-CQIV	8.6503	65	3	7
-CVLQ	8.6932	675	31	25
-CQVC	8.7108	240	11	10
-CIMA	8.7834	132	6	6
-CGIC	8.7834	44	2	8

-CRLM	8.81	662	30	75
-CVIL	8.9122	692	31	51
-CSIV	8.9631	449	20	29
-CSLM	8.9761	652	29	59
-CTLA	8.9899	653	29	30
-CSMM	8.9963	338	15	35
-CIIS	9.0115	158	7	19
-CCII	9.0401	317	14	15
-CMII	9.0496	136	6	17
-CPIL	9.0914	797	35	45
-CNMM	9.1028	114	5	12
-CVAQ	9.1122	388	17	24
-CYVC	9.1383	206	9	15
-CCIY	9.1826	69	3	6
-CQIF	9.1826	23	1	7
-CMIL	9.2326	185	8	26
-CTVM	9.3278	514	22	44
-CGLM	9.347	796	34	52
-CRMM	9.3508	445	19	27
-CGVM	9.3623	469	20	30
-CCMQ	9.3823	47	2	3
-CILN	9.3887	729	31	40
-CNLC	9.4186	519	22	21
-CYIV	9.4222	236	10	20
-CVIG	9.473	261	11	19
-CVTF	9.4768	451	19	39
-CCLC	9.4868	499	21	34
-CWLM	9.502	119	5	13
-CNIC	9.5819	216	9	20
-CWIA	9.696	170	7	20
-CIMS	9.7017	243	10	18
-CSIS	9.7097	608	25	65
-CVMF	9.753	171	7	7
-CVIV	9.7815	147	6	23
-CCIF	9.8481	222	9	16
-CHIQ	9.9811	75	3	13
-CALM	9.9811	325	13	31
-CCVN	9.9811	25	1	8
-CSVM	9.9811	250	10	37
-CSIL	10.0118	1304	52	64
-CAMM	10.0255	452	18	20
-CLIQ	10.0255	226	9	18
-CHVM	10.031	201	8	36
-CPII	10.0952	177	7	16
-CVIA	10.1237	355	14	40
-CVIY	10.1408	127	5	10
-CPLC	10.1586	229	9	25
-CLIC	10.2207	128	5	10
-CSLC	10.2621	694	27	41
-CPLM	10.2624	1131	44	71
-CIIL	10.3138	620	24	35
-CDLM	10.4111	339	13	24
-CLII	10.4945	184	7	17
-CQIC	10.5256	290	11	7
-CINM	10.58	53	2	9
-CVMY	10.58	53	2	9
-CQVM	10.6465	160	6	17

-CWIM	10.6465	80	3	15
-CACF	10.7131	161	6	10
-CRIV	10.7131	322	12	20
-CRFM	10.7796	567	21	35
-CMIT	10.7796	27	1	7
-CFLM	10.8628	653	24	39
-CAIT	10.8794	218	8	21
-CCFQ	11.0902	250	9	15
-CAIN	11.1789	140	5	18
-CHIL	11.1789	84	3	21
-CIQM	11.1789	224	8	14
-CLCQ	11.1789	280	10	16
-CQIQ	11.1789	56	2	2
-CRIQ	11.1789	140	5	12
-CQII	11.2232	253	9	7
-CVCC	11.2787	113	4	4
-CNIQ	11.312	85	3	13
-CSII	11.35	398	14	29
-CTVS	11.4017	1228	43	70
-CCIV	11.464	201	7	10
-CCFM	11.5055	317	11	23
-CCTQ	11.5781	29	1	13
-CEIQ	11.5781	58	2	4
-CVIT	11.5781	87	3	21
-CAIY	11.7378	147	5	12
-CVLS	11.8038	1360	46	95
-CLIV	11.9411	329	11	33
-CCIN	11.9774	30	1	7
-CHIF	11.9774	30	1	6
-CNIA	12.0661	544	18	19
-CLMM	12.177	305	10	24
-CIQC	12.2435	92	3	11
-CKCM	12.2768	123	4	13
-CILM	12.3196	216	7	11
-CTLQ	12.421	560	18	35
-CACM	12.5762	504	16	18
-CWCM	12.6871	572	18	12
-CHIV	12.7759	64	2	15
-CNVC	12.7759	64	2	13
-CYIC	12.7759	32	1	8
-CLFM	12.8646	580	18	22
-CICN	12.8757	129	4	20
-CRVQ	13.042	98	3	26
-CLTM	13.061	458	14	44
-CNIY	13.1751	66	2	8
-CHLM	13.3642	636	19	26
-CFIV	13.3747	67	2	12
-CWMQ	13.3747	67	2	5
-CAFM	13.4292	370	11	26
-CSLQ	13.4679	506	15	47
-CRIA	13.4888	473	14	26
-CFIA	13.5743	204	6	20
-CRIT	13.5743	272	8	23
-CTMQ	13.6665	445	13	12
-CMIF	13.8405	104	3	9
-CLCC	13.8671	521	15	11
-CLMC	13.9237	279	8	22

-CNII	14.1732	426	12	23
-CIIC	14.3728	72	2	5
-CIWQ	14.3728	72	2	5
-CTLC	14.3728	432	12	33
-CSIN	14.4527	181	5	27
-CCIC	14.4726	145	4	14
-CQLQ	14.5725	146	4	23
-CCMC	14.6922	368	10	5
-CAVQ	14.7322	369	10	17
-CMIQ	14.7721	37	1	11
-CVIQ	14.9318	187	5	17
-CIIF	14.9717	75	2	9
-CVID	15.0116	376	10	18
-CWCQ	15.3044	230	6	12
-CPIC	15.371	539	14	12
-CAMC	15.4565	271	7	12
-CTTM	15.5107	777	20	44
-CCLQ	15.6276	274	7	23
-CYLM	15.6504	196	5	35
-CIIM	15.6704	157	4	12
-CIMF	15.8558	278	7	15
-CDIC	15.9698	120	3	7
-CHII	15.9698	40	1	11
-CCLM	16.3691	328	8	33
-CQIN	16.3691	41	1	19
-CITM	16.5687	498	12	9
-CITC	16.6685	167	4	10
-CQIT	16.7683	84	2	5
-CSCQ	17.0345	128	3	10
-CAIL	17.0877	642	15	40
-CICS	17.4071	218	5	14
-CSIA	17.5169	351	8	41
-CLWQ	17.5668	132	3	21
-CLIL	17.7034	1685	38	66
-CPVC	17.7442	400	9	24
-CVII	17.7949	312	7	23
-CCVM	17.966	90	2	18
-CSCM	18.3653	276	6	15
-CALQ	18.5782	698	15	33
-CNIV	18.7645	141	3	21
-CWLQ	18.7645	282	6	18
-CTVQ	18.8644	378	8	26
-CTCQ	19.1331	623	13	36
-CMMQ	19.2636	193	4	23
-CEVM	19.4299	146	3	22
-CNLQ	19.4904	537	11	21
-CEIM	19.563	49	1	11
-CICQ	19.6961	148	3	5
-CRLQ	19.7294	593	12	48
-CIIH	19.7627	198	4	12
-CAII	20.0954	151	3	2
-CMLM	20.1619	101	2	21
-CVTC	20.1619	303	6	12
-CMCM	20.3615	153	3	7
-CNIM	20.7608	156	3	16
-CAVM	21.0269	158	3	24
-CSMQ	21.3818	482	9	15

-CWIS	21.6163	379	7	18
-CFMQ	21.9585	55	1	7
-CPIM	22.8568	458	8	20
-CTIT	22.9965	288	5	34
-CWVM	23.7152	297	5	11
-CAIS	24.1258	423	7	36
-CTFM	25.1525	63	1	26
-CLIN	25.219	379	6	23
-CMIM	25.3521	127	2	6
-CRCM	26.065	457	7	28
-CGIQ	26.1284	589	9	18
-CLMQ	27.6677	693	10	16
-CPIF	28.1183	493	7	16
-CQVS	28.1468	141	2	12
-CYCQ	28.7013	647	9	6
-CHMM	29.5442	74	1	14
-CIVN	31.3408	157	2	17
-CIFC	31.9396	80	1	4
-CNIS	32.9378	330	4	30
-CHIS	34.3351	86	1	21
-CCCM	35.5329	89	1	3
-CACQ	39.8247	399	4	16
-CFLQ	59.6872	299	2	38
-CYCM	70.6665	177	1	12
