# Supplementary material for: Towards the Systematic Mapping and Engineering of the Protein Prenylation Machinery in Saccharomyces cerevisiae
Source: PLoS One. 2015 Mar 13;10(3):e0120716. doi: 10.1371/journal.pone.0120716 (PMC4358939; doi:10.1371/journal.pone.0120716)
Supplement: S3 File — (PDF) [file pone.0120716.s003.pdf]

Motif	Counts 37 °C	25 °C	Naive
CCFD	1	1	1
CCNF	1	1	1
CCNY	1	1	1
CDFI	1	1	1
CDWC	1	1	1
CEDE	1	1	1
CGHF	1	1	1
CHYY	1	1	1
CICD	1	1	1
CIWC	1	1	1
CIYP	1	1	1
CMDC	1	1	1
CMDE	1	1	1
CQEC	1	1	1
CQQE	1	1	1
CQQQ	1	1	1
CQWI	1	1	1
CWEE	1	1	1
CWGH	1	1	1
CWGQ	1	1	1
CWHF	1	1	1
CWIN	1	1	1
CWNM	1	1	1
CYWY	1	1	1
CYYF	1	1	1
CCCI	1	1	2
CCEM	1	1	2
CCYH	1	1	2
CDFE	1	1	2
CDQE	1	1	2
CDWF	1	1	2
CDYW	1	1	2
CEGM	1	1	2
CEKK	1	1	2
CEMK	1	1	2
CEWH	1	1	2
CEWI	1	1	2
CFEW	1	1	2
CFFW	1	1	2
CGFN	1	1	2
CGHW	1	1	2
CHKD	1	1	2
CHMW	1	1	2
CHYW	1	1	2
CIAF	1	1	2
CIEF	1	1	2
CIMI	1	1	2
CIWH	1	1	2
CKDY	1	1	2
CKEA	1	1	2

CKFF	1	1	2
CKHD	1	1	2
CKQP	1	1	2
CKWI	1	1	2
CMCC	1	1	2
CMCK	1	1	2
CMDW	1	1	2
CMHH	1	1	2
CMME	1	1	2
CMMW	1	1	2
CMQP	1	1	2
CNFD	1	1	2
CPWC	1	1	2
CQCD	1	1	2
CQHE	1	1	2
CQKW	1	1	2
CQNF	1	1	2
CQPC	1	1	2
CQPD	1	1	2
CQWC	1	1	2
CVDK	1	1	2
CWCW	1	1	2
CWDA	1	1	2
CWDW	1	1	2
CWKF	1	1	2
CWKQ	1	1	2
CWMF	1	1	2
CWMH	1	1	2
CWWD	1	1	2
CWWN	1	1	2
CYCY	1	1	2
CYFI	1	1	2
CYKW	1	1	2
CYWD	1	1	2
CCFN	1	1	3
CCHM	1	1	3
CCQE	1	1	3
CDHI	1	1	3
CEEI	1	1	3
CEHH	1	1	3
CEWW	1	1	3
CFDW	1	1	3
CFEF	1	1	3
CFFQ	1	1	3
CFHK	1	1	3
CFIH	1	1	3
CFPW	1	1	3
CFWF	1	1	3
CFWK	1	1	3
CFYC	1	1	3
CHFF	1	1	3
CHFN	1	1	3
CHHF	1	1	3
CHHW	1	1	3

CHIP	1	1	3
CHKM	1	1	3
CHYF	1	1	3
CHYQ	1	1	3
CIED	1	1	3
CIFE	1	1	3
CKDK	1	1	3
CKFI	1	1	3
CKIF	1	1	3
CKMW	1	1	3
CKNI	1	1	3
CKYI	1	1	3
CMFC	1	1	3
CMFD	1	1	3
CMKH	1	1	3
CMWW	1	1	3
CMYF	1	1	3
CNFY	1	1	3
CNYQ	1	1	3
CNYW	1	1	3
CQCW	1	1	3
CQDC	1	1	3
CQEA	1	1	3
CQHA	1	1	3
CQNN	1	1	3
CQNW	1	1	3
CQQY	1	1	3
CQWM	1	1	3
CQYE	1	1	3
CQYW	1	1	3
CWAH	1	1	3
CWCI	1	1	3
CWCY	1	1	3
CWDP	1	1	3
CWEQ	1	1	3
CWFF	1	1	3
CWII	1	1	3
CWIW	1	1	3
CWNC	1	1	3
CWQF	1	1	3
CWWE	1	1	3
CYAC	1	1	3
CYYY	1	1	3
CAHH	1	1	4
CCCG	1	1	4
CCEA	1	1	4
CCMN	1	1	4
CCNW	1	1	4
CCWF	1	1	4
CEDM	1	1	4
CEEQ	1	1	4
CEFQ	1	1	4
CEHC	1	1	4
CEKI	1	1	4

CEQF	1	1	4
CEQQ	1	1	4
CEWF	1	1	4
CFFY	1	1	4
CFKE	1	1	4
CFKI	1	1	4
CFMF	1	1	4
CFWE	1	1	4
CHCG	1	1	4
CHHK	1	1	4
CHNF	1	1	4
CHQP	1	1	4
CHWY	1	1	4
CICE	1	1	4
CIKI	1	1	4
CIWM	1	1	4
CIYF	1	1	4
CKDC	1	1	4
CKEW	1	1	4
CKQY	1	1	4
CMCI	1	1	4
CMDF	1	1	4
CMFF	1	1	4
CMQM	1	1	4
CMQY	1	1	4
CNCD	1	1	4
CNWF	1	1	4
CNWI	1	1	4
CPPW	1	1	4
CPQD	1	1	4
CQCK	1	1	4
CQEF	1	1	4
CQHC	1	1	4
CQHF	1	1	4
CQHN	1	1	4
CQPE	1	1	4
CQPK	1	1	4
CQQM	1	1	4
CQYQ	1	1	4
CQYY	1	1	4
CTWI	1	1	4
CWDV	1	1	4
CWEI	1	1	4
CWFW	1	1	4
CWGF	1	1	4
CWHD	1	1	4
CWHE	1	1	4
CWKK	1	1	4
CWKV	1	1	4
CWQH	1	1	4
CWVY	1	1	4
CWWI	1	1	4
CWWW	1	1	4
CWYC	1	1	4

CWYY	1	1	4
CYEY	1	1	4
CYNE	1	1	4
CYQY	1	1	4
CYWW	1	1	4
CAQQ	1	1	5
CCAH	1	1	5
CCDC	1	1	5
CCFH	1	1	5
CCNG	1	1	5
CCQF	1	1	5
CCWT	1	1	5
CCWV	1	1	5
CCWW	1	1	5
CDCW	1	1	5
CDIY	1	1	5
CDWI	1	1	5
CEDF	1	1	5
CEFV	1	1	5
CEGW	1	1	5
CEHQ	1	1	5
CEID	1	1	5
CEKY	1	1	5
CEPI	1	1	5
CEYK	1	1	5
CFEC	1	1	5
CFFD	1	1	5
CFHH	1	1	5
CFHI	1	1	5
CFMW	1	1	5
CFWG	1	1	5
CFWH	1	1	5
CFWW	1	1	5
CFWY	1	1	5
CGGC	1	1	5
CGQY	1	1	5
CHCA	1	1	5
CHFY	1	1	5
CHNW	1	1	5
CHWQ	1	1	5
CIEW	1	1	5
CIFP	1	1	5
CINC	1	1	5
CINP	1	1	5
CIVW	1	1	5
CKCE	1	1	5
CKHY	1	1	5
CKIW	1	1	5
CKQH	1	1	5
CKWF	1	1	5
CKYQ	1	1	5
CMCW	1	1	5
CMFH	1	1	5
CMKF	1	1	5

CMMY	1	1	5
CMPG	1	1	5
CMYW	1	1	5
CNCC	1	1	5
CNDC	1	1	5
CNFQ	1	1	5
CNMI	1	1	5
CNQH	1	1	5
CQDW	1	1	5
CQFH	1	1	5
CQHI	1	1	5
CQIW	1	1	5
CQWE	1	1	5
CQWH	1	1	5
CQWW	1	1	5
CQYM	1	1	5
CWCC	1	1	5
CWCN	1	1	5
CWDC	1	1	5
CWDD	1	1	5
CWFY	1	1	5
CWMA	1	1	5
CWMD	1	1	5
CWNW	1	1	5
CWPP	1	1	5
CWQI	1	1	5
CWQP	1	1	5
CWWV	1	1	5
CYFA	1	1	5
CYFG	1	1	5
CYFW	1	1	5
CYHY	1	1	5
CYWF	1	1	5
CACY	1	1	6
CADM	1	1	6
CAQC	1	1	6
CCGI	1	1	6
CCNI	1	1	6
CCQN	1	1	6
CCWK	1	1	6
CDCC	1	1	6
CDEG	1	1	6
CDFY	1	1	6
CDTA	1	1	6
CDWH	1	1	6
CEAF	1	1	6
CEDC	1	1	6
CEFA	1	1	6
CEFF	1	1	6
CEHY	1	1	6
CEME	1	1	6
CEQD	1	1	6
CFKC	1	1	6
CFKH	1	1	6

CFQD	1	1	6
CFQF	1	1	6
CFYD	1	1	6
CFYF	1	1	6
CFYG	1	1	6
CGFW	1	1	6
CGQF	1	1	6
CGWH	1	1	6
CGWI	1	1	6
CGWM	1	1	6
CGYW	1	1	6
CHCC	1	1	6
CHCF	1	1	6
CHHC	1	1	6
CHQC	1	1	6
CHWK	1	1	6
CIEC	1	1	6
CIWE	1	1	6
CIWI	1	1	6
CIYW	1	1	6
CKKW	1	1	6
CMEA	1	1	6
CMFP	1	1	6
CMHF	1	1	6
CMQH	1	1	6
CMQQ	1	1	6
CMWY	1	1	6
CNCA	1	1	6
CNCW	1	1	6
CNDI	1	1	6
CNNI	1	1	6
CNQQ	1	1	6
CNWC	1	1	6
CNYC	1	1	6
CPFF	1	1	6
CPHF	1	1	6
CQDF	1	1	6
CQDM	1	1	6
CQFN	1	1	6
CQHK	1	1	6
CQHP	1	1	6
CQKQ	1	1	6
CQMY	1	1	6
CQPG	1	1	6
CQQK	1	1	6
CQQP	1	1	6
CQVH	1	1	6
CTWW	1	1	6
CVQH	1	1	6
CWAF	1	1	6
CWCF	1	1	6
CWEK	1	1	6
CWFE	1	1	6
CWGK	1	1	6

CWHH	1	1	6
CWMY	1	1	6
CWNN	1	1	6
CWNY	1	1	6
CWPF	1	1	6
CWTY	1	1	6
CWVH	1	1	6
CWYW	1	1	6
CYFF	1	1	6
CYFM	1	1	6
CYMC	1	1	6
CYNP	1	1	6
CYWC	1	1	6
CYWN	1	1	6
CCCE	1	1	7
CCEG	1	1	7
CCGH	1	1	7
CCIW	1	1	7
CCKW	1	1	7
CCKY	1	1	7
CCNM	1	1	7
CCPH	1	1	7
CCQW	1	1	7
CCTH	1	1	7
CCVE	1	1	7
CCWL	1	1	7
CCWM	1	1	7
CCWP	1	1	7
CCYI	1	1	7
CDNI	1	1	7
CEEG	1	1	7
CEQM	1	1	7
CEWC	1	1	7
CFFF	1	1	7
CFKM	1	1	7
CFYP	1	1	7
CGAH	1	1	7
CGFC	1	1	7
CGFE	1	1	7
CGPQ	1	1	7
CGWW	1	1	7
CHDG	1	1	7
CHFD	1	1	7
CHWW	1	1	7
CHYI	1	1	7
CICW	1	1	7
CIEY	1	1	7
CIFI	1	1	7
CIPQ	1	1	7
CIYI	1	1	7
CKDD	1	1	7
CKHH	1	1	7
CKWY	1	1	7
CLWY	1	1	7

CMCT	1	1	7
CMGW	1	1	7
CMHQ	1	1	7
CMRM	1	1	7
CMWC	1	1	7
CNHH	1	1	7
CNKE	1	1	7
CPAQ	1	1	7
CPQM	1	1	7
CPWH	1	1	7
CQCH	1	1	7
CQEI	1	1	7
CQHG	1	1	7
CQPH	1	1	7
CQPM	1	1	7
CQRW	1	1	7
CQWK	1	1	7
CRHW	1	1	7
CRYI	1	1	7
CTQP	1	1	7
CTYY	1	1	7
CVFH	1	1	7
CVWY	1	1	7
CWDF	1	1	7
CWDN	1	1	7
CWIK	1	1	7
CWKM	1	1	7
CWKW	1	1	7
CWMI	1	1	7
CWND	1	1	7
CWVE	1	1	7
CWWC	1	1	7
CWYN	1	1	7
CYCW	1	1	7
CYFY	1	1	7
CYMM	1	1	7
CYQC	1	1	7
CYQD	1	1	7
CYWK	1	1	7
CAED	1	1	8
CCIK	1	1	8
CCKC	1	1	8
CCQG	1	1	8
CCYN	1	1	8
CDQA	1	1	8
CDWY	1	1	8
CDYI	1	1	8
CEQP	1	1	8
CETQ	1	1	8
CEVF	1	1	8
CEWY	1	1	8
CFCF	1	1	8
CFCN	1	1	8
CFHN	1	1	8

CFIK	1	1	8
CFYA	1	1	8
CFYW	1	1	8
CGCW	1	1	8
CGFY	1	1	8
CGHE	1	1	8
CGQD	1	1	8
CHEK	1	1	8
CHFQ	1	1	8
CHMF	1	1	8
CIAN	1	1	8
CIFW	1	1	8
CIQP	1	1	8
CKQQ	1	1	8
CKWE	1	1	8
CKWR	1	1	8
CKYG	1	1	8
CMCN	1	1	8
CMDY	1	1	8
CMEC	1	1	8
CMEI	1	1	8
CMHC	1	1	8
CMMI	1	1	8
CMQF	1	1	8
CMWM	1	1	8
CMYM	1	1	8
CNEN	1	1	8
CNIW	1	1	8
CNNE	1	1	8
CQCC	1	1	8
CQDN	1	1	8
CQKA	1	1	8
CQSK	1	1	8
CQWF	1	1	8
CQWR	1	1	8
CTWY	1	1	8
CVHH	1	1	8
CWFI	1	1	8
CWHK	1	1	8
CWIH	1	1	8
CWNQ	1	1	8
CWQW	1	1	8
CWRF	1	1	8
CWRI	1	1	8
CYKH	1	1	8
CYMW	1	1	8
CYQQ	1	1	8
CAHQ	1	1	9
CANH	1	1	9
CAPC	1	1	9
CAQP	1	1	9
CAYC	1	1	9
CAYQ	1	1	9
CCAC	1	1	9

CCCW	1	1	9
CCLW	1	1	9
CEQH	1	1	9
CFAW	1	1	9
CFDH	1	1	9
CFDI	1	1	9
CFFC	1	1	9
CFFN	1	1	9
CFNC	1	1	9
CFWC	1	1	9
CFWV	1	1	9
CFYH	1	1	9
CGWF	1	1	9
CHCN	1	1	9
CHKW	1	1	9
CHMN	1	1	9
CHNH	1	1	9
CHNI	1	1	9
CHQQ	1	1	9
CICP	1	1	9
CIHF	1	1	9
CIHQ	1	1	9
CIWW	1	1	9
CIWY	1	1	9
CKCT	1	1	9
CMMN	1	1	9
CMQC	1	1	9
CMQN	1	1	9
CMWI	1	1	9
CMYC	1	1	9
CNQI	1	1	9
CQFF	1	1	9
CQPF	1	1	9
CSNQ	1	1	9
CSQP	1	1	9
CTWH	1	1	9
CWCG	1	1	9
CWFA	1	1	9
CWFC	1	1	9
CWFV	1	1	9
CWHA	1	1	9
CWIE	1	1	9
CWMW	1	1	9
CWTN	1	1	9
CWTW	1	1	9
CWVF	1	1	9
CYCE	1	1	9
CYMG	1	1	9
CYWI	1	1	9
CAQH	1	1	10
CAWH	1	1	10
CDFW	1	1	10
CDWK	1	1	10
CGMF	1	1	10

CGQQ	1	1	10
CHIW	1	1	10
CHKI	1	1	10
CHME	1	1	10
CIGW	1	1	10
CIQH	1	1	10
CMAA	1	1	10
CMEW	1	1	10
CMFK	1	1	10
CMKV	1	1	10
CMNY	1	1	10
CMWH	1	1	10
CNGC	1	1	10
CNQT	1	1	10
CPYI	1	1	10
CQFL	1	1	10
CQHW	1	1	10
CQHY	1	1	10
CQKC	1	1	10
CQPP	1	1	10
CQQF	1	1	10
CQQH	1	1	10
CQWA	1	1	10
CQWN	1	1	10
CRNW	1	1	10
CRQP	1	1	10
CVQF	1	1	10
CWEY	1	1	10
CWFT	1	1	10
CWYF	1	1	10
CYCH	1	1	10
CYFP	1	1	10
CYHC	1	1	10
CYID	1	1	10
CYLF	1	1	10
CADY	1	1	11
CAFW	1	1	11
CANN	1	1	11
CEPK	1	1	11
CFHD	1	1	11
CFIE	1	1	11
CFIW	1	1	11
CFKY	1	1	11
CFNP	1	1	11
CFWM	1	1	11
CGWC	1	1	11
CHPF	1	1	11
CHYN	1	1	11
CIAW	1	1	11
CIRF	1	1	11
CIYA	1	1	11
CKWC	1	1	11
CLFY	1	1	11
CMHG	1	1	11

CMMV	1	1	11
CMRF	1	1	11
CMWN	1	1	11
CMYY	1	1	11
CNMH	1	1	11
CPCC	1	1	11
CPMQ	1	1	11
CPQN	1	1	11
CPYH	1	1	11
CQCY	1	1	11
CQFY	1	1	11
CQHQ	1	1	11
CQPQ	1	1	11
CQPT	1	1	11
CQTH	1	1	11
CSWM	1	1	11
CTHM	1	1	11
CVHY	1	1	11
CVQQ	1	1	11
CWAC	1	1	11
CWIY	1	1	11
CWNH	1	1	11
CWVP	1	1	11
CWWH	1	1	11
CYFC	1	1	11
CYYN	1	1	11
CYYQ	1	1	11
CAPQ	1	1	12
CCCK	1	1	12
CEPE	1	1	12
CEYC	1	1	12
CFHT	1	1	12
CFLD	1	1	12
CGEC	1	1	12
CHHM	1	1	12
CHLY	1	1	12
CHPM	1	1	12
CHTH	1	1	12
CIFF	1	1	12
CKNK	1	1	12
CKWK	1	1	12
CKYW	1	1	12
CMFY	1	1	12
CMWG	1	1	12
CMYN	1	1	12
CPFW	1	1	12
CPQQ	1	1	12
CQAF	1	1	12
CQPV	1	1	12
CQPW	1	1	12
CRKC	1	1	12
CSCW	1	1	12
CTCK	1	1	12
CVFY	1	1	12

CWFD	1	1	12
CWSF	1	1	12
CWWQ	1	1	12
CWYT	1	1	12
CYAM	1	1	12
CYQH	1	1	12
CYRF	1	1	12
CYYC	1	1	12
CCFG	1	1	13
CDHW	1	1	13
CFSY	1	1	13
CGYQ	1	1	13
CHPW	1	1	13
CHQW	1	1	13
CHVF	1	1	13
CHYM	1	1	13
CIKG	1	1	13
CIWF	1	1	13
CKFV	1	1	13
CKHM	1	1	13
CKPQ	1	1	13
CPKI	1	1	13
CPMF	1	1	13
CPWN	1	1	13
CQWY	1	1	13
CRQH	1	1	13
CWIR	1	1	13
CWLW	1	1	13
CWPW	1	1	13
CWQL	1	1	13
CWVW	1	1	13
CYHW	1	1	13
CYKK	1	1	13
CYMF	1	1	13
CYNY	1	1	13
CYRI	1	1	13
CYTF	1	1	13
CYVN	1	1	13
CANP	1	1	14
CCGW	1	1	14
CDYH	1	1	14
CFHA	1	1	14
CHMQ	1	1	14
CHRY	1	1	14
CIQR	1	1	14
CIWN	1	1	14
CKDW	1	1	14
CKMY	1	1	14
CMCY	1	1	14
CMSF	1	1	14
CQFQ	1	1	14
CTNY	1	1	14
CVNQ	1	1	14
CWFP	1	1	14

CYFK	1	1	14
CAIW	1	1	15
CCFW	1	1	15
CEVQ	1	1	15
CFHM	1	1	15
CFMV	1	1	15
CFNI	1	1	15
CFPF	1	1	15
CHMK	1	1	15
CHNQ	1	1	15
CIEQ	1	1	15
CKYY	1	1	15
CRWH	1	1	15
CTQY	1	1	15
CVYC	1	1	15
CVYW	1	1	15
CWPV	1	1	15
CWWT	1	1	15
CYDK	1	1	15
CYFV	1	1	15
CADN	1	1	16
CAQM	1	1	16
CATH	1	1	16
CAYW	1	1	16
CCWY	1	1	16
CDYF	1	1	16
CFTP	1	1	16
CHCT	1	1	16
CHQM	1	1	16
CHYD	1	1	16
CKTM	1	1	16
CLFF	1	1	16
CMEY	1	1	16
CMWL	1	1	16
CPHQ	1	1	16
CRAF	1	1	16
CSFI	1	1	16
CTPQ	1	1	16
CVYQ	1	1	16
CWHG	1	1	16
CWKR	1	1	16
CYYI	1	1	16
CCDN	1	1	17
CFHL	1	1	17
CFYI	1	1	17
CHAM	1	1	17
CIDF	1	1	17
CLHC	1	1	17
CLWW	1	1	17
CMPW	1	1	17
CMWP	1	1	17
CNVW	1	1	17
CNYY	1	1	17
CQPI	1	1	17

CSMW	1	1	17
CTIW	1	1	17
CVWN	1	1	17
CWLF	1	1	17
CAAQ	1	1	18
CCWS	1	1	18
CGGW	1	1	18
CHNK	1	1	18
CKHL	1	1	18
CKYC	1	1	18
CPFT	1	1	18
CPRW	1	1	18
CPWA	1	1	18
CPWG	1	1	18
CRQF	1	1	18
CRYW	1	1	18
CSIW	1	1	18
CTWN	1	1	18
CVWF	1	1	18
CYSK	1	1	18
CAWV	1	1	19
CHDM	1	1	19
CKPV	1	1	19
CKWA	1	1	19
CRNK	1	1	19
CWCT	1	1	19
CWWF	1	1	19
CAPM	1	1	20
CCSF	1	1	20
CDLW	1	1	20
CKQR	1	1	20
CVFQ	1	1	20
CWPY	1	1	20
CWRG	1	1	20
CYWL	1	1	20
CIYS	1	1	21
CLFW	1	1	21
CQPR	1	1	21
CRWI	1	1	21
CTYP	1	1	21
CWQY	1	1	21
CNRH	1	1	22
CQNK	1	1	22
CWHM	1	1	22
CYIN	1	1	22
CCAA	1	1	23
CETM	1	1	23
CGWP	1	1	23
CNRE	1	1	23
CYMH	1	1	23
CVFW	1	1	24
CAQT	1	1	26
CKFS	1	1	26
CPLH	1	1	26

CRYM	1	1	26
CVYA	1	1	26
CYHR	1	1	26
CLWH	1	1	27
CITY	1	1	28
CNQL	1	1	30
CLWM	1	1	31
CSPM	1	1	39
CQTM	1	1	40
CTLW	1	1	47
