# Supplementary material for: Towards the Systematic Mapping and Engineering of the Protein Prenylation Machinery in Saccharomyces cerevisiae
Source: PLoS One. 2015 Mar 13;10(3):e0120716. doi: 10.1371/journal.pone.0120716 (PMC4358939; doi:10.1371/journal.pone.0120716)
Supplement: S4 File — (PDF) [file pone.0120716.s004.pdf]

Distribution of CaaX-Box Motives in the Naïve and Three Independently Simulated Libraries

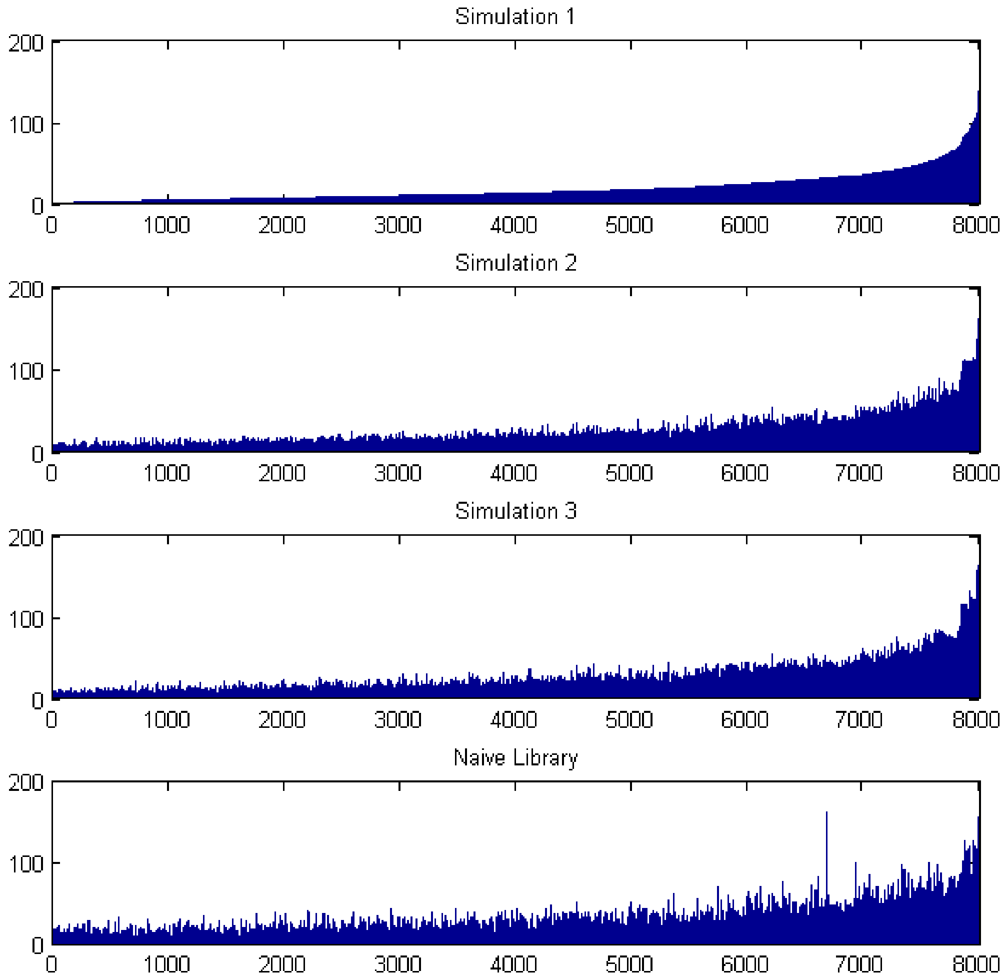

Figure S1: In the naïve library, the count of 8000 theoretically possible individual CaaX-box motifs ranges from 0 to 153 within a total of 168,627 of NGS counts. This relatively large spread in the occurrence of individual CaaX-box motives in the naïve library primarily arises from the different codon frequencies encoded by the degenerate NNS codons as three independently simulated CaaX-box libraries yield distributions that are comparable to the naïve library. Here, on average, the normalized fold-deviation of different CaaX-box motives in Simulation 1 compared to Simulation 2 and 3 differs by 9% while Simulation 1 compared to the naïve library (for CaaX-box motives occurring more than twice) differs by 28%.
