# Supplementary material for: Towards the Systematic Mapping and Engineering of the Protein Prenylation Machinery in Saccharomyces cerevisiae
Source: PLoS One. 2015 Mar 13;10(3):e0120716. doi: 10.1371/journal.pone.0120716 (PMC4358939; doi:10.1371/journal.pone.0120716)
Supplement: S5 File — (PDF) [file pone.0120716.s005.pdf]

Correlating Putative CAAX-box Motives with Endogenous Protein Functions

- Screening was performed in the context of the Hypervariable Region of H-Ras:

```
   170              175              180              185
... K  L  N  P  P  D  E  S  G  P  G  C  M  S  C  K  C  X  X  X
```

- Endogenous CaaX-box motives were identified with the pattern match tool (http://www.yeastgenome.org/cgi-bin/PATMATCH/nph-patmatch) at the Saccharomyces Genome Database using query 'CXXX>'.

High Confidence Set: Enrichments > 3

Motif	Enrichment	37 °C	25 °C	Naïve	Gene ID	Protein Function
-CFIF	3.1940	8	1	4	YBR042C	Putative transferase involved in phospholipid biosynthesis
-CVIM	3.3714	152	18	19	YBL061C	Activator of Chs3p (chitin synthase III) during vegetative growth
-CAIM	4.3303	141	13	13	YML006C	CAAX box containing protein of unknown function
-CVVM	5.1482	245	19	16	YPL191C	Putative protein of unknown function
-CIII	5.3898	162	12	16	YIR032C	Ureidoglycolate lyase
-CVIS	5.6350	494	35	53	YIR007W	Putative glycosidase
-CTIL	6.2198	592	38	72	YGR152C	GTP-binding protein of the Ras superfamily
-CSIM	6.4349	274	17	20	YCR027C	Putative Rheb-related GTPase
-CCVC	6.5523	279	17	19	YFL066C	Helicase-like protein encoded within the telomeric Y' element
-CTVA	7.7758	409	21	36	YML116W	Multidrug efflux pump of the major facilitator superfamily
-CCIQ	7.8138	137	7	15	YLR090W	Chaperone with a role in facilitating mitochondrial protein import
-CTIM	8.1320	387	19	19	YIL118W	Non-essential small GTPase of the Rho/Rac family of Ras-like proteins
-CVIL	8.9122	692	31	51	YNL180C	Non-essential small GTPase of the Rho/Rac family of Ras-like proteins
-CVIA	10.123	355	14	40	YDR461W	Mating pheromone a-factor
-CIIL	10.313	620	24	35	YOL014W	Putative protein of unknown function
-CIIM	15.670	157	4	12	YKR055W	Non-essential small GTPase
-CAIL	17.088	642	15	40	YLR229C	Small rho-like GTPase

Ambiguous Set: Enrichments 1-3 + Low Occurrences at 37 °C and 25 °C

-CCIM	1.1977	3	1	9	YJL204C	F-box protein involved in recycling endocytosed proteins
-CIHH	1.1977	3	1	16	YOL164W-A	Putative protein of unknown function

Low Confidence Set: Enrichments < 1

-CCLD	0.0240	1	16	40	YBR087W	Subunit of heteropentameric Replication factor C (RF-C)
-CQRK	0.0240	1	16	22	YOR034C-A	Putative protein of unknown function
-CRNR	0.0240	1	16	42	YML041C	Nucleosome-binding component of the SWR1 complex
-CALD	0.0285	1	14	36	YJL062W	Integral plasma membrane protein
-CTDS	0.0319	2	25	43	YOR257W	Calcium-binding component of the spindle pole body (SPB) half-bridge
-CIKS	0.0347	2	23	39	YML075C	HMG-CoA reductase
-CTRK	0.0363	1	11	39	YDR528W	Protein involved in regulation of cell wall composition and integrity
-CLAK	0.0399	2	20	34	YDR307W	Putative protein mannosyltransferase similar to Pmt1p
-CSNA	0.0399	1	10	32	YMR265C	Putative protein of unknown function
-CSGL	0.0413	3	29	77	YJR107W	Putative lipase
-CRVK	0.0499	2	16	33	YMR158W	Mitochondrial ribosomal protein of the small subunit
-CRME	0.0570	1	7	11	YJL059W	Protein required for the ATP-dependent transport of arginine
-CCPS	0.0614	2	13	23	YFL065C	Putative protein of unknown function
-CKQS	0.0665	1	6	8	YKR048C	Histone chaperone
-CVKK	0.0665	2	12	33	YDR257C	Ribosomal lysine methyltransferase
-CVKM	0.0665	1	6	17	YBR150C	Putative protein of unknown function
-CDDD	0.0798	1	5	8	YGR068C	Protein proposed to regulate endocytosis of plasma membrane proteins
-CWKD	0.0798	2	10	15	YOR231W	MAPKK involved in the protein kinase C signaling pathway
-CYNA	0.0798	1	5	19	YCR020C	Protein of unknown function
-CNAG	0.0887	2	9	18	YKL176C	Protein possibly involved in a post-Golgi secretory pathway
-CASL	0.0939	8	34	74	YDR261C	"Exo-1,3-beta-glucanase"
-CCCS	0.0998	2	8	24	YJL118W	Putative protein of unknown function
-CDFS	0.0998	3	12	32	YGR282C	"Endo-beta-1,3-glucanase"
-CHHD	0.0998	1	4	11	YDL186W	Putative protein of unknown function
-CPNY	0.0998	1	4	13	YPL109C	Putative protein of unknown function
-CVFK	0.0998	1	4	27	YKL069W	Methionine-R-sulfoxide reductase
-CYDA	0.0998	2	8	13	YOR299W	Member of the ChAPs family (Chs5p-Arf1p-binding proteins)
-CDPN	0.1140	2	7	28	YNL106C	Polyphosphatidylinositol phosphatase
-CQGK	0.1140	2	7	17	YDR301W	RNA-binding subunit of the mRNA cleavage and polyadenylation factor
-CVLL	0.1159	9	31	128	YPR165W	GTP-binding protein of the rho subfamily of Ras-like proteins
-CHDE	0.1331	1	3	8	YPR093C	Ubiquitin ligase that modifies and regulates RNA Pol II
-CPKA	0.1331	1	3	17	YNL255C	Translational activator for mRNAs with internal ribosome entry sites
-CSEI	0.1497	6	16	16	YBR096W	Putative protein of unknown function
-CAPY	0.1996	1	2	15	YIL134C-A	Putative protein of unknown function
-CIGK	0.1996	1	2	9	YMR023C	Mitochondrial protein
-CIQF	0.1996	1	2	13	YGL169W	Protein involved in threonylcarbamoyl adenosine biosynthesis
-CKCI	0.1996	1	2	6	YBL018C	Subunit of both RNase MRP and nuclear RNase P
-CNDV	0.1996	1	2	18	YGL263W	Protein of unknown function
-CIDL	0.2541	7	11	36	YOR242C	Sporulation specific protein that localizes to the spore wall
-CDDY	0.2662	2	3	11	YGL045W	Protein involved in proteolytic activation of Rim101p
-CKGE	0.2662	2	3	5	YMR187C	Putative protein of unknown function
-CASQ	0.3992	3	3	37	YNL064C	Type I HSP40 co-chaperone
-CMYV	0.7186	9	5	25	YMR272W-B	Protein of unknown function

Unconfirmed Motives: **Neither Observed in the 37 °C or 25 °C Library Sets → Lack of Transformants?**

-CADY	0.3992	1	1	11	YMR300C	Phosphoribosylpyrophosphate amidotransferase (PRPPAT)
-CKCT	0.3993	1	1	9	YBL049W	Protein of unknown function
-CKQQ	0.3993	1	1	8	YDL065C	Chaperone and import receptor for newly-synthesized class I PMPs
-CKYI	0.3993	1	1	3	YMR060C	Component of the Sorting and Assembly Machinery (SAM) complex
-CPFW	0.3993	1	1	12	YJR066W	PIK-related protein kinase and rapamycin target
