# Supplementary material for: Towards the Systematic Mapping and Engineering of the Protein Prenylation Machinery in Saccharomyces cerevisiae
Source: PLoS One. 2015 Mar 13;10(3):e0120716. doi: 10.1371/journal.pone.0120716 (PMC4358939; doi:10.1371/journal.pone.0120716)
Supplement: S6 File — (PDF) [file pone.0120716.s006.pdf]

1. Enrichment Factors of Sequencing Motives Ending with -LL

Motif	Enrichment	Counts		
		37 °C	25 °C	Naive
-CPLL	0.02218	2	36	83
-CKLL	0.027534	2	29	65
-CQLL	0.057035	2	14	36
-CTLL	0.061422	2	13	102
-CDLL	0.074278	8	43	101
-CSLL	0.075533	7	37	150
-CFLL	0.099811	2	8	64
-CGLL	0.099811	8	32	71
-CVLL	0.11591	9	31	128
-CNLL	0.12284	4	13	53
-CALL	0.15526	7	18	103
-CLLL	0.19012	10	21	144
-CWLL	0.23955	3	5	39
-CELL	0.24398	11	18	53
-CRLL	0.29036	16	22	130
-CHLL	0.29418	14	19	60
-CMLL	0.29943	3	4	36
-CYLL	0.29943	12	16	68
-CCLL	0.3194	4	5	41
-CILL	0.3194	4	5	45

Note: -CVLL of Rho1p is highlighted in yellow

2. Enrichment Factors of Sequencing Motives Ending with -VL

Motif	Enrichment	Counts		
		37 °C	25 °C	Naive
-CGVL	0.03327	3	36	71
-CEVL	0.063879	4	25	58
-CRVL	0.063879	4	25	95
-CQVL	0.10506	5	19	39
-CMVL	0.11407	2	7	29
-CSVL	0.13688	12	35	93
-CYVL	0.14518	4	11	56
-CLVL	0.14639	11	30	93
-CKVL	0.14972	3	8	36
-CDVL	0.1597	8	20	40
-CFVL	0.1597	2	5	40
-CPVL	0.19962	12	24	57
-CHVL	0.28182	12	17	86
-CWVL	0.39925	2	2	18
-CAVL	0.41826	22	21	59
-CCVL	0.58351	19	13	38
-CNVL	1.0188	74	29	51
-CIVL	3.3366	234	28	57
-CVVL	5.2953	504	38	98
-CTVL	5.4896	550	40	110

3. Enrichment Factors of Sequencing Motives Ending with -IL

		Counts		
**Motif**	**Enrichment**	**37 °C**	**25 °C**	**Naive**
-CWIL	0.26616	2	3	15
-CKIL	0.34221	6	7	25
-CGIL	0.35488	8	9	26
-CRIL	0.6341	27	17	51
-CDIL	1.9004	119	25	28
-CFIL	1.9962	5	1	14
-CYIL	2.3755	119	20	31
-CNIL	4.9011	356	29	18
-CEIL	5.0305	315	25	23
-CTIL	6.2198	592	38	72
-CQIL	7.7454	194	10	26
-CCIL	8.6237	324	15	22
-CVIL	8.9122	692	31	51
-CPIL	9.0914	797	35	45
-CMIL	9.2326	185	8	26
-CSIL	10.0118	1304	52	64
-CIIL	10.3138	620	24	35
-CHIL	11.1789	84	3	21
-CAIL	17.0877	642	15	40
-CLIL	17.7034	1685	38	66

Note: -CTIL of Cdc42p and –CIIL of Ras2p are highlighted in yellow
