# Supplementary material for: Towards the Systematic Mapping and Engineering of the Protein Prenylation Machinery in Saccharomyces cerevisiae
Source: PLoS One. 2015 Mar 13;10(3):e0120716. doi: 10.1371/journal.pone.0120716 (PMC4358939; doi:10.1371/journal.pone.0120716)
Supplement: S7 File — (PDF) [file pone.0120716.s007.pdf]

1. Enrichment Factors of GGTase II Compatible CAAX-box Motives: -CCXC Pattern

		Counts		
Motif	Enrichment	37 °C	25 °C	Naive
-CCGC	0.099811	1	4	22
-CCQC	0.099811	1	4	21
-CCSC	0.099811	1	4	6
-CCEC	0.1597	2	5	15
-CCRC	0.1597	2	5	16
-CCFC	0.19962	3	6	14
-CCTC	0.19962	1	2	10
-CCWC	0.19962	1	2	6
-CCYC	0.19962	1	2	8
-CCNC	0.26616	2	3	12
-CCAC	0.39925	1	1	9
-CCDC	0.39925	1	1	5
-CCKC	0.39925	1	1	8
-CCCC	0.79849	2	1	6
-CCHC	0.79849	2	1	5
-CCPC	0.79849	2	1	9
-CCVC	6.5523	279	17	19
-CCLC	9.4868	499	21	34
-CCIC	14.4726	145	4	14
-CCMC	14.6922	368	10	5

2. Enrichment Factors of GGTase II Compatible CAAX-box Motives: -CXCC Pattern

		Counts		
Motif	Enrichment	37 °C	25 °C	Naive
-CFCC	0.19962	1	2	3
-CKCC	0.19962	1	2	18
-CECC	0.26616	2	3	20
-CACC	0.34221	6	7	9
-CDCC	0.39925	1	1	6
-CHCC	0.39925	1	1	6
-CMCC	0.39925	1	1	2
-CNCC	0.39925	1	1	5
-CPCC	0.39925	1	1	11
-CQCC	0.39925	1	1	8
-CWCC	0.39925	1	1	5
-CYCC	0.39925	2	2	8
-CGCC	0.59887	3	2	10
-CCCC	0.79849	2	1	6
-CRCC	0.79849	2	1	10
-CSCC	0.79849	4	2	19
-CTCC	1.4639	11	3	14
-CICC	1.9962	5	1	7
-CVCC	11.2787	113	4	4
-CLCC	13.8671	521	15	11
