# Supplementary material for: Towards the Systematic Mapping and Engineering of the Protein Prenylation Machinery in Saccharomyces cerevisiae
Source: PLoS One. 2015 Mar 13;10(3):e0120716. doi: 10.1371/journal.pone.0120716 (PMC4358939; doi:10.1371/journal.pone.0120716)
Supplement: S8 File — (PDF) [file pone.0120716.s008.pdf]

1. Enrichment Factors of Sequencing Motives Ending with –CXID/E

Motif	Enrichment	Counts		
		37 °C	25 °C	Naive
-CSIE	0.019962	1	20	33
-CNIE	0.03327	1	12	8
-CHIE	0.044361	1	9	10
-CFID	0.057035	1	7	10
-CPIE	0.057035	1	7	20
-CGIE	0.061422	2	13	30
-CHID	0.066541	1	6	14
-CQIE	0.066541	1	6	12
-CMIE	0.079849	2	10	10
-CEIE	0.088721	2	9	9
-CDIE	0.099811	1	4	13
-CGID	0.099811	1	4	9
-CKIE	0.099811	1	4	4
-CNID	0.099811	2	8	16
-CPID	0.099811	1	4	7
-CSID	0.099811	2	8	30
-CYIE	0.099811	1	4	8
-CDID	0.13308	1	3	7
-CLID	0.13308	1	3	12
-CQID	0.13308	1	3	11
-CWID	0.13308	1	3	5
-CCID	0.14972	3	8	13
-CAIE	0.17744	4	9	20
-CRID	0.19962	1	2	18
-CRIE	0.19962	2	4	22
-CAID	0.29943	3	4	16
-CCIE	0.34221	6	7	14
-CEID	0.39925	1	1	5
-CFIE	0.39925	1	1	11
-CLIE	0.39925	2	2	5
-CWIE	0.39925	1	1	9
-CYID	0.39925	1	1	10
-CKID	0.59887	3	2	6
-CTID	0.62739	11	7	10
-CMID	1.1977	3	1	13
-CIIE	1.4639	11	3	5
-CTIE	2.8945	58	8	16
-CIID	5.0904	51	4	18
-CVIE	5.6054	351	25	41
-CVID	15.0116	376	10	18

Note:β-branched in A1 are highlighted in yellow

2. Enrichment Factors of Sequencing Motives Ending with –CXXD/E

		Counts		
**Motif**	**Enrichment**	**37 °C**	**25 °C**	**Naive**
-CLTE	0.012879	1	31	46
-CVRE	0.015356	1	26	26
-CYED	0.017359	1	23	16
-CSRD	0.019012	1	21	34
-CLSE	0.019475	2	41	50
-CSIE	0.019962	1	20	33
-CCTE	0.021013	1	19	29
-CEVD	0.02218	1	18	6
-CSNE	0.02218	1	18	15
-CSTE	0.02218	1	18	23
-CSVD	0.023061	16	277	162
-CISD	0.023485	1	17	28
-CCLD	0.024953	1	16	40
-CKTE	0.024953	1	16	20
-CVQE	0.024953	1	16	15
-CVSE	0.025758	2	31	48
-CEFD	0.026616	1	15	11
-CFPE	0.026616	1	15	12
-CALD	0.028518	1	14	36
-CAME	0.028518	1	14	18
-CDEE	0.028518	1	14	5
-CDLD	0.028518	1	14	18
-CNSD	0.028518	1	14	22
-CPDE	0.028518	1	14	16
-CPNE	0.028518	1	14	16
-CPRE	0.028518	1	14	36
-CTRE	0.028518	1	14	31
-CVTD	0.028518	1	14	22
-CDGE	0.030711	1	13	15
-CELE	0.030711	1	13	22
-CESE	0.030711	1	13	20
-CFQE	0.030711	1	13	14
-CGTE	0.030711	1	13	16
-CHSE	0.030711	1	13	25
-CKLE	0.030711	1	13	23
-CLTD	0.030711	1	13	42
-CYEE	0.030711	1	13	14
-CANE	0.03327	1	12	12
-CGGE	0.03327	1	12	8
-CGMD	0.03327	1	12	15
-CIGE	0.03327	1	12	11
-CKDE	0.03327	1	12	21
-CKLD	0.03327	1	12	25
-CLNE	0.03327	1	12	23
-CNIE	0.03327	1	12	8
-CNTE	0.03327	1	12	30
-CPKE	0.03327	1	12	21
-CRAE	0.03327	1	12	28
-CRND	0.03327	1	12	15
-CRNE	0.03327	1	12	39
-CPSE	0.034717	2	23	38

-CPTE	0.034717	2	23	21
-CAPE	0.036295	1	11	17
-CARE	0.036295	1	11	20
-CCDE	0.036295	1	11	12
-CDSE	0.036295	1	11	18
-CERD	0.036295	1	11	22
-CEYE	0.036295	1	11	12
-CGFD	0.036295	1	11	19
-CGGD	0.036295	1	11	11
-CNDE	0.036295	1	11	19
-CNTD	0.036295	2	22	15
-CPLD	0.036295	1	11	24
-CRCE	0.036295	1	11	24
-CRYD	0.036295	1	11	39
-CTGE	0.036295	1	11	18
-CTSD	0.036295	2	22	32
-CVAD	0.036295	1	11	22
-CGLD	0.038023	2	21	43
-CASE	0.039925	1	10	29
-CCGE	0.039925	1	10	23
-CDME	0.039925	2	20	17
-CGVD	0.039925	1	10	21
-CHGD	0.039925	1	10	9
-CHTE	0.039925	1	10	20
-CLFD	0.039925	1	10	22
-CLKD	0.039925	1	10	12
-CMSE	0.039925	2	20	34
-CMTE	0.039925	1	10	20
-CPHE	0.039925	1	10	16
-CSEE	0.039925	1	10	15
-CSKD	0.039925	2	20	18
-CTGD	0.039925	1	10	6
-CYLE	0.039925	1	10	29
-CYRE	0.039925	1	10	25
-CQTE	0.041301	3	29	17
-CESD	0.042026	2	19	21
-CGEE	0.042026	2	19	13
-CGLE	0.042026	2	19	43
-CLAE	0.042026	2	19	19
-CRTE	0.042026	2	19	25
-CSGE	0.042026	2	19	41
-CVRD	0.042026	2	19	30
-CAFD	0.044361	1	9	23
-CALE	0.044361	1	9	29
-CDCD	0.044361	2	18	8
-CDDE	0.044361	1	9	14
-CGND	0.044361	1	9	11
-CHIE	0.044361	1	9	10
-CHRE	0.044361	1	9	29
-CIHD	0.044361	1	9	20
-CITE	0.044361	2	18	11
-CLGD	0.044361	1	9	20
-CLPE	0.044361	2	18	32
-CNRD	0.044361	2	18	19
-CSLD	0.044361	2	18	47
-CTED	0.044361	2	18	29
-CTTD	0.044361	1	9	12

-CVEE	0.044361	1	9	16
-CRRD	0.044361	3	27	46
-CGSE	0.04697	2	17	24
-CLFE	0.04697	2	17	31
-CRFE	0.04697	2	17	35
-CDNE	0.049906	1	8	16
-CDRE	0.049906	1	8	18
-CDTE	0.049906	1	8	12
-CECE	0.049906	1	8	15
-CEGD	0.049906	2	16	3
-CEHE	0.049906	1	8	13
-CERE	0.049906	1	8	22
-CFND	0.049906	1	8	11
-CGPD	0.049906	1	8	12
-CHCD	0.049906	1	8	11
-CHED	0.049906	1	8	3
-CIPD	0.049906	1	8	10
-CKCD	0.049906	1	8	18
-CKEE	0.049906	1	8	5
-CKMD	0.049906	1	8	10
-CKNE	0.049906	1	8	20
-CLCE	0.049906	1	8	21
-CLEE	0.049906	2	16	23
-CLQD	0.049906	2	16	19
-CMSD	0.049906	1	8	14
-CNVD	0.049906	1	8	16
-CNYE	0.049906	1	8	25
-CPLE	0.049906	3	24	38
-CQSD	0.049906	1	8	18
-CQVE	0.049906	1	8	11
-CRKE	0.049906	1	8	24
-CSDE	0.049906	3	24	25
-CVAE	0.049906	2	16	30
-CVND	0.049906	1	8	12
-CWSE	0.049906	1	8	18
-CYTD	0.049906	1	8	12
-CHEE	0.053233	2	15	13
-CLLD	0.053233	2	15	50
-CSPD	0.053233	2	15	26
-CVGE	0.053233	2	15	21
-CTSE	0.054443	3	22	49
-CECD	0.057035	1	7	9
-CEKE	0.057035	1	7	7
-CFCE	0.057035	1	7	8
-CFID	0.057035	1	7	10
-CGYE	0.057035	1	7	16
-CHAE	0.057035	1	7	12
-CHSD	0.057035	1	7	22
-CKPE	0.057035	1	7	6
-CNLE	0.057035	1	7	9
-CPIE	0.057035	1	7	20
-CPKD	0.057035	1	7	25
-CPVE	0.057035	1	7	13
-CQGD	0.057035	1	7	9
-CRGD	0.057035	2	14	12
-CRGE	0.057035	1	7	22
-CRME	0.057035	1	7	11

-CRQD	0.057035	1	7	13
-CRQE	0.057035	1	7	12
-CRYE	0.057035	1	7	14
-CSAD	0.057035	2	14	30
-CSDD	0.057035	2	14	13
-CSGD	0.057035	2	14	28
-CSHD	0.057035	1	7	16
-CSME	0.057035	1	7	24
-CELD	0.057035	3	21	31
-CGIE	0.061422	2	13	30
-CGQE	0.061422	2	13	13
-CPFD	0.061422	2	13	7
-CPRD	0.061422	2	13	23
-CTCD	0.061422	2	13	14
-CTFE	0.061422	4	26	27
-CEYD	0.063039	3	19	10
-CLHE	0.063039	3	19	39
-CAAE	0.066541	1	6	19
-CADE	0.066541	1	6	21
-CAVD	0.066541	1	6	29
-CCAD	0.066541	1	6	15
-CCED	0.066541	2	12	8
-CDAE	0.066541	1	6	7
-CDFD	0.066541	1	6	12
-CEED	0.066541	1	6	10
-CEVE	0.066541	1	6	16
-CFCD	0.066541	1	6	14
-CFSE	0.066541	2	12	19
-CGKD	0.066541	1	6	9
-CGRE	0.066541	1	6	14
-CHID	0.066541	1	6	14
-CHLE	0.066541	1	6	22
-CHTD	0.066541	2	12	27
-CIEE	0.066541	1	6	11
-CIQE	0.066541	1	6	13
-CIRE	0.066541	1	6	14
-CKAD	0.066541	1	6	7
-CKKD	0.066541	1	6	7
-CKQD	0.066541	2	12	14
-CKQE	0.066541	1	6	13
-CKVE	0.066541	1	6	18
-CLDD	0.066541	1	6	20
-CLQE	0.066541	1	6	4
-CLRE	0.066541	2	12	30
-CLYD	0.066541	1	6	31
-CMLD	0.066541	1	6	33
-CMPD	0.066541	1	6	9
-CNEE	0.066541	1	6	10
-CNHD	0.066541	1	6	10
-CNHE	0.066541	2	12	18
-CNKD	0.066541	1	6	8
-CNND	0.066541	1	6	8
-CPFE	0.066541	1	6	17
-CQIE	0.066541	1	6	12
-CQLD	0.066541	1	6	12
-CQNE	0.066541	1	6	10
-CRDE	0.066541	1	6	22

-CSFE	0.066541	1	6	8
-CTHD	0.066541	1	6	17
-CTPE	0.066541	2	12	34
-CTRD	0.066541	2	12	24
-CVSD	0.066541	1	6	19
-CVTE	0.066541	2	12	46
-CVYD	0.066541	1	6	15
-CWNE	0.066541	1	6	9
-CWSD	0.066541	1	6	9
-CYKE	0.066541	1	6	3
-CYLD	0.066541	1	6	29
-CGSD	0.066541	3	18	29
-CTLD	0.066541	3	18	45
-CYPD	0.066541	3	18	32
-CSMD	0.070455	3	17	25
-CASD	0.07259	2	11	26
-CCHE	0.07259	2	11	4
-CLED	0.07259	2	11	16
-CLGE	0.07259	2	11	30
-CMTD	0.07259	2	11	24
-CMVE	0.07259	2	11	14
-CTQE	0.07259	2	11	9
-CVDE	0.07259	2	11	17
-CLLE	0.074859	3	16	39
-CTTE	0.076047	4	21	47
-CAEE	0.079849	2	10	18
-CAFE	0.079849	1	5	15
-CAMD	0.079849	1	5	10
-CCYE	0.079849	1	5	3
-CDDD	0.079849	1	5	8
-CDHD	0.079849	1	5	9
-CDWE	0.079849	1	5	7
-CDYE	0.079849	1	5	14
-CEAD	0.079849	1	5	11
-CETD	0.079849	3	15	25
-CFAE	0.079849	1	5	12
-CFGD	0.079849	1	5	11
-CFGE	0.079849	1	5	3
-CGAE	0.079849	1	5	11
-CHGE	0.079849	1	5	6
-CHMD	0.079849	1	5	8
-CIAE	0.079849	1	5	18
-CIKE	0.079849	1	5	8
-CITD	0.079849	1	5	10
-CIYE	0.079849	2	10	7
-CKME	0.079849	1	5	7
-CKSD	0.079849	1	5	25
-CKYD	0.079849	1	5	7
-CLHD	0.079849	1	5	20
-CLVE	0.079849	3	15	38
-CMDD	0.079849	2	10	10
-CMFE	0.079849	1	5	12
-CMIE	0.079849	2	10	10
-CMMD	0.079849	2	10	22
-CMRE	0.079849	1	5	18
-CNAE	0.079849	1	5	10
-CNGE	0.079849	1	5	16

-CNPE	0.079849	1	5	10
-CNYD	0.079849	1	5	9
-CPMD	0.079849	2	10	15
-CPQE	0.079849	1	5	9
-CPYD	0.079849	1	5	20
-CQEE	0.079849	1	5	3
-CQKE	0.079849	1	5	9
-CQMD	0.079849	1	5	7
-CRAD	0.079849	3	15	44
-CRVE	0.079849	1	5	18
-CTAE	0.079849	1	5	27
-CTFD	0.079849	2	10	11
-CTKD	0.079849	1	5	9
-CTME	0.079849	3	15	22
-CTND	0.079849	2	10	14
-CTPD	0.079849	2	10	10
-CTVE	0.079849	2	10	30
-CVDD	0.079849	1	5	14
-CVGD	0.079849	1	5	15
-CVLD	0.079849	2	10	24
-CVQD	0.079849	1	5	18
-CWKD	0.079849	2	10	15
-CWLD	0.079849	1	5	30
-CWPE	0.079849	2	10	11
-CWQD	0.079849	1	5	15
-CWQE	0.079849	2	10	4
-CWTE	0.079849	1	5	13
-CYAE	0.079849	2	10	15
-CYHE	0.079849	1	5	6
-CYPE	0.079849	1	5	6
-CYTE	0.079849	1	5	10
-CFDD	0.085553	3	14	15
-CSFD	0.085553	3	14	24
-CAQD	0.088721	2	9	11
-CEIE	0.088721	2	9	9
-CHPE	0.088721	2	9	8
-CIDD	0.088721	2	9	17
-CLAD	0.088721	2	9	21
-CNSE	0.088721	2	9	18
-CQSE	0.088721	2	9	19
-CSKE	0.088721	2	9	22
-CSLE	0.088721	4	18	77
-CSWE	0.088721	4	18	22
-CTKE	0.088721	2	9	22
-CYAD	0.088721	2	9	8
-CYSE	0.088721	4	18	20
-CLRD	0.088721	6	27	26
-CAKD	0.092134	3	13	31
-CHVE	0.092134	3	13	16
-CRTD	0.095058	5	21	35
-CAGD	0.099811	1	4	4
-CAGE	0.099811	2	8	30
-CAHE	0.099811	1	4	4
-CAKE	0.099811	1	4	8
-CATE	0.099811	3	12	21
-CAYE	0.099811	1	4	13
-CCDD	0.099811	1	4	7

-CCFE	0.099811	1	4	20
-CCKE	0.099811	1	4	15
-CCME	0.099811	1	4	9
-CCPE	0.099811	2	8	10
-CCSE	0.099811	1	4	22
-CCTD	0.099811	1	4	15
-CDIE	0.099811	1	4	13
-CDPE	0.099811	1	4	18
-CDQD	0.099811	2	8	2
-CDTD	0.099811	2	8	14
-CDVE	0.099811	1	4	13
-CEAE	0.099811	1	4	11
-CEEE	0.099811	1	4	4
-CEPD	0.099811	1	4	14
-CEWD	0.099811	1	4	3
-CFFE	0.099811	1	4	11
-CFNE	0.099811	1	4	11
-CFRD	0.099811	1	4	13
-CGCD	0.099811	1	4	9
-CGDE	0.099811	1	4	15
-CGID	0.099811	1	4	9
-CGME	0.099811	2	8	18
-CGVE	0.099811	1	4	16
-CGYD	0.099811	1	4	8
-CHCE	0.099811	1	4	8
-CHHD	0.099811	1	4	11
-CHND	0.099811	1	4	15
-CKIE	0.099811	1	4	4
-CKRD	0.099811	1	4	9
-CKRE	0.099811	2	8	8
-CLKE	0.099811	1	4	13
-CLMD	0.099811	1	4	28
-CMPE	0.099811	2	8	13
-CMYD	0.099811	1	4	11
-CNAD	0.099811	1	4	10
-CNID	0.099811	2	8	16
-CNMD	0.099811	1	4	11
-CNPD	0.099811	1	4	16
-CPCE	0.099811	1	4	12
-CPEE	0.099811	2	8	12
-CPGD	0.099811	2	8	10
-CPID	0.099811	1	4	7
-CPWE	0.099811	1	4	5
-CQDE	0.099811	1	4	9
-CQKD	0.099811	1	4	3
-CQME	0.099811	1	4	6
-CQVD	0.099811	1	4	20
-CRKD	0.099811	2	8	15
-CRLD	0.099811	1	4	25
-CRRE	0.099811	2	8	34
-CSID	0.099811	2	8	30
-CSQE	0.099811	1	4	17
-CSSE	0.099811	3	12	50
-CSTD	0.099811	3	12	27
-CTDD	0.099811	2	8	18
-CTLE	0.099811	3	12	42
-CVFE	0.099811	1	4	16

-CVNE	0.099811	1	4	20
-CVYE	0.099811	1	4	10
-CWCD	0.099811	1	4	11
-CWGE	0.099811	1	4	9
-CWKE	0.099811	1	4	6
-CWLE	0.099811	1	4	26
-CWTD	0.099811	1	4	13
-CYIE	0.099811	1	4	8
-CYKD	0.099811	1	4	13
-CYME	0.099811	1	4	12
-CYRD	0.099811	1	4	19
-CYWE	0.099811	1	4	3
-CLSD	0.10351	7	27	35
-CAVE	0.10889	3	11	18
-CLPD	0.10889	3	11	12
-CRCD	0.10889	3	11	25
-CRLE	0.10889	3	11	56
-CRSD	0.10889	3	11	45
-CSRE	0.10889	3	11	46
-CVPE	0.10889	3	11	38
-CAQE	0.11407	2	7	16
-CARD	0.11407	2	7	17
-CCPD	0.11407	2	7	18
-CDAD	0.11407	2	7	10
-CMYE	0.11407	2	7	19
-CNLD	0.11407	2	7	22
-CPVD	0.11407	2	7	18
-CRMD	0.11407	2	7	24
-CSCD	0.11407	2	7	12
-CSYE	0.11407	2	7	17
-CYGD	0.11407	2	7	17
-CRED	0.11977	3	10	17
-CRSE	0.11977	3	10	62
-CVCE	0.11977	3	10	21
-CAAD	0.13308	1	3	18
-CACE	0.13308	1	3	7
-CAHD	0.13308	2	6	7
-CCAE	0.13308	2	6	18
-CCKD	0.13308	1	3	8
-CCRE	0.13308	1	3	12
-CCYD	0.13308	1	3	12
-CDCE	0.13308	1	3	6
-CDID	0.13308	1	3	7
-CDKD	0.13308	1	3	9
-CDYD	0.13308	1	3	14
-CEND	0.13308	2	6	10
-CETE	0.13308	1	3	14
-CEWE	0.13308	1	3	3
-CFAD	0.13308	1	3	17
-CFHE	0.13308	1	3	9
-CFKD	0.13308	1	3	9
-CFMD	0.13308	1	3	4
-CFTD	0.13308	1	3	2
-CFYE	0.13308	1	3	13
-CGDD	0.13308	1	3	7
-CGKE	0.13308	1	3	15
-CGPE	0.13308	2	6	18

-CGWE	0.13308	1	3	9
-CHDE	0.13308	1	3	8
-CHKE	0.13308	1	3	8
-CIDE	0.13308	1	3	11
-CIKD	0.13308	1	3	9
-CIQD	0.13308	2	6	10
-CIYD	0.13308	1	3	4
-CKED	0.13308	2	6	6
-CKKE	0.13308	1	3	4
-CKND	0.13308	1	3	7
-CKSE	0.13308	2	6	15
-CKYE	0.13308	1	3	12
-CLID	0.13308	1	3	12
-CMAD	0.13308	2	6	9
-CMCE	0.13308	1	3	4
-CMED	0.13308	1	3	4
-CMEE	0.13308	2	6	11
-CMGE	0.13308	1	3	15
-CMHD	0.13308	1	3	6
-CMLE	0.13308	1	3	20
-CMVD	0.13308	1	3	5
-CMWE	0.13308	1	3	8
-CNME	0.13308	1	3	25
-CPED	0.13308	2	6	6
-CPHD	0.13308	1	3	5
-CPTD	0.13308	4	12	25
-CQAD	0.13308	1	3	3
-CQDD	0.13308	1	3	8
-CQGE	0.13308	2	6	10
-CQHD	0.13308	1	3	20
-CQID	0.13308	1	3	11
-CQRD	0.13308	1	3	17
-CQYD	0.13308	1	3	8
-CREE	0.13308	1	3	8
-CRFD	0.13308	1	3	18
-CSAE	0.13308	2	6	26
-CSND	0.13308	1	3	15
-CSPE	0.13308	1	3	30
-CSWD	0.13308	1	3	8
-CTDE	0.13308	1	3	5
-CTEE	0.13308	1	3	14
-CTNE	0.13308	2	6	18
-CTVD	0.13308	2	6	12
-CVCD	0.13308	1	3	13
-CVHE	0.13308	1	3	17
-CVKD	0.13308	2	6	10
-CVKE	0.13308	1	3	11
-CWCE	0.13308	1	3	9
-CWGD	0.13308	1	3	3
-CWID	0.13308	1	3	5
-CWVD	0.13308	1	3	5
-CYCD	0.13308	1	3	7
-CYFE	0.13308	1	3	8
-CYGE	0.13308	1	3	5
-CATD	0.13308	3	9	36
-CENE	0.13308	3	9	16
-CLYE	0.13308	3	9	28

-CRPD	0.13308	3	9	28
-CSVE	0.13308	6	18	42
-CVPD	0.13308	3	9	22
-CGAD	0.14091	6	17	9
-CEKD	0.14518	4	11	16
-CSHE	0.14709	7	19	32
-CCID	0.14972	3	8	13
-CDVD	0.14972	3	8	19
-CLME	0.14972	3	8	20
-CPAD	0.14972	3	8	23
-CSSD	0.14972	6	16	40
-CQRE	0.15356	5	13	10
-CRPE	0.15356	5	13	34
-CAND	0.1597	2	5	11
-CFPD	0.1597	2	5	13
-CFSD	0.1597	4	10	14
-CGCE	0.1597	2	5	13
-CGHD	0.1597	2	5	12
-CGWD	0.1597	2	5	7
-CISE	0.1597	2	5	20
-CLND	0.1597	2	5	20
-CNDD	0.1597	2	5	14
-CNFE	0.1597	2	5	4
-CRHD	0.1597	2	5	20
-CSCE	0.1597	2	5	13
-CWPD	0.1597	2	5	11
-CMRD	0.16635	5	12	23
-CPYE	0.16635	5	12	13
-CQTD	0.17111	3	7	14
-CYVE	0.17111	3	7	19
-CAIE	0.17744	4	9	20
-CVLE	0.18148	5	11	37
-CACD	0.19962	1	2	10
-CADD	0.19962	2	4	23
-CAWD	0.19962	1	2	17
-CAWE	0.19962	1	2	8
-CAYD	0.19962	2	4	17
-CCGD	0.19962	1	2	1
-CCMD	0.19962	1	2	2
-CCND	0.19962	1	2	3
-CCNE	0.19962	1	2	5
-CCQD	0.19962	1	2	6
-CCVD	0.19962	1	2	21
-CCWE	0.19962	1	2	4
-CDED	0.19962	1	2	6
-CDHE	0.19962	1	2	10
-CDKE	0.19962	2	4	16
-CDMD	0.19962	1	2	4
-CEFE	0.19962	1	2	4
-CEHD	0.19962	1	2	7
-CFDE	0.19962	1	2	6
-CFED	0.19962	1	2	1
-CFEE	0.19962	2	4	9
-CFLE	0.19962	4	8	7
-CFVD	0.19962	2	4	13
-CFVE	0.19962	1	2	9
-CFWD	0.19962	1	2	5

-CGED	0.19962	3	6	11
-CGRD	0.19962	2	4	23
-CHFE	0.19962	1	2	2
-CHHE	0.19962	1	2	8
-CHLD	0.19962	4	8	26
-CHQD	0.19962	1	2	6
-CHWD	0.19962	1	2	8
-CHWE	0.19962	1	2	5
-CHYE	0.19962	2	4	16
-CIAD	0.19962	1	2	11
-CIFD	0.19962	1	2	9
-CIGD	0.19962	2	4	11
-CIHE	0.19962	2	4	10
-CILD	0.19962	2	4	26
-CIME	0.19962	1	2	4
-CIND	0.19962	1	2	9
-CIPE	0.19962	1	2	14
-CIWD	0.19962	1	2	8
-CKGD	0.19962	1	2	7
-CKHE	0.19962	1	2	6
-CKTD	0.19962	2	4	19
-CLCD	0.19962	1	2	6
-CLDE	0.19962	3	6	13
-CLVD	0.19962	1	2	20
-CLWE	0.19962	1	2	8
-CMCD	0.19962	1	2	12
-CMKD	0.19962	1	2	8
-CMND	0.19962	1	2	6
-CMQD	0.19962	1	2	2
-CMQE	0.19962	2	4	6
-CMWD	0.19962	1	2	8
-CNGD	0.19962	1	2	6
-CNQE	0.19962	1	2	2
-CNVE	0.19962	2	4	21
-CNWD	0.19962	1	2	4
-CNWE	0.19962	1	2	7
-CPPD	0.19962	2	4	8
-CPPE	0.19962	2	4	12
-CQCE	0.19962	1	2	7
-CQED	0.19962	1	2	7
-CQFD	0.19962	3	6	3
-CQFE	0.19962	1	2	7
-CQQD	0.19962	1	2	12
-CRID	0.19962	1	2	18
-CRIE	0.19962	2	4	22
-CRVD	0.19962	3	6	21
-CRWE	0.19962	2	4	13
-CTHE	0.19962	2	4	21
-CTWE	0.19962	1	2	13
-CTYE	0.19962	1	2	17
-CVED	0.19962	2	4	24
-CVFD	0.19962	1	2	12
-CVHD	0.19962	1	2	10
-CVME	0.19962	2	4	18
-CVWD	0.19962	1	2	17
-CVWE	0.19962	1	2	4
-CWAE	0.19962	1	2	7

-CWDE	0.19962	1	2	1
-CWME	0.19962	1	2	14
-CWRD	0.19962	1	2	12
-CWRE	0.19962	1	2	4
-CYDD	0.19962	1	2	5
-CYFD	0.19962	1	2	6
-CYYD	0.19962	1	2	6
-CYYE	0.19962	1	2	17
-CVVD	0.19962	5	10	14
-CDRD	0.21777	6	11	19
-CHVD	0.22814	4	7	16
-CILE	0.22814	4	7	20
-CPSD	0.23114	11	19	27
-CCEE	0.23955	3	5	2
-CFTE	0.23955	3	5	19
-CPCD	0.23955	3	5	6
-CQAE	0.24953	5	8	17
-CCCD	0.26616	2	3	10
-CCLE	0.26616	2	3	22
-CCWD	0.26616	2	3	2
-CDLE	0.26616	2	3	16
-CDND	0.26616	2	3	15
-CGNE	0.26616	2	3	8
-CKAE	0.26616	2	3	11
-CKFD	0.26616	2	3	18
-CKGE	0.26616	2	3	5
-CKWD	0.26616	2	3	5
-CMAE	0.26616	2	3	13
-CMKE	0.26616	2	3	7
-CPDD	0.26616	2	3	10
-CQND	0.26616	2	3	6
-CRWD	0.26616	2	3	10
-CTCE	0.26616	2	3	21
-CTQD	0.26616	2	3	10
-CTWD	0.26616	2	3	5
-CWED	0.26616	2	3	7
-CWYD	0.26616	2	3	25
-CWYE	0.26616	2	3	12
-CYDE	0.26616	2	3	8
-CYMD	0.26616	2	3	14
-CTAD	0.26616	6	9	24
-CTYD	0.26616	6	9	22
-CTMD	0.28518	5	7	18
-CSYD	0.29036	8	11	21
-CAID	0.29943	3	4	16
-CDGD	0.29943	3	4	15
-CGTD	0.29943	3	4	7
-CPME	0.29943	3	4	15
-CPND	0.29943	3	4	9
-CSED	0.29943	6	8	31
-CRDD	0.31052	7	9	22
-CDWD	0.3194	4	5	9
-CEGE	0.3194	4	5	3
-CYVD	0.3194	4	5	19
-CDSD	0.32666	9	11	23
-CIVE	0.3327	5	6	17
-CNED	0.3327	5	6	13

-CCIE	0.34221	6	7	14
-CYSD	0.34221	6	7	18
-CAED	0.39925	1	1	8
-CAPD	0.39925	2	2	10
-CCCE	0.39925	1	1	7
-CCFD	0.39925	1	1	1
-CCQE	0.39925	1	1	3
-CCSD	0.39925	4	4	13
-CCVE	0.39925	1	1	7
-CDFE	0.39925	1	1	2
-CDQE	0.39925	1	1	2
-CEDE	0.39925	1	1	1
-CEID	0.39925	1	1	5
-CEME	0.39925	1	1	6
-CEPE	0.39925	1	1	12
-CEQD	0.39925	1	1	6
-CEQE	0.39925	2	2	9
-CFFD	0.39925	1	1	5
-CFHD	0.39925	1	1	11
-CFIE	0.39925	1	1	11
-CFKE	0.39925	1	1	4
-CFLD	0.39925	1	1	12
-CFME	0.39925	2	2	14
-CFQD	0.39925	1	1	6
-CFWE	0.39925	1	1	4
-CFYD	0.39925	1	1	6
-CGFE	0.39925	1	1	7
-CGHE	0.39925	1	1	8
-CGQD	0.39925	1	1	8
-CHDD	0.39925	2	2	6
-CHFD	0.39925	1	1	7
-CHKD	0.39925	1	1	2
-CHME	0.39925	1	1	10
-CHNE	0.39925	2	2	15
-CHPD	0.39925	2	2	7
-CHQE	0.39925	2	2	17
-CHRD	0.39925	8	8	10
-CHYD	0.39925	1	1	16
-CICD	0.39925	1	1	1
-CICE	0.39925	1	1	4
-CIED	0.39925	1	1	3
-CIFE	0.39925	1	1	3
-CINE	0.39925	2	2	8
-CIRD	0.39925	2	2	8
-CIWE	0.39925	1	1	6
-CKCE	0.39925	1	1	5
-CKDD	0.39925	1	1	7
-CKFE	0.39925	2	2	7
-CKHD	0.39925	1	1	2
-CKVD	0.39925	2	2	7
-CKWE	0.39925	1	1	8
-CLIE	0.39925	2	2	5
-CLWD	0.39925	2	2	19
-CMDE	0.39925	1	1	1
-CMFD	0.39925	1	1	3
-CMGD	0.39925	2	2	8
-CMME	0.39925	1	1	2

-CNCD	0.39925	1	1	4
-CNFD	0.39925	1	1	2
-CNKE	0.39925	1	1	7
-CNNE	0.39925	1	1	8
-CNRE	0.39925	1	1	23
-CPQD	0.39925	1	1	4
-CPWD	0.39925	2	2	11
-CQCD	0.39925	1	1	2
-CQHE	0.39925	1	1	2
-CQPD	0.39925	1	1	2
-CQPE	0.39925	1	1	4
-CQQE	0.39925	1	1	1
-CQWD	0.39925	2	2	3
-CQWE	0.39925	1	1	5
-CQYE	0.39925	1	1	3
-CWAD	0.39925	2	2	6
-CWDD	0.39925	1	1	5
-CWEE	0.39925	1	1	1
-CWFD	0.39925	1	1	12
-CWFE	0.39925	1	1	6
-CWHD	0.39925	1	1	4
-CWHE	0.39925	1	1	4
-CWIE	0.39925	1	1	9
-CWMD	0.39925	1	1	5
-CWND	0.39925	1	1	7
-CWVE	0.39925	1	1	7
-CWWD	0.39925	1	1	2
-CWWE	0.39925	1	1	3
-CYCE	0.39925	1	1	9
-CYHD	0.39925	2	2	6
-CYID	0.39925	1	1	10
-CYNE	0.39925	1	1	4
-CYQD	0.39925	1	1	7
-CYQE	0.39925	2	2	9
-CYWD	0.39925	1	1	2
-CDPD	0.46579	7	6	18
-CVVE	0.50813	28	22	31
-CMHE	0.53233	4	3	9
-CPAE	0.53233	4	3	24
-CCHD	0.59887	3	2	8
-CEDD	0.59887	3	2	3
-CFRE	0.59887	3	2	17
-CIVD	0.59887	12	8	21
-CKID	0.59887	3	2	6
-CNCE	0.59887	6	4	23
-CSQD	0.59887	3	2	10
-CYND	0.59887	3	2	10
-CTID	0.62739	11	7	10
-CPGE	0.66541	5	3	20
-CCRD	0.79849	4	2	24
-CEMD	0.79849	2	1	6
-CHAD	0.79849	2	1	18
-CIMD	0.79849	2	1	3
-CKPD	0.79849	2	1	6
-CMNE	0.79849	2	1	7
-CNQD	0.79849	2	1	5
-CQLE	0.79849	2	1	9

-CRHE	0.79849	2	1	14
-CVMD	0.79849	2	1	11
-CMID	1.1977	3	1	13
-CIIE	1.4639	11	3	5
-CTIE	2.8945	58	8	16
-CIID	5.0904	51	4	18
-CVIE	5.6054	351	25	41
-CVID	15.0116	376	10	18

Note:β-branched in A1 and Ile in A2 are highlighted in yellow

3. Enrichment Factors of Sequencing Motives Ending with –CXXR/K

		Counts		
Motif	Enrichment	37 °C	25 °C	Naive
-CEVR	0.014259	1	28	41
-CLNR	0.014787	1	27	76
-CERR	0.015356	1	26	52
-CDSK	0.01597	1	25	29
-CRSK	0.01597	1	25	32
-CVPR	0.01597	1	25	37
-CERK	0.017359	1	23	19
-CLGK	0.017359	1	23	33
-CNTR	0.017359	1	23	39
-CVDR	0.017359	1	23	31
-CASR	0.018148	1	22	64
-CGSK	0.018148	1	22	40
-CVNR	0.018148	1	22	49
-CTAR	0.019475	2	41	74
-CPER	0.019962	1	20	33
-CLDK	0.021013	1	19	23
-CSNR	0.021581	2	37	58
-CPLR	0.02218	1	18	77
-CSTK	0.02218	1	18	35
-CSYR	0.02218	1	18	64
-CLTR	0.022814	2	35	115
-CGPR	0.023485	1	17	51
-CNSR	0.023485	2	34	52
-CPLK	0.023485	2	34	36
-CPSR	0.023485	2	34	82
-CQRK	0.024953	1	16	22
-CRNR	0.024953	1	16	42
-CISK	0.026616	1	15	28
-CKNR	0.026616	1	15	19
-CRPR	0.026616	1	15	58
-CSAK	0.026616	1	15	31
-CTYR	0.026616	1	15	49
-CYIK	0.026616	1	15	11
-CGKR	0.028518	1	14	53
-CIGR	0.028518	1	14	35
-CIVK	0.028518	1	14	19
-CNMK	0.028518	1	14	15
-CVSR	0.028518	2	28	54
-CANR	0.030711	1	13	43
-CHLK	0.030711	1	13	32
-CKAR	0.030711	1	13	28
-CKGR	0.030711	1	13	19
-CKRK	0.030711	1	13	27
-CLTK	0.030711	2	26	25
-CPTR	0.030711	2	26	41
-CQDR	0.030711	1	13	16
-CQMK	0.030711	1	13	10
-CRLK	0.030711	1	13	39
-CSHK	0.030711	1	13	16
-CSKR	0.030711	1	13	54

-CTDK	0.030711	1	13	16
-CVER	0.030711	1	13	35
-CVVK	0.030711	1	13	28
-CSSR	0.031313	4	51	105
-CLSK	0.03194	2	25	57
-CCKK	0.03327	1	12	18
-CDFR	0.03327	1	12	39
-CELK	0.03327	1	12	18
-CETK	0.03327	1	12	13
-CFGR	0.03327	1	12	32
-CKLK	0.03327	1	12	40
-CKRR	0.03327	1	12	44
-CKVR	0.03327	2	24	34
-CQLK	0.03327	1	12	13
-CSHR	0.03327	1	12	36
-CTYK	0.03327	1	12	25
-CPRR	0.034717	2	23	66
-CSRK	0.034717	2	23	51
-CTPR	0.034717	2	23	45
-CDGR	0.036295	1	11	29
-CEFK	0.036295	1	11	15
-CEGR	0.036295	1	11	23
-CESK	0.036295	2	22	34
-CGDK	0.036295	1	11	21
-CIRK	0.036295	1	11	33
-CKPR	0.036295	1	11	26
-CKQK	0.036295	1	11	16
-CLKR	0.036295	2	22	50
-CMPK	0.036295	1	11	26
-CTER	0.036295	1	11	34
-CTGK	0.036295	1	11	29
-CTKK	0.036295	1	11	32
-CTRK	0.036295	1	11	39
-CVEK	0.036295	1	11	19
-CYSR	0.036295	1	11	30
-CLNK	0.038023	2	21	25
-CPQK	0.038023	2	21	15
-CLLK	0.038637	3	31	73
-CEER	0.039925	1	10	12
-CENR	0.039925	1	10	21
-CFTK	0.039925	1	10	12
-CIPR	0.039925	1	10	24
-CLAK	0.039925	2	20	34
-CLAR	0.039925	3	30	59
-CLKK	0.039925	1	10	19
-CNLK	0.039925	2	20	40
-CQTR	0.039925	1	10	40
-CRAK	0.039925	2	20	46
-CRKR	0.039925	2	20	44
-CSVK	0.039925	2	20	42
-CWTK	0.039925	1	10	9
-CWTR	0.039925	1	10	32
-CYWR	0.039925	1	10	28
-CTSK	0.041301	3	29	40
-CISR	0.042026	2	19	33
-CPPK	0.042026	2	19	18
-CSLK	0.042026	2	19	64

-CVPK	0.042026	2	19	21
-CAIR	0.044361	1	9	38
-CDYR	0.044361	1	9	30
-CEDR	0.044361	1	9	8
-CEKR	0.044361	1	9	21
-CFPR	0.044361	1	9	26
-CIHR	0.044361	1	9	18
-CIYK	0.044361	1	9	31
-CKMK	0.044361	1	9	11
-CLVK	0.044361	1	9	18
-CNIK	0.044361	1	9	8
-CRQR	0.044361	1	9	47
-CSAR	0.044361	2	18	79
-CTPK	0.044361	1	9	23
-CTVK	0.044361	2	18	24
-CYVK	0.044361	1	9	38
-CRSR	0.044361	3	27	77
-CDLR	0.045369	5	44	54
-CGSR	0.04697	4	34	50
-CITR	0.04697	2	17	74
-CPVK	0.04697	2	17	26
-CTTK	0.04697	2	17	14
-CVKR	0.04697	2	17	40
-CRDR	0.047909	3	25	43
-CVLR	0.048688	5	41	83
-CDGK	0.049906	1	8	19
-CESR	0.049906	4	32	39
-CFFR	0.049906	1	8	11
-CGGR	0.049906	1	8	33
-CHLR	0.049906	1	8	75
-CHMR	0.049906	1	8	12
-CIAR	0.049906	2	16	33
-CIEK	0.049906	2	16	26
-CKCK	0.049906	1	8	19
-CKSR	0.049906	2	16	62
-CMAR	0.049906	1	8	22
-CMLR	0.049906	1	8	60
-CMNK	0.049906	1	8	11
-CMSR	0.049906	1	8	31
-CNFR	0.049906	1	8	20
-CNHK	0.049906	1	8	20
-CPEK	0.049906	1	8	8
-CPFK	0.049906	1	8	17
-CPRK	0.049906	2	16	34
-CPTK	0.049906	1	8	22
-CPVR	0.049906	3	24	78
-CRVK	0.049906	2	16	33
-CSPR	0.049906	2	16	73
-CSQR	0.049906	1	8	27
-CTMK	0.049906	1	8	16
-CYAR	0.049906	1	8	30
-CYTR	0.049906	2	16	52
-CSSK	0.052076	3	23	64
-CARK	0.053233	2	15	50
-CKLR	0.053233	2	15	71
-CPKR	0.053233	2	15	30
-CRER	0.053233	2	15	46

-CTFK	0.053233	2	15	21
-CTHK	0.053233	2	15	18
-CTKR	0.053233	2	15	41
-CAKK	0.054443	3	22	36
-CLRR	0.054443	3	22	88
-CAAR	0.057035	1	7	25
-CAVK	0.057035	1	7	35
-CCSK	0.057035	1	7	19
-CDQR	0.057035	1	7	19
-CETR	0.057035	1	7	16
-CFRR	0.057035	1	7	44
-CFYR	0.057035	1	7	23
-CGER	0.057035	1	7	19
-CHPR	0.057035	1	7	21
-CHVK	0.057035	1	7	11
-CIPK	0.057035	1	7	22
-CIYR	0.057035	1	7	27
-CKIR	0.057035	1	7	23
-CKKR	0.057035	1	7	12
-CLFK	0.057035	1	7	12
-CMPR	0.057035	1	7	32
-CNEK	0.057035	1	7	21
-CPIR	0.057035	2	14	36
-CPKK	0.057035	1	7	18
-CQYR	0.057035	1	7	28
-CSFK	0.057035	1	7	21
-CSIK	0.057035	1	7	29
-CTNK	0.057035	1	7	18
-CVNK	0.057035	1	7	19
-CWSR	0.057035	1	7	28
-CYCR	0.057035	1	7	28
-CRTR	0.057035	3	21	111
-CVSK	0.057035	3	21	45
-CNKR	0.059887	3	20	32
-CSRR	0.059887	3	20	96
-CVGK	0.059887	3	20	18
-CLPR	0.060755	7	46	72
-CDDR	0.061422	2	13	31
-CEMR	0.061422	2	13	17
-CTNR	0.061422	4	26	54
-CVRK	0.061422	2	13	36
-CVTR	0.061422	2	13	30
-CNDR	0.063039	3	19	25
-CVAR	0.063039	3	19	59
-CAGR	0.066541	1	6	33
-CAKR	0.066541	2	12	30
-CATR	0.066541	2	12	43
-CAYR	0.066541	1	6	29
-CCLK	0.066541	1	6	6
-CCNK	0.066541	1	6	13
-CCPK	0.066541	1	6	8
-CCPR	0.066541	1	6	22
-CDCR	0.066541	1	6	24
-CDMR	0.066541	2	12	21
-CDRK	0.066541	2	12	30
-CEDK	0.066541	1	6	12
-CEHR	0.066541	1	6	19

-CEPR	0.066541	2	12	30
-CFAK	0.066541	1	6	14
-CFDR	0.066541	2	12	13
-CFLR	0.066541	2	12	41
-CFRK	0.066541	1	6	12
-CGAR	0.066541	1	6	43
-CGFR	0.066541	1	6	24
-CGMK	0.066541	1	6	17
-CGTR	0.066541	1	6	52
-CIIK	0.066541	1	6	9
-CILK	0.066541	1	6	23
-CIMK	0.066541	1	6	25
-CIWK	0.066541	1	6	4
-CKFK	0.066541	1	6	16
-CKYK	0.066541	1	6	6
-CLYK	0.066541	2	12	42
-CMDK	0.066541	1	6	10
-CMGK	0.066541	1	6	20
-CMKR	0.066541	1	6	13
-CMLK	0.066541	1	6	22
-CNGK	0.066541	1	6	10
-CNNK	0.066541	1	6	10
-CNQR	0.066541	1	6	14
-CNWK	0.066541	1	6	16
-CPAR	0.066541	2	12	39
-CPMR	0.066541	1	6	37
-CQAR	0.066541	1	6	14
-CQGR	0.066541	1	6	18
-CQHR	0.066541	1	6	21
-CQKR	0.066541	2	12	16
-CQLR	0.066541	2	12	30
-CRKK	0.066541	1	6	17
-CRLR	0.066541	4	24	142
-CSCK	0.066541	1	6	20
-CSKK	0.066541	1	6	29
-CSNK	0.066541	2	12	18
-CVCK	0.066541	1	6	13
-CVKK	0.066541	2	12	33
-CVLK	0.066541	4	24	33
-CVQK	0.066541	1	6	34
-CVYK	0.066541	1	6	15
-CWRR	0.066541	1	6	26
-CYER	0.066541	1	6	19
-CYLK	0.066541	1	6	48
-CDIK	0.066541	3	18	21
-CKTR	0.066541	3	18	47
-CLER	0.066541	3	18	39
-CLSR	0.066541	5	30	95
-CSTR	0.066541	6	36	96
-CSVR	0.066541	6	36	68
-CTVR	0.068164	7	41	82
-CYVR	0.069434	4	23	58
-CGLR	0.070455	3	17	72
-CKDR	0.070455	3	17	26
-CTDR	0.070455	3	17	34
-CTRR	0.070455	3	17	92
-CDAR	0.07259	2	11	37

-CELR	0.07259	6	33	63
-CGFK	0.07259	2	11	12
-CLCR	0.07259	2	11	43
-CPHR	0.07259	2	11	42
-CPYR	0.07259	2	11	34
-CQFR	0.07259	2	11	23
-CTAK	0.07259	2	11	18
-CMVR	0.074859	3	16	31
-CQVR	0.074859	3	16	32
-CSYK	0.074859	3	16	34
-CSIR	0.075533	7	37	73
-CRRR	0.076047	4	21	94
-CSQK	0.076047	4	21	39
-CAEK	0.079849	1	5	1
-CAQR	0.079849	1	5	22
-CARR	0.079849	2	10	65
-CASK	0.079849	2	10	49
-CAVR	0.079849	4	20	53
-CAWR	0.079849	1	5	24
-CAYK	0.079849	1	5	19
-CCAK	0.079849	2	10	12
-CCEK	0.079849	1	5	19
-CCER	0.079849	2	10	28
-CCQK	0.079849	1	5	22
-CCSR	0.079849	2	10	28
-CCYR	0.079849	1	5	13
-CDAK	0.079849	1	5	11
-CDDK	0.079849	1	5	9
-CDER	0.079849	3	15	20
-CDHK	0.079849	1	5	9
-CFKK	0.079849	1	5	2
-CFSK	0.079849	2	10	22
-CFVK	0.079849	1	5	6
-CGMR	0.079849	2	10	42
-CGNR	0.079849	1	5	17
-CHDK	0.079849	1	5	14
-CIER	0.079849	1	5	26
-CKHR	0.079849	1	5	6
-CKYR	0.079849	1	5	10
-CLEK	0.079849	1	5	15
-CMAK	0.079849	1	5	16
-CMRK	0.079849	1	5	20
-CMVK	0.079849	1	5	10
-CNDK	0.079849	2	10	13
-CNGR	0.079849	10	50	72
-CNRK	0.079849	1	5	19
-CNTK	0.079849	2	10	7
-CPAK	0.079849	1	5	28
-CPDK	0.079849	1	5	9
-CPDR	0.079849	2	10	26
-CPFR	0.079849	2	10	38
-CPSK	0.079849	3	15	39
-CQNR	0.079849	1	5	34
-CRMK	0.079849	1	5	20
-CSDR	0.079849	3	15	47
-CSEK	0.079849	1	5	16
-CSFR	0.079849	2	10	22

-CTEK	0.079849	1	5	10
-CWNR	0.079849	1	5	21
-CWSK	0.079849	1	5	19
-CYCK	0.079849	1	5	13
-CYDR	0.079849	5	25	25
-CYNR	0.079849	1	5	29
-CYQK	0.079849	1	5	13
-CGVR	0.085553	3	14	22
-CLQK	0.085553	3	14	20
-CNPR	0.085553	3	14	27
-CRTK	0.085553	3	14	47
-CTLK	0.085553	3	14	47
-CAFR	0.088721	2	9	41
-CDLK	0.088721	2	9	22
-CFLK	0.088721	2	9	24
-CHSR	0.088721	2	9	36
-CMDR	0.088721	2	9	15
-CNAR	0.088721	2	9	36
-CPPR	0.088721	2	9	51
-CSLR	0.08983	9	40	127
-CFSR	0.090152	7	31	69
-CEAK	0.092134	3	13	22
-CLYR	0.092134	3	13	60
-CNVR	0.092134	6	26	62
-CSGR	0.092134	6	26	77
-CTFR	0.092134	3	13	47
-CTHR	0.092134	3	13	38
-CSER	0.093157	7	30	50
-CMTR	0.09394	4	17	42
-CLLR	0.094558	9	38	135
-CGYR	0.095058	5	21	42
-CTLR	0.097114	9	37	98
-CACK	0.099811	1	4	15
-CADR	0.099811	1	4	31
-CAIK	0.099811	1	4	20
-CAMR	0.099811	3	12	39
-CANK	0.099811	1	4	24
-CAQK	0.099811	2	8	18
-CAWK	0.099811	1	4	7
-CCAR	0.099811	3	12	31
-CCDK	0.099811	1	4	10
-CCKR	0.099811	1	4	16
-CCYK	0.099811	1	4	6
-CDFK	0.099811	1	4	6
-CDIR	0.099811	3	12	39
-CDKR	0.099811	1	4	19
-CDNR	0.099811	1	4	27
-CDPK	0.099811	2	8	9
-CDQK	0.099811	1	4	5
-CDSR	0.099811	3	12	43
-CDTR	0.099811	2	8	42
-CDVR	0.099811	6	24	61
-CECR	0.099811	1	4	13
-CENK	0.099811	1	4	7
-CEVK	0.099811	1	4	4
-CFDK	0.099811	1	4	4
-CFKR	0.099811	1	4	15

-CFPK	0.099811	1	4	10
-CFTR	0.099811	1	4	26
-CGKK	0.099811	1	4	11
-CGQK	0.099811	1	4	10
-CHCK	0.099811	1	4	8
-CHCR	0.099811	1	4	26
-CHIK	0.099811	1	4	11
-CHKR	0.099811	1	4	18
-CHNR	0.099811	1	4	18
-CHRK	0.099811	1	4	23
-CHRR	0.099811	2	8	35
-CHSK	0.099811	2	8	13
-CILR	0.099811	4	16	59
-CIQK	0.099811	1	4	6
-CITK	0.099811	3	12	24
-CKFR	0.099811	1	4	29
-CKPK	0.099811	1	4	11
-CLCK	0.099811	1	4	16
-CLDR	0.099811	4	16	50
-CMER	0.099811	2	8	12
-CMHK	0.099811	1	4	8
-CMKK	0.099811	1	4	6
-CMMK	0.099811	1	4	11
-CMMR	0.099811	2	8	20
-CMRR	0.099811	1	4	31
-CMTK	0.099811	2	8	26
-CNIR	0.099811	2	8	30
-CNPK	0.099811	1	4	6
-CNVK	0.099811	2	8	15
-CPCK	0.099811	1	4	20
-CPNR	0.099811	2	8	30
-CQER	0.099811	2	8	20
-CQIR	0.099811	1	4	17
-CREK	0.099811	1	4	8
-CRPK	0.099811	1	4	24
-CRRK	0.099811	3	12	50
-CSGK	0.099811	2	8	21
-CTTR	0.099811	6	24	56
-CVFK	0.099811	1	4	27
-CVWK	0.099811	1	4	20
-CVWR	0.099811	1	4	35
-CWAR	0.099811	1	4	11
-CWLK	0.099811	1	4	12
-CWPR	0.099811	1	4	22
-CWWK	0.099811	1	4	14
-CYAK	0.099811	2	8	25
-CYGK	0.099811	1	4	5
-CYHK	0.099811	1	4	11
-CYKR	0.099811	1	4	22
-CYNK	0.099811	1	4	16
-CYTK	0.099811	2	8	12
-CQSR	0.099811	5	20	50
-CTSR	0.10303	8	31	107
-CNLR	0.10647	4	15	72
-CSDK	0.10647	4	15	28
-CYPR	0.10647	4	15	29
-CATK	0.10889	3	11	38

-CMSK	0.10889	3	11	36
-CVTK	0.10889	3	11	27
-CAPR	0.11407	2	7	40
-CCVR	0.11407	4	14	50
-CDHR	0.11407	4	14	38
-CHTR	0.11407	2	7	21
-CIFR	0.11407	2	7	23
-CIRR	0.11407	4	14	65
-CMHR	0.11407	2	7	30
-CPIK	0.11407	2	7	18
-CQGK	0.11407	2	7	17
-CRGK	0.11407	2	7	32
-CRGR	0.11407	2	7	38
-CTGR	0.11407	2	7	40
-CWCK	0.11407	2	7	9
-CWDK	0.11407	2	7	8
-CYQR	0.11407	2	7	24
-CDPR	0.11743	5	17	21
-CFVR	0.11977	3	10	26
-CNMR	0.11977	3	10	31
-CYRK	0.11977	3	10	38
-CLGR	0.12284	8	26	61
-CRYR	0.12284	4	13	56
-CKSK	0.12608	6	19	23
-CRVR	0.12703	7	22	80
-CAFK	0.13308	1	3	15
-CAHK	0.13308	1	3	7
-CAPK	0.13308	1	3	14
-CCGK	0.13308	2	6	30
-CCMK	0.13308	1	3	12
-CCTK	0.13308	1	3	27
-CCTR	0.13308	1	3	26
-CCWR	0.13308	1	3	13
-CDMK	0.13308	1	3	12
-CDNK	0.13308	2	6	17
-CDYK	0.13308	2	6	11
-CEHK	0.13308	1	3	11
-CFFK	0.13308	1	3	12
-CFMK	0.13308	1	3	10
-CFWR	0.13308	1	3	15
-CGEK	0.13308	1	3	19
-CGGK	0.13308	2	6	14
-CGIR	0.13308	2	6	33
-CGPK	0.13308	1	3	11
-CGRK	0.13308	2	6	21
-CGWK	0.13308	1	3	13
-CHIR	0.13308	1	3	21
-CHYK	0.13308	1	3	12
-CIAK	0.13308	2	6	18
-CIDK	0.13308	1	3	5
-CIDR	0.13308	4	12	16
-CIHK	0.13308	1	3	8
-CIKR	0.13308	1	3	21
-CKAK	0.13308	2	6	16
-CKHK	0.13308	1	3	11
-CKIK	0.13308	1	3	18
-CKVK	0.13308	1	3	11

-CLHR	0.13308	2	6	47
-CLMK	0.13308	1	3	19
-CLWK	0.13308	1	3	15
-CMFR	0.13308	1	3	11
-CMQR	0.13308	1	3	10
-CNCK	0.13308	1	3	13
-CNHR	0.13308	2	6	40
-CNYK	0.13308	2	6	20
-CPMK	0.13308	1	3	6
-CQDK	0.13308	1	3	10
-CRDK	0.13308	1	3	16
-CRWK	0.13308	1	3	22
-CTQK	0.13308	1	3	10
-CVFR	0.13308	2	6	16
-CVHK	0.13308	2	6	26
-CVHR	0.13308	1	3	50
-CVIK	0.13308	1	3	23
-CWAK	0.13308	1	3	7
-CWDR	0.13308	2	6	10
-CWER	0.13308	1	3	12
-CWLR	0.13308	1	3	46
-CWQR	0.13308	1	3	16
-CWRK	0.13308	1	3	15
-CWVK	0.13308	1	3	11
-CWWR	0.13308	1	3	14
-CYEK	0.13308	1	3	6
-CYMK	0.13308	1	3	16
-CGCR	0.13308	3	9	34
-CKTK	0.13308	3	9	15
-CLFR	0.13308	3	9	43
-CTWR	0.13308	3	9	45
-CYFR	0.13308	3	9	25
-CHDR	0.13974	7	20	26
-CRAR	0.14091	6	17	91
-CLVR	0.14259	10	28	66
-CFER	0.14518	4	11	15
-CLPK	0.14518	4	11	33
-CALR	0.14787	10	27	106
-CCIR	0.14972	3	8	27
-CNNR	0.14972	3	8	23
-CRCR	0.14972	3	8	39
-CSPK	0.14972	6	16	29
-CEAR	0.15356	5	13	36
-CADK	0.1597	2	5	23
-CCFR	0.1597	2	5	22
-CDKK	0.1597	2	5	15
-CEFR	0.1597	4	10	27
-CEGK	0.1597	2	5	18
-CFGK	0.1597	2	5	8
-CGDR	0.1597	2	5	41
-CGQR	0.1597	2	5	25
-CIWR	0.1597	2	5	21
-CLQR	0.1597	2	5	25
-CNKK	0.1597	2	5	21
-CNRR	0.1597	2	5	45
-CPGR	0.1597	2	5	19
-CPNK	0.1597	2	5	16

-CVIR	0.1597	8	20	46
-CVMK	0.1597	2	5	14
-CWCR	0.1597	2	5	14
-CYMR	0.1597	2	5	25
-CLIR	0.1644	7	17	47
-CTIR	0.16635	5	12	43
-CFEK	0.17111	3	7	3
-CHGR	0.17111	3	7	19
-CLMR	0.17111	3	7	57
-CNER	0.17111	3	7	19
-CRFR	0.17744	4	9	50
-CQRR	0.18148	5	11	41
-CVGR	0.18427	6	13	62
-CALK	0.18912	9	19	61
-CVVR	0.19012	10	21	33
-CSMR	0.19962	9	18	83
-CAER	0.19962	2	4	30
-CAGK	0.19962	2	4	13
-CAHR	0.19962	2	4	31
-CAMK	0.19962	1	2	14
-CCHK	0.19962	2	4	15
-CCRR	0.19962	2	4	40
-CDCK	0.19962	1	2	2
-CDEK	0.19962	2	4	6
-CECK	0.19962	1	2	8
-CEEK	0.19962	1	2	4
-CEIK	0.19962	2	4	7
-CEIR	0.19962	3	6	19
-CEQK	0.19962	1	2	3
-CEWK	0.19962	1	2	5
-CEWR	0.19962	1	2	28
-CFHR	0.19962	1	2	12
-CFIR	0.19962	1	2	18
-CFQK	0.19962	2	4	14
-CFQR	0.19962	2	4	28
-CFYK	0.19962	1	2	7
-CGCK	0.19962	1	2	17
-CGHR	0.19962	2	4	26
-CGIK	0.19962	1	2	14
-CGLK	0.19962	1	2	23
-CGNK	0.19962	1	2	9
-CGYK	0.19962	1	2	14
-CHAK	0.19962	1	2	8
-CHAR	0.19962	2	4	24
-CHFR	0.19962	1	2	13
-CHKK	0.19962	2	4	15
-CHPK	0.19962	1	2	17
-CHQK	0.19962	1	2	8
-CHQR	0.19962	3	6	28
-CHYR	0.19962	1	2	20
-CIFK	0.19962	1	2	8
-CIGK	0.19962	1	2	9
-CINK	0.19962	2	4	15
-CINR	0.19962	1	2	25
-CKEK	0.19962	1	2	4
-CKGK	0.19962	1	2	7
-CMEK	0.19962	1	2	7

-CMGR	0.19962	1	2	18
-CMQK	0.19962	1	2	7
-CMWK	0.19962	1	2	11
-CNQK	0.19962	1	2	8
-CNWR	0.19962	2	4	28
-CNYR	0.19962	2	4	28
-CPCR	0.19962	2	4	56
-CPGK	0.19962	2	4	8
-CPWK	0.19962	1	2	18
-CPWR	0.19962	1	2	35
-CQCR	0.19962	3	6	13
-CQEK	0.19962	1	2	11
-CQFK	0.19962	1	2	11
-CQIK	0.19962	1	2	11
-CQKK	0.19962	1	2	4
-CQMR	0.19962	2	4	16
-CQVK	0.19962	4	8	12
-CQYK	0.19962	1	2	2
-CRHR	0.19962	3	6	50
-CRIK	0.19962	1	2	11
-CRIR	0.19962	6	12	58
-CRQK	0.19962	2	4	12
-CRWR	0.19962	2	4	34
-CSMK	0.19962	3	6	27
-CTWK	0.19962	2	4	15
-CVCR	0.19962	1	2	28
-CWFR	0.19962	1	2	11
-CWGR	0.19962	2	4	8
-CWMK	0.19962	1	2	17
-CWNK	0.19962	1	2	11
-CWPK	0.19962	1	2	6
-CWYK	0.19962	1	2	10
-CYGR	0.19962	2	4	33
-CYYK	0.19962	1	2	13
-CIVR	0.20913	11	21	46
-CPQR	0.21293	8	15	25
-CTCR	0.21498	7	13	50
-CRMR	0.2218	5	9	79
-CNSK	0.22358	14	25	34
-CVMR	0.22458	9	16	38
-CACR	0.23955	6	10	42
-CGTK	0.23955	3	5	14
-CMCR	0.23955	3	5	20
-CQAK	0.23955	3	5	13
-CYRR	0.23955	3	5	48
-CLRK	0.25407	7	11	56
-CCGR	0.26616	2	3	28
-CCHR	0.26616	2	3	19
-CFNR	0.26616	2	3	28
-CGWR	0.26616	2	3	19
-CICK	0.26616	2	3	7
-CKER	0.26616	2	3	5
-CKKK	0.26616	2	3	7
-CKMR	0.26616	2	3	33
-CNFK	0.26616	2	3	16
-CRYK	0.26616	2	3	17
-CSWK	0.26616	2	3	26

-CTQR	0.26616	4	6	31
-CVAK	0.26616	8	12	27
-CVYR	0.26616	2	3	21
-CWMR	0.26616	2	3	18
-CWQK	0.26616	2	3	17
-CWYR	0.26616	2	3	12
-CYPK	0.26616	2	3	20
-CDRR	0.27149	17	25	61
-CYLR	0.27947	7	10	35
-CCRK	0.29943	3	4	21
-CHTK	0.29943	3	4	27
-CMNR	0.30711	10	13	33
-CICR	0.3194	4	5	11
-CMIR	0.3194	4	5	22
-CCLR	0.3327	5	6	66
-CHHR	0.3327	5	6	20
-CGRR	0.34934	7	8	31
-CSCR	0.36295	10	11	47
-CTMR	0.36295	10	11	50
-CAAK	0.39925	2	2	16
-CCCK	0.39925	1	1	12
-CCFK	0.39925	2	2	8
-CCIK	0.39925	1	1	8
-CCNR	0.39925	3	3	16
-CCQR	0.39925	2	2	15
-CCWK	0.39925	1	1	6
-CDTK	0.39925	3	3	14
-CDVK	0.39925	2	2	17
-CDWK	0.39925	1	1	10
-CEKK	0.39925	1	1	2
-CEMK	0.39925	1	1	2
-CEPK	0.39925	1	1	11
-CEYK	0.39925	1	1	5
-CEYR	0.39925	3	3	12
-CFAR	0.39925	2	2	17
-CFCK	0.39925	2	2	6
-CFHK	0.39925	1	1	3
-CFIK	0.39925	1	1	8
-CFMR	0.39925	2	2	21
-CFNK	0.39925	2	2	8
-CFWK	0.39925	1	1	3
-CHEK	0.39925	1	1	8
-CHHK	0.39925	1	1	4
-CHMK	0.39925	1	1	15
-CHNK	0.39925	1	1	18
-CHWK	0.39925	1	1	6
-CIIR	0.39925	4	4	15
-CIKK	0.39925	2	2	10
-CIQR	0.39925	1	1	14
-CKDK	0.39925	1	1	3
-CKNK	0.39925	1	1	12
-CKQR	0.39925	1	1	20
-CKWK	0.39925	1	1	12
-CKWR	0.39925	1	1	8
-CLHK	0.39925	2	2	16
-CLWR	0.39925	2	2	31
-CMCK	0.39925	1	1	2

-CMFK	0.39925	1	1	10
-CMYR	0.39925	2	2	11
-CNAK	0.39925	2	2	16
-CNCR	0.39925	2	2	10
-CQCK	0.39925	1	1	4
-CQHK	0.39925	1	1	6
-CQNK	0.39925	1	1	22
-CQPK	0.39925	1	1	4
-CQPR	0.39925	1	1	21
-CQQK	0.39925	1	1	6
-CQSK	0.39925	1	1	8
-CQWK	0.39925	1	1	7
-CQWR	0.39925	1	1	8
-CRCK	0.39925	2	2	15
-CRHK	0.39925	2	2	32
-CRNK	0.39925	1	1	19
-CSWR	0.39925	2	2	44
-CTCK	0.39925	1	1	12
-CTIK	0.39925	2	2	18
-CVDK	0.39925	1	1	2
-CWEK	0.39925	1	1	6
-CWFK	0.39925	2	2	5
-CWGK	0.39925	1	1	6
-CWHK	0.39925	1	1	8
-CWHR	0.39925	2	2	16
-CWIK	0.39925	1	1	7
-CWIR	0.39925	1	1	13
-CWKK	0.39925	1	1	4
-CWKR	0.39925	1	1	16
-CWVR	0.39925	2	2	23
-CYDK	0.39925	1	1	15
-CYFK	0.39925	1	1	14
-CYHR	0.39925	1	1	26
-CYIR	0.39925	2	2	29
-CYKK	0.39925	1	1	13
-CYSK	0.39925	1	1	18
-CYWK	0.39925	1	1	7
-CYYR	0.39925	3	3	21
-CVRR	0.45628	16	14	83
-CIMR	0.49906	10	8	19
-CCDR	0.53233	4	3	13
-CHER	0.53233	4	3	25
-CMWR	0.55894	7	5	23
-CKCR	0.59887	3	2	11
-CQQR	0.59887	6	4	27
-CVQR	0.59887	6	4	22
-CHVR	0.66541	10	6	43
-CHGK	0.69868	7	4	11
-CGAK	0.71864	9	5	17
-CCCR	0.79849	4	2	15
-CCVK	0.79849	2	1	10
-CDWR	0.79849	2	1	15
-CFCR	0.79849	2	1	8
-CGHK	0.79849	2	1	19
-CHFK	0.79849	2	1	3
-CHWR	0.79849	2	1	18
-CMIK	0.79849	2	1	8

-CPHK	0.79849	2	1	11
-CRFK	0.79849	2	1	18
-CLIK	0.8484	17	8	23
-CQTK	1.5471	31	8	29
-CGVK	1.6968	68	16	21
-CMYK	2.3955	6	1	5
-CPYK	2.462	37	6	23
-CCMR	2.7947	7	1	15
-CEQR	2.7947	14	2	9
