# Supplementary material for: Towards the Systematic Mapping and Engineering of the Protein Prenylation Machinery in Saccharomyces cerevisiae
Source: PLoS One. 2015 Mar 13;10(3):e0120716. doi: 10.1371/journal.pone.0120716 (PMC4358939; doi:10.1371/journal.pone.0120716)
Supplement: S9 File — (PDF) [file pone.0120716.s009.pdf]

CAAX-Box Processing Requirements during Pheromone Maturation Compared to the RRS

- CAAX-box dependent pheromone diffusion data is derived from Figure 2 in Trueblood *et al.*, 2000, MCB, 20, 4381-4392

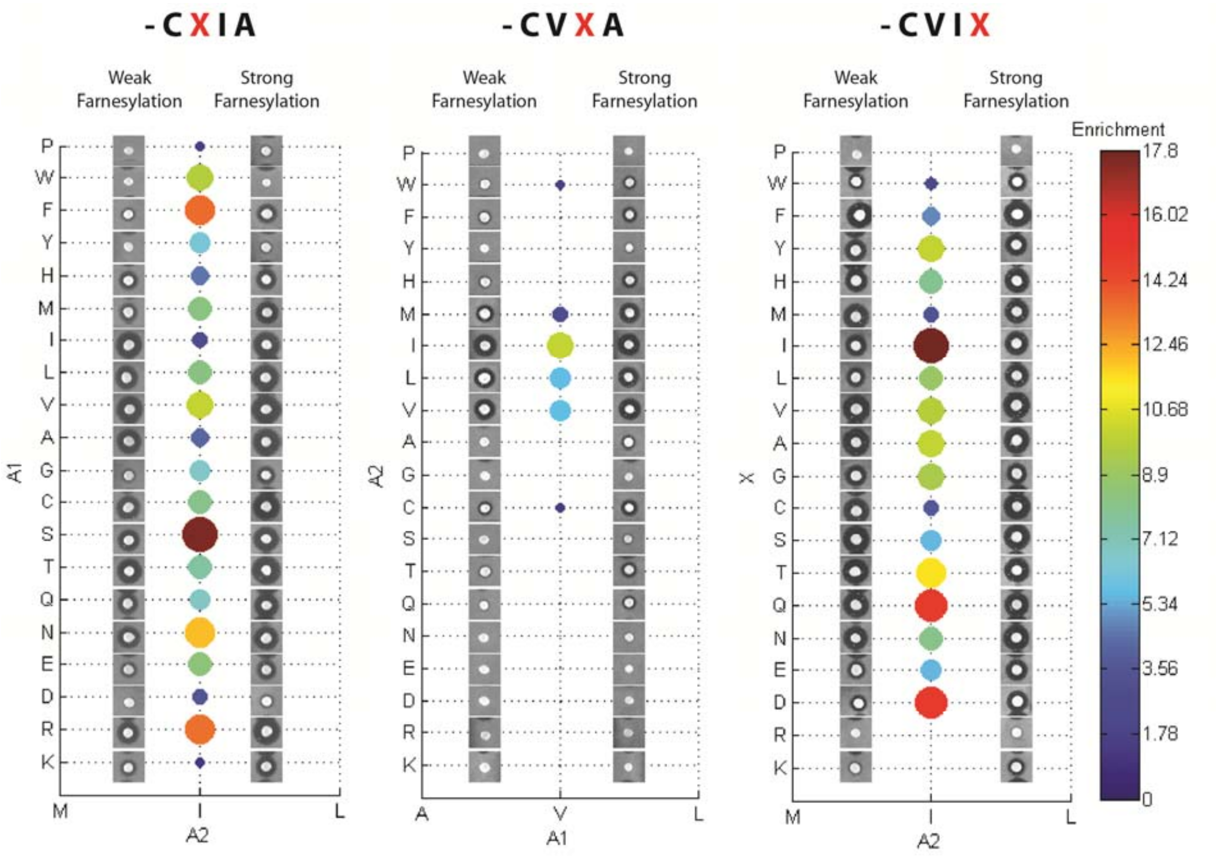

Figure S2: Comparing the CaaX-box specificity requirements for membrane recruitment in the RRS and in the pheromone diffusion assay: A positive read-out in the pheromone diffusion assay (as judged by the formation of dark halo around a yeast colony) strictly depends on farnesylation as well as proteolytic processing of **a**-factor. To dissect the substrate specificity requirements for **a**-factor maturation, systematic sequence-structure-function have previously been conducted in strains with weak farnesylation activity (based on the W303 background that features a less active FTase mutant β-FTase^{H83Y}) and strong farnesylation activity where wild-type β-FTase was additionally expressed from a high-copy plasmid. See Trueblood et al, 2000, MCB, 20, 4381-3992 for details. A comparison of the two data sets shows that the CaaX-box dependent membrane recruitment in the RRS and **a**-factor maturation impose similar substrate specificity requirements in the a_2 and X positions. Differences are primarily observed for a_1 where the substrate specificity is more relaxed for a positive read-out in the RRS compared to in the pheromone diffusion assay. In particular, bulky hydrophobic residues are enriched in the RRS, but not in the pheromone diffusion assay. The difference could be due to a relaxed requirement for proteolytic processing in the RRS which is strictly necessary for the function of the **a**-factor while bulky, hydrophobic residues confer sufficient membrane affinity to enable a positive read-out in the RRS.

1. Enrichment Factors of Sequencing Motives Ending with -CXIA

Motif	Enrichment	Counts 37 °C	25 °C	Naive
-CKIA	1.254	44	14	10
-CPIA	1.597	28	7	17
-CIIA	2.935	125	17	24
-CDIA	3.513	132	15	23
-CAIA	4.232	212	20	35
-CHIA	4.551	57	5	6
-CYIA	6.247	266	17	22
-CGIA	6.787	34	2	9
-CQIA	6.844	120	7	19
-CTIA	7.563	341	18	40
-CCIA	8.051	121	6	18
-CLIA	8.239	227	11	32
-CMIA	8.284	166	8	16
-CEIA	8.441	148	7	11
-CWIA	9.696	170	7	20
-CVIA	10.12	355	14	40
-CNIA	12.06	544	18	19
-CRIA	13.48	473	14	26
-CFIA	13.57	204	6	20
-CSIA	17.51	351	8	41

2. Enrichment Factors of Sequencing Motives Ending with -CVXA

Motif	Enrichment	Counts 37 °C	25 °C	Naive
-CVEA	0.030	1	13	24
-CVRA	0.033	2	24	77
-CVTA	0.033	1	12	38
-CVKA	0.036	1	11	14
-CVGA	0.042	2	19	20
-CVSA	0.057	2	14	19
-CVAA	0.079	1	5	20
-CVQA	0.079	1	5	20
-CVPA	0.099	2	8	25
-CVNA	0.332	5	6	31
-CVYA	0.399	1	1	26
-CVDA	0.548	11	8	13
-CVFA	0.798	2	1	23
-CVHA	0.798	4	2	16
-CVCA	1.397	28	8	27
-CVWA	1.597	4	1	22
-CVMA	2.951	207	28	24
-CVLA	5.737	388	27	29
-CVVA	5.781	362	25	45
-CVIA	10.12	355	14	40

3. Enrichment Factors of Sequencing Motives Ending with -CVIX

		Counts		
**Motif**	**Enrichment**	**37 °C**	**25 °C**	**Naive**
-CVIK	0.133	1	3	23
-CVIR	0.159	8	20	46
-CVIP	0.332	5	6	27
-CVIW	2.395	30	5	11
-CVIM	3.371	152	18	19
-CVIC	3.811	105	11	11
-CVIF	4.899	135	11	11
-CVIE	5.605	351	25	41
-CVIS	5.635	494	35	53
-CVIH	7.984	80	4	9
-CVIN	8.144	102	5	15
-CVIL	8.912	692	31	51
-CVIG	9.473	261	11	19
-CVIV	9.781	147	6	23
-CVIA	10.12	355	14	40
-CVIY	10.14	127	5	10
-CVIT	11.57	87	3	21
-CVIQ	14.93	187	5	17
-CVID	15.01	376	10	18
-CVII	17.79	312	7	23
